# Cross-Trait Genetic Analyses Indicate Pleiotropy and Complex Causal Relationships between Headache and Thyroid Function Traits

**DOI:** 10.3390/genes14010016

**Published:** 2022-12-21

**Authors:** Sana Tasnim, Scott G. Wilson, John P. Walsh, Dale R. Nyholt

**Affiliations:** 1 Statistical and Genomic Epidemiology Laboratory, School of Biomedical Sciences, Faculty of Health, and Centre for Genomics and Personalised Health, Queensland University of Technology, Brisbane, QLD 4059, Australia; 2 Department of Endocrinology and Diabetes, Sir Charles Gairdner Hospital, Nedlands, WA 6009, Australia; 3 School of Biomedical Sciences, University of Western Australia, Nedlands, WA 6009, Australia; 4 Department of Twin Research and Genetic Epidemiology, King’s College London, London SE1 7EH, UK; 5 Medical School, University of Western Australia, Nedlands, WA 6009, Australia

**Keywords:** headache, thyroid traits, GWAS, cross-trait meta-analysis, pairwise-GWAS, gene-based analysis, causal relationship

## Abstract

Epidemiological studies have reported a comorbid relationship between headache and thyroid traits; however, little is known about the shared genetics and causality that contributes to this association. We investigated the genetic overlap and associations between headache and thyroid function traits using genome-wide association study (GWAS) data. We found a significant genetic correlation (*r*_g_) with headache and hypothyroidism (*r*_g_ = 0.09, *p* = 2.00 × 10^−4^), free thyroxine (fT4) (*r*_g_ = 0.08, *p* = 5.50 × 10^−3^), and hyperthyroidism (*r*_g_ = −0.14, *p* = 1.80 × 10^−3^), a near significant genetic correlation with secondary hypothyroidism (*r*_g_ = 0.20, *p* = 5.24 × 10^−2^), but not with thyroid stimulating hormone (TSH). Pairwise-GWAS analysis revealed six, 14, four and five shared (pleiotropic) loci with headache and hypothyroidism, hyperthyroidism, secondary hypothyroidism, and fT4, respectively. Cross-trait GWAS meta-analysis identified novel genome-wide significant loci for headache: five with hypothyroidism, three with secondary hypothyroidism, 12 with TSH, and nine with fT4. Of the genes at these loci, six (*FAF1*, *TMX2-CTNND1*, *AARSD1*, *PLCD3*, *ZNF652*, and *C20orf203*; headache-TSH) and six (*HMGB1P45*, *RPL30P1*, *ZNF462*, *TMX2-CTNND1*, *ITPK1*, *SECISBP2L*; headache-fT4) were significant in our gene-based analysis (*p*_Fisher’s combined *p*-value_ < 2.09 × 10^−6^). Our causal analysis suggested a positive causal relationship between headache and secondary hypothyroidism (*p* = 3.64 × 10^−4^). The results also suggest a positive causal relationship between hypothyroidism and headache (*p* = 2.45 × 10^−3^) and a negative causal relationship between hyperthyroidism and headache (*p* = 1.16 × 10^−13^). These findings suggest a strong evidence base for a genetic correlation and complex causal relationships between headache and thyroid traits.

## 1. Introduction

Headache is one of the most common neurological symptoms affecting the central nervous system, causing pain in the head, neck, and shoulders. Its overall estimated global prevalence accounts for 52% of the general population [1]. It affects people of all ages, races, and socioeconomic statuses with the highest prevalence in the age group of 10 to 19 years and affecting a greater proportion of females than males (57.7% vs. 44.4%) [1,2]. 

Headache disorders are classified into two major categories: primary headache—when associated with no underlying cause, and secondary headache—when associated with an underlying medical illness [2,3]. Primary headaches mainly include migraine, tension-type, and cluster headaches, while secondary headaches include pain caused due to trauma, tumors, ischemic or hemorrhagic lesions and inflammatory or infectious diseases. Over recent years, studies have been conducted on understanding the possible links between headache and immunological/autoimmune diseases [3]. On these grounds, several epidemiological and case–control studies have indicated a comorbid relationship between headache with thyroid function and dysfunction [4,5,6,7,8]. Thyroid dysfunction is the second most common endocrine disorder with a global prevalence of 2 to 6% which negatively influences the quality of life [9]. Studies have reported that women with hypothyroidism have an increased risk of severe preeclampsia (odds ratio [OR] = 1.6) as well as hypertension (OR = 1.75) [10,11]. Hyperthyroidism has been reported to be associated with an increased risk of developing atrial fibrillation (OR = 37.4) and hypertension (risk ratio = 1.64) [12,13].

The endocrine diseases studied with respect to the pathological processes within the thyroid gland are related to the two most common thyroid dysfunction traits, hypothyroidism, and hyperthyroidism. However, if the pathological processes occur within the hypothalamus or pituitary gland, it results in a rare thyroid dysfunction trait, secondary hypothyroidism [14,15]. These traits are differentiated based on the inverse relationship shared between thyroid hormone levels, thyroid stimulating hormone (TSH) and free thyroxine (fT4) [16]. Hypothyroidism relates to an increase in TSH levels with reduced fT4 levels; hyperthyroidism relates to reduced TSH levels with an increase in fT4 levels; secondary hypothyroidism relates to low fT4 levels with inappropriately low TSH levels [14,15]. These traits have been associated with an increased prevalence among headache cases and vice versa [4,6,7,17]. For example, a case–control study reported the incidence of hypothyroidism to be 8.2% in the headache population and 6.2% in the general population with a hazard ratio of 1.210 (95% CI = 1.01–1.46). The study also found headache cases have a 21% increased risk of developing hypothyroidism [4].

Despite observational epidemiological evidence, a clear interpretation of the increased co-occurrence of headache and thyroid traits is lacking, including the role of genetic factors in their comorbidity. Thus in the present study, we utilise genome-wide association studies (GWAS) summary statistics to investigate the genetic overlap between headache and thyroid traits including thyroid dysfunction traits, hypothyroidism, hyperthyroidism, and secondary hypothyroidism; and thyroid hormones, TSH and fT4, at both the genome-wide as well as the regional locus level. We also used genetic approaches including cross-trait meta-analysis and gene-level genetic overlap, to identify shared genetic components and related pathophysiology between headache and thyroid traits. Finally, we utilise Mendelian Randomisation (MR) to look for causal relationships that occur between headache and thyroid traits. 

## 2. Materials and Methods

### 2.1. Overview

The genetic association between headache and thyroid traits was examined both at the single nucleotide polymorphism (SNP)-, as well as the gene-level. SNP-level genetic association was calculated using four approaches. We first estimated the SNP-level genetic correlation between headache and each thyroid trait using the linkage disequilibrium score regression (LDSC) analysis. Next, to access genetic correlation within segments across the genome we performed pairwise-GWAS (GWAS-PW). We then performed a cross-trait GWAS meta-analysis to identify novel SNPs shared by headache and each thyroid trait. Further, to infer the likely causal relationships of headache with the thyroid traits, we performed the MR analysis. To complement our SNP-level genetic association analysis, we performed a gene-based analysis to identify genome-wide significant (GWS) genes shared by each of the headache and thyroid trait combinations and then further assess the gene-level genetic overlap between the headache and each thyroid trait. Finally, we conducted pathway analysis to delve into the biological insights shared between headache and each thyroid trait.

### 2.2. Summary Statistics

The GWAS summary statistics for broadly defined headache and thyroid dysfunction traits (hypothyroidism, hyperthyroidism, and secondary hypothyroidism) were obtained from PANUK Biobank which can be publicly accessed at https://pan.ukbb.broadinstitute.org/ (accessed on 29 August 2020) [18], while TSH and fT4 were obtained from the Teumer et al. (2018) study which can be publicly accessed at https://transfer.sysepi.medizin.uni-greifswald.de/thyroidomics/datasets/ (accessed on 29 January 2019) [19]. The PANUK Biobank GWAS summary statistics have been generated through electronic medical records as well as participants’ responses to questionnaires provided to them, online (self-reported) or at the clinic (interviewed by a trained nurse) [18]. Headache cases have been defined as the ones who experienced a headache in the last month which interfered with their usual activities [18], whereas thyroid trait cases (hypothyroidism, hyperthyroidism, and secondary hypothyroidism) have been defined based on the varying levels of thyroid hormones (TSH and fT4) when its measurement was outside of the reference range [18].

The headache GWAS summary statistics were obtained from the PANUK Biobank for ‘headache pain experienced in last month’ comprising of 84,036 cases and 335,552 controls [18]. The GWAS summary statistics for thyroid dysfunction traits comprised of 20,563 cases and 399,910 controls for hypothyroidism, 3197 cases and 417,276 controls for hyperthyroidism, and 1430 cases and 399,034 controls for secondary hypothyroidism. The PANUK Biobank GWAS phenocode 6159_1 was used to determine headache experienced in the last month, 20002_1225 was used to determine non-cancer illness code for hyperthyroidism, 20002_1226 was used to determine non-cancer illness code for hypothyroidism, and 244.1 was used to determine secondary hypothyroidism [18]. The detailed descriptions of the headache GWAS is available at https://biobank.ctsu.ox.ac.uk/crystal/field.cgi?id=6159, and hypothyroidism and hyperthyroidism GWAS are available at http://biobank.ctsu.ox.ac.uk/crystal/field.cgi?id=20002. The TSH and fT4 GWAS summary statistics were obtained from a GWAS of up to 72,167 individuals (meta-analysing 22 and 19 independent cohorts for TSH and fT4, respectively) [19]. Because there were 22 different studies there was a slight variation in the cut-off values across some of the studies, but they range from 0.4 to 4.0 mU/L for TSH and 11.5 to 20.8 pmol/L for fT4. Further descriptions of the included cohorts, quality control, and statistical analysis performed are available from the original publication [19]. These are the largest and most powerful known GWAS for these traits. All summary statistics utilised in our analyses were based on hg19 and were from European ancestry.

The confounding factors that may influence the thyroid status (physiological changes, for example, age and pregnancy; intercurrent (non-thyroidal) illness or with a history of thyroid surgery; and medication use, for example, thyroxine, amiodarone, heparin) were excluded from all analysis. Additionally, confounding factors of age and socio-economic burden exists for datasets accessed from PANUK Biobank. However, GWAS have been performed in individuals with only European ancestries and thus the associations are less likely to be driven by these non-causal factors. Most importantly, the consequence of these and other potential confounding factors is to introduce ‘noise’ which reduces the power of the individual GWAS to detect association to the respective trait. That is, confounding factors do not create post-positive genetic association signals in the individual trait GWAS, nor do they create false positive cross-trait relationships.

### 2.3. Genome-Wide Genetic Correlation

To estimate SNP-based heritability (*h*^2^) between headache and thyroid traits and to assess genome-wide genetic correlation (*r*_g_) between them, we performed cross-trait linkage disequilibrium (LD) score regression (LDSC) (https://github.com/bulik/ldsc (accessed on 13 September 2021)) [20]. LDSC analyses were performed on the liability scale and LDSC-provided pre-calculated European LD scores from the 1000 Genomes Project phase 3 (1000 G) EUR reference sample. 

We first estimated *h*^2^ for headache and hypothyroidism, hyperthyroidism, secondary hypothyroidism, TSH, and fT4; then assessed for cross-trait genetic correlation between headache and each thyroid trait. Because of overlapping PANUK Biobank samples between headache and hypothyroidism, hyperthyroidism, and secondary hypothyroidism, we performed both constrained and unconstrained (genetic covariance intercept) genetic correlation analyses. Additionally, to deal with the multiple testing burden, Bonferroni correction was used to adjust *p*-values across all trait combinations (*p* < 0.01).

### 2.4. Local Genetic Correlation between the Traits

We performed local genetic correlation to identify shared genomic regions influencing headache and thyroid traits using pairwise-GWAS (GWAS-PW) (https://github.com/joepickrell/gwas-pw (accessed on 12 April 2022)) software v0.21 by Pickrell et al. [21]. GWAS-PW estimates local (regional) genetic correlations between a pair of traits in 1703 pre-defined LD-independent segments using Z scores and variance of effect size for each SNP. It then estimates the four posterior probabilities of association (PPA) of genomic regions shared across headache and thyroid traits: (i) association with headache only (PPA1); (ii) association with a thyroid trait only (PPA2); (iii) shared association with both headache and thyroid trait via a SNP (PPA3); shared association with both headache and thyroid trait but via two distinct SNPs (PPA4). For headache and thyroid traits GWAS with overlapping samples, their genetic correlation (as calculated by LDSC) was used to specify the expected correlation in summary statistics under the null. To estimate the genetic overlap, we considered PPA3 > 0.5 to identify pleiotropic regions across headache and thyroid traits via a shared SNP.

### 2.5. Cross-Trait GWAS Meta-Analysis and Its Characterization

We conducted a genome-wide cross-trait GWAS meta-analysis of headache and thyroid traits to identify shared loci using the METASOFT software (http://genetics.cs.ucla.edu/meta (accessed on 4 January 2022)) (https://www.dropbox.com/s/9h7p22rmwxi6wbd/Metasoft.zip?dl=0 (accessed on 4 January 2022)) inverse variance-weighted (IVW) fixed effect (FE) model [22]. This method uses SNP effect size estimate (*β*) and their estimated standard errors (SE) to calculate their meta-analysed *p*-value. The METASOFT Han and Eskin’s random-effects (RE2) model was also utilised to allow for heterogeneity effects observed across traits [22]. A meta-analysed SNP with a *p*-value < 5 × 10^−8^ was considered to be GWS, while SNP with a *p*-value < 0.05 was considered to be nominally significant. To be considered as novel SNP/loci, SNPs should have a meta-analysed GWS *p*-value (*p*_meta-analysed_ < 5 × 10^−8^) but should not be GWS in either of the two traits (5 × 10^−8^ < *p*_headache/thyroid trait_ < 0.05).

Following meta-analyses, the SNPs were annotated using the FUMA software (https://fuma.ctglab.nl/) to characterise the SNPs and loci associated with headache and thyroid traits [23]. The meta-analysed SNPs were first filtered based on their association with headache and thyroid traits, i.e., with *p*-values of both traits in the range of 5 × 10^−8^ < *p*_headache/thyroid trait_ < 0.05. The remaining GWS SNPs were used as input to FUMA. FUMA then performed LD clumping to identify regions containing SNPs that are correlated (in LD) with each other at *r*^2^ < 0.6, and independent lead SNPs that are not in LD with each other at *r*^2^ < 0.1.

A six-step procedure was followed to identify novel loci for headache and a thyroid trait, that were LD-independent to GWS SNPs in either of the two traits. As an example, we detail the following steps to identify novel loci from a cross-trait meta-analysis of headache and hypothyroidism: (1) LD clumping was performed on 972 GWS SNPs (with *p*_meta_ < 5 × 10^−8^ and trait 5 × 10^−8^ < *p*_headache,hypothyroidism_ < 0.05) using FUMA through which 22 SNPs were identified as lead SNPs (*r*^2^ < 0.1); (2) a list with the observed *p*-value of the 22 LD-independent GWS index SNPs obtained from step 1 changed to a non-GWS *p*-value was prepared; (3) a list of all GWS SNPs from the headache GWAS was prepared; (4) a list of all GWS SNPs from the hypothyroidism GWAS was prepared; (5) after combining lists from steps 2, 3, and 4, LD clumping was performed using FUMA to obtain LD-independent GWS index SNPs which identified 279 index SNPs; and (6) we overlapped SNP lists in steps 2 and 5, to obtain GWS index SNPs from meta-analysis of headache and hypothyroidism which were LD-independent to the GWS SNPs listed in steps 3 and 4.

### 2.6. Causal Analyses between Headache and Thyroid Traits

To investigate the causal relationship between headache and thyroid traits, we performed Mendelian Randomisation (MR) analysis. We estimated their bidirectional causal relationships using two-sample Mendelian randomisation (2SMR) analysis in R statistical package (https://mrcieu.github.io/TwoSampleMR/) [24]. First, we tested for a causal effect of each thyroid trait as the exposure on headache as the outcome. Second, we performed reverse MR analyses to test for a causal relationship between genetic risk for headache as the exposure and each thyroid trait as the outcome. For 2SMR analysis, LD-independent GWS SNPs at a *p*-value < 5 × 10^−8^ (*r*^2^ < 0.1), except for secondary hypothyroidism for which the threshold was lowered to *p*-value < 1 × 10^−5^ (*r*^2^ < 0.1) (due to a small number of GWS SNPs) were utilised as instrumental variables (IVs). We performed 2SMR analyses using the inverse variance weighted (IVW), the weighted median, and the MR-Egger method [25,26]. To further test for horizontal pleiotropy (considered to be a violation of MR assumptions) and possible outliers, we conducted the Mendelian randomisation pleiotropy residual sum and outlier (MR-PRESSO) analysis [27]. Then, to further validate our results, we performed Cochran’s Q statistics for heterogeneity test, Wald ratio for single SNP MR analysis and IVW for ‘leave-one-out’ analysis.

Another bi-directional Generalised Summary-data based Mendelian randomisation (GSMR) MR analysis was performed using GSMR software [28]. This analysis estimates the effect of LD-independent GWS SNPs on summary statistics of a thyroid trait (b_zx_) and headache (b_zy_) to estimate the causal association (b_xy_) between a thyroid trait (exposure) and headache (outcome) and vice versa. To exclude putative pleiotropic SNPs from the analysis, the GSMR package also uses the HEIDI outlier method (HEIDI-outlier *p*-value < 0.01). Similar to 2SMR, the LD-independent GWS SNPs for headache and thyroid traits were selected at *p*-value < 5 × 10^−8^ (*r*^2^ < 0.1), except for secondary hypothyroidism for which the threshold was lowered to *p*-value < 1 × 10^−5^ (*r*^2^ < 0.1) due to a small number of GWS SNPs.

Additionally, to test for a causal relationship at the genome-wide level we performed latent causal variable (LCV) analysis which estimates the genetic causality proportion (gcp) of each thyroid trait on headache [29]. It is based on a latent variable mediating the genetic correlation between the two traits, where a gcp of zero would mean no genetic causality and a gcp of one would mean full genetic causality. The LCV analysis was performed on the GWAS summary statistics of each trait and pre-calculated LD scores from 1000 G accompanying the LDSC software.

### 2.7. Gene-Based Association Study

To further confirm our SNP-level genetic overlap analysis and identify shared genes across headache and thyroid traits, we performed gene-based association analysis. Our gene-based analysis was conducted using the GATES test [30] implemented in the Fast ASsociation Tests (FAST) [31] package. The common SNPs between headache and thyroid traits were assigned to 34,212 genes from NCBI if they mapped between 15 kb 5′ of the transcription start site (TSS) and 15 kb 3′of the transcription end site (TES). This 15-kb gene boundary around the TSS and TES was applied based on the observation that 90% of SNPs affecting expression quantitative trait loci (eQTLs) were within this proximity [32]. Gene-based test are performed by adjusting the observed *p*-value of the most significant SNP assigned to a gene by the total effective number of independent SNPs tested across the gene estimated from an eigenvalue analysis of the n × n SNP LD correlation matrix (estimated from the 1000 Genomes Project [released on May 2012] CEU reference population) [30]. The output of GATES provides us with the best significant SNP assigned to a gene with their respective *p*-values. We note that neighbouring genes may have correlated results due to LD between the topmost significant SNP assigned to each gene.

### 2.8. Independent Gene-Based Test

We performed independent gene-based analysis using the ‘genetic type I error calculator’ GEC software [33] as implemented in previous studies [34,35] to estimate the effective number of independent genes across headache and thyroid traits. The best SNPs assigned to genes across headache and thyroid traits GWAS were used as input for GEC analysis. GEC was utilised (i) to overcome the potential for correlation across neighbouring gene-based association results; and (ii) to generate unbiased data for assessing the gene-level genetic overlap between headache and thyroid traits.

### 2.9. Gene-Level Genetic Overlap

Further analysing the effective number of independent genes obtained using independent gene-based association results, we assessed whether the proportion of genes overlapping headache and thyroid traits, at three nominal *p*-value thresholds (*p* < 0.1, *p* < 0.05, *p* < 0.01), were more than expected by chance. 

To test for gene-level genetic overlap between headache and hypothyroidism, we first estimated the effective number of independent genes overlapping headache and hypothyroidism within the three *p*-value thresholds. We then assigned ‘headache’ as the discovery dataset and ‘hypothyroidism’ as the target dataset, to calculate the proportion of expected as well as observed genes overlapping both traits using GEC analysis. The expected proportion of overlapping genes across headache and hypothyroidism was defined as the effective number of independent genes with a *p*-value less than the threshold in the target dataset divided by the total effective number of independent genes in the target dataset. The observed proportion of overlapping genes across headache and hypothyroidism was defined as the observed effective number of independent overlapping genes divided by the effective number of independent genes with a *p*-value less than the threshold in the discovery dataset. Finally, using a one-sided binomial test, we test the statistical significance of the observed proportion of overlapping genes against the expected proportion of genes overlapping headache and hypothyroidism for the three *p*-value thresholds. A significant binomial test *p*-value indicates that the observed number of overlapping genes was more than expected by chance.

A similar analysis was performed to test for gene-level genetic overlap between headache and hyperthyroidism, secondary hypothyroidism, TSH, and fT4.

To identify individual genes associated with headache and thyroid traits at a *p* < 0.05, we combined the gene-based *p*-value for the respective traits using Fisher’s combined *p*-value method (FCP) [34,35]. We then assessed the shared genes for both headache and thyroid traits reaching genome-wide significance based on our FCP results.

### 2.10. Pathway Analysis

To identify potential biological mechanisms and pathways related to the overlapping genes of headache and thyroid traits, we performed functional enrichment analysis using ‘g:GOst’ tool implemented in the g:Profiler web server (http://biit.cs.ut.ee/gprofiler/ (accessed on 1 August 2022).) [36]. The tool covers data sources including Gene Ontology, Reactome, WikiPathways, Kyoto Encyclopedia of Genes (KEGG), Human Protein Atlas, CORUM, and Human Phenotype Ontology, updated on a regular basis [36]. In the present study, to identify shared pathways and mechanisms, we utilised genes overlapping headache and thyroid traits at *p* < 0.05 (FCP < 1 × 10^−4^). We applied the recommended ‘g:SCS algorithm’ to adjust for multiple testing and restricted the term size of the pathways to 5 and 350. In case the pathways were biased due to the presence of LD across the neighbouring genes, we ensured that the top SNPs of the genes in any respective pathway were not in LD (*r*^2^ < 0.1). Enrichmentmap within Cytoscape was utilised to interpret the enriched pathways [37].

## 3. Results

### 3.1. Univariate SNP Heritability across Headache and Thyroid Traits

The SNP-based heritability (*h*^2^_SNP_) estimates from LDSC analysis of the headache and thyroid traits GWAS summary statistics are reported in Supplementary Table S1. Headache and all five thyroid traits were found to have significant (*p* < 0.05) *h*^2^_SNP_ and were therefore utilised in subsequent cross-trait genetic analysis.

### 3.2. Genetic Correlation between Headache and Thyroid Traits

Using LDSC, we found a significant positive genetic correlation between headache and hypothyroidism (*r*_g_ = 0.09, *p* = 2.00 × 10^−4^), and headache and fT4 (*r*_g_ = 0.08, *p* = 5.50 × 10^−3^). We also found a significant negative genetic correlation between headache and hyperthyroidism (*r*_g_ = −0.14, *p* = 1.80 × 10^−3^). Additionally, although not significant we found a positive genetic correlation between headache and secondary hypothyroidism (*r*_g_ = 0.20, *p* = 5.24 × 10^−2^). However, we did not find a significant correlation between headache and TSH (*r*_g_ = −0.04, *p* = 0.26). LDSC indicated a small amount of sample overlap for headache with hypothyroidism, hyperthyroidism, and secondary hypothyroidism, although the genecov intercept were very close to zero (0.0160, −0.0038, and 0.0061, respectively) and when constrained to zero had a negligible impact on the results. Detailed SNP-based genetic correlation results are provided in Table 1.

### 3.3. Shared Local Variants between Headache and Thyroid Traits

We used the GWAS-PW approach to identify pleiotropic loci associated in both the headache and thyroid traits GWAS. A pleiotropic locus in a region identifies SNPs associated with both traits regardless of the SNPs’ direction of effect. Similar to the LDSC genome-wide genetic correlation results, applying GWAS-PW analysis to headache and TSH trait combination, none of the 1703 tested genomic regions had a PPA3 > 0.5, indicating that no genomic region contained a SNP that was strongly associated with both headache and TSH.

**Figure 1:**
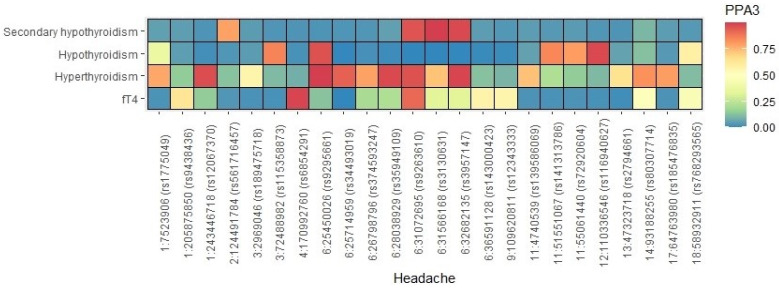
GWAS-PW comparisons with headache and thyroid traits. Pleiotropic loci represented by the lead (most significant) SNP (and its chromosome and base pair position) reaching a PPA3 > 0.5 from GWAS-PW analysis of headache and thyroid traits (hypothyroidism, hyperthyroidism, secondary hypothyroidism, and fT4). SNP, single nucleotide polymorphism; GWAS-PW, pairwise GWAS; PPA3, posterior probability of association.

When applying the GWAS-PW approach to headache and hypothyroidism, hyperthyroidism, secondary hypothyroidism, and fT4, we identified significant GWS local genetic correlation at six loci across five chromosomes, 14 loci across seven chromosomes, four loci across two chromosomes and five loci across four chromosomes (PPA3 > 0.5), respectively (Figure 1, Supplementary Tables S2–S5). When examining the direction of the effect allele of the six, 14, and five index SNPs at the pleiotropic loci in headache and hypothyroidism, hyperthyroidism, and fT4, respectively, the effect allele for four, and 12 index SNPs were associated with an increased risk for hypothyroidism and hyperthyroidism being associated with an increased risk for headache, while the effect allele for all five index SNPs were associated with increased fT4 levels being associated with an increased risk for headache (Supplementary Tables S2, S3, and S5). Interestingly, when examining the direction of the effect allele of the four index SNPs at the pleiotropic loci in headache and secondary hypothyroidism, the effect allele for all four index SNPs were associated with a reduced risk for secondary hypothyroidism being associated with an increased risk for headache (Supplementary Table S4). Supplementary Tables S2–S5 list the SNPs that are significant for headache and hypothyroidism, hyperthyroidism, secondary hypothyroidism, and fT4, within the implicated genomic regions.

### 3.4. Shared Loci between Headache and Thyroid Traits

A genome-wide cross-trait meta-analysis approach, METASOFT, was utilised to identify loci that may share an association with headache and thyroid traits. We selected SNPs with a meta-analyses *p*-value < 5 × 10^−8^ and trait specific 5 × 10^−8^ < *p*_headache/thyroid trait_ < 0.05.

Applying the approach detailed in the methods section identified five, three, 12, and nine novel GWS loci from the meta-analysis of headache and hypothyroidism, headache and secondary hypothyroidism, headache and TSH, and headache and fT4, respectively (Table 2).

Among the identified novel loci throughout the headache and thyroid trait combinations, the strongest and most significant association was at chromosome 14, rs12883201 (*p*_meta_ = 4.55 × 10^−10^) for headache and fT4, located near *ITPK1*. Interestingly, this gene was also associated with headache and secondary hypothyroidism (lead SNP: rs28540738, *p*_meta_ = 2.57 × 10^−8^). The second strongest association was at chromosome 17, rs4986170 (*p*_meta_ = 5.20 × 10^−10^) for headache and TSH, located near *PLCD3*.

### 3.5. Causal Relationships

To decipher the potential causal relationships between headache and each thyroid trait, we conducted 2SMR, GSMR, and LCV analyses.

When testing for a causal effect of each thyroid trait on headache, we utilised 229, 22, 39, 61, and 31 independent GWS SNPs as IVs for hypothyroidism, hyperthyroidism, secondary hypothyroidism, TSH, and fT4 GWAS, respectively. 2SMR analyses found evidence for a significant causal effect of hypothyroidism on headache with weighted median (*β* = −0.02, *p* = 1.99 × 10^−2^); hyperthyroidism on headache with IVW (*β* = −0.04, *p* = 4.76 × 10^−2^), weighted median (*β* = −0.03, *p* = 8.71 × 10^−3^), and MR-Egger (*β* = −0.09, *p* = 4.13 × 10^−2^); and TSH on headache with MR-PRESSO (*β* = 0.03, *p* = 3.88 × 10^−2^). The MR-Egger intercept for hypothyroidism on headache (intercept = −0.001, *p* = 0.93), hyperthyroidism on headache (intercept = 0.015, *p* = 0.17), and TSH on headache (intercept = −0.002, *p* = 0.47) did not deviate significantly from zero, suggesting no evidence for pleiotropy. Applying GSMR analysis found a significant positive causal relationship between hypothyroidism and headache (*β* = 0.01, *p* = 2.45 × 10^−3^) and a significant negative causal relationship between hyperthyroidism and headache (*β* = –0.04, *p* = 1.16 × 10^−13^), however, no causal effect was observed between TSH and headache (Table 3a,b).

In contrast, when testing for a causal effect of headache on each thyroid trait, we utilised 51 independent GWS SNPs from the headache GWAS as IVs. Reverse 2SMR analysis only found a significant causal effect of headache on secondary hypothyroidism with IVW (*β* = 0.37, *p* = 4.19 × 10^−3^), weighted median (*β* = 0.47, *p* = 9.81 × 10^−3^), and MR-PRESSO (*β* = 0.40, *p* = 1.60 × 10^−3^), but not with MR-Egger (*β* = 0.46, *p* = 0.21). The MR-Egger intercept for headache on secondary hypothyroidism (intercept = −0.005, *p* = 0.80) did not deviate significantly from zero, suggesting no evidence for pleiotropy. Similarly, reverse GSMR analyses found a significant positive causal relationship between headache and secondary hypothyroidism (*β* = 0.50, *p* = 3.64 × 10^−4^). Interestingly, the reverse GSMR analyses also found weak evidence for a causal relationship between headache and hyperthyroidism (*β* = 0.19, *p* = 4.89 × 10^−2^), but no evidence for headache on hypothyroidism, TSH, or fT4 (Table 3a,b).

LCV analyses detected no evidence for genome-wide causality between headache and any thyroid trait (Table 3c).

### 3.6. Gene-Level Genetic Overlap

We performed gene-level genetic overlap analysis to identify and assess the proportion of associated genes overlapping the headache and each thyroid trait GWAS. To determine if the proportion of overlapping genes was more than expected by chance, we performed binomial tests for genes associated at three *p*-value thresholds (Table 4, Supplementary Tables S6–S9). For instance, when analysing headache and hypothyroidism at *p*_gene_ < 0.05, the observed proportion (0.291) of overlapping associated genes was significantly higher (*p*_binomial_ = 2.83 × 10^−55^) than the empirically derived expected proportion (0.183). A similar pattern of results was observed at the other two thresholds (*p*_gene_ < 0.01 and *p*_gene_ < 0.1). 

All genes overlapping headache and each thyroid trait GWAS are reported in Supplementary Tables S10–S14. A gene was considered to be GWS at FCP *p*_gene_ < 2.09 × 10^−6^ adjusted for testing the estimated maximum number of independent gene-based tests (0.05/23,957). Combining gene-based tests for association across headache and each thyroid trait using the FCP method, we identified 802 unique genes showing evidence of being shared by both traits in the present study.

### 3.7. Pathway Analysis of Overlapping Genes

We performed pathway analysis using the g:GOst tool implemented in the g:Profiler software of overlapping genes associated with headache and each thyroid trait at a *p*_gene_ < 0.05 threshold.

**Figure 2:**
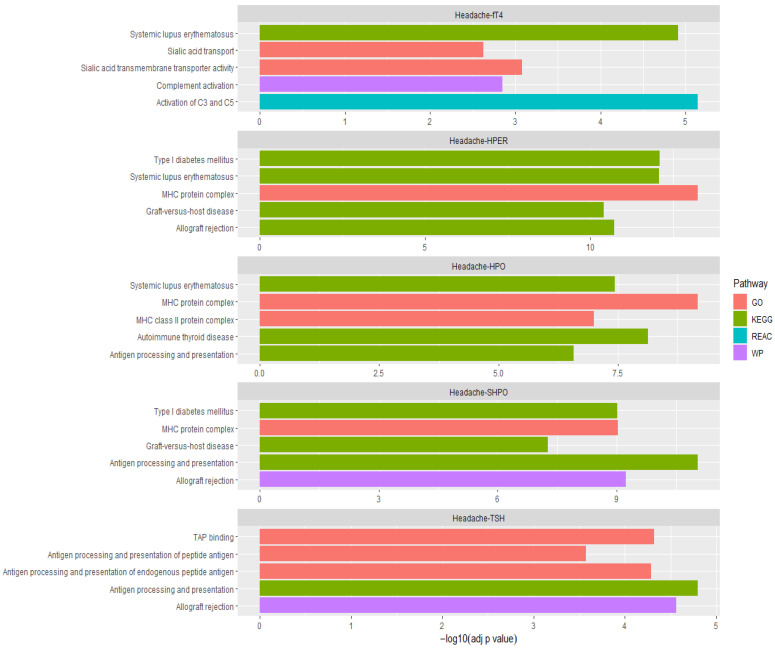
Top five significantly enriched pathways (GO, KEGG, REAC, and WP) across all headache and thyroid traits. (HPO, hypothyroidism; HPER, hyperthyroidism; SHPO, secondary hypothyroidism; TSH, thyroid stimulating hormone; fT4, free thyroxine; GO, Gene Ontology; KEGG, Kyoto Encyclopedia of Genes; REAC, Reactome; WP, Wiki Pathways).

**Figure 3:**
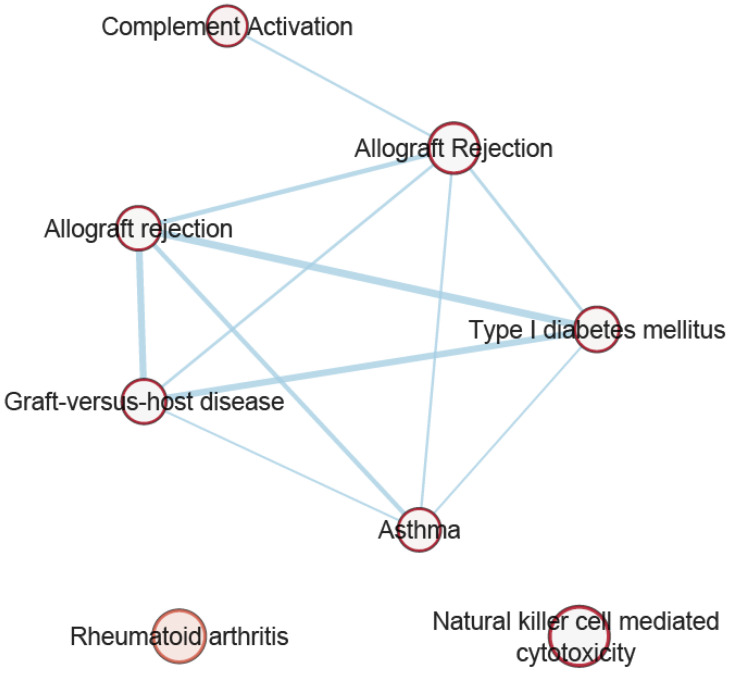
The network of the common pathways identified from pathway analysis. Node (circle) represents gene sets; node size represents number of genes within the gene sets; edges (connecting lines) represent overlap between gene sets; edge width represents number of genes that overlap between a pair of gene sets.

After retaining the most significant gene within sets of correlated genes with their top significant SNPs in LD (*r*^2^ < 0.1), pathway analysis was performed for 482, 226, 69, 170, and 246 genes associated with *p*_FCP_ < 1 × 10^−4^ overlapping headache with hypothyroidism, hyperthyroidism, secondary hypothyroidism, TSH, and fT4, respectively. Supplementary Tables S15–S19 present our findings from pathway analyses identifying 110, 71, 71, 34, and 19 biological pathways/processes enriched for the overlapping genes. Figure 2 illustrates the top five significantly enriched pathways (GO, KEGG, REAC, WP) across all headache and thyroid trait combinations. Some of the significantly enriched pathways include allograft rejection, inflammatory bowel disease (IBD), systemic lupus erythematosus (SLE), type 1 diabetes mellitus (T1D), autoimmune thyroid disease (AITD), rheumatoid arthritis (RA), and involvement of complement component 3 (C3), C4, and C5 proteins of the immune system. Figure 3 illustrates the network of the common pathways identified from pathway analysis. Additionally, of interest were the genes involved in the pathways of *Staphylococcus aureus*, leukemia virus 1 infection and coronavirus disease (COVID-19).

## 4. Discussion

Many observational epidemiological and cross-sectional association studies have been performed to understand the relationship between headache and thyroid traits and have reported their increased co-occurrence. A recent GWAS study in a global headache cohort reported 28 genetic loci associated with headache. They also highlighted that 14 of these 28 loci identified were previously reported for migraine suggesting these loci to be involved in broader headache pathways [38]. GWAS studies have also been performed to identify genetic loci associated with thyroid traits. Thus, several SNPs and genes have been identified to be associated with headache and/or thyroid traits. Here, we perform the first known genetic study to investigate the genetic and causal relationship between headache and thyroid traits. Utilising GWAS summary statistics, we found a significant genetic correlation between headache and hypothyroidism, hyperthyroidism, and fT4; and also shared pleiotropic regions between headache and hypothyroidism, hyperthyroidism, secondary hypothyroidism, and ft4. Furthermore, cross-trait meta-analysis and gene-level overlap analysis identified a shared genetic basis underlying headache and thyroid traits. Our causal analysis found a significant causal relationship between headache and secondary hypothyroidism, hypothyroidism and headache, and hyperthyroidism and headache.

LDSC testing for genome-wide genetic correlation quantifies the average sharing of genetic effects between the two traits [20,39]. The observed positive genetic correlation between headache and hypothyroidism is accordant to the cross-sectional case–control studies previously reported indicating similar conclusions [4,6,7,17]. Neuroimaging results have observed that the hypothalamus is connected to the limbic pathway along with other brain regions [40]. A recent finding reported that in response to pain initiated 48 h prior to the headache attack, increased connectivity between the hypothalamo-limbic system was observed, however, this connectivity was reduced during the actual attack. This suggests that the attack is caused because of a loss of hypothalamus control over the limbic pathway [41]. This could explain the observed positive, although not significant, genetic correlation between headache and secondary hypothyroidism. The positive genetic correlation observed for hypothyroidism and fT4 with headache, although inconsistent with the known inverse relationship between TSH and fT4, could suggest the relationship between the traits being driven by poly(genetic) factors across the genome. The negative genetic correlation observed between headache and TSH is consistent with a study reporting low levels of serum TSH being associated with high prevalence of headache, thus indicating an inverse relationship between TSH levels and headache [8]. The established complex relationship between TSH and fT4 is frequently broken and sometime inverted when their correlation is being studied with common conditions, as observed in our results [42,43]. Our finding of a significant genetic correlation between headache and thyroid traits suggests that shared genetic factors could, in part, be responsible for their comorbidity.

We next identified pleiotropic loci shared across headache and thyroid traits and identified six, 13, four, and five pleiotropic regions influencing both headache and hypothyroidism, hyperthyroidism, secondary hypothyroidism, and fT4, respectively. With the observed pleiotropic regions, four loci, i.e., rs9295661 (headache with hypothyroidism and hyperthyroidism), rs3957147 (headache with hyperthyroidism and secondary hypothyroidism), rs9263610 (headache with hyperthyroidism, secondary hypothyroidism, and fT4), and rs3130631 (headache with hyperthyroidism and secondary hypothyroidism) at chromosome 6 overlapped across the traits.

The novel loci identified in our cross-trait GWAS meta-analysis identified a number of novel loci. Interestingly, some of these loci mapped to genes including *ITPK1*, *ZNF462*, *RPL30P1*, *ANKDD1B*, *THADA*, *ZNF652*, and *C20orf203* that have been reported as genome-wide significant in the most recent migraine GWAS [44]. Other genes of interest were *PLCD3*, *Aff3*, *PDE1C*, and *RSPH6A*. *ITPK1*, (Inositol-tetrakisphosphate 1-kinase 1) (lead SNP: rs12883201 and rs28540738) a protein coding gene, encodes an enzyme that is involved in inositol phosphate metabolism crucial in the development of the neural tube. It also plays a key role in both signalling and metabolic pathways [45]. This gene has previously been reported to be associated with lower levels of TSH (lead SNP: rs11624776) [46]. *THADA*, (thyroid adenoma-associated protein) (lead SNP: rs7590268) a protein coding gene, is the known target of 2p21 chromosomal aberrations in benign thyroid adenomas and the encoded protein is possibly involved in the death receptor pathway as well as apoptosis. *ZNF652* (zinc finger protein 652) (lead SNP: rs6504608) is also a protein coding gene active in the nucleus and is involved in the regulation of transcription by RNA polymerase II. *ITPK1*, *THADA*, and *ZNF652* have also been reported to be GWS in the recent TSH GWAS [47] implying that these genes are important in headache, migraine, and thyroid traits physiology. Mutations in these genes have been reported for disorders such as coronary artery disease, cardiovascular disease, chronic obstructive pulmonary disorder, and endometriosis, and these disorders have been associated with both headache and thyroid traits. This suggests that the novel variants within these genes might have potential effects on headache and thyroid traits comorbidity. Additionally, *PLCD3* (phospholipase C delta 3) (lead SNP: rs4986170) catalyses the hydrolysis of phosphatidylinositol 4,5-biphosphate generating diacylglycerol (DAG) and inositol 1,4,5-triphosphate (IP3) which mediate activation of protein kinase C (PKC) and release of calcium (Ca^2+^), respectively. Although the exact role of this gene in headache and thyroid traits is unknown, it is expressed in both brain as well as thyroid gland tissues.

Our gene-level analysis revealed a significant genetic overlap across headache and the tested thyroid trait (*p*_gene_ < 0.05). Of the genes, mapping to novel loci identified from the cross-trait meta-analysis, six (*FAF1*, *TMX2-CTNND1*, *AARSD1*, *PLCD3*, *ZNF652*, and *C20orf203*; headache and TSH) and six (*HMGB1P45*, *RPL30P1*, *ZNF462*, *TMX2-CTNND1*, *ITPK1*, *SECISBP2L*; headache and fT4) were identified to be GWS in our gene-based analysis (*p*_FCP_ < 2.09 × 10^−6^). *TMX2-CTNND1* is a locus representing a naturally occurring read-through transcription between the two neighbouring genes on chromosome 11, *TMX2* (thioredoxin-related transmembrane protein 2) and *CTNND1* (catenin, cadherin-associated protein, delta 1). The transcript is a candidate for nonsense-mediated mRNA decay (NMD) and thus is unlikely to produce a protein product. *TMX2* has been identified as an important regulator in the developing brain and variants within the gene have been reported to cause severe neurological disorders [48], however, its role in headache or thyroid physiology is still unknown. *HMGB1P45* (high mobility group box 1 pseudogene 45) is a pseudogene that belongs to the *HMGB1*, which encodes a non-histone, nuclear DNA-binding protein that regulates transcription and is involved in DNA organisation. This gene has been considered a promoter in diseases such as cancers and myocardial infarction [49], where these diseases have been previously associated with headache and hypothyroidism [50,51,52,53]. We note that the observed sample prevalence of headache GWAS (20%) utilised in the present study is in line with the tension-type headache population prevalence (26%) [1], noting PANUK Biobank is an unselected population sample. That is, a potential limitation of this study relates to using a broadly defined headache phenotype; however, it is reasonable to assume the vast majority of cases would suffer tension-type headache. The majority of migraine cases (over 90%) have been reported to suffer from tension-type headache [54], and it is thus possible to suggest that migraine and tension-type headache might share common genetic components [38]. Our recent publication provided strong evidence for genetic correlation and causal relationships between migraine and thyroid traits [55]. Thus implying that these overlapping genes are important in headache, migraine, and thyroid traits physiology and might share pathways.

Lastly, pathway analysis utilising overlapping genes for headache and thyroid traits identified some interesting biological pathways/processes. These shared pathways complement our observed evidence of the genetic correlation between headache and thyroid traits. Some of these pathways, including allograft rejection, autoimmune diseases (IBD, SLE, T1D, AITD, RA), C3/C4/C5 complement system and COVID-19, overlapped across headache and thyroid traits. Due to the polygenic contribution of TSH and fT4-associated variants and their shared physiology with headache, we observed some overlapping genes between the traits which relate to some autoimmune diseases. The complement system is made up of a network of plasma and membrane proteins that interacts with the innate and adaptive immune systems. Dysregulated or impaired complement system activation is known to contribute to neurodegenerative diseases with headache as the most common symptom [56]. Similarly, C3/C4 complement system is reported to be overactivated in hypothyroidism patients [57]. A recent review characterising headaches in COVID-19 cases reported the high rate of males suffering from long-lasting headaches as compared to females who have a higher prevalence of headaches in the general population [58]. On the other hand, it has been suggested that COVID-19 triggers the activation of pre-existing thyroid disease/autoimmunity [59] and is causally associated with a higher risk of hypothyroidism [60], whereas a recent study has also suggested that the presence of thyroid abnormalities increases the risk of COVID-19 [61].

Our causal analysis found a significant positive causal relationship between hypothyroidism and headache, and a significant negative causal relationship between hyperthyroidism and headache. We also found a significant positive causal relationship between headache and secondary hypothyroidism. These causal relationships suggest that their association might be due to both shared molecular genetic mechanisms as well as causality. Although the underlying mechanism(s) is unclear, the role of the hypothalamus in the comorbid state of headache and thyroid dysfunction is a possible hypothesis. A recent study has reported that cases with headache/migraine conditions are represented by neuro-endocrinological changes (regulated by the hypothalamus and limbic system) and thus leads to change in the hormone status of hypothalamically regulated hormones such as TSH, growth hormones, and testosterone. The pain during headache might contribute to the increase or decrease of TSH and fT4 levels (which are based on the individual set-point of the hypothalamus-pituitary-thyroid (HPT) axis) leading to thyroid dysfunction in cases with headache [62]. This hypothesis could explain the mechanism underlying the observed causal relationship between headache and secondary hypothyroidism. Thus, understanding the cause of secondary hypothyroidism is important to assess the headache. The pathophysiological mechanism relating to thyroid dysfunction causing headache is not known. A few hypotheses which could suggest the possible role of thyroid hormone to increase oxidative stress in the brain, cause vasospasm and vasoconstriction and thus causing headache have been studied [63]. This could possibly explain the causal relationship observed between hypothyroidism and hyperthyroidism with headache in the present study.

Our genetic results suggests that in euthyroidism it is important to maintain the levels of TSH and fT4 within the reference range and even a slight change in the levels of fT4 could be associated with headache, however other mechanisms are involved when comparing cases of primary or secondary hypothyroidism (very low levels of fT4) and cases of hyperthyroidism (very high levels of fT4). TSH levels are very sensitive to minor changes in the fT4 levels, thus in thyroid dysfunction abnormal TSH levels are detected earlier than abnormal fT4 levels and the results from our genetic study indicate that fT4 levels have a significant relationship with headache. Additionally, because of the complex relationship observed with secondary hypothyroidism, fT4 levels in blood should be measured in headache patients to understand if thyroid dysfunction causes headache. Thus, testing of blood TSH and fT4 levels should be aided, particularly when symptoms of thyroid dysfunction, e.g., fatigue, constipation, weight gain, muscle weakness, joint pain, slow heart rate depression for hypothyroidism; and/or anxiety, lack of sleep, diarrhoea, tiredness for hyperthyroidism co-occur with headache in a patient [15]. This is not currently recommended by the national or international guidelines to test for headache and thyroid traits comorbidity.

The present study has three possible limitations. First, since we utilised GWAS summary statistics from European ancestry our conclusions may not generalise to other ancestries. Second, our conclusions are limited to the general susceptibility of headache and thyroid traits as the sex-specific GWAS datasets were not available. Third, although there is a slight sample overlap between the headache and PANUK thyroid traits GWAS datasets, we do not expect it to affect our conclusions, indeed the LDSC genetic correlation analyses indicated a very small sample overlap with genecov intercepts very close to zero.

## 5. Conclusions

The present study provides insight into the genetic and causal relationships between headache and thyroid traits which will help to understand the disease co-occurrence and their management. Our results show a significant genetic correlation between headache and hypothyroidism, hyperthyroidism, and fT4. The shared loci, genes and pathways identified between headache and thyroid traits provide biological insight into their comorbid relationship. Additionally, the causal relationship observed between headache and secondary hypothyroidism; and the genomic regions shared between headache, hypothyroidism, hyperthyroidism, secondary hypothyroidism, and fT4 indicate the importance of measuring levels of both TSH and fT4 when assessing patients with headaches.

## Figures and Tables

**Table 1:** Genetic correlation between headache and thyroid traits.

Trait1 (t1)	Cases/Controls	*h* ^2^	Trait2 (t2)	Cases/Controls	*h* ^2^	Valid SNPs	*r*_g_	SE	Z	*p*	Intercept t1(SE)/t2(SE)	Intercept (Genecov)
Headache	84,036/335,552	0.10	Hypothyroidism	20,563/399,910	0.22	1181077	0.09	0.02	3.79	**2.00 × 10^−4^**	1.03(0.01)/1.03(0.02)	0.0160
Headache	84,036/335,552	0.10	Hyperthyroidism	3197/417,276	0.15	1181077	−0.14	0.04	−3.12	**1.80 × 10^−3^**	1.03(0.01)/1.01(0.01)	−0.0038
Headache	84,036/335,552	0.10	Secondary Hypothyroidism	1430/399,034	0.04	1181077	0.20	0.10	1.94	5.24 × 10^−2^	1.03(0.01)/1.01(0.01)	0.0061
Headache	84,036/335,552	0.10	TSH	72,167	0.11	1164428	−0.04	0.03	−1.13	0.2597	1.03(0.01)/1.04(0.01)	Constrained to 0
Headache	84,036/335,552	0.10	fT4	72,167	0.15	1164126	0.08	0.03	2.78	**5.50 × 10^−3^**	1.03(0.01)/1.01(0.01)	Constrained to 0

*h*^2^, heritability of the traits; valid SNPs, number of SNPs used in estimating genetic correlation; *r*_g_, genetic correlation; SE, standard error; Z, Z-score; *p*, *p*-value; Intercept t1(SE)/t2(SE), LD score regression intercept for trait 1 (SE) and trait 2 (SE); Intercept (genecov), LD score regression intercept on genetic covariance scale. Significant genetic correlations (*p* < 0.05) are shown in bold.

**Table 2:** Genome-wide significant SNPs from cross-trait meta-analysis between headache and thyroid traits.

Trait 1	Trait 2	SNP	EA	NEA	chr	pos	Meta-Analysis			Trait 1			Trait 2			Nearest Gene
							*p*	*β*	SE	*p*	*β*	SE	*p*	*β*	SE	
Headache	HPO	rs187359614	A	G	4	29763484	2.12 × 10^−8^	0.36	0.064	6.63 × 10^−7^	0.36	0.073	9.78 × 10^−3^	0.35	0.134	*AC109351.1*
		rs3960788	C	T	4	103915618	3.53 × 10^−8^	−0.03	0.005	4.53 × 10^−7^	−0.03	0.006	2.42 × 10^−2^	−0.03	0.011	*SLC9B1*
		rs215695	T	C	7	32397908	1.29 × 10^−9^	−0.03	0.006	1.16 × 10^−7^	−0.03	0.006	3.10 × 10^−3^	−0.04	0.012	*PDE1C*
		rs7485814	T	A	12	84326974	2.96 × 10^−8^	0.03	0.005	4.12 × 10^−7^	0.03	0.006	2.23 × 10^−2^	0.03	0.011	*SNORA3*
		rs12912956	C	T	15	72568275	4.94 × 10^−8^	0.03	0.006	3.76 × 10^−6^	0.03	0.007	3.55 × 10^−3^	0.04	0.012	*CELF6*
	SHPO	rs59382356	T	C	2	100484479	3.50 × 10^−8^	−0.03	0.006	1.33 × 10^−7^	−0.03	0.006	4.70 × 10^−2^	−0.08	0.039	*Aff3*
		rs42854	C	G	5	74963277	4.35 × 10^−8^	−0.03	0.006	2.33 × 10^−7^	−0.03	0.006	1.62 × 10^−2^	−0.10	0.041	*ANKDD1B*
		rs28540738	G	A	14	93591673	2.57 × 10^−8^	−0.03	0.006	1.14 × 10^−7^	−0.03	0.006	3.15 × 10^−2^	−0.09	0.041	*ITPK1*
	TSH	rs12723104	C	T	1	33760197	1.42 × 10^−8^	−0.03	0.005	4.57 × 10^−3^	−0.02	0.007	1.34 × 10^−7^	−0.04	0.007	*ZNF362*
		rs6672112	C	T	1	51188594	1.73 × 10^−9^	0.03	0.005	6.44 × 10^−4^	0.02	0.006	2.58 × 10^−7^	0.03	0.007	*FAF1*
		rs7590268	G	T	2	43540125	3.33 × 10^−9^	−0.03	0.005	1.94 × 10^−3^	−0.02	0.007	8.10 × 10^−8^	−0.04	0.007	*THADA*
		rs12549150	T	C	8	11422936	1.17 × 10^−8^	−0.02	0.004	1.17 × 10^−7^	−0.03	0.006	7.90 × 10^−3^	−0.02	0.006	*BLK*
		rs71460549	C	G	11	57484130	9.68 × 10^−9^	0.03	0.005	2.47 × 10^−7^	0.03	0.006	4.33 × 10^−3^	0.02	0.007	*TMX2:TMX2-CTNND1*
		rs16958839	T	A	16	82934879	2.94 × 10^−8^	−0.06	0.011	1.56 × 10^−5^	−0.06	0.014	5.11 × 10^−4^	−0.06	0.018	*CDH13*
		rs4793187	A	G	17	41121263	8.30 × 10^−9^	−0.03	0.005	5.64 × 10^−7^	−0.03	0.006	1.91 × 10^−3^	−0.02	0.007	*PTGES3L-AARSD1:PTGES3L*
		rs4986170	A	G	17	43207858	5.20 × 10^−10^	−0.03	0.006	6.94 × 10^−6^	−0.03	0.008	1.79 × 10^−5^	−0.04	0.008	*PLCD3*
		rs6504608	A	C	17	47424681	1.31 × 10^−8^	0.02	0.004	7.19 × 10^−3^	0.02	0.006	7.16 × 10^−8^	0.03	0.006	*ZNF652*
		rs116855522	T	C	19	5293048	1.03 × 10^−8^	−0.03	0.005	2.47 × 10^−4^	−0.02	0.006	5.61 × 10^−6^	−0.03	0.007	*PTPRS*
		rs8108474	T	C	19	46301479	9.65 × 10^−9^	−0.03	0.004	5.62 × 10^−6^	−0.03	0.006	3.90 × 10^−4^	−0.02	0.006	*RSPH6A*
		rs6141766	G	A	20	31222769	1.55 × 10^−9^	0.04	0.006	9.22 × 10^−7^	0.04	0.008	3.38 × 10^−4^	0.03	0.009	*C20orf203*
	fT4	rs2356864	A	G	1	50839740	6.93 × 10^−9^	−0.03	0.004	2.15 × 10^−7^	−0.03	0.006	3.82 × 10^−3^	−0.02	0.007	*HMGB1P45*
		rs6668959	T	C	1	174057626	6.04 × 10^−10^	−0.03	0.004	6.09 × 10^−6^	−0.03	0.006	2.37 × 10^−5^	−0.03	0.007	*RPL30P1*
		rs143000423	A	C	6	36591128	1.30 × 10^−8^	0.09	0.016	6.15 × 10^−6^	0.09	0.020	5.64 × 10^−4^	0.09	0.027	*MIR3925*
		rs17055186	G	A	8	26260910	4.70 × 10^−8^	0.04	0.007	1.13 × 10^−4^	0.03	0.009	1.03 × 10^−4^	0.04	0.010	*BNIP3L*
		rs12343333	C	T	9	109620811	5.90 × 10^−10^	0.03	0.005	2.12 × 10^−5^	0.03	0.006	5.58 × 10^−6^	0.03	0.007	*ZNF462*
		rs2926664	G	A	11	57400495	1.32 × 10^−8^	−0.03	0.004	3.49 × 10^−3^	−0.02	0.006	1.20 × 10^−7^	−0.04	0.007	*AP000662.4*
		rs580223	A	T	11	57541120	2.00 × 10^−9^	−0.03	0.005	8.89 × 10^−6^	−0.03	0.007	5.42 × 10^−5^	−0.03	0.008	*TMX2-CTNND1:RP11-691N7.6:CTNND1*
		rs12883201	T	C	14	93570702	4.55 × 10^−10^	−0.03	0.005	7.92 × 10^−5^	−0.03	0.007	6.98 × 10^−7^	−0.04	0.008	*ITPK1*
		rs8029914	T	C	15	49304651	2.99 × 10^−8^	0.02	0.004	5.90 × 10^−5^	0.02	0.006	1.33 × 10^−4^	0.03	0.007	*SECISBP2L*

SNP, single nucleotide polymorphism; EA, effect allele; NEA, non-effect allele; chr, chromosome number; pos, position of SNP; *p*, *p*-value; *β*, effect size of EA; SE, standard error of *β*; HPO, hypothyroidism; HPER, hyperthyroidism, SHPO, secondary hypothyroidism; TSH, thyroid stimulating hormone; fT4, free thyroxine.

**Table 3:** MR results for headache and thyroid traits.

**(a) 2SMR**														
**Exposure**	**Outcome**	**IVW**		**Weighted median**		**MR-Egger**		**MR-Egger** **Intercept**		**MR-PRESSO**				
		***β***	***p***	***β***	***p***	***β***	***p***	**Intercept**	***p***	**Global test *p***	**Raw *β***	**Raw *p***	**Corr-*β***	**Corr- *p***
HPO	Headache	−0.01	0.12	−0.02	**1.99 × 10^−2^**	−0.01	0.53	−0.001	0.93	< 1 × 10^−4^	−0.01	0.22	−0.01	0.21
Headache	HPO	0.04	0.69	−0.03	0.65	−0.30	0.24	0.019	0.16	< 1 × 10^−4^	0.04	0.66	0.04	0.49
HPER	Headache	−0.04	**4.76 × 10^−2^**	−0.03	**8.71 × 10^−3^**	−0.09	**4.13 × 10^−2^**	0.015	0.17	< 1 × 10^−4^	−0.04	0.06	−0.01	0.35
Headache	HPER	−0.17	0.5	0.11	0.43	−0.37	0.6	0.011	0.76	< 1 × 10^−4^	−0.15	0.53	0.17	0.09
SHPO	Headache	0.02	0.14	0.03	0.13	0.001	0.97	0.007	0.53	0.3581	−0.0001	0.97	-	-
Headache	SHPO	0.37	**4.19 × 10^−3^**	0.47	**9.81 × 10^−3^**	0.46	0.21	−0.005	0.8	0.2104	0.4	**1.60 × 10^−3^**	-	-
TSH	Headache	0.004	0.83	0.02	0.38	0.03	0.46	−0.002	0.47	0.0499	0.02	0.18	0.03	**3.88 × 10^−2^**
Headache	TSH	0.008	0.83	−0.04	0.24	−0.11	0.29	0.007	0.22	< 1 × 10^−4^	0.02	0.56	0.04	0.09
fT4	Headache	0.012	0.49	0.07	0.22	0.08	0.66	−0.001	0.8	0.0027	0.01	0.69	−0.01	0.63
Headache	fT4	0.04	0.28	0.04	0.37	−0.02	0.81	0.004	0.51	0.0014	−0.01	0.76	−0.02	0.48
**(b) GSMR**														
**Exposure**			**Outcome**			***β***			**SE**			***p***		
HPO			Headache			0.01			0.004			**2.45 × 10^−3^**		
Headache			HPO			−0.05			0.044			0.28		
HPER			Headache			−0.04			0.005			**1.16 × 10^−13^**		
Headache			HPER			0.19			0.1			**4.89 × 10^−2^**		
SHPO			Headache			−0.03			0.097			0.76		
Headache			SHPO			0.5			0.14			**3.64 × 10^−4^**		
TSH			Headache			0.001			0.014			0.94		
Headache			TSH			−0.01			0.0235			0.64		
fT4			Headache			−0.004			0.0199			0.82		
Headache			fT4			0.04			0.0255			0.16		
**(c) LCV**														
**Trait 1**			**Trait 2**			**gcp**			**SE**			***p***		
Headache			HPO			0.01			0.58			0.99		
			HPER			0.17			0.48			0.89		
			SHPO			−0.50			0.08			0.56		
			TSH			−0.02			0.57			0.89		
			fT4			−0.14			0.25			0.64		

SNPs, single nucleotide polymorphisms utilised as instrumental variables; IVW, inverse variance weighted; MR, Mendelian randomisation; MR-PRESSO, Mendelian Randomisation Pleiotropy RESidual Sum and Outlier; *β*, effect size; *p*, *p*-value; Corr-*β*, corrected *β*; Corr-*p*, corrected *p*-value; SE, standard error; gcp; genetic causality proportion; HPO, hypothyroidism; HPER, hyperthyroidism, SHPO, secondary hypothyroidism; TSH, thyroid stimulating hormone; fT4, free thyroxine. Significant causal relationships (*p* < 0.05) are shown in bold.

**Table 4:** Independent gene-based association analysis and gene-based genetic overlap between headache and hypothyroidism.

**(a) Effective number of independent genes in headache and hypothyroidism**											
**Disorder**	**Total genes**		***p* < 0.1**			***p* < 0.05**			***p* < 0.01**		
	**Raw ^3^**	**Effective ^4^**	**Raw ^3^**	**Effective ^4^**	**Proportion ^5^**	**Raw ^3^**	**Effective ^4^**	**Proportion ^5^**	**Raw ^3^**	**Effective ^4^**	**Proportion ^5^**
Headache ^1^	33,264	22,365	8107	5381	0.241	5378	3555	0.159	2394	1536	0.069
Hypothyroidism ^2^	33,264	22,567	8677	5832	0.258	6148	4133	0.183	3218	2123	0.094
**(b) Number of overlapping genes and binomial test *p*-value for gene-level genetic overlap**											
**Discovery set**		**Target set**		**Overlapping genes**		**Proportion of overlap**				**Binomial test *p*-value**	
				**Raw**	**Effective**	**Expected**		**Observed**			
***p* < 0.1**											
Headache		Hypothyroidism		2790	1819	5832/22,567= 0.258		1819/5381 = 0.338		4.72 × 10^−39^	
***p* < 0.05**											
Headache		Hypothyroidism		1638	1034	4133/22,567= 0.183		1034/3555 = 0.291		2.83 × 10^−55^	
***p* < 0.01**											
Headache		Hypothyroidism		709	392	2123/22,567= 0.094		392/1536 = 0.255		6.83 × 10^−75^	

^1^ Headache dataset obtained from PANUK Biobank Neale lab, ^2^ Hypothyroidism dataset obtained from PANUK Biobank Neale lab, ^3^ Raw number of genes (total number of genes obtained in the gene-based association analysis using GATES software), ^4^ Effective number of independent genes (the total number of independent genes obtained in the independent gene-based test using the ‘genetic type 1 error calculator’ method), ^5^ Proportion of the total effective number of independent genes.

## Data Availability

The present study was based on a secondary analysis of GWAS summary statistics, and all data generated are included in this published article (and its Supplementary Tables). The GWAS datasets analysed for all traits are available online through the links and references provided in the subsection describing summary statistics of GWAS datasets.

## Supplementary Materials

The following supporting information can be downloaded at: https://www.mdpi.com/article/10.3390/genes14010016/s1, Table S1: Estimated univariate heritability for headache and thyroid traits using LDSC; Table S2: Pleiotropic loci with top significant SNPs influencing headache and hypothyroidism identified by GWAS-PW; Table S3: Pleiotropic loci with top significant SNPs influencing headache and hyperthyroidism identified by GWAS-PW; Table S4: Pleiotropic loci with top significant SNPs influencing headache and secondary hypothyroidism identified by GWAS-PW; Table S5: Pleiotropic loci with top significant SNPs influencing headache and fT4 identified by GWAS-PW; Table S6: Independent gene-based association analysis and gene-based genetic overlap between headache and hyperthyroidism; Table S7: Independent gene-based association analysis and gene-based genetic overlap between headache and secondary hypothyroidism; Table S8: Independent gene-based association analysis and gene-based genetic overlap between headache and TSH; Table S9: Independent gene-based association analysis and gene-based genetic overlap between headache and fT4; Table S10: Genes overlapping headache and hypothyroidism at *p* < 0.05 and Fisher combined *p*-value (*p*_FCP_ < 2.09 × 10^−6^); Table S11: Genes overlapping headache and hyperthyroidism at *p* < 0.05 and Fisher combined *p*-value (*p*_FCP_ < 2.09 × 10^−6^); Table S12: Genes overlapping headache and secondary hypothyroidism at *p* < 0.05 and Fisher combined *p*-value (*p*_FCP_ < 2.09 × 10^−6^); Table S13: Genes overlapping headache and TSH at *p* < 0.05 and Fisher combined *p*-value (*p*_FCP_ < 2.09 × 10^−6^); Table S14: Genes overlapping headache and fT4 at *p* < 0.05 and Fisher combined *p*-value (*p*_FCP_ < 2.09 × 10^−6^); Table S15: Pathways associated with headache and hypothyroidism; Table S16: Pathways associated with headache and hyperthyroidism; Table S17: Pathways associated with headache and secondary hypothyroidism; Table S18: Pathways associated with headache and TSH; Table S19: Pathways associated with headache and fT4.
