# Supplementary material for: Cross-Trait Genetic Analyses Indicate Pleiotropy and Complex Causal Relationships between Headache and Thyroid Function Traits
Source: Genes (Basel). 2022 Dec 21;14(1):16. doi: 10.3390/genes14010016 (PMC9858525; doi:10.3390/genes14010016)
Supplement: Supplementary file 1 [file genes-14-00016-s001.zip › genes-2083022-supplementary.pdf]

Supplementary Table S1. Estimated univariate heritability for headache and thyroid traits using LDSC.

**Trait**	**Sample prevalence (%)**	**Population prevalence (%)**	**Cases/Controls**	**Valid SNPs**	**h^2 (SE)**	**95% CI**	**Intercept (SE)**	**Z score**	***p***
Headache (PANUKBB)	20	50	84,036/ 335,552	1181077	0.0993 (0.0057)	(0.09-0.11)	1.0334 (0.0084)	17.42	5.71E-68
Hypothyroidism (PANUKBB)	4.9	5.3	20,563/399,910	1181077	0.2037 (0.0183)	(0.17-0.24)	1.0513 (0.0129)	11.13	8.96E-29
Hyperthyroidism (PANUKBB)	0.76	1.3	3,197/417,276	1181077	0.141 (0.0275)	(0.09-0.19)	1.0031 (0.0068)	5.13	2.90E-07
Secondary Hypothyroidism (PANUKBB)	0.36	0.0013	1,430/399,034	1181077	0.0407 (0.0188)	(0.004-0.08)	1.0004 (0.0068)	2.16	3.08E-02
TSH (Teumer et al.)	NA	NA	72,167	1166342	0.1253 (0.0192)	(0.09-0.16)	1.0352 (0.0098)	6.53	6.58E-11
fT4 (Teumer et al.)	NA	NA	72,167	1166086	0.1514 (0.0162)	(0.12-0.18)	1.0137 (0.0078)	9.35	8.76E-21

Valid SNPs, number of SNPs used for estimating univariate heritability; h^2: univariate heritability; CI, Confidence Interval; SE, standard error; Z score, z_score of univariate h^2 (h^2/h^2_se); *p*, calculated using R software.

Supplementary Table S2. Pleiotropic loci with top significant SNPs influencing headache and hypothyroidism identified by GWAS-PW.

chunk	NSNP	chr	st	sp	PPA_1	PPA_2	PPA_3	PPA_4	Headache					Hypothyroidism			LD
									SNP	EA	NEA	Beta	*p*	SNP	Beta	*p*	
1244	8366	12	109025901	110336618	3.03×10^{-7}	9.58×10^{-8}	0.9747	0.0253	rs116940627	T	C	–0.07	1.09×10^{-7}	rs116940627	–0.12	3.77×10^{-6}	1
649	8160	6	24852296	25684555	9.22×10^{-11}	9.60×10^{-9}	0.9594	0.0406	rs9295661	C	A	0.05	1.09×10^{-7}	rs9295661	0.12	9.53×10^{-11}	1
324	16101	3	70449028	72529129	9.53×10^{-6}	3.15×10^{-8}	0.8513	0.1487	rs115358873	A	G	0.68	7.96×10^{-3}	rs115358873	0.88	5.87×10^{-2}	1
1124	12278	11	49866050	54694395	1.26×10^{-5}	2.49×10^{-6}	0.8420	0.1579	rs141313786	C	G	–0.09	1.70×10^{-5}	rs141313786	0.15	6.63×10^{-5}	1
1125	2969	11	54696361	55082567	1.38×10^{-5}	3.72×10^{-6}	0.7980	0.2019	rs72920604	T	C	–0.08	2.12×10^{-5}	rs72920604	0.13	2.90×10^{-4}	1
1562	10525	18	57630824	59020745	1.32×10^{-5}	1.66×10^{-5}	0.5792	0.4205	rs768293565	T	G	–0.31	1.54×10^{-2}	rs768293565	–0.58	1.51×10^{-2}	1

chunk, ID representing the LD region; NSNP, number of SNP in the LD region; chr, chromosome; st, start position of the LD region; sp, stop position of the LD region; PPA_1, estimated posterior probability of model 1 (locus affecting only headache); PPA_2, estimated posterior probability of model 2 (locus affecting only hypothyroidism); PPA_3, estimated posterior probability of model 3 (locus affecting both headache and hypothyroidism via a single SNP); PPA_4, estimated posterior probability of model 4 (locus affecting both headache and hypothyroidism via two different SNPs); SNP, single nucleotide polymorphism; EA, effect allele; NEA, non-effect allele; Beta, effect of association; *p*, p-value; LD, linkage disequilibrium.

Supplementary Table S3. Pleiotropic loci with top significant SNPs influencing headache and hyperthyroidism identified by GWAS-PW.

chunk	NSNP	chr	st	sp	PPA_1	PPA_2	PPA_3	PPA_4	Headache					Hyperthyroidism			LD
									SNP	EA	NEA	Beta	*p*	SNP	Beta	*p*	
649	8160	6	24852296	25684555	3.52×10^{-6}	9.07×10^{-9}	0.9938	0.0062	rs9295661	C	A	0.05	1.09×10^{-7}	rs9295661	0.28	7.38×10^{-10}	1
657	8771	6	32682664	33236268	7.68×10^{-75}	1.37×10^{-7}	0.9833	0.0167	rs3957147	T	C	–0.03	7.40×10^{-6}	rs3957147	–0.69	1.71×10^{-83}	1
652	7238	6	28018246	28917525	6.99×10^{-28}	9.27×10^{-11}	0.9732	0.0268	rs35949109	C	T	0.04	1.04×10^{-7}	rs35949109	–0.35	5.93×10^{-21}	1
129	16175	1	242071724	244108295	1.82×10^{-4}	4.23×10^{-8}	0.9663	0.0335	rs12067370	C	T	0.03	1.90×10^{-6}	rs12067370	0.15	1.82×10^{-8}	1
655	15927	6	30798168	31571130	1.56×10^{-70}	9.73×10^{-10}	0.9590	0.0410	rs9263610	C	T	–0.05	1.02×10^{-8}	rs9263610	–0.66	5.21×10^{-66}	1
650	8795	6	25684587	26789690	3.73×10^{-16}	1.18×10^{-11}	0.9251	0.0749	rs34493019	G	A	–0.06	3.57×10^{-9}	rs34493019	–0.30	3.44×10^{-12}	1
1369	8893	14	93132477	94325277	0.0306	2.90×10^{-6}	0.8203	0.1490	rs80307714	A	G	0.88	9.42×10^{-3}	rs80307714	–3.08	4.39×10^{-2}	1
1520	11698	17	63148928	64799756	0.0519	7.03×10^{-5}	0.7976	0.1486	rs185476835	A	G	–0.11	4.14×10^{-2}	rs185476835	–0.54	2.16×10^{-2}	1
651	9167	6	26791421	28017544	1.96×10^{-20}	1.43×10^{-10}	0.7890	0.2110	rs374593247	T	G	–0.06	1.91×10^{-11}	rs374593247	–0.36	9.68×10^{-18}	1
5	16356	1	7247335	9365093	0.0805	7.74×10^{-5}	0.7783	0.1377	rs1775049	C	T	0.01	5.34×10^{-2}	rs1775049	0.06	2.25×10^{-2}	1
1097	9993	11	3665604	4746391	0.1346	7.66×10^{-5}	0.7269	0.1328	rs139586069	A	C	–0.09	5.00×10^{-3}	rs139586069	–0.34	1.57×10^{-2}	1
656	19952	6	31571330	32682590	7.56×10^{-94}	4.18×10^{-8}	0.7228	0.2772	rs3130631	G	C	–0.05	1.45×10^{-8}	rs3130631	–0.75	5.96×10^{-80}	1
1279	5878	13	46495674	47430788	0.1489	3.35×10^{-7}	0.6453	0.2058	rs2794661	C	T	–0.03	4.64×10^{-4}	rs2794661	0.07	3.21×10^{-2}	1
278	16565	3	1441779	2991923	0.2186	1.03×10^{-4}	0.5559	0.2177	rs189475718	C	T	–0.17	3.51×10^{-2}	rs189475718	–0.85	1.85×10^{-2}	1

chunk, ID representing the LD region; NSNP, number of SNP in the LD region; chr, chromosome; st, start position of the LD region; sp, stop position of the LD region; PPA_1, estimated posterior probability of model 1 (locus affecting only headache); PPA_2, estimated posterior probability of model 2 (locus affecting only hyperthyroidism); PPA_3, estimated posterior probability of model 3 (locus affecting both headache and hyperthyroidism via a single SNP); PPA_4, estimated posterior probability of model 4 (locus affecting both headache and hyperthyroidism via two different SNPs); SNP, single nucleotide polymorphism; EA, effect allele; NEA, non-effect allele; Beta, effect of association; *p*, p-value; LD, linkage disequilibrium.

Supplementary Table S4. Pleiotropic loci with top significant SNPs influencing headache and secondary hypothyroidism identified by GWAS-PW.

chunk	NSNP	chr	st	sp	PPA_1	PPA_2	PPA_3	PPA_4	Headache					Secondary Hypothyroidism			LD
									SNP	EA	NEA	Beta	*p*	SNP	Beta	*p*	
656	19952	6	31571330	32682590	9.68×10^{-5}	4.63×10^{-9}	0.9932	0.0067	rs3130631	G	C	–0.05	1.45×10^{-8}	rs3130631	0.31	2.62×10^{-8}	1
657	8771	6	32682664	33236268	0.0096	6.39×10^{-7}	0.9731	0.0173	rs3957147	T	C	–0.03	7.41×10^{-6}	rs3957147	0.18	1.75×10^{-4}	1
655	15927	6	30798168	31571130	0.0034	4.74×10^{-9}	0.9525	0.0440	rs9263610	C	T	–0.05	1.02×10^{-8}	rs9263610	0.26	1.26×10^{-6}	1
203	12688	2	123302906	124732010	0.1463	8.64×10^{-5}	0.7901	0.0621	rs561716457	T	C	–0.51	4.14×10^{-2}	rs561716457	4.22	2.37×10^{-2}	1

chunk, ID representing the LD region; NSNP, number of SNP in the LD region; chr, chromosome; st, start position of the LD region; sp, stop position of the LD region; PPA_1, estimated posterior probability of model 1 (locus affecting only headache); PPA_2, estimated posterior probability of model 2 (locus affecting only secondary hypothyroidism); PPA_3, estimated posterior probability of model 3 (locus affecting both headache and secondary hypothyroidism via a single SNP); PPA_4, estimated posterior probability of model 4 (locus affecting both headache and secondary hypothyroidism via two different SNPs); SNP, single nucleotide polymorphism; EA, effect allele; NEA, non-effect allele; Beta, effect of association; *p*, p-value; LD, linkage disequilibrium.

Supplementary Table S5. Pleiotropic loci with top significant SNPs influencing headache and fT4 identified by GWAS-PW.

chunk	NSNP	chr	st	sp	PPA_1	PPA_2	PPA_3	PPA_4	Headache					fT4			LD
									SNP	EA	NEA	Beta	*p*	SNP	Beta	*p*	
508	6141	4	170776510	172558507	5.37×10^{-20}	4.68×10^{-6}	0.9825	0.0175	rs6854291	A	G	0.12	1.30×10^{-24}	rs6854291	0.05	2.19×10^{-6}	1
654	1974	6	31040488	31570931	0.0211	1.45×10^{-7}	0.9092	0.0697	rs9263610	C	T	–0.05	1.02×10^{-8}	rs9263610	–0.05	2.15×10^{-4}	1
105	3745	1	204681068	205921859	0.0032	0.0050	0.6289	0.3609	rs9438436	T	C	0.02	5.85×10^{-3}	rs9438436	0.02	1.24×10^{-3}	1
658	5908	6	35455756	37572375	0.2326	0.0010	0.5582	0.1123	rs143000423	A	C	0.09	1.30×10^{-8}	rs143000423	0.09	6.15×10^{-6}	1
988	4181	9	109298916	110694926	0.0620	0.0005	0.5567	0.3770	rs12343333	C	T	0.03	5.90×10^{-10}	rs12343333	0.03	2.12×10^{-5}	1

chunk, ID representing the LD region; NSNP, number of SNP in the LD region; chr, chromosome; st, start position of the LD region; sp, stop position of the LD region; fT4, free thyroxine; PPA_1, estimated posterior probability of model 1 (locus affecting only headache); PPA_2, estimated posterior probability of model 2 (locus affecting only fT4); PPA_3, estimated posterior probability of model 3 (locus affecting both headache and fT4 via a single SNP); PPA_4, estimated posterior probability of model 4 (locus affecting both headache and fT4 via two different SNPs); SNP, single nucleotide polymorphism; EA, effect allele; NEA, non-effect allele; Beta, effect of association; *p*, p-value; LD, linkage disequilibrium.

Supplementary Table S6. Independent gene-based association analysis and gene-based genetic overlap between headache and hyperthyroidism.

A) Effective number of independent genes in headache and hyperthyroidism

Disorder	Total genes		p < 0.1			p < 0.05			p < 0.01		
	Raw[3]	Effective[4]	Raw[3]	Effective[4]	Proportion[5]	Raw[3]	Effective[4]	Proportion[5]	Raw[3]	Effective[4]	Proportion[5]
Headache[1]	33,264	22,365	8,107	5,381	0.241	5,378	3,555	0.159	2,394	1,536	0.069
Hyperthyroidism[2]	33,264	22,094	4,786	3,018	0.137	2,708	1,710	0.077	984	577	0.026

B) Number of overlapping genes and binomial test p-value for gene-level genetic overlap

Discovery set	Target set	Overlapping genes		Proportion of overlap		Binomial test p-value
		Raw	Effective	Expected	Observed	
p < 0.1						
Headache	Hyperthyroidism	1,564	1,0.15	3,018/22,094= 0.137	1,076/7,084 = 0.152	4.35E-26
p < 0.05						
Headache	Hyperthyroidism	788	483	1,710/22,094= 0.077	483/3,555= 0.136	2.64E-33
p < 0.01						
Headache	Hyperthyroidism	413	196	577/22,094= 0.026	196/1,536= 0.128	1.86E-73

[1]Headache dataset obtained from PANUK Biobank Neale lab, [2]Hyperthyroidism dataset obtained from PANUK Biobank Neale lab, [3]Raw number of genes (total number of genes obtained in the gene-based association analysis using GATES software), [4]Effective number of independent genes (the total number of independent genes obtained in the independent gene-based test using the 'genetic type 1 error calculator' method), [5]Proportion of the total effective number of independent genes.

Supplementary Table S7. Independent gene-based association analysis and gene-based genetic overlap between headache and secondary hypothyroidism.

A) Effective number of independent genes in headache and secondary hypothyroidism

Disorder	Total genes		p < 0.1			p < 0.05			p < 0.01		
	Raw[3]	Effective[4]	Raw[3]	Effective[4]	Proportion[5]	Raw[3]	Effective[4]	Proportion[5]	Raw[3]	Effective[4]	Proportion[5]
Headache[1]	33,264	22,365	8,107	5,381	0.241	5,378	3,555	0.159	2,394	1,536	0.069
Secondary hypothyroidism[2]	33,264	21,965	3,821	2,489	0.113	2,074	1,375	0.063	581	368	0.017

B) Number of overlapping genes and binomial test p-value for gene-level genetic overlap

Discovery set	Target set	Overlapping genes		Proportion of overlap		Binomial test p-value
		Raw	Effective	Expected	Observed	
p < 0.1						
Headache	Secondary hypothyroidism	1,099	724	2,489/21,965= 0.113	724/5,381= 0.135	6.19E-07
p < 0.05						
Headache	Secondary hypothyroidism	411	281	1,375/21,965= 0.063	281/3,555= 0.079	8.05E-05
p < 0.01						
Headache	Secondary hypothyroidism	121	70	368/21,965= 0.017	70/1,536= 0.046	4.79E-13

[1]Headache dataset obtained from PANUK Biobank Neale lab, [2]Secondary hypothyroidism dataset obtained from PANUK Biobank Neale lab, [3]Raw number of genes (total number of genes obtained in the gene-based association analysis using GATES software), [4]Effective number of independent genes (the total number of independent genes obtained in the independent gene-based test using the 'genetic type 1 error calculator' method), [5]Proportion of the total effective number of independent genes.

Supplementary Table S8. Independent gene-based association analysis and gene-based genetic overlap between headache and TSH.

A) Effective number of independent genes in headache and TSH

Disorder	Total genes		p < 0.1			p < 0.05			p < 0.01		
	Raw[3]	Effective[4]	Raw[3]	Effective[4]	Proportion[5]	Raw[3]	Effective[4]	Proportion[5]	Raw[3]	Effective[4]	Proportion[5]
Headache[1]	32,029	23,957	7,475	5,267	0.22	5,242	3,625	0.151	2,447	1,604	0.067
TSH[2]	32,029	23,895	4,484	3,255	0.136	2,814	2,032	0.085	978	712	0.03

B) Number of overlapping genes and binomial test p-value for gene-level genetic overlap

Discovery set	Target set	Overlapping genes		Proportion of overlap		Binomial test p-value
		Raw	Effective	Expected	Observed	
p < 0.1						
Headache	TSH	1,250	915	3,255/23,895= 0.136	915/5,267= 0.174	7.09E-15
p < 0.05						
Headache	TSH	618	456	2,032/23,895= 0.085	456/3,625= 0.126	7.68E-01
p < 0.01						
Headache	TSH	186	120	712/23,895= 0.03	120/1,604= 0.075	4.42E-19

[1]Headache dataset obtained from PANUK Biobank Neale lab, [2]TSH dataset obtained from Teumer et al., [3]Raw number of genes (total number of genes obtained in the gene-based association analysis using GATES software), [4]Effective number of independent genes (the total number of independent genes obtained in the independent gene-based test using the 'genetic type 1 error calculator' method), [5]Proportion of the total effective number of independent genes.

Supplementary Table S9. Independent gene-based association analysis and gene-based genetic overlap between headache and fT4.

A) Effective number of independent genes in headache and fT4

Disorder	Total genes		p < 0.1			p < 0.05			p < 0.01		
	Raw[3]	Effective[4]	Raw[3]	Effective[4]	Proportion[5]	Raw[3]	Effective[4]	Proportion[5]	Raw[3]	Effective[4]	Proportion[5]
Headache[1]	31,933	23,919	7,427	5,239	0.219	5,180	3,595	0.150	2,421	1,593	0.067
fT4[2]	31,933	23,866	5,054	3,634	0.152	3,189	2,233	0.094	1,090	754	0.032

B) Number of overlapping genes and binomial test p-value for gene-level genetic overlap

Discovery set	Target set	Overlapping genes		Proportion of overlap		Binomial test p-value
		Raw	Effective	Expected	Observed	
p < 0.1						
Headache	fT4	1,399	991	3,634/23,866= 0.152	991/5,239= 0.190	2.06E-13
p < 0.05						
Headache	fT4	743	494	2,233/23,866= 0.094	494/3,595= 0.137	2.56E-17
p < 0.01						
Headache	fT4	246	146	754/23,866= 0.032	146/1,593= 0.092	9.11E-29

[1]Headache dataset obtained from PANUK Biobank Neale lab, [2]fT4 dataset obtained from Teumer et al., [3]Raw number of genes (total number of genes obtained in the gene-based association analysis using GATES software), [4]Effective number of independent genes (the total number of independent genes obtained in the independent gene-based test using the 'genetic type 1 error calculator' method), [5]Proportion of the total effective number of independent genes.

Supplementary Table S10. Genes overlapping headache and hypothyroidism at $p < 0.05$ and Fisher combined p-value ($p_{FCP} < 2.09 \times 10^{-6}$).

Chr	Gene.id	Name	Start	End	SNP.id	SNP.pos	pval(headache)	pval(hypoPAN)	X^2,4df	FCP
6	GeneID:3117	*HLA-DQA1*	32605183	32611429	rs9272729	32609594	4.50E-05	1.36E-163	770.0444066	2.3637E-165
6	GeneID:3119	*HLA-DQB1*	32627657	32634466	rs1130389	32632627	1.09E-05	1.61E-144	685.0340201	6.0632E-147
6	GeneID:29113	*C6orf15*	31079000	31080332	rs2233980	31079644	9.56E-07	1.12E-142	681.4349883	3.647E-146
6	GeneID:1041	*CDSN*	31082865	31088252	rs2233980	31079644	1.09E-06	1.28E-142	680.8952914	4.773E-146
6	GeneID:170679	*PSORS1C1*	31082608	31107869	rs2233980	31079644	1.44E-06	1.68E-142	679.801353	8.2345E-146
12	GeneID:10019	*SH2B3*	111843752	111889427	rs3184504	111884608	3.36E-03	2.50E-93	437.8410411	1.84626E-93
12	GeneID:6311	*ATXN2*	111890018	112037480	rs3184504	111884608	5.06E-03	4.42E-93	435.881299	4.89672E-93
12	GeneID:100101246	*LOC100101246*	112019098	112019749	rs653178	112007756	6.74E-03	4.58E-92	430.6346376	6.66757E-92
12	GeneID:642580	*LOC642580*	111819590	111823484	rs10849943	111819589	3.03E-03	5.39E-82	385.8537979	3.16629E-82
6	GeneID:3127	*HLA-DRB5*	32485151	32498006	rs115250958	32509842	3.77E-05	2.42E-78	377.8070808	1.73288E-80
6	GeneID:3128	*HLA-DRB6*	32520490	32527779	rs115250958	32509842	3.81E-05	2.75E-78	377.5319214	1.98702E-80
6	GeneID:170680	*PSORS1C2*	31105311	31107127	rs3130557	31094703	2.78E-06	9.99E-76	370.972594	5.18766E-79
6	GeneID:54535	*CCHCR1*	31110216	31126015	rs3132541	31098734	4.22E-06	9.07E-76	370.3337079	7.12786E-79
12	GeneID:8315	*BRAP*	112079950	112123790	rs601663	112123284	1.06E-03	5.56E-73	346.4537471	1.02244E-73
12	GeneID:217	*ALDH2*	112204691	112247789	rs737280	112194976	4.04E-04	5.08E-71	339.3463108	3.49986E-72
6	GeneID:3106	*HLA-B*	31321649	31324989	rs2523593	31326703	1.14E-06	2.99E-68	338.3279385	5.80616E-72
12	GeneID:80724	*ACAD10*	112123857	112194911	rs737280	112194976	5.48E-04	6.90E-71	338.1223397	6.4309E-72
6	GeneID:729816	*DHFRP2*	31331244	31334742	rs2523593	31326703	1.22E-06	3.18E-68	338.0754086	6.58268E-72
12	GeneID:51275	*C12orf47*	112277573	112280706	rs7137386	112283914	5.36E-04	4.58E-68	325.1718007	4.01408E-69
12	GeneID:8550	*MAPKAPK5*	112280279	112331228	rs7309970	112312368	8.49E-04	8.16E-68	323.0964509	1.12585E-68
12	GeneID:283450	*C12orf51*	112597992	112819896	rs7978523	112795744	1.38E-03	2.28E-66	315.4717536	4.9757E-67
12	GeneID:10906	*TRAFD1*	112563349	112591408	rs17630235	112591686	6.17E-03	1.23E-66	313.6976727	1.20132E-66
12	GeneID:6128	*RPL6*	112842994	112847443	rs3759377	112848570	4.27E-04	2.24E-65	313.2418582	1.50664E-66
12	GeneID:5781	*PTPN11*	112856536	112947717	rs3759377	112848570	1.05E-03	2.94E-65	310.9000957	4.82256E-66
12	GeneID:100500889	*MIR3657*	112475403	112475519	rs4767296	112463366	2.65E-04	1.33E-63	306.0300468	5.41979E-65
**Chr**	**Gene.id**	**Name**	**Start**	**End**	**SNP.id**	**SNP.pos**	**pval(headache)**	**pval(hypoPAN)**	**X^2,4df**	**FCP**

12	GeneID:80018	*NAA25*	112464493	112546635	rs4767296	112463366	6.72E-04	4.28E-63	301.8271485	4.37184E-64
6	GeneID:6890	*TAP1*	32812986	32821748	rs45506201	32808299	9.10E-06	1.48E-59	294.134729	1.99487E-62
6	GeneID:100507463	*LOC100507463*	32811863	32814277	rs45506201	32808299	9.66E-06	1.57E-59	293.8971336	2.2447E-62
6	GeneID:5696	*PSMB8*	32808494	32812712	rs45506201	32808299	1.03E-05	1.68E-59	293.6215515	2.57393E-62
6	GeneID:6891	*TAP2*	32789610	32806547	rs45506201	32808299	1.35E-05	2.20E-59	292.552821	4.37618E-62
6	GeneID:1589	*CYP21A2*	32006093	32009447	rs433061	32014828	1.77E-07	2.26E-55	282.7501709	5.68872E-60
6	GeneID:3112	*HLA-DOB*	32780540	32784825	rs241437	32797684	4.26E-03	3.18E-59	280.3084544	1.91199E-59
6	GeneID:7148	*TNXB*	32008932	32077151	rs1269852	32080191	3.04E-07	4.85E-55	280.1373599	2.0815E-59
6	GeneID:721	*C4B*	31982572	32003195	rs433061	32014828	1.16E-07	1.34E-49	257.0073044	2.01305E-54
6	GeneID:100287272	*USP8P1*	31243351	31246528	rs9264490	31232578	5.11E-05	1.68E-50	248.9848815	1.07705E-52
6	GeneID:3107	*HLA-C*	31236529	31239855	rs1793890	31222056	6.16E-05	2.45E-50	247.8576796	1.88387E-52
6	GeneID:5491	*PPIAP9*	31486754	31488108	rs3130614	31476458	2.59E-07	3.05E-47	244.5479389	9.7266E-52
6	GeneID:4277	*MICB*	31465855	31478901	rs3130614	31476458	4.11E-07	4.84E-47	242.6955967	2.43734E-51
6	GeneID:80736	*SLC44A4*	31830969	31846823	rs501942	31840477	4.53E-07	1.51E-45	235.6251009	8.11936E-50
6	GeneID:10919	*EHMT2*	31847536	31865464	rs501942	31840477	4.78E-07	1.59E-45	235.4148971	9.0112E-50
6	GeneID:5698	*PSMB9*	32821938	32827628	rs45506201	32808299	5.28E-06	1.58E-45	230.6224482	9.69602E-49
6	GeneID:4758	*NEU1*	31826829	31830709	rs501942	31840477	5.11E-07	1.32E-43	226.4342052	7.7298E-48
6	GeneID:100129921	*RPL15P4*	31495853	31496498	rs114751021	31504194	6.52E-03	2.07E-47	225.0577811	1.52908E-47
6	GeneID:401251	*C6orf26*	31730773	31732627	rs3130484	31715882	2.01E-07	5.56E-38	202.4067876	1.14127E-42
6	GeneID:1192	*CLIC1*	31698358	31704341	rs3130484	31715882	2.25E-07	6.21E-38	201.9623813	1.42215E-42
6	GeneID:80740	*LY6G6C*	31686425	31689510	rs3131383	31704294	1.48E-07	9.52E-38	201.948745	1.43179E-42
6	GeneID:100532732	*MSH5-C6orf26*	31707725	31732627	rs3130484	31715882	3.09E-07	8.55E-38	200.6825681	2.67991E-42
6	GeneID:80739	*C6orf25*	31691121	31694487	rs3101018	31705864	1.77E-07	1.54E-37	200.621343	2.76238E-42
6	GeneID:23564	*DDAH2*	31694817	31698039	rs3132445	31712196	2.03E-07	1.78E-37	200.0578684	3.65117E-42
6	GeneID:100302242	*MIR1236*	31924616	31924717	rs1270942	31918860	1.43E-07	6.38E-37	198.2060464	9.13169E-42
6	GeneID:7936	*RDBP*	31919864	31926864	rs1270942	31918860	1.48E-07	6.61E-37	198.0646404	9.79378E-42
6	GeneID:80737	*C6orf27*	31733371	31745108	rs3117573	31718396	2.15E-07	4.62E-37	198.0320305	9.95315E-42
6	GeneID:6499	*SKIV2L*	31926581	31937532	rs1270942	31918860	1.75E-07	7.81E-37	197.3940114	1.36495E-41
**Chr**	**Gene.id**	**Name**	**Start**	**End**	**SNP.id**	**SNP.pos**	**pval(headache)**	**pval(hypoPAN)**	**X^2,4df**	**FCP**

6	GeneID:629	*CFB*	31913721	31919861	rs1270942	31918860	1.76E-07	7.86E-37	197.3711651	1.38047E-41
6	GeneID:717	*C2*	31868776	31913449	rs1270942	31918860	2.79E-07	1.18E-36	195.6356941	3.259E-41
6	GeneID:1388	*ATF6B*	32083045	32096017	rs1269852	32080191	1.64E-07	3.69E-36	194.4199555	5.94838E-41
6	GeneID:4049	*LTA*	31539876	31542098	rs1800628	31546850	1.76E-07	9.69E-36	192.3524362	1.65485E-40
6	GeneID:259197	*NCR3*	31556660	31560762	rs1800628	31546850	1.79E-07	9.88E-36	192.2758059	1.71881E-40
6	GeneID:7124	*TNF*	31543350	31546112	rs1800628	31546850	1.86E-07	1.02E-35	192.1346136	1.8432E-40
6	GeneID:7940	*LST1*	31553956	31556686	rs1800628	31546850	1.90E-07	1.05E-35	192.039511	1.93201E-40
6	GeneID:4050	*LTB*	31548335	31550202	rs1800628	31546850	1.92E-07	1.06E-35	192.0069724	1.96337E-40
6	GeneID:100287329	*LOC100287329*	31527348	31540474	rs1800628	31546850	2.48E-07	1.02E-35	191.5665469	2.44148E-40
6	GeneID:692233	*SNORD117*	31504151	31504226	rs9267488	31514247	6.99E-07	6.73E-36	190.318507	4.52744E-40
6	GeneID:3305	*HSPA1L*	31777396	31782835	rs1043618	31783507	7.84E-04	6.41E-39	190.1877827	4.82996E-40
6	GeneID:3303	*HSPA1A*	31783291	31785719	rs1043618	31783507	8.06E-04	6.59E-39	190.0761108	5.10435E-40
6	GeneID:692199	*SNORD84*	31508878	31508955	rs9267488	31514247	8.34E-07	8.04E-36	189.6116556	6.42309E-40
6	GeneID:57819	*LSM2*	31765173	31774743	rs1043618	31783507	9.25E-04	7.57E-39	189.5239918	6.70782E-40
6	GeneID:534	*ATP6V1G2*	31512228	31514625	rs9267488	31514247	8.95E-07	8.63E-36	189.3287935	7.38797E-40
6	GeneID:100532737	*ATP6V1G2-DDX39B*	31497996	31514625	rs9267488	31514247	1.00E-06	9.65E-36	188.8815388	9.21783E-40
6	GeneID:4795	*NFKBIL1*	31514628	31526606	rs9267488	31514247	1.10E-06	1.06E-35	188.50989	1.10786E-39
6	GeneID:100131609	*HNRNPA1P2*	32293175	32294298	rs116667074	32285362	7.28E-06	2.83E-36	187.3662486	1.95078E-39
6	GeneID:10665	*C6orf10*	32260475	32339656	rs116667074	32285362	1.46E-05	5.69E-36	184.5717562	7.77264E-39
6	GeneID:7407	*VARS*	31745295	31763712	rs2075800	31777946	4.51E-02	6.53E-39	182.0443573	2.71308E-38
6	GeneID:116935	*RPL3P2*	31248068	31249348	rs114203148	31260364	3.97E-02	1.14E-32	153.5643523	3.50594E-32
6	GeneID:3304	*HSPA1B*	31795512	31798031	rs1043618	31783507	6.14E-04	3.89E-30	150.2313464	1.81615E-31
12	GeneID:144717	*FAM109A*	111798455	111806925	rs10849943	111819589	3.13E-03	7.93E-30	145.5468124	1.83152E-30
12	GeneID:10961	*ERP29*	112451152	112461024	rs4767296	112463366	3.44E-04	3.59E-28	142.3398006	8.90556E-30
12	GeneID:89894	*TMEM116*	112369086	112451023	rs4767296	112463366	6.69E-04	2.98E-28	141.3812057	1.42864E-29
12	GeneID:100420505	*LOC100420505*	112364998	112366738	rs11066085	112355949	3.53E-03	1.61E-28	139.2925284	4.00038E-29
6	GeneID:100507679	*MUC22*	30973729	31003179	rs141725002	30971976	3.36E-02	3.55E-27	128.5962206	7.77295E-27
6	GeneID:387122	*WASF5P*	31255162	31256941	rs114203148	31260364	2.36E-02	7.70E-26	123.1470539	1.13592E-25
**Chr**	**Gene.id**	**Name**	**Start**	**End**	**SNP.id**	**SNP.pos**	**pval(headache)**	**pval(hypoPAN)**	**X^2,4df**	**FCP**

6	GeneID:401250	*MCCD1*	31496739	31498008	rs114751021	31504194	6.50E-03	3.16E-25	122.8998219	1.28285E-25
6	GeneID:58496	*LY6G5B*	31638728	31640227	rs114733011	31631531	8.31E-03	1.25E-24	119.6544265	6.33096E-25
6	GeneID:1460	*CSNK2B*	31633657	31637843	rs114733011	31631531	9.37E-03	1.41E-24	119.1722547	8.02503E-25
6	GeneID:7918	*GPANK1*	31629006	31634060	rs114733011	31631531	1.06E-02	1.60E-24	118.6767976	1.02389E-24
6	GeneID:55937	*APOM*	31623671	31625987	rs114733011	31631531	1.18E-02	1.78E-24	118.2536015	1.26074E-24
6	GeneID:57827	*C6orf47*	31626075	31628549	rs114733011	31631531	1.18E-02	1.78E-24	118.2512115	1.26222E-24
6	GeneID:7917	*BAG6*	31606805	31620477	rs114733011	31631531	1.43E-02	2.16E-24	117.4737949	1.84983E-24
6	GeneID:720	*C4A*	31949834	31970457	rs6941112	31946614	2.32E-04	1.62E-22	117.0808126	2.24407E-24
6	GeneID:1797	*DOM3Z*	31937588	31940032	rs6941112	31946614	2.56E-04	1.79E-22	116.6853816	2.72558E-24
6	GeneID:8859	*STK19*	31938952	31949223	rs6941112	31946614	2.56E-04	1.79E-22	116.6853816	2.72558E-24
6	GeneID:3108	*HLA-DMA*	32916391	32920899	rs73396802	32915797	1.41E-03	1.13E-22	114.1978129	9.2561E-24
6	GeneID:6046	*BRD2*	32936437	32949282	rs57651384	32923849	1.52E-03	1.17E-22	113.980594	1.02987E-23
6	GeneID:100289545	*HIST1H3PS1*	26322104	26343616	rs9393703	26356951	7.53E-10	3.46E-15	108.6065521	1.44257E-22
12	GeneID:100128618	*LOC100128618*	112430798	112431862	rs2339907	112418146	2.47E-04	2.07E-20	107.2608748	2.79275E-22
12	GeneID:724085	*LOC724085*	112437040	112438873	rs7300252	112430346	4.49E-04	2.30E-20	105.8562766	5.56438E-22
12	GeneID:100302227	*MIR1302-1*	113132839	113132981	rs6489853	113129584	4.08E-02	2.69E-22	105.7340854	5.90824E-22
12	GeneID:654427	*PCNPP1*	112104737	112108183	rs10849966	112100773	6.46E-04	1.92E-20	105.485422	6.675E-22
6	GeneID:7738	*ZNF184*	27418521	27440897	rs35716472	27406607	9.90E-09	2.56E-15	104.060552	1.34296E-21
6	GeneID:10340	*HIST1H2BPS2*	27831840	27832179	rs45509595	27840926	8.08E-08	4.49E-16	103.3421039	1.91038E-21
6	GeneID:8332	*HIST1H2AL*	27833107	27833576	rs45509595	27840926	8.22E-08	4.56E-16	103.2758369	1.97349E-21
6	GeneID:3009	*HIST1H1B*	27834570	27835359	rs45509595	27840926	8.37E-08	4.64E-16	103.2047723	2.0435E-21
6	GeneID:8368	*HIST1H4L*	27840926	27841289	rs45509595	27840926	8.63E-08	4.79E-16	103.0820947	2.17023E-21
6	GeneID:8354	*HIST1H3I*	27839623	27840099	rs45509595	27840926	8.90E-08	4.94E-16	102.9576198	2.30686E-21
12	GeneID:100271245	*RPS2P41*	112317484	112318057	rs7309970	112312368	3.00E-04	1.76E-19	102.5851323	2.76925E-21
12	GeneID:160762	*CCDC63*	111284811	111345338	rs71458365	111274102	3.76E-05	1.76E-18	102.1380741	3.44809E-21
6	GeneID:100189185	*TRNAQ15*	26311975	26312046	rs13220522	26316295	1.10E-07	1.54E-15	100.2694426	8.61952E-21
6	GeneID:100189321	*TRNAQ25*	26311424	26311495	rs13220522	26316295	1.10E-07	1.54E-15	100.2692604	8.62029E-21
6	GeneID:100189436	*TRNAW9*	26319330	26319401	rs34107459	26328353	9.91E-08	1.79E-15	100.162534	9.0833E-21
**Chr**	**Gene.id**	**Name**	**Start**	**End**	**SNP.id**	**SNP.pos**	**pval(headache)**	**pval(hypoPAN)**	**X^2,4df**	**FCP**

6	GeneID:100189410	*TRNAR28*	26323046	26323118	rs34107459	26328353	1.01E-07	1.83E-15	100.0836446	9.44145E-21
6	GeneID:100189094	*TRNAM6*	26313352	26313423	rs34107459	26328353	1.01E-07	1.83E-15	100.0779994	9.46762E-21
6	GeneID:100189430	*TRNAS27*	26312824	26312905	rs13220522	26316295	1.20E-07	1.68E-15	99.90628511	1.0299E-20
6	GeneID:100189382	*TRNAS25*	26327817	26327898	rs9467714	26340785	9.25E-08	2.33E-15	99.77790291	1.0968E-20
6	GeneID:100189396	*TRNAR26*	26328368	26328440	rs9467714	26340785	9.83E-08	2.47E-15	99.53679184	1.23439E-20
12	GeneID:100421482	*LOC100421482*	112760277	112760900	rs10850031	112771063	8.46E-04	2.93E-19	99.50231748	1.25543E-20
6	GeneID:100189040	*TRNAM4*	26330529	26330600	rs9467714	26340785	9.87E-08	2.68E-15	99.36551414	1.3425E-20
6	GeneID:100189048	*TRNAW2*	26331672	26331743	rs9467714	26340785	1.03E-07	2.80E-15	99.18883222	1.46393E-20
6	GeneID:3122	*HLA-DRA*	32407619	32412823	rs116479312	32408500	2.78E-03	1.12E-19	99.03829447	1.57603E-20
6	GeneID:100189218	*TRNAS16*	26305718	26305801	rs13220522	26316295	1.53E-07	2.15E-15	98.93397401	1.6587E-20
6	GeneID:7232	*TRNAR3*	26299905	26299977	rs10484439	26309908	1.80E-07	2.14E-15	98.62018085	1.93444E-20
6	GeneID:645950	*HNRNPA1P1*	27491226	27492316	rs34573979	27480526	1.61E-08	2.28E-13	94.10282708	1.76822E-19
6	GeneID:100129192	*MICC*	30382490	30387543	rs149543464	30400763	2.63E-07	1.50E-14	93.9568032	1.89926E-19
6	GeneID:100189189	*TRNAQ16*	27487308	27487379	rs34573979	27480526	1.74E-08	2.47E-13	93.79062564	2.06024E-19
6	GeneID:790951	*TRNAS4*	27499987	27500068	rs13195636	27509493	2.93E-08	1.87E-13	93.30050868	2.61889E-19
6	GeneID:100507444	*PPP1R2P1*	32844255	32847851	rs115591082	32844103	5.24E-06	1.60E-15	92.4549026	3.96157E-19
10	GeneID:100500866	*MIR3941*	124176481	124176583	rs79508005	124164620	2.69E-12	4.14E-09	91.89289245	5.21572E-19
6	GeneID:23540	*TRNAS3*	27473607	27473688	rs34573979	27480526	1.56E-08	1.10E-12	91.02606617	7.97105E-19
6	GeneID:23437	*TRNAS2*	27463593	27463674	rs58616630	27474715	1.71E-08	1.05E-12	90.93131171	8.34927E-19
6	GeneID:100189292	*TRNAD14*	27471523	27471594	rs34573979	27480526	1.64E-08	1.15E-12	90.82158559	8.80972E-19
6	GeneID:100189247	*TRNAS19*	27470818	27470899	rs34573979	27480526	1.70E-08	1.19E-12	90.69439002	9.37534E-19
10	GeneID:59338	*PLEKHA1*	124134094	124191871	rs117634244	124199016	5.73E-12	7.35E-09	89.22825351	1.92057E-18
6	GeneID:394263	*MUC21*	30951485	30957675	rs141725002	30971976	1.79E-02	3.64E-18	88.35338427	2.94592E-18
6	GeneID:26695	*OR2U1P*	29230436	29231856	rs144447022	29244219	6.18E-09	1.28E-11	87.97426425	3.54584E-18
6	GeneID:26694	*OR2U2P*	29236242	29237198	rs144447022	29244219	7.17E-09	1.48E-11	87.3756728	4.7512E-18
6	GeneID:8348	*HIST1H2BO*	27861203	27861669	rs13199649	27868792	4.66E-08	2.30E-12	87.35609589	4.79688E-18
6	GeneID:8336	*HIST1H2AM*	27860477	27860963	rs13199649	27868792	4.67E-08	2.31E-12	87.35156672	4.80751E-18
6	GeneID:8356	*HIST1H3J*	27858093	27858570	rs13199649	27868792	4.98E-08	2.46E-12	87.08946835	5.4646E-18
**Chr**	**Gene.id**	**Name**	**Start**	**End**	**SNP.id**	**SNP.pos**	**pval(headache)**	**pval(hypoPAN)**	**X^2,4df**	**FCP**

6	GeneID:100189077	*TRNAS10*	27446591	27446672	rs7749305	27446566	1.40E-07	9.38E-13	86.95168386	5.84529E-18
6	GeneID:100189158	*TRNAD8*	27447453	27447524	rs7749305	27446566	1.41E-07	9.47E-13	86.9139405	5.95412E-18
6	GeneID:645927	*MCFD2P1*	27375502	27377470	rs34071253	27391802	1.07E-08	1.34E-11	86.7856888	6.33928E-18
6	GeneID:100147813	*RNU7-26P*	27865284	27865343	rs61742093	27879982	4.37E-08	3.41E-12	86.69975939	6.61118E-18
8	GeneID:100421823	*LOC100421823*	8011887	8074438	rs2955581	8088877	2.24E-05	7.20E-15	86.5410639	7.14433E-18
6	GeneID:26212	*OR2B6*	27925019	27925960	rs149583087	27912437	1.69E-08	9.74E-12	86.50191337	7.28234E-18
6	GeneID:442190	*OR2B4P*	29258373	29259527	rs144447022	29244219	9.80E-09	2.02E-11	86.12710554	8.74613E-18
6	GeneID:346157	*ZNF391*	27356524	27369227	rs67859638	27357978	1.21E-08	1.66E-11	86.10730487	8.83117E-18
6	GeneID:11119	*BTN3A1*	26402465	26415444	rs2072803	26392515	1.17E-10	1.83E-09	85.97741861	9.40983E-18
6	GeneID:81406	*OR2W6P*	27905182	27906179	rs149583087	27912437	1.95E-08	1.12E-11	85.93045787	9.62825E-18
6	GeneID:442175	*RPLP2P1*	27932953	27933234	rs28360499	27945396	2.22E-08	1.98E-11	84.53423567	1.9045E-17
6	GeneID:3879	*KRT18P1*	28936847	28938244	rs148696809	28934352	5.80E-09	7.71E-11	84.5014265	1.93527E-17
6	GeneID:10385	*BTN2A2*	26383324	26395102	rs2072803	26392515	1.73E-10	2.72E-09	84.39836566	2.03518E-17
6	GeneID:100189315	*TRNAM17*	27870271	27870342	rs13218875	27884012	3.31E-08	1.66E-11	84.085962	2.37065E-17
6	GeneID:81407	*OR2W4P*	27944929	27945851	rs28360499	27945396	2.52E-08	2.25E-11	84.02749814	2.43932E-17
6	GeneID:100189314	*TRNAG29*	27870686	27870756	rs13218875	27884012	3.41E-08	1.71E-11	83.96894196	2.51008E-17
6	GeneID:54718	*BTN2A3P*	26421619	26430816	rs9379875	26444732	4.74E-10	1.30E-09	83.86195551	2.64471E-17
6	GeneID:81697	*OR2B2*	27878963	27880174	rs13218875	27884012	3.53E-08	1.77E-11	83.83143989	2.68442E-17
6	GeneID:81694	*OR2W2P*	28001693	28002640	rs13193295	28003228	2.65E-08	2.62E-11	83.61867654	2.97834E-17
6	GeneID:100189111	*TRNAM8*	28921042	28921114	rs148696809	28934352	7.63E-09	1.01E-10	83.40820107	3.30072E-17
6	GeneID:222698	*NKAPL*	28227098	28228736	rs17720293	28214698	1.85E-08	4.32E-11	83.34424167	3.40543E-17
6	GeneID:7746	*ZNF193*	28193029	28201265	rs17720293	28214698	2.28E-08	3.50E-11	83.34274051	3.40793E-17
6	GeneID:100419608	*LOC100419608*	28083427	28083998	rs66886492	28089731	2.61E-08	3.08E-11	83.32989143	3.42938E-17
6	GeneID:81695	*OR2B7P*	28014213	28015147	rs13193295	28003228	2.81E-08	3.10E-11	83.16403502	3.71865E-17
6	GeneID:140468	*COX11P1*	28414697	28415407	rs67381177	28411941	3.40E-08	2.89E-11	82.92491793	4.17915E-17
6	GeneID:387032	*ZKSCAN4*	28212490	28220002	rs17720293	28214698	2.24E-08	4.39E-11	82.92347385	4.1821E-17
6	GeneID:100420941	*LOC100420941*	26478093	26478749	rs13195401	26463574	7.28E-10	1.38E-09	82.88848873	4.25414E-17
6	GeneID:26719	*OR2E1P*	28423307	28423934	rs67381177	28411941	3.44E-08	2.92E-11	82.88842956	4.25427E-17
**Chr**	**Gene.id**	**Name**	**Start**	**End**	**SNP.id**	**SNP.pos**	**pval(headache)**	**pval(hypoPAN)**	**X^2,4df**	**FCP**

6	GeneID:100189159	*TRNAS13*	28180815	28180896	rs13197633	28174757	3.37E-08	3.03E-11	82.85204777	4.33051E-17
6	GeneID:100419756	*LOC100419756*	28376002	28377027	rs35883476	28368508	3.09E-08	3.30E-11	82.85133352	4.33202E-17
6	GeneID:340192	*IQCB2P*	27978329	27980680	rs71559067	27994416	3.19E-08	3.24E-11	82.8257375	4.38649E-17
6	GeneID:222696	*ZSCAN23*	28400432	28411279	rs13201681	28394680	3.46E-08	3.28E-11	82.64111646	4.80022E-17
6	GeneID:222699	*TOB2P1*	28183116	28186707	rs13197633	28174757	3.70E-08	3.33E-11	82.47984963	5.1934E-17
6	GeneID:80345	*ZSCAN16*	28092387	28097857	rs66886492	28089731	3.25E-08	4.03E-11	82.35099987	5.53054E-17
6	GeneID:9753	*ZSCAN12*	28346598	28367544	rs35883476	28368508	3.71E-08	3.56E-11	82.33916493	5.56259E-17
6	GeneID:7211	*TRMEP1*	28448503	28448575	rs34676049	28453618	2.74E-08	4.98E-11	82.26957909	5.75478E-17
6	GeneID:65944	*OR2B8P*	28020906	28022043	rs13200214	28017250	3.71E-08	3.74E-11	82.24093851	5.8358E-17
6	GeneID:8344	*HIST1H2BE*	26184024	26184458	rs67575965	26196593	5.08E-09	2.73E-10	82.2388561	5.84173E-17
6	GeneID:100189344	*TRNAT16*	28442329	28442402	rs34676049	28453618	2.76E-08	5.02E-11	82.23818068	5.84366E-17
6	GeneID:100189035	*TRNAT5*	28456770	28456843	rs34676049	28453618	2.70E-08	5.22E-11	82.20940875	5.92631E-17
6	GeneID:7209	*TRNAL47P*	28446350	28446431	rs34676049	28453618	2.85E-08	5.18E-11	82.11286217	6.21228E-17
6	GeneID:100189118	*TRNAL12*	28446400	28446481	rs34676049	28453618	2.85E-08	5.18E-11	82.11286217	6.21228E-17
6	GeneID:100189131	*TRNAI6*	27599200	27599293	rs35715914	27592003	2.50E-08	6.28E-11	81.98926792	6.59858E-17
6	GeneID:100189300	*TRNAT13*	27586135	27586208	rs35715914	27592003	2.53E-08	6.34E-11	81.94941647	6.72819E-17
6	GeneID:100189352	*TRNAS24*	27509554	27509635	rs35848276	27521096	3.03E-08	5.42E-11	81.90006301	6.89223E-17
6	GeneID:100189336	*TRNAI20*	27144994	27145067	rs34569203	27153984	2.62E-10	6.30E-09	81.88983321	6.92673E-17
6	GeneID:94017	*TRNAS1*	27513468	27513549	rs35848276	27521096	3.07E-08	5.48E-11	81.85407746	7.04867E-17
6	GeneID:100189268	*TRNAQ21*	27515531	27515602	rs35848276	27521096	3.08E-08	5.50E-11	81.8399524	7.09743E-17
6	GeneID:222701	*ZNF192P2*	28155716	28157189	rs13195291	28169241	3.64E-08	4.76E-11	81.79384808	7.25895E-17
6	GeneID:651302	*ZNF192P1*	28129551	28137376	rs35749575	28114818	3.58E-08	4.92E-11	81.76053789	7.37793E-17
6	GeneID:11120	*BTN2A1*	26458153	26476849	rs9379875	26444732	7.43E-10	2.47E-09	81.67876452	7.67833E-17
6	GeneID:8360	*HIST1H4D*	26188938	26189304	rs67575965	26196593	5.99E-09	3.22E-10	81.57811479	8.06492E-17
6	GeneID:100189070	*TRNAS9*	27521192	27521273	rs35848276	27521096	3.30E-08	5.89E-11	81.56616024	8.11211E-17
8	GeneID:286042	*FLJ10661*	8086092	8102387	rs2955581	8088877	1.64E-04	1.19E-14	81.55565091	8.15382E-17
6	GeneID:100129195	*LOC100129195*	28058336	28105071	rs35749575	28114818	4.09E-08	4.84E-11	81.52529896	8.2755E-17
6	GeneID:100189219	*TRNAY8*	26595102	26595190	rs34781270	26593037	8.07E-10	2.46E-09	81.5195759	8.29865E-17
**Chr**	**Gene.id**	**Name**	**Start**	**End**	**SNP.id**	**SNP.pos**	**pval(headache)**	**pval(hypoPAN)**	**X^2,4df**	**FCP**

6	GeneID:221584	*ZSCAN12P1*	28058929	28063493	rs35952432	28074901	4.07E-08	4.94E-11	81.49268467	8.40827E-17
6	GeneID:285819	*LOC285819*	26472172	26482737	rs13195401	26463574	1.02E-09	1.97E-09	81.48939526	8.42178E-17
6	GeneID:7754	*ZNF204P*	27325602	27343153	rs67859638	27357978	1.16E-08	1.77E-10	81.44457241	8.60803E-17
6	GeneID:7745	*ZNF192*	28109716	28125236	rs35749575	28114818	3.81E-08	5.49E-11	81.41679022	8.72554E-17
6	GeneID:696	*BTN1A1*	26501495	26510653	rs34148261	26511744	1.28E-09	1.65E-09	81.39491814	8.81917E-17
6	GeneID:100189143	*TRNAT7*	27130050	27130123	rs13194053	27143883	4.41E-10	5.00E-09	81.31154057	9.18541E-17
6	GeneID:80317	*ZKSCAN3*	28317691	28336954	rs13214023	28332141	5.02E-08	4.46E-11	81.28425987	9.30851E-17
6	GeneID:100189012	*TRNAR5*	27529963	27530049	rs35848276	27521096	3.57E-08	6.39E-11	81.24452731	9.49075E-17
6	GeneID:100128240	*LOC100128240*	27528605	27529550	rs35848276	27521096	3.57E-08	6.39E-11	81.24324134	9.49671E-17
6	GeneID:100189074	*TRNAQ10*	27763640	27763711	rs35037868	27759115	7.56E-08	3.08E-11	81.20616663	9.67009E-17
6	GeneID:100189526	*TRNAS32P*	27261671	27261744	rs13207082	27251379	2.15E-09	1.13E-09	81.12206061	1.00752E-16
6	GeneID:8341	*HIST1H2BN*	27806440	27806888	rs35202262	27799514	1.38E-07	1.77E-11	81.11098539	1.01298E-16
6	GeneID:8330	*HIST1H2AK*	27805658	27806117	rs35202262	27799514	1.38E-07	1.77E-11	81.1100317	1.01345E-16
6	GeneID:100189002	*TRNAS5*	27265775	27265856	rs13207082	27251379	2.16E-09	1.13E-09	81.10502551	1.01593E-16
6	GeneID:100189329	*TRNAQ27*	27263212	27263283	rs13207082	27251379	2.22E-09	1.16E-09	80.99645276	1.07121E-16
6	GeneID:100189162	*TRNAY7*	26577332	26577420	rs13190739	26587373	9.73E-10	2.77E-09	80.91068809	1.11699E-16
6	GeneID:394255	*RPS10P1*	26202351	26202943	rs67575965	26196593	7.07E-09	3.81E-10	80.91013102	1.11729E-16
6	GeneID:8343	*HIST1H2BF*	26199787	26200216	rs67575965	26196593	7.17E-09	3.86E-10	80.8588828	1.14558E-16
6	GeneID:100189372	*TRNAI22*	26745255	26745328	rs13213200	26755915	2.38E-10	1.16E-08	80.85817418	1.14598E-16
6	GeneID:100189214	*TRNAQ17*	27759135	27759206	rs35037868	27759115	8.18E-08	3.53E-11	80.77258499	1.19485E-16
6	GeneID:3013	*HIST1H2AD*	26199012	26199471	rs67575965	26196593	7.38E-09	3.97E-10	80.74349059	1.21193E-16
6	GeneID:64288	*ZNF323*	28292514	28324048	rs13214023	28332141	5.49E-08	5.45E-11	80.70102993	1.2373E-16
6	GeneID:8351	*HIST1H3D*	26197012	26199464	rs67575965	26196593	7.56E-09	4.07E-10	80.64715251	1.27026E-16
6	GeneID:7741	*ZNF187*	28234788	28245981	rs13208096	28225311	6.43E-08	5.03E-11	80.54608303	1.33446E-16
6	GeneID:100189337	*TRNAY12*	26575798	26575887	rs13190739	26587373	1.07E-09	3.03E-09	80.54595345	1.33455E-16
6	GeneID:100126484	*TRNAM-CAU*	26735574	26735646	rs35144506	26739487	5.93E-10	5.49E-09	80.5321696	1.34355E-16
6	GeneID:10384	*BTN3A3*	26440700	26453643	rs9379875	26444732	6.71E-10	4.89E-09	80.51499179	1.35486E-16
6	GeneID:442179	*OR1F12*	28041026	28042002	rs71559070	28038929	5.63E-08	6.48E-11	80.30493295	1.50107E-16
**Chr**	**Gene.id**	**Name**	**Start**	**End**	**SNP.id**	**SNP.pos**	**pval(headache)**	**pval(hypoPAN)**	**X^2,4df**	**FCP**

6	GeneID:100189205	*TRNAS15*	27640229	27640310	rs13217620	27653120	3.55E-08	1.03E-10	80.29625059	1.50744E-16
6	GeneID:84547	*PGBD1*	28249314	28270326	rs33932084	28268824	4.40E-08	8.45E-11	80.26636985	1.52958E-16
6	GeneID:8362	*HIST1H4K*	27798952	27799305	rs35202262	27799514	1.34E-07	2.79E-11	80.25015909	1.54172E-16
6	GeneID:100189161	*TRNAV17*	27648885	27648957	rs13217620	27653120	3.64E-08	1.06E-10	80.1889959	1.58842E-16
11	GeneID:6506	*SLC1A2*	35272752	35441532	rs10836381	35388274	7.77E-03	5.00E-16	80.18047438	1.59503E-16
6	GeneID:100507241	*LOC100507241*	28046570	28048908	rs35902873	28058949	6.06E-08	6.97E-11	80.01138002	1.73218E-16
6	GeneID:100189428	*TRNAR29*	27638344	27638416	rs13217620	27653120	3.82E-08	1.11E-10	79.99540064	1.74574E-16
6	GeneID:8363	*HIST1H4J*	27791903	27792258	rs35202262	27799514	1.21E-07	3.81E-11	79.84101329	1.88228E-16
6	GeneID:7718	*ZNF165*	28048482	28057341	rs34166054	28065801	6.34E-08	7.29E-11	79.83213162	1.89046E-16
6	GeneID:8367	*HIST1H4E*	26204873	26205249	rs67575965	26196593	6.51E-09	7.27E-10	79.78316429	1.93615E-16
6	GeneID:100189340	*TRNAT15*	27652474	27652547	rs13217620	27653120	4.05E-08	1.18E-10	79.76601207	1.95242E-16
6	GeneID:100507141	*LOC100507141*	26864941	26865525	rs77666565	26851415	1.32E-09	3.67E-09	79.74061295	1.97676E-16
6	GeneID:729400	*LOC729400*	26866104	26868093	rs77666565	26851415	1.32E-09	3.68E-09	79.72333647	1.99349E-16
6	GeneID:100189071	*TRNAI4*	27655967	27656040	rs13217620	27653120	4.15E-08	1.21E-10	79.67254118	2.04349E-16
6	GeneID:10337	*HIST1H4PS1*	27774945	27775115	rs13195728	27771106	1.50E-07	3.41E-11	79.62291455	2.09356E-16
6	GeneID:8969	*HIST1H2AG*	27100817	27101314	rs67330695	27103654	7.43E-10	6.93E-09	79.61310249	2.10361E-16
6	GeneID:85236	*HIST1H2BK*	27106072	27114637	rs61240102	27124904	7.30E-10	7.07E-09	79.61249299	2.10423E-16
6	GeneID:80864	*EGFL8*	32132382	32136062	rs192471087	32119730	1.94E-03	2.72E-15	79.57043193	2.14785E-16
6	GeneID:8329	*HIST1H2AI*	27775977	27776445	rs13195728	27771106	1.53E-07	3.47E-11	79.56167123	2.15704E-16
6	GeneID:8340	*HIST1H2BL*	27775257	27775709	rs13195728	27771106	1.54E-07	3.49E-11	79.53223774	2.18823E-16
6	GeneID:8970	*HIST1H2BJ*	27100095	27100575	rs67330695	27103654	7.66E-10	7.14E-09	79.49494662	2.2284E-16
6	GeneID:100189272	*TRNAY10*	26569086	26569176	rs72844462	26563864	1.98E-09	2.86E-09	79.42749913	2.30292E-16
6	GeneID:352957	*MICF*	29819964	29821829	rs114853934	29812896	8.48E-04	6.84E-15	79.37604032	2.36145E-16
6	GeneID:8345	*HIST1H2BH*	26251879	26252303	rs55706012	26266311	1.36E-07	4.37E-11	79.32543036	2.42046E-16
6	GeneID:8355	*HIST1H3G*	26271146	26271612	rs55706012	26266311	1.37E-07	4.40E-11	79.30066543	2.44987E-16
6	GeneID:8342	*HIST1H2BM*	27782822	27783267	rs13195728	27771106	1.63E-07	3.71E-11	79.29069749	2.46181E-16
6	GeneID:100189041	*TRNAS7*	27177628	27177709	rs4713071	27187919	4.97E-10	1.23E-08	79.27424841	2.48164E-16
6	GeneID:100009603	*TRNAA2*	26572092	26572164	rs72844462	26563864	2.07E-09	2.99E-09	79.24864159	2.51282E-16
**Chr**	**Gene.id**	**Name**	**Start**	**End**	**SNP.id**	**SNP.pos**	**pval(headache)**	**pval(hypoPAN)**	**X^2,4df**	**FCP**

6	GeneID:8346	*HIST1H2BI*	26273204	26273640	rs55706012	26266311	1.39E-07	4.46E-11	79.24719081	2.5146E-16
6	GeneID:442172	*LOC442172*	27235885	27237008	rs35982103	27243134	1.09E-09	5.72E-09	79.24119902	2.52196E-16
6	GeneID:8357	*HIST1H3H*	27777842	27778314	rs13195728	27771106	1.65E-07	3.76E-11	79.23952862	2.52402E-16
6	GeneID:8331	*HIST1H2AJ*	27782080	27782518	rs13195728	27771106	1.66E-07	3.78E-11	79.22097262	2.54696E-16
8	GeneID:83655	*LINC00208*	11434044	11438850	rs12549150	11422936	6.22E-06	1.03E-12	79.18193294	2.59592E-16
6	GeneID:100507025	*LOC100507025*	26281278	26285763	rs55706012	26266311	1.41E-07	4.54E-11	79.17812225	2.60075E-16
6	GeneID:100189167	*TRNAR17*	27181623	27181695	rs4713071	27187919	5.12E-10	1.26E-08	79.15730992	2.62728E-16
6	GeneID:7202	*TRNAI1*	27241739	27241812	rs35982103	27243134	1.11E-09	5.86E-09	79.1467742	2.64081E-16
6	GeneID:8333	*HIST1H2APS4*	26272421	26272768	rs55706012	26266311	1.43E-07	4.60E-11	79.1213984	2.6737E-16
6	GeneID:100189368	*TRNAV28*	27173867	27173939	rs4713071	27187919	5.18E-10	1.28E-08	79.11462738	2.68254E-16
6	GeneID:100189099	*TRNAR10*	27182952	27183024	rs4713071	27187919	5.21E-10	1.29E-08	79.08876493	2.71659E-16
6	GeneID:100189213	*TRNAI11*	27242990	27243063	rs35982103	27243134	1.13E-09	5.94E-09	79.08628032	2.71988E-16
6	GeneID:442171	*RPL10P2*	27179023	27179663	rs4713071	27187919	5.26E-10	1.30E-08	79.04939269	2.76925E-16
6	GeneID:100507547	*LOC100507547*	32120579	32122142	rs192471087	32119730	2.27E-03	3.18E-15	78.94378656	2.9156E-16
6	GeneID:100189157	*TRNAS12*	27065085	27065166	rs34388707	27050396	8.80E-10	9.16E-09	78.71991142	3.25191E-16
6	GeneID:9374	*PPT2*	32121229	32131458	rs192471087	32119730	2.40E-03	3.37E-15	78.7154636	3.25897E-16
6	GeneID:100532746	*PPT2-EGFL8*	32121776	32136062	rs192471087	32119730	2.40E-03	3.37E-15	78.71499848	3.25971E-16
6	GeneID:100189462	*TRNAI28P*	27251864	27251937	rs35982103	27243134	1.35E-09	6.13E-09	78.66439461	3.34115E-16
6	GeneID:100189345	*TRNAM18*	27300764	27300835	rs71537559	27309779	3.24E-08	2.62E-10	78.61877679	3.4163E-16
6	GeneID:100189363	*TRNAV27*	27248049	27248121	rs35982103	27243134	1.38E-09	6.25E-09	78.58944831	3.4655E-16
6	GeneID:729392	*POM121L6P*	26838446	26865940	rs77666565	26851415	1.77E-09	4.93E-09	78.55523785	3.52379E-16
6	GeneID:100189182	*TRNAK19*	27302769	27302841	rs71537559	27309779	3.33E-08	2.69E-10	78.50801222	3.60588E-16
6	GeneID:100189172	*TRNAP10*	27059521	27059592	rs6904071	27047256	8.75E-10	1.03E-08	78.4980025	3.62352E-16
6	GeneID:100189408	*TRNAV31*	27258405	27258477	rs13207082	27251379	2.01E-09	5.32E-09	78.15142159	4.29058E-16
6	GeneID:7212	*TRNAM2*	27745664	27745735	rs35037868	27759115	7.31E-08	1.48E-10	78.1305794	4.3344E-16
6	GeneID:100507173	*LOC100507173*	27661814	27678001	rs13217620	27653120	6.23E-08	1.82E-10	78.04009077	4.52989E-16
6	GeneID:100129755	*RSL24D1P1*	27748462	27748890	rs35037868	27759115	7.50E-08	1.52E-10	78.02426121	4.56498E-16
6	GeneID:791231	*TRNAV8*	27618707	27618779	rs144969912	27616843	2.89E-08	3.98E-10	78.00835668	4.60051E-16
**Chr**	**Gene.id**	**Name**	**Start**	**End**	**SNP.id**	**SNP.pos**	**pval(headache)**	**pval(hypoPAN)**	**X^2,4df**	**FCP**

6	GeneID:100189301	*TRNAL28*	27573417	27573524	rs34105070	27560805	3.65E-08	3.39E-10	77.86225307	4.94013E-16
6	GeneID:387046	*RPL8P1*	27620370	27620512	rs144969912	27616843	3.02E-08	4.17E-10	77.82984485	5.01879E-16
17	GeneID:644256	*DND1P2*	44336419	44337979	rs117629202	44344596	1.01E-15	1.27E-02	77.79664949	5.10067E-16
6	GeneID:353009	*3.8-1.4*	29833692	29834864	rs62389319	29847388	1.35E-03	9.52E-15	77.79242125	5.11119E-16
17	GeneID:644253	*MAPK8IPP*	44320972	44322410	rs111535660	44314148	7.46E-16	1.88E-02	77.61617799	5.56971E-16
6	GeneID:100189038	*TRNAL8*	27570348	27570454	rs34105070	27560805	3.95E-08	3.67E-10	77.54370207	5.76999E-16
6	GeneID:100189390	*TRNAK31*	27543846	27543918	rs10484399	27534528	5.29E-08	2.76E-10	77.53264593	5.80117E-16
11	GeneID:960	*CD44*	35160417	35253949	rs76276019	35239888	1.43E-02	1.06E-15	77.45699169	6.01908E-16
6	GeneID:29777	*ABT1*	26597171	26600278	rs34781270	26593037	8.44E-10	1.83E-08	77.41550682	6.14203E-16
6	GeneID:100189101	*TRNAV15*	27696327	27696399	rs34409918	27685348	1.15E-07	1.35E-10	77.39310137	6.20947E-16
6	GeneID:387317	*VN1R11P*	27051071	27051995	rs6904071	27047256	1.02E-09	1.57E-08	77.34208748	6.3658E-16
6	GeneID:100189297	*TRNAM15*	27560600	27560671	rs34105070	27560805	4.16E-08	3.86E-10	77.33889989	6.37569E-16
6	GeneID:79692	*ZNF322*	26634611	26659980	rs149123117	26670618	7.23E-10	2.26E-08	77.30300543	6.48822E-16
6	GeneID:387316	*VN1R10P*	27292695	27293631	rs56401801	27301512	4.43E-08	3.78E-10	77.25868446	6.6299E-16
6	GeneID:100189200	*TRNAD9*	27551236	27551307	rs34105070	27560805	4.26E-08	3.96E-10	77.24061023	6.68856E-16
6	GeneID:100189053	*TRNAK8*	27559593	27559665	rs34105070	27560805	4.29E-08	3.98E-10	77.21691354	6.76625E-16
6	GeneID:100189164	*TRNAL16*	27688898	27688980	rs34409918	27685348	1.22E-07	1.43E-10	77.18258841	6.8804E-16
6	GeneID:100189374	*TRNAT18*	27694473	27694546	rs34409918	27685348	1.23E-07	1.45E-10	77.13072243	7.05654E-16
6	GeneID:83954	*FKSG83*	27292578	27293742	rs56401801	27301512	4.58E-08	3.90E-10	77.12637058	7.07152E-16
6	GeneID:100189095	*TRNAI5*	27636362	27636435	rs56405707	27640246	4.12E-08	4.44E-10	77.08202467	7.22601E-16
6	GeneID:11118	*BTN3A2*	26365398	26378548	rs2072803	26392515	1.58E-10	1.21E-07	76.98831138	7.56368E-16
6	GeneID:8364	*HIST1H4C*	26104176	26104565	rs2237228	26104630	5.85E-08	3.50E-10	76.85268211	8.08051E-16
6	GeneID:394254	*GPR89P*	27704354	27706293	rs13202291	27698857	1.60E-07	1.30E-10	76.82036394	8.20878E-16
6	GeneID:387036	*GUSBP2*	26839266	26924333	rs77666565	26851415	2.51E-09	8.36E-09	76.80431783	8.27321E-16
17	GeneID:100132570	*LOC100132570*	44344293	44347172	rs117629202	44344596	1.38E-15	1.73E-02	76.54288482	9.39725E-16
6	GeneID:8334	*HIST1H2AC*	26124373	26124918	rs13200797	26122957	7.26E-08	3.37E-10	76.50160434	9.58818E-16
6	GeneID:8347	*HIST1H2BC*	26123695	26124132	rs13200797	26122957	7.28E-08	3.38E-10	76.48848707	9.64966E-16
6	GeneID:3077	*HFE*	26087509	26095469	rs71557316	26072981	4.78E-08	5.29E-10	76.43504736	9.90423E-16
**Chr**	**Gene.id**	**Name**	**Start**	**End**	**SNP.id**	**SNP.pos**	**pval(headache)**	**pval(hypoPAN)**	**X^2,4df**	**FCP**

6	GeneID:94026	*POM121L2*	27276842	27280011	rs56114371	27274834	2.66E-08	9.85E-10	76.36481813	1.0249E-15
6	GeneID:100379623	*LOC100379623*	26164261	26168404	rs17598658	26175866	8.29E-08	3.23E-10	76.31475282	1.05021E-15
6	GeneID:387320	*VN1R14P*	26631313	26631964	rs67777156	26633711	1.99E-09	1.58E-08	75.99116658	1.22954E-15
6	GeneID:3017	*HIST1H2BD*	26158349	26171577	rs17598658	26175866	1.06E-07	4.13E-10	75.33739384	1.69064E-15
6	GeneID:85303	*HIST1H2APS2*	25882154	25882644	rs13208859	25894609	4.48E-08	1.07E-09	75.15206084	1.85035E-15
17	GeneID:644354	*LOC644354*	43578301	43578645	rs189777036	43574229	1.73E-15	2.86E-02	75.0882999	1.90872E-15
6	GeneID:10246	*SLC17A2*	25912982	25930946	rs35169013	25918027	4.09E-08	1.22E-09	75.06714309	1.92848E-15
6	GeneID:100652981	*LOC100652981*	26854360	26854727	rs77666565	26851415	1.36E-09	3.69E-08	75.05993199	1.93527E-15
17	GeneID:55073	*LRRC37A4*	43583249	43597889	rs189777036	43574229	1.46E-15	3.73E-02	74.89222403	2.09997E-15
8	GeneID:640	*BLK*	11351521	11422108	rs12549150	11422936	1.65E-05	3.82E-12	74.60762715	2.41214E-15
6	GeneID:10786	*SLC17A3*	25845328	25874471	rs6913795	25848025	5.33E-08	1.33E-09	74.37318102	2.70383E-15
6	GeneID:8335	*HIST1H2AB*	26033320	26033796	rs3752417	26045905	3.79E-08	2.02E-09	74.21714265	2.91726E-15
6	GeneID:3006	*HIST1H1C*	26055968	26056699	rs3752417	26045905	3.96E-08	1.95E-09	74.20190483	2.93898E-15
6	GeneID:8358	*HIST1H3B*	26031817	26032288	rs3752417	26045905	3.87E-08	2.06E-09	74.14132277	3.02696E-15
6	GeneID:6568	*SLC17A1*	25783125	25832287	rs9467607	25809477	3.82E-08	2.31E-09	73.93606086	3.34508E-15
8	GeneID:9258	*MFHAS1*	8641999	8751131	rs7013471	8687325	8.85E-05	1.21E-12	73.54358354	4.04931E-15
6	GeneID:8352	*HIST1H3C*	26045639	26046097	rs3752417	26045905	3.84E-08	2.97E-09	73.41908965	4.30228E-15
6	GeneID:10341	*HIST1H2APS5*	26044128	26044778	rs3752417	26045905	3.85E-08	2.98E-09	73.40736564	4.3269E-15
6	GeneID:100270746	*LOC100270746*	26987145	26988085	rs13217285	26999845	4.70E-09	2.45E-08	73.40587539	4.33004E-15
6	GeneID:3018	*HIST1H2BB*	26043455	26043885	rs3752417	26045905	3.94E-08	3.05E-09	73.31347735	4.52922E-15
6	GeneID:100189028	*TRNAI2*	26988125	26988218	rs13217285	26999845	4.86E-09	2.53E-08	73.27108554	4.62364E-15
17	GeneID:9842	*PLEKHM1*	43513266	43568146	rs189777036	43574229	3.43E-15	3.98E-02	73.06042555	5.12284E-15
6	GeneID:387321	*VN1R12P*	27001125	27002122	rs35741362	27007687	4.07E-09	3.36E-08	73.05815502	5.12851E-15
6	GeneID:10338	*HIST1H1PS2*	26016335	26017069	rs10484435	26031811	7.15E-08	1.99E-09	72.97847872	5.33128E-15
6	GeneID:3024	*HIST1H1A*	26017260	26018040	rs10484435	26031811	7.42E-08	2.06E-09	72.83240842	5.72405E-15
6	GeneID:100131289	*LOC100131289*	27729523	27730966	rs35501037	27739566	1.70E-07	9.06E-10	72.81917596	5.76103E-15
6	GeneID:8366	*HIST1H4B*	26027124	26027480	rs10484435	26031811	7.48E-08	2.08E-09	72.79749726	5.82212E-15
6	GeneID:221527	*ZBTB12*	31867394	31869769	rs115062572	31862876	1.46E-02	1.10E-14	72.73488804	6.00224E-15
**Chr**	**Gene.id**	**Name**	**Start**	**End**	**SNP.id**	**SNP.pos**	**pval(headache)**	**pval(hypoPAN)**	**X^2,4df**	**FCP**

6	GeneID:8350	*HIST1H3A*	26020718	26021186	rs10484435	26031811	7.65E-08	2.13E-09	72.7111107	6.07209E-15
6	GeneID:8359	*HIST1H4A*	26021907	26022278	rs10484435	26031811	7.66E-08	2.13E-09	72.70531909	6.08923E-15
6	GeneID:791230	*TRNAV7*	27721179	27721251	rs17693963	27710165	1.55E-07	1.10E-09	72.61841511	6.35225E-15
6	GeneID:10475	*TRIM38*	25963071	25985358	rs13203673	25979122	7.55E-08	2.30E-09	72.58020918	6.47145E-15
6	GeneID:100189067	*TRNAL10*	27198334	27198416	rs4713071	27187919	5.29E-10	3.87E-07	72.24975746	7.60028E-15
17	GeneID:9884	*LRRC37A*	44372497	44415160	rs2458218	44361383	6.20E-15	3.40E-02	72.19211396	7.81645E-15
6	GeneID:10050	*SLC17A4*	25754927	25781403	rs13200921	25790378	3.74E-08	7.56E-09	71.60596751	1.03955E-14
8	GeneID:137075	*CLDN23*	8559666	8561617	rs1109618	8571364	5.89E-05	5.23E-12	71.43558538	1.12937E-14
6	GeneID:100189310	*TRNAI17*	27205350	27205423	rs35589403	27219491	9.44E-10	3.84E-07	71.10871208	1.32397E-14
6	GeneID:10590	*SCGN*	25652429	25702011	rs35436081	25700342	6.86E-08	5.57E-09	71.00429062	1.39294E-14
6	GeneID:10279	*PRSS16*	27215502	27224399	rs35589403	27219491	1.09E-09	3.94E-07	70.76620882	1.56391E-14
6	GeneID:3010	*HIST1H1T*	26107640	26108364	rs2237228	26104630	8.76E-08	5.99E-09	70.36780148	1.8982E-14
6	GeneID:100189014	*TRNAV12*	27203288	27203360	rs13212921	27205422	1.91E-09	3.73E-07	69.75199792	2.56065E-14
6	GeneID:58530	*LY6G6D*	31683133	31685581	rs148571474	31672712	1.03E-02	7.97E-14	69.4733411	2.93204E-14
6	GeneID:79136	*LY6G6E*	31679753	31681842	rs148571474	31672712	1.05E-02	8.12E-14	69.40261298	3.03458E-14
17	GeneID:100423004	*MIR4315-1*	43552729	43552801	rs148269941	43540472	4.35E-14	2.06E-02	69.30272625	3.18552E-14
6	GeneID:221613	*HIST1H2AA*	25726291	25726790	rs34493019	25714959	1.72E-07	5.36E-09	69.24491686	3.27628E-14
6	GeneID:255626	*HIST1H2BA*	25727137	25727573	rs34493019	25714959	1.75E-07	5.47E-09	69.16351259	3.40849E-14
6	GeneID:100616230	*MIR4646*	31668806	31668868	rs148571474	31672712	1.18E-02	9.12E-14	68.93675252	3.80553E-14
6	GeneID:259215	*LY6G6F*	31674684	31678372	rs148571474	31672712	1.28E-02	9.94E-14	68.59072053	4.50227E-14
6	GeneID:7920	*ABHD16A*	31654726	31671137	rs148571474	31672712	1.29E-02	1.00E-13	68.55457508	4.58203E-14
6	GeneID:7231	*TRNAR2*	26537726	26537798	rs66941101	26530376	5.18E-10	2.83E-06	68.31505735	5.14745E-14
6	GeneID:100189227	*TRNAV21*	26538282	26538354	rs66941101	26530376	5.21E-10	2.84E-06	68.29066969	5.20879E-14
17	GeneID:4905	*NSF*	44668035	44834830	rs1378358	44787312	1.04E-13	1.89E-02	67.72021834	6.87179E-14
6	GeneID:100189288	*TRNAL26*	26521436	26521518	rs66941101	26530376	5.88E-10	3.48E-06	67.64696618	7.12066E-14
6	GeneID:100189319	*TRNAT14*	26533145	26533218	rs66941101	26530376	6.04E-10	3.51E-06	67.57656537	7.36832E-14
19	GeneID:100507004	*LOC100507004*	4800220	4801278	rs115654559	4797674	2.77E-02	7.93E-14	67.50487334	7.62936E-14
6	GeneID:3139	*HLA-L*	30227339	30234728	rs188226252	30236078	2.40E-02	1.04E-13	67.25031481	8.63319E-14
**Chr**	**Gene.id**	**Name**	**Start**	**End**	**SNP.id**	**SNP.pos**	**pval(headache)**	**pval(hypoPAN)**	**X^2,4df**	**FCP**

6	GeneID:10473	*HMGN4*	26538572	26547165	rs66941101	26530376	7.01E-10	3.83E-06	67.10208659	9.27744E-14
6	GeneID:80741	*LY6G5C*	31644461	31648150	rs114733011	31631531	8.54E-03	3.22E-13	67.05192598	9.50616E-14
6	GeneID:493812	*HCG11*	26521934	26527621	rs66941101	26530376	7.02E-10	4.15E-06	66.93923993	1.00407E-13
6	GeneID:100189308	*TRNAA29*	26553731	26553802	rs34246779	26549212	1.07E-09	2.77E-06	66.89614931	1.0253E-13
6	GeneID:100189223	*TRNAP13*	26555498	26555569	rs34246779	26549212	1.10E-09	2.84E-06	66.80093787	1.0738E-13
6	GeneID:100189110	*TRNAK14*	26556774	26556846	rs34246779	26549212	1.11E-09	2.86E-06	66.77554168	1.08712E-13
6	GeneID:100189153	*TRNAI9*	26554350	26554423	rs34246779	26549212	1.13E-09	2.91E-06	66.70367207	1.12572E-13
8	GeneID:83648	*FAM167A*	11278973	11324276	rs11775149	11335242	3.34E-04	1.02E-11	66.61575546	1.17481E-13
6	GeneID:100189369	*TRNAA37*	26682715	26682787	rs149123117	26670618	3.78E-10	9.50E-06	66.52260412	1.22915E-13
6	GeneID:352996	*P5-04*	29910314	29910426	rs2524005	29899677	3.23E-04	1.12E-11	66.50836557	1.23767E-13
6	GeneID:3105	*HLA-A*	29910247	29913661	rs2524005	29899677	3.23E-04	1.12E-11	66.50378866	1.24042E-13
6	GeneID:387322	*VN1R13P*	27028149	27029046	rs35608615	27040402	2.60E-09	1.48E-06	66.38930653	1.3113E-13
6	GeneID:353003	*HCG4P5*	29908689	29909578	rs2524005	29899677	3.36E-04	1.17E-11	66.34179656	1.34189E-13
6	GeneID:352965	*HLA-U*	29901541	29902657	rs2524005	29899677	3.42E-04	1.19E-11	66.27643922	1.38514E-13
6	GeneID:100126501	*TRNAA-AGC*	26796006	26796078	rs7454868	26799828	3.57E-10	1.21E-05	66.14675366	1.47513E-13
6	GeneID:352995	*P5.8*	29903015	29904157	rs2524005	29899677	3.59E-04	1.25E-11	66.08099057	1.52297E-13
6	GeneID:100189391	*TRNAM20*	26766444	26766516	rs13213200	26755915	2.73E-10	1.68E-05	66.02992674	1.56118E-13
6	GeneID:404024	*TRIM26P1*	30206078	30210056	rs191662147	30211196	2.79E-02	1.77E-13	65.8848972	1.67502E-13
6	GeneID:100419971	*LOC100419971*	25679007	25679651	rs13191296	25684606	4.57E-07	1.22E-08	65.64115328	1.88532E-13
6	GeneID:100189358	*TRNAA36*	26673590	26673662	rs149123117	26670618	4.92E-10	1.24E-05	65.4632486	2.05529E-13
6	GeneID:100189354	*TRNAM19*	26758550	26758622	rs13213200	26755915	3.16E-10	1.95E-05	65.44680247	2.07176E-13
10	GeneID:5654	*HTRA1*	124221041	124274424	rs2223089	124210160	2.31E-11	2.87E-04	65.29164109	2.23373E-13
6	GeneID:100507100	*LOC100507100*	26681354	26688069	rs149123117	26670618	5.23E-10	1.32E-05	65.21835492	2.31458E-13
10	GeneID:387715	*ARMS2*	124214179	124216868	rs78438709	124201071	3.62E-12	1.91E-03	65.2068932	2.32748E-13
6	GeneID:7932	*OR2H2*	29555683	29556745	rs115440940	29562188	7.16E-03	1.13E-12	64.89359730	2.7095E-13
8	GeneID:79660	*PPP1R3B*	8993764	9009152	rs330943	9021026	9.14E-08	8.97E-08	64.86832971	2.74291E-13
6	GeneID:692092	*SNORD32B*	29550029	29550105	rs115440940	29562188	7.32E-03	1.16E-12	64.8068531	2.82593E-13
6	GeneID:387043	*RPL13AP*	29550285	29550802	rs115440940	29562188	7.32E-03	1.16E-12	64.80657806	2.82631E-13
**Chr**	**Gene.id**	**Name**	**Start**	**End**	**SNP.id**	**SNP.pos**	**pval(headache)**	**pval(hypoPAN)**	**X^2,4df**	**FCP**

6	GeneID:100189156	*TRNAA20*	26771290	26771362	rs34244947	26761745	7.29E-10	1.27E-05	64.63383982	3.0733E-13
6	GeneID:100130756	*UQCRHP1*	31578860	31579133	rs2736188	31565648	1.07E-02	8.80E-13	64.58897804	3.1409E-13
8	GeneID:157285	*SGK223*	8175258	8239257	rs7005904	8218597	1.53E-03	6.30E-12	64.54656573	3.20617E-13
8	GeneID:100419209	*LOC100419209*	10332392	10356247	rs4841356	10334694	2.36E-06	4.67E-09	64.27553442	3.65652E-13
6	GeneID:3109	*HLA-DMB*	32902406	32908847	rs73396802	32915797	1.55E-03	8.26E-12	63.97763184	4.22474E-13
8	GeneID:4482	*MSRA*	9911830	10286401	rs7818437	10209623	1.78E-05	7.39E-10	63.92782745	4.328E-13
6	GeneID:353002	*HCG4P4*	29922982	29923410	rs116174003	29928557	1.83E-03	7.20E-12	63.91908354	4.34639E-13
6	GeneID:3138	*HLA-K*	29894436	29897616	rs2524005	29899677	3.34E-04	3.96E-11	63.91532117	4.35432E-13
6	GeneID:352966	*HLA-W*	29923611	29926835	rs116174003	29928557	1.84E-03	7.23E-12	63.90494364	4.37629E-13
6	GeneID:352994	*P5-05*	29894219	29894350	rs2524005	29899677	3.24E-04	4.11E-11	63.90161452	4.38336E-13
6	GeneID:80868	*HCG4B*	29892369	29893428	rs2524005	29899677	3.31E-04	4.20E-11	63.81052308	4.58127E-13
6	GeneID:353004	*HCG4P7*	29853887	29854784	rs114222883	29854585	7.54E-04	1.87E-11	63.78693636	4.63396E-13
6	GeneID:2550	*GABBR1*	29570005	29600962	rs115440940	29562188	1.08E-02	1.71E-12	63.23608924	6.05223E-13
4	GeneID:7098	*TLR3*	186990309	187006252	rs28811953	186980866	2.88E-02	6.45E-13	63.23234352	6.06323E-13
6	GeneID:352991	*P5-09*	29855513	29855662	rs114222883	29854585	7.46E-04	2.63E-11	63.12062935	6.40056E-13
6	GeneID:3136	*HLA-H*	29855383	29858856	rs114222883	29854585	7.46E-04	2.63E-11	63.12042121	6.40121E-13
17	GeneID:7473	*WNT3*	44841686	44896082	rs199533	44828931	8.09E-13	2.71E-02	62.90652739	7.10035E-13
12	GeneID:6231	*RPS26*	56435686	56438007	rs1702877	56427808	9.88E-05	3.10E-10	62.23434919	9.83377E-13
12	GeneID:488	*ATP2A2*	110719032	110788898	rs73206853	110708746	1.46E-10	2.36E-04	61.99254631	1.10558E-12
12	GeneID:8759	*ADAM1*	112336867	112339706	rs12296574	112322777	2.78E-02	1.31E-12	61.88947271	1.16217E-12
8	GeneID:83650	*SLC35G5*	11188495	11189695	rs7837036	11175489	1.33E-03	3.22E-11	61.56797334	1.35798E-12
12	GeneID:64375	*IKZF4*	56414689	56432219	rs1702877	56427808	1.18E-04	3.70E-10	61.52226919	1.38837E-12
6	GeneID:55604	*LRRC16A*	25279656	25620758	rs927985	25412811	2.27E-06	1.99E-08	61.45589985	1.43372E-12
12	GeneID:28981	*IFT81*	110562140	110656600	rs60013768	110576095	4.66E-10	1.04E-04	61.31034672	1.53841E-12
6	GeneID:80863	*PRRT1*	32116140	32119720	rs192471087	32119730	2.26E-03	2.27E-11	61.20139777	1.62174E-12
12	GeneID:6821	*SUOX*	56391043	56399309	rs1701704	56412487	1.79E-04	2.91E-10	61.17571769	1.64203E-12
8	GeneID:100505734	*LOC100505734*	11173368	11182938	rs7837036	11175489	1.62E-03	4.20E-11	60.63344364	2.13497E-12
12	GeneID:100131294	*LOC100131294*	56373755	56374851	rs773109	56374695	2.24E-04	3.20E-10	60.53157031	2.24288E-12
**Chr**	**Gene.id**	**Name**	**Start**	**End**	**SNP.id**	**SNP.pos**	**pval(headache)**	**pval(hypoPAN)**	**X^2,4df**	**FCP**

8	GeneID:100420053	*LOC100420053*	10194293	10195166	rs7818437	10209623	2.36E-06	3.47E-08	60.26503891	2.55169E-12
12	GeneID:400073	*C12orf76*	110478983	110505500	rs67974983	110519237	2.15E-09	3.85E-05	60.24382763	2.57801E-12
12	GeneID:2065	*ERBB3*	56473809	56497291	rs2292239	56482180	2.89E-04	3.23E-10	60.00077276	2.89978E-12
8	GeneID:83656	*C8orf12*	11225911	11296166	rs12156009	11285219	3.04E-03	3.22E-11	59.9111223	3.02833E-12
8	GeneID:66036	*MTMR9*	11142000	11185655	rs10098488	11130977	1.63E-03	6.40E-11	59.78201185	3.22354E-12
8	GeneID:613211	*DEFB134*	11851489	11853760	rs117672255	11867580	4.15E-03	2.85E-11	59.53070345	3.64027E-12
8	GeneID:645960	*LOC645960*	8819254	8819633	rs7821807	8805612	3.98E-06	3.13E-08	59.42943084	3.82305E-12
12	GeneID:5869	*RAB5B*	56367862	56388490	rs773109	56374695	3.21E-04	4.05E-10	59.33841287	3.99512E-12
1	GeneID:54507	*ADAMTSL4*	150521898	150533413	rs6693567	150510660	3.90E-11	4.09E-03	58.93062346	4.86613E-12
8	GeneID:286043	*MRPS18CP2*	8791228	8791520	rs878504	8783506	1.39E-06	1.23E-07	58.78818228	5.21312E-12
1	GeneID:100422997	*MIR4257*	150524405	150524490	rs6693567	150510660	3.05E-11	6.01E-03	58.65249522	5.56661E-12
12	GeneID:1017	*CDK2*	56360556	56366568	rs773109	56374695	2.73E-04	6.85E-10	58.61498702	5.66849E-12
4	GeneID:4299	*AFF1*	87856154	88062206	rs342458	88060025	3.17E-07	7.72E-07	58.07585682	7.35627E-12
8	GeneID:613209	*DEFB135*	11839830	11842099	rs79321779	11849367	2.41E-02	1.45E-11	57.36064676	1.03935E-11
8	GeneID:392193	*RPL19P13*	11113499	11114187	rs958648	11103895	7.03E-04	5.64E-10	57.11212823	1.17194E-11
12	GeneID:6490	*PMEL*	56347889	56360496	rs773109	56374695	3.26E-04	1.37E-09	56.86646843	1.3196E-11
12	GeneID:79600	*TCTN1*	111051832	111086935	rs146921795	111044793	6.38E-09	7.53E-05	56.72963724	1.40975E-11
17	GeneID:147040	*KCTD11*	7255208	7258263	rs3809831	7257669	4.70E-02	1.04E-11	56.69969923	1.43029E-11
12	GeneID:84329	*HVCN1*	111086491	111127617	rs7961345	111081598	8.12E-09	7.26E-05	56.31958215	1.71847E-11
8	GeneID:157739	*TDH*	11197146	11225961	rs17797894	11212081	1.91E-03	3.49E-10	56.07154967	1.9371E-11
11	GeneID:100507164	*LOC100507164*	35231590	35236568	rs76276019	35239888	5.07E-03	1.98E-10	55.25583974	2.87168E-11
12	GeneID:100653008	*LOC100653008*	56494149	56511615	rs2292239	56482180	2.54E-04	4.06E-09	55.20180808	2.94754E-11
8	GeneID:100616350	*MIR4660*	8905955	8906028	rs2953805	8914757	2.63E-05	3.96E-08	55.18242076	2.97524E-11
12	GeneID:144712	*HMGA1P3*	110866403	110868198	rs147356273	110876462	2.81E-09	3.82E-04	55.11658635	3.07127E-11
12	GeneID:160760	*PPTC7*	110972237	111021064	rs148331092	111018167	4.98E-09	3.82E-04	53.97814936	5.31842E-11
12	GeneID:51434	*ANAPC7*	110810705	110841535	rs140441196	110802707	5.02E-09	3.83E-04	53.95439415	5.37968E-11
12	GeneID:100129882	*RPL31P49*	110898765	110899206	rs148167533	110909778	3.67E-09	5.43E-04	53.88513693	5.56234E-11
2	GeneID:6772	*STAT1*	191833762	191878976	rs148933924	191827528	1.47E-02	1.59E-10	53.56983404	6.47541E-11
**Chr**	**Gene.id**	**Name**	**Start**	**End**	**SNP.id**	**SNP.pos**	**pval(headache)**	**pval(hypoPAN)**	**X^2,4df**	**FCP**

18	GeneID:5771	*PTPN2*	12785477	12884334	rs72872144	12889046	1.05E-02	2.53E-10	53.30441806	7.35906E-11
12	GeneID:51184	*GPN3*	110890291	110906526	rs147356273	110876462	3.39E-09	7.90E-04	53.29238537	7.40186E-11
12	GeneID:10094	*ARPC3*	110872706	110888158	rs147356273	110876462	3.18E-09	1.21E-03	52.56558828	1.05055E-10
12	GeneID:29902	*C12orf24*	110906232	110928192	rs118053970	110932820	4.73E-09	8.67E-04	52.44102172	1.11551E-10
12	GeneID:144715	*RAD9B*	110940005	110969891	rs118053970	110932820	5.04E-09	8.66E-04	52.31383092	1.18598E-10
8	GeneID:1508	*CTSB*	11700033	11725646	rs1293298	11712443	4.96E-04	9.07E-09	52.2541455	1.22056E-10
13	GeneID:432369	*ATP5EP2*	28519343	28519710	rs9579134	28508988	4.55E-03	1.00E-09	52.22572217	1.23738E-10
6	GeneID:100188987	*TRNAH3*	27125906	27125977	rs67540232	27140866	4.78E-10	1.00E-02	52.12760504	1.29725E-10
8	GeneID:100421446	*LOC100421446*	11856339	11857014	rs117672255	11867580	3.92E-03	1.40E-09	51.85251006	1.48097E-10
1	GeneID:23248	*RPRD2*	150336990	150449042	rs698915	150388318	2.45E-10	2.27E-02	51.82515889	1.5006E-10
3	GeneID:1237	*CCR8*	39371197	39375171	rs77200706	39384057	4.39E-03	1.34E-09	51.70906078	1.58686E-10
6	GeneID:100422934	*MIR3143*	27115405	27115467	rs61240102	27124904	6.22E-10	9.52E-03	51.7041345	1.59062E-10
6	GeneID:85235	*HIST1H2AH*	27114908	27115346	rs61240102	27124904	6.22E-10	9.52E-03	51.70394676	1.59077E-10
12	GeneID:51699	*VPS29*	110929330	110939916	rs118053970	110932820	3.96E-09	1.50E-03	51.69282895	1.5993E-10
6	GeneID:100189196	*TRNAV20*	27118022	27118094	rs61240102	27124904	6.31E-10	9.66E-03	51.64553072	1.63613E-10
8	GeneID:286046	*XKR6*	10753654	11058875	rs17722940	10898459	1.23E-03	5.10E-09	51.58508265	1.68444E-10
6	GeneID:8294	*HIST1H4I*	27107088	27107457	rs67330695	27103654	6.42E-10	1.06E-02	51.42840431	1.81638E-10
8	GeneID:90459	*ERI1*	8860314	8890849	rs2956244	8885166	1.68E-04	5.39E-08	50.85163961	2.39737E-10
8	GeneID:402333	*OR7E160P*	11855115	11892149	rs117672255	11867580	5.35E-03	2.01E-09	50.51120892	2.82392E-10
8	GeneID:2222	*FDFT1*	11660190	11696818	rs1293296	11711157	1.06E-03	1.32E-08	49.98988391	3.62847E-10
8	GeneID:2626	*GATA4*	11561717	11617509	rs2409814	11606152	7.72E-03	1.91E-09	49.88020152	3.82493E-10
4	GeneID:645481	*LOC645481*	26113487	26115450	rs7673206	26126838	3.78E-02	4.16E-10	49.74971745	4.07253E-10
13	GeneID:100132234	*LOC100132234*	28527669	28529500	rs9512935	28515327	2.01E-02	8.51E-10	49.58082604	4.41691E-10
6	GeneID:352993	*HCP5P6*	29883530	29885892	rs2524005	29899677	2.56E-04	7.56E-08	49.33333936	4.97475E-10
12	GeneID:5501	*PPP1CC*	111157613	111180783	rs73194035	111165793	4.46E-08	4.73E-04	49.16232304	5.40079E-10
11	GeneID:3992	*FADS1*	61567097	61584529	rs61897793	61599347	4.36E-04	6.36E-08	48.61569246	7.0225E-10
3	GeneID:645715	*EEF1A1P24*	39400036	39401410	rs73826410	39403292	1.81E-03	2.08E-08	48.00665262	9.40775E-10
6	GeneID:353014	*HCG4P9*	29766201	29767903	rs143525179	29751998	7.75E-06	5.48E-06	47.76523252	1.05635E-09
**Chr**	**Gene.id**	**Name**	**Start**	**End**	**SNP.id**	**SNP.pos**	**pval(headache)**	**pval(hypoPAN)**	**X^2,4df**	**FCP**

8	GeneID:100271154	*RPL17P29*	11032519	11033125	rs4840551	11029039	2.01E-03	2.16E-08	47.72633318	1.07625E-09
11	GeneID:100302263	*MIR1908*	61582633	61582712	rs968567	61595564	1.03E-03	4.28E-08	47.68495973	1.09784E-09
12	GeneID:642493	*RPL29P25*	111279361	111279808	rs71458365	111274102	1.53E-05	3.22E-06	47.46546491	1.21977E-09
6	GeneID:221547	*RANP1*	30453662	30454724	rs115485852	30469193	3.58E-02	1.42E-09	47.40291004	1.25693E-09
19	GeneID:100271529	*RPL39P38*	18395976	18396355	rs75374192	18385125	4.95E-02	1.07E-09	47.31614938	1.31035E-09
11	GeneID:9415	*FADS2*	61595713	61634825	rs61897793	61599347	9.20E-04	6.08E-08	47.21476344	1.37566E-09
19	GeneID:100422833	*MIR3188*	18392887	18392971	rs75374192	18385125	4.90E-02	1.21E-09	47.09351099	1.45804E-09
4	GeneID:4790	*NFKB1*	103422486	103538459	rs3774968	103531112	2.47E-02	2.43E-09	47.07446687	1.47141E-09
6	GeneID:353008	*3.8-1.3*	29878012	29879177	rs1632908	29893403	6.35E-04	1.08E-07	46.81407262	1.66713E-09
6	GeneID:3133	*HLA-E*	30457183	30461982	rs115485852	30469193	4.25E-02	1.69E-09	46.71288325	1.75001E-09
6	GeneID:100421582	*PAIP1P1*	30154575	30156383	rs188837807	30155864	1.41E-03	5.11E-08	46.71164502	1.75105E-09
6	GeneID:7726	*TRIM26*	30152232	30181271	rs188837807	30155864	1.82E-03	4.38E-08	46.50423624	1.93412E-09
6	GeneID:554223	*LOC554223*	29759683	29765584	rs143525179	29751998	1.07E-05	7.55E-06	46.48051725	1.95624E-09
19	GeneID:55527	*FEM1A*	4791728	4795571	rs115654559	4797674	3.16E-02	2.73E-09	46.34464358	2.08789E-09
1	GeneID:80222	*TARS2*	150459920	150479756	rs7549396	150464603	3.32E-09	2.72E-02	46.25491416	2.17964E-09
18	GeneID:10666	*CD226*	67530192	67624232	rs74523488	67532869	3.94E-03	2.44E-08	46.12897386	2.31525E-09
3	GeneID:344697	*HNRNPA1P21*	39376477	39377368	rs77200706	39384057	3.86E-03	2.95E-08	45.79492858	2.71712E-09
12	GeneID:5036	*PA2G4*	56498103	56507694	rs798829	56485269	3.46E-04	3.62E-07	45.60057896	2.98224E-09
6	GeneID:353016	*HCP5P13*	29760911	29762023	rs143525179	29751998	1.01E-05	1.43E-05	45.31607991	3.41757E-09
11	GeneID:693196	*MIR611*	61559967	61560033	rs61896141	61556039	1.73E-03	9.04E-08	45.15789827	3.68648E-09
6	GeneID:352962	*HLA-V*	29759530	29760527	rs143525179	29751998	1.07E-05	1.51E-05	45.09707494	3.79541E-09
6	GeneID:54435	*HCG4*	29758808	29760850	rs143525179	29751998	1.07E-05	1.51E-05	45.09375306	3.80145E-09
10	GeneID:118663	*BTBD16*	124030821	124097676	rs56163147	124109797	2.36E-07	7.13E-04	45.01208324	3.95303E-09
11	GeneID:2237	*FEN1*	61560109	61564716	rs61896141	61556039	1.80E-03	9.42E-08	44.99383205	3.98772E-09
12	GeneID:84260	*TCHP*	110338079	110355874	rs116940627	110336546	4.59E-06	3.76E-05	44.96176584	4.0494E-09
11	GeneID:745	*C11orf9*	61520121	61555989	rs61896068	61513400	8.45E-04	2.06E-07	44.94235257	4.08721E-09
11	GeneID:746	*C11orf10*	61556602	61560085	rs61896141	61556039	1.90E-03	9.93E-08	44.78297489	4.41122E-09
13	GeneID:3651	*PDX1*	28494168	28500451	rs7982864	28500496	3.70E-03	5.51E-08	44.62652067	4.7542E-09
**Chr**	**Gene.id**	**Name**	**Start**	**End**	**SNP.id**	**SNP.pos**	**pval(headache)**	**pval(hypoPAN)**	**X^2,4df**	**FCP**

6	GeneID:100131814	*LINC00271*	135818939	136011976	rs9376121	135965357	3.87E-02	5.56E-09	44.51774415	5.00822E-09
8	GeneID:693182	*MIR597*	9599182	9599278	rs35169606	9604066	9.97E-04	2.26E-07	44.42402964	5.2379E-09
9	GeneID:7464	*CORO2A*	100883257	100954956	rs12551475	100923751	2.10E-03	1.10E-07	44.36738103	5.38181E-09
16	GeneID:9114	*ATP6V0D1*	67471917	67515089	rs72790338	67504682	1.23E-02	1.91E-08	44.34050817	5.45145E-09
6	GeneID:100422429	*LOC100422429*	30929178	30929755	rs143764990	30943138	2.56E-02	1.02E-08	44.13050235	6.02756E-09
6	GeneID:100420530	*LOC100420530*	30931992	30933937	rs143764990	30943138	2.56E-02	1.02E-08	44.1215765	6.05335E-09
16	GeneID:29100	*TMEM208*	67261016	67263182	rs55691975	67250992	2.30E-02	1.37E-08	43.75240882	7.22219E-09
4	GeneID:260422	*LOC260422*	87834081	87836070	rs2705620	87835616	6.05E-04	5.61E-07	43.60705727	7.74194E-09
3	GeneID:56983	*POGLUT1*	119187785	119213555	rs73187899	119228515	3.87E-02	8.77E-09	43.60674032	7.74311E-09
16	GeneID:181	*AGRP*	67516474	67517716	rs72790338	67504682	6.88E-03	5.92E-08	43.24266789	9.21493E-09
8	GeneID:100128174	*LOC100128174*	11901012	11907134	rs117378333	11892737	9.98E-03	4.09E-08	43.24194847	9.2181E-09
8	GeneID:693183	*MIR598*	10892716	10892812	rs17722940	10898459	5.23E-04	7.84E-07	43.23067984	9.26788E-09
6	GeneID:135656	*DPCR1*	30908777	30921998	rs138678612	30932223	4.97E-02	9.21E-09	43.00880143	1.03045E-08
3	GeneID:100302112	*MIR1284*	71591121	71591240	rs7610856	71579022	2.84E-07	1.64E-03	42.97549286	1.04697E-08
12	GeneID:6171	*RPL41*	56510374	56511616	rs4759228	56508409	5.38E-04	8.80E-07	42.94134944	1.06419E-08
4	GeneID:643974	*RPL6P13*	87791318	87792586	rs11722905	87791982	9.71E-04	4.99E-07	42.89715898	1.0869E-08
3	GeneID:51300	*TIMMDC1*	119217368	119243128	rs73187899	119228515	4.69E-02	1.06E-08	42.84102071	1.11644E-08
6	GeneID:100048904	*DDX39BP1*	29874324	29875226	rs114298287	29865311	7.12E-04	7.16E-07	42.79487695	1.14132E-08
11	GeneID:100302280	*MIR1237*	64136074	64136175	rs138662412	64125119	1.56E-02	3.30E-08	42.76857416	1.15575E-08
6	GeneID:6824	*ETF1P1*	29999490	30001654	rs114315683	30012624	3.35E-04	1.59E-06	42.70040717	1.194E-08
6	GeneID:352964	*HLA-T*	29864220	29866724	rs114222883	29854585	7.28E-04	7.68E-07	42.60831551	1.24769E-08
6	GeneID:285830	*HLA-F-AS1*	29694378	29716826	rs183103224	29690291	4.70E-03	1.21E-07	42.56846819	1.27166E-08
6	GeneID:4280	*MICE*	29709234	29716880	rs62391801	29694443	6.49E-03	9.30E-08	42.45863094	1.34014E-08
7	GeneID:79783	*C7orf10*	40174575	40900366	rs12532479	40427634	3.28E-08	1.86E-02	42.43247172	1.35699E-08
16	GeneID:29800	*ZDHHC1*	67428322	67450339	rs189527310	67447829	4.50E-02	1.37E-08	42.41691414	1.36711E-08
4	GeneID:57563	*KLHL8*	88082214	88141674	rs1466364	88068599	3.16E-07	1.98E-03	42.38476721	1.38825E-08
6	GeneID:100419974	*HMGB1P17*	135957248	135957851	rs9376121	135965357	1.38E-02	4.69E-08	42.32143776	1.43087E-08
12	GeneID:84872	*ZC3H10*	56512030	56516278	rs4759228	56508409	6.44E-04	1.05E-06	42.22483641	1.49841E-08
**Chr**	**Gene.id**	**Name**	**Start**	**End**	**SNP.id**	**SNP.pos**	**pval(headache)**	**pval(hypoPAN)**	**X^2,4df**	**FCP**

1	GeneID:9047	*SH2D2A*	156776035	156786640	rs2234883	156787110	2.25E-03	3.02E-07	42.21685141	1.50413E-08
16	GeneID:26231	*LRRC29*	67241042	67260901	rs55691975	67250992	3.64E-02	2.18E-08	41.90734556	1.74358E-08
11	GeneID:283234	*CCDC88B*	64107690	64125006	rs138662412	64125119	2.92E-02	2.81E-08	41.84419676	1.79693E-08
6	GeneID:3008	*HIST1H1E*	26156559	26157343	rs7749823	26158079	1.47E-04	5.95E-06	41.71767399	1.90875E-08
16	GeneID:29109	*FHOD1*	67263292	67281425	rs55691975	67250992	3.90E-02	2.33E-08	41.64083607	1.98002E-08
1	GeneID:1893	*ECM1*	150480487	150486265	rs12031973	150485566	3.61E-08	2.71E-02	41.49330066	2.1244E-08
6	GeneID:100507399	*HCG8*	29978606	29980440	rs141548494	29983301	4.59E-04	2.29E-06	41.35178177	2.27275E-08
12	GeneID:88455	*ANKRD13A*	110437235	110477237	rs12312565	110449173	2.87E-05	3.68E-05	41.33536959	2.29061E-08
11	GeneID:8986	*RPS6KA4*	64126625	64139687	rs138662412	64125119	2.81E-02	3.78E-08	41.3284034	2.29823E-08
4	GeneID:132989	*C4orf36*	87797358	87813575	rs11722905	87791982	1.57E-03	6.88E-07	41.29397227	2.33628E-08
16	GeneID:51673	*TPPP3*	67423712	67427421	rs143082214	67442216	4.74E-02	2.80E-08	40.87818611	2.84853E-08
11	GeneID:100129473	*LOC100129473*	61534400	61534987	rs10792318	61531443	2.25E-03	6.40E-07	40.72032288	3.07113E-08
12	GeneID:51228	*GLTP*	110288748	110318293	rs73202468	110295897	2.38E-05	7.41E-05	40.31211218	3.73053E-08
6	GeneID:11074	*TRIM31*	30070674	30080867	rs114071505	30060631	7.34E-03	2.80E-07	40.00716423	4.31368E-08
8	GeneID:8658	*TNKS*	9413445	9639856	rs35169606	9604066	6.22E-03	3.31E-07	40.00253939	4.32319E-08
1	GeneID:4914	*NTRK1*	156785542	156851642	rs2234883	156787110	3.47E-03	6.71E-07	39.75579135	4.86213E-08
1	GeneID:5546	*PRCC*	156737274	156770609	rs2735657	156780306	6.02E-03	4.08E-07	39.6498692	5.11357E-08
6	GeneID:10255	*HCG9*	29942892	29946180	rs116174003	29928557	1.71E-03	1.45E-06	39.63848411	5.14135E-08
6	GeneID:352967	*MICG*	29780167	29780469	rs114181339	29784983	8.68E-04	3.22E-06	39.39220395	5.7807E-08
6	GeneID:4279	*MICD*	29938149	29943522	rs116174003	29928557	1.83E-03	1.56E-06	39.34948954	5.89939E-08
6	GeneID:80862	*ZNRD1-AS1*	29968788	30028961	rs141548494	29983301	8.58E-04	3.48E-06	39.25840593	6.16067E-08
16	GeneID:146206	*RLTPR*	67679030	67691472	rs73597575	67677001	1.49E-02	2.06E-07	39.20925663	6.30642E-08
4	GeneID:442777	*LOC442777*	87870690	87871257	rs2705616	87862396	1.66E-02	1.87E-07	39.17384128	6.41357E-08
16	GeneID:6236	*RRAD*	66955582	66959439	rs3848284	66947383	1.03E-02	3.10E-07	39.11783844	6.58673E-08
6	GeneID:2794	*GNL1*	30509154	30525371	rs116099574	30517414	3.52E-02	9.52E-08	39.02664944	6.87871E-08
1	GeneID:2029	*ENSA*	150594599	150602098	rs138520356	150586501	5.90E-06	5.70E-04	39.02109893	6.89689E-08
22	GeneID:5594	*MAPK1*	22113946	22221970	rs3859841	22232671	6.41E-07	5.68E-03	38.86159768	7.44041E-08
6	GeneID:346171	*ZFP57*	29640169	29644931	rs138358844	29659400	7.55E-03	4.85E-07	38.84894993	7.48529E-08
**Chr**	**Gene.id**	**Name**	**Start**	**End**	**SNP.id**	**SNP.pos**	**pval(headache)**	**pval(hypoPAN)**	**X^2,4df**	**FCP**

Chr	Gene.id	Name	Start	End	SNP.id	SNP.pos	pval(headache)	pval(hypoPAN)	X^2,4df	FCP
6	GeneID:100126314	*MIR877*	30552109	30552194	rs151218088	30540762	1.45E-02	2.63E-07	38.77532851	7.75197E-08
6	GeneID:5758	*PTMAP1*	30601227	30603024	rs114148005	30587406	1.97E-02	1.93E-07	38.76847355	7.77727E-08
6	GeneID:442191	*OR14J1*	29274467	29275432	rs150254595	29278406	6.51E-04	5.90E-06	38.75376263	7.83186E-08
2	GeneID:3899	*AFF3*	100163715	100759037	rs4851239	100489966	3.39E-05	1.16E-04	38.71810589	7.96577E-08
1	GeneID:84072	*HORMAD1*	150670535	150693364	rs17600020	150671887	4.08E-05	9.61E-05	38.71128665	7.99164E-08
1	GeneID:11313	*LYPLA2*	24117646	24122029	rs12041159	24127660	1.61E-04	2.50E-05	38.66311844	8.17676E-08
6	GeneID:28973	*MRPS18B*	30585486	30594174	rs114148005	30587406	2.07E-02	2.03E-07	38.57141195	8.54111E-08
6	GeneID:353013	*RPL7AP7*	29770910	29771797	rs114181339	29784983	9.28E-04	4.69E-06	38.50664474	8.80815E-08
16	GeneID:51647	*FAM96B*	66965958	66968320	rs185206552	66952572	1.75E-02	2.63E-07	38.39043538	9.30832E-08
1	GeneID:2582	*GALE*	24122089	24127294	rs12041159	24127660	1.74E-04	2.67E-05	38.37897994	9.35914E-08
6	GeneID:79969	*ATAT1*	30594613	30614598	rs114148005	30587406	2.24E-02	2.19E-07	38.26801667	9.86588E-08
6	GeneID:352963	*HLA-P*	29767821	29770856	rs114181339	29784983	9.88E-04	4.99E-06	38.25601525	9.92231E-08
6	GeneID:3135	*HLA-G*	29794756	29798899	rs114853934	29812896	7.26E-04	7.08E-06	38.17105887	1.0331E-07
6	GeneID:353005	*HCG4P8*	29793906	29794891	rs114181339	29784983	8.33E-04	6.18E-06	38.16874312	1.03424E-07
12	GeneID:23344	*ESYT1*	56521986	56538460	rs4759228	56508409	9.15E-04	5.64E-06	38.16423252	1.03646E-07
16	GeneID:1014	*CDH16*	66942025	66952887	rs3848284	66947383	1.32E-02	3.95E-07	38.1486107	1.04418E-07
6	GeneID:353000	*HCGVIII-2*	29801426	29802705	rs114853934	29812896	7.38E-04	7.12E-06	38.12926066	1.05382E-07
3	GeneID:55254	*TMEM39A*	119149701	119182471	rs17203118	119152795	4.27E-02	1.25E-07	38.09263869	1.07232E-07
6	GeneID:5514	*PPP1R10*	30568182	30585020	rs114148005	30587406	2.27E-02	2.52E-07	37.95964646	1.14224E-07
12	GeneID:9815	*GIT2*	110367607	110434194	rs12312565	110449173	3.41E-05	1.70E-04	37.93398991	1.15625E-07
1	GeneID:100509213	*LOC100509213*	24114238	24120044	rs12041159	24127660	2.00E-04	2.90E-05	37.93009955	1.15839E-07
4	GeneID:100616463	*MIR4452*	87463635	87463705	rs56164000	87458247	1.70E-03	3.50E-06	37.87811504	1.18734E-07
6	GeneID:100189348	*TRNAI21*	28505367	28505460	rs115709379	28518299	7.76E-03	7.74E-07	37.86153401	1.19673E-07
6	GeneID:352989	*P5-11*	29795606	29795751	rs114181339	29784983	9.10E-04	6.75E-06	37.81580177	1.22301E-07
6	GeneID:100189034	*TRNAR7*	28510891	28510963	rs115709379	28518299	7.85E-03	7.83E-07	37.81552177	1.22317E-07
2	GeneID:2744	*GLS*	191745547	191829776	rs148933924	191827528	1.30E-02	4.94E-07	37.72194961	1.27874E-07
16	GeneID:10664	*CTCF*	67596310	67673088	rs73597575	67677001	2.65E-02	2.57E-07	37.60979205	1.34868E-07
4	GeneID:100506746	*LOC100506746*	87846046	87856002	rs2705620	87835616	5.77E-04	1.19E-05	37.5929706	1.3595E-07

1	GeneID:1513	*CTSK*	150768684	150780917	rs150253674	150788065	1.49E-05	4.67E-04	37.56119689	1.38016E-07
6	GeneID:100048907	*ZDHHC20P1*	29675918	29676324	rs183103224	29690291	2.97E-03	2.37E-06	37.54559414	1.39042E-07
12	GeneID:59341	*TRPV4*	110220892	110271212	rs76219614	110272635	1.17E-04	6.02E-05	37.54512053	1.39073E-07
2	GeneID:87178	*PNPT1*	55861198	55921011	rs62165233	55911454	2.85E-03	2.60E-06	37.43420214	1.46592E-07
8	GeneID:100420404	*LOC100420404*	9311804	9314336	rs7824444	9317065	1.06E-03	7.19E-06	37.38676285	1.4993E-07
16	GeneID:23491	*CES3*	66995132	67009052	rs71649613	67006541	4.94E-02	1.58E-07	37.34260454	1.53105E-07
1	GeneID:3155	*HMGCL*	24128367	24151949	rs12041159	24127660	2.38E-04	3.53E-05	37.1875494	1.64795E-07
16	GeneID:124460	*SNX20*	50700211	50715264	rs35581802	50710646	2.35E-02	3.64E-07	37.15545122	1.67324E-07
6	GeneID:80742	*PRR3*	30524486	30532473	rs151218088	30540762	1.53E-02	5.65E-07	37.1312748	1.69254E-07
1	GeneID:57095	*PITHD1*	24104876	24114722	rs12041159	24127660	2.50E-04	3.64E-05	37.02818856	1.77738E-07
6	GeneID:23	*ABCF1*	30539170	30559309	rs151218088	30540762	2.25E-02	4.08E-07	37.01497699	1.78855E-07
6	GeneID:100189128	*TRNAA18*	26687485	26687557	rs9467835	26678512	7.11E-06	1.35E-03	36.93136116	1.86091E-07
2	GeneID:100132762	*LOC100132762*	162424958	162426866	rs140568161	162413257	1.69E-03	5.69E-06	36.92055928	1.87047E-07
6	GeneID:353020	*HCG4P11*	29688955	29689944	rs183103224	29690291	3.60E-03	2.97E-06	36.70535939	2.07145E-07
6	GeneID:6148	*RPL23AP1*	29694409	29694931	rs183103224	29690291	3.65E-03	3.01E-06	36.65561463	2.12088E-07
6	GeneID:3134	*HLA-F*	29691117	29695073	rs183103224	29690291	3.65E-03	3.01E-06	36.6524545	2.12406E-07
22	GeneID:100286925	*LOC100286925*	22292609	22297806	rs5995257	22285987	8.76E-07	1.37E-02	36.47475946	2.31075E-07
17	GeneID:23533	*PIK3R5*	8782233	8869024	rs61759573	8813578	3.49E-03	4.06E-06	36.14314858	2.70397E-07
2	GeneID:9855	*FARP2*	242295664	242434256	rs886815	242396426	4.67E-02	3.84E-07	35.6755902	3.37423E-07
6	GeneID:100189303	*TRNAM16*	26701712	26701784	rs2451729	26705859	5.23E-06	3.43E-03	35.6695956	3.38382E-07
1	GeneID:55204	*GOLPH3L*	150618701	150669672	rs79726307	150622075	4.06E-05	4.44E-04	35.66143633	3.39691E-07
22	GeneID:9647	*PPM1F*	22273792	22307250	rs5995257	22285987	1.49E-06	1.23E-02	35.62654756	3.45349E-07
1	GeneID:3645	*INSRR*	156810665	156828712	rs6689750	156798725	5.67E-03	3.66E-06	35.37939643	3.88208E-07
12	GeneID:100616454	*MIR4497*	110271153	110271241	rs76219614	110272635	6.01E-05	3.46E-04	35.37507929	3.89001E-07
8	GeneID:94137	*RP1L1*	10463859	10512617	rs4395858	10521021	6.92E-04	3.37E-05	35.14797511	4.33131E-07
3	GeneID:100506815	*FOXP1-IT1*	71619425	71623608	rs62244889	71609007	1.04E-05	2.35E-03	35.06022403	4.51489E-07
12	GeneID:246329	*STAC3*	57637242	57644969	rs116973701	57629608	2.38E-06	1.06E-02	34.99236106	4.66216E-07
5	GeneID:134505	*LOC134505*	102368017	102368765	rs30718	102354355	2.95E-03	9.20E-06	34.84419968	5.00054E-07
**Chr**	**Gene.id**	**Name**	**Start**	**End**	**SNP.id**	**SNP.pos**	**pval(headache)**	**pval(hypoPAN)**	**X^2,4df**	**FCP**

9	GeneID:3440	*IFNA2*	21384254	21385396	rs594544	21372123	2.76E-03	9.84E-06	34.8396974	5.0112E-07
12	GeneID:6601	*SMARCC2*	56555636	56583351	rs150823486	56570811	1.55E-03	1.76E-05	34.83879583	5.01334E-07
4	GeneID:5783	*PTPN13*	87515468	87736329	rs72665770	87513053	1.57E-03	1.87E-05	34.6962536	5.36285E-07
4	GeneID:345274	*SLC10A6*	87744621	87770416	rs17751003	87771621	1.70E-02	1.73E-06	34.68963188	5.37967E-07
6	GeneID:100189033	*TRNAA5*	28697092	28697163	rs148076211	28708629	2.35E-03	1.26E-05	34.67245114	5.42354E-07
6	GeneID:100189178	*TRNAV18*	28703206	28703277	rs148076211	28708629	2.36E-03	1.26E-05	34.66476633	5.44328E-07
6	GeneID:442181	*RPSAP2*	28699714	28700751	rs148076211	28708629	2.36E-03	1.26E-05	34.65572912	5.46658E-07
1	GeneID:405	*ARNT*	150782181	150849244	rs150253674	150788065	3.59E-05	8.44E-04	34.62353622	5.55041E-07
6	GeneID:100189433	*TRNAT21*	28693795	28693868	rs148076211	28708629	2.50E-03	1.34E-05	34.42578773	6.09416E-07
6	GeneID:100189472	*TRNAF15P*	28694855	28694927	rs148076211	28708629	2.50E-03	1.34E-05	34.42578773	6.09416E-07
16	GeneID:442901	*MIR328*	67236224	67236298	rs55691975	67250992	3.01E-02	1.32E-06	34.07804148	7.18225E-07
2	GeneID:645834	*KRT8P15*	203704809	203706580	rs72932780	203695826	8.01E-05	5.04E-04	34.05205884	7.27092E-07
6	GeneID:100189411	*TRNAA41*	26705606	26705678	rs2451729	26705859	4.93E-06	8.18E-03	34.05006784	7.27776E-07
6	GeneID:80352	*RNF39*	30038043	30043628	rs115985474	30055179	7.79E-03	5.21E-06	34.04119533	7.30832E-07
9	GeneID:3461	*IFNA11P*	21398613	21399138	rs649053	21387418	4.45E-03	9.22E-06	34.02080153	7.37905E-07
6	GeneID:100189092	*TRNAA12*	28687481	28687552	rs114950784	28696844	3.48E-03	1.24E-05	33.92485996	7.72103E-07
1	GeneID:2517	*FUCA1*	24171567	24194859	rs61778163	24202985	4.44E-03	9.87E-06	33.88584395	7.86458E-07
18	GeneID:220164	*DOK6*	67068284	67516323	rs34143434	67336322	3.17E-02	1.44E-06	33.80980745	8.15203E-07
12	GeneID:6472	*SHMT2*	57623356	57628718	rs116973701	57629608	2.70E-06	1.74E-02	33.75048354	8.38354E-07
12	GeneID:56901	*NDUFA4L2*	57628686	57634545	rs116973701	57629608	2.70E-06	1.74E-02	33.7491493	8.38882E-07
8	GeneID:203074	*PRSS55*	10383056	10411676	rs4841381	10418221	3.32E-03	1.45E-05	33.69712781	8.59736E-07
16	GeneID:79767	*ELMO3*	67233028	67237927	rs55691975	67250992	3.31E-02	1.46E-06	33.6948098	8.60677E-07
6	GeneID:4340	*MOG*	29624758	29640149	rs62392957	29649219	1.02E-02	4.95E-06	33.60108185	8.99603E-07
9	GeneID:3447	*IFNA13*	21367371	21368075	rs594544	21372123	3.41E-03	1.58E-05	33.4773289	9.53699E-07
16	GeneID:653319	*KIAA0895L*	67209505	67217883	rs77960079	67232523	3.85E-02	1.54E-06	33.28012469	1.04668E-06
8	GeneID:606553	*C8orf49*	11618765	11620309	rs2409814	11606152	9.33E-03	6.36E-06	33.27919245	1.04714E-06
9	GeneID:56262	*LRRC8A*	131644391	131680318	rs188368517	131638853	8.45E-03	7.06E-06	33.26823071	1.05257E-06
22	GeneID:7008	*TEF*	41763392	41795330	rs11090046	41769083	4.59E-02	1.32E-06	33.24229813	1.06552E-06
**Chr**	**Gene.id**	**Name**	**Start**	**End**	**SNP.id**	**SNP.pos**	**pval(headache)**	**pval(hypoPAN)**	**X^2,4df**	**FCP**

1	GeneID:6924	*TCEB3*	24069856	24088549	rs2076346	24083649	2.95E-03	2.09E-05	33.20324793	1.08532E-06
9	GeneID:883	*CCBL1*	131595392	131644354	rs188368517	131638853	9.46E-03	6.70E-06	33.14693952	1.11453E-06
17	GeneID:339168	*TMEM95*	7258497	7260538	rs3809831	7257669	4.70E-02	1.37E-06	33.11025323	1.13398E-06
5	GeneID:5066	*PAM*	102201527	102366809	rs30718	102354355	8.94E-03	7.25E-06	33.10323096	1.13774E-06
16	GeneID:1874	*E2F4*	67226068	67232821	rs77960079	67232523	4.03E-02	1.61E-06	33.102312	1.13823E-06
16	GeneID:283849	*EXOC3L1*	67218282	67224107	rs77960079	67232523	4.09E-02	1.63E-06	33.04488747	1.16947E-06
11	GeneID:441601	*LOC441601*	50238999	50257633	rs72907375	50256111	8.45E-06	7.97E-03	33.0269068	1.17942E-06
12	GeneID:341333	*LOC341333*	68429547	68430554	rs117555625	68423869	1.47E-02	4.64E-06	33.00307319	1.19275E-06
8	GeneID:100422982	*MIR4286*	10524488	10524580	rs4395858	10521021	1.88E-04	4.02E-04	32.79437637	1.31604E-06
8	GeneID:728938	*SUB1P1*	11647461	11648594	rs2740444	11648789	3.69E-03	2.05E-05	32.7940873	1.31622E-06
2	GeneID:55759	*WDR12*	203745323	203776949	rs35212307	203765756	6.01E-05	1.29E-03	32.74902333	1.34447E-06
11	GeneID:4607	*MYBPC3*	47352957	47374253	rs79187134	47346465	1.45E-03	5.78E-05	32.59126313	1.44822E-06
12	GeneID:4637	*MYL6*	56552045	56555366	rs7960225	56564811	1.24E-02	6.90E-06	32.5546313	1.47342E-06
8	GeneID:5747	*PTK2*	141668481	142011412	rs4961285	141700074	3.99E-02	2.22E-06	32.48138194	1.52515E-06
3	GeneID:27086	*FOXP1*	71004736	71633140	rs7610856	71579022	5.40E-06	1.80E-02	32.29490371	1.66514E-06
16	GeneID:1506	*CTRL*	67963473	67965778	rs148240639	67954932	4.86E-02	2.08E-06	32.21601692	1.72815E-06
16	GeneID:80262	*C16orf70*	67143915	67182442	rs191091369	67139616	3.20E-02	3.22E-06	32.18003584	1.75767E-06
1	GeneID:100270904	*RPS3AP11*	42957386	42958184	rs11805616	42966550	1.82E-05	5.68E-03	32.16596988	1.76934E-06
4	GeneID:133308	*SLC9B2*	103946652	103998170	rs7688014	103975060	4.98E-06	2.15E-02	32.10210523	1.82334E-06
6	GeneID:6992	*PPP1R11*	30034932	30038110	rs189720814	30049788	4.50E-02	2.39E-06	32.09528133	1.82921E-06
6	GeneID:26529	*OR12D2*	29364416	29365448	rs3128852	29364135	4.93E-04	2.22E-04	32.05055816	1.86812E-06
6	GeneID:729177	*LOC729177*	22134831	22147422	rs9295536	22131929	2.69E-05	4.09E-03	32.0466838	1.87153E-06
6	GeneID:100131607	*LOC100131607*	34664094	34665247	rs2814949	34656722	3.89E-02	2.86E-06	32.02075259	1.89451E-06
4	GeneID:100128183	*PABPC1P7*	103817886	103819829	rs10012413	103817531	4.99E-05	2.35E-03	31.91521505	1.99097E-06
16	GeneID:65057	*ACD*	67691415	67694718	rs73597575	67677001	1.31E-02	9.09E-06	31.88112372	2.02316E-06
12	GeneID:140465	*MYL6B*	56546204	56551771	rs7960225	56564811	1.48E-02	8.25E-06	31.83912488	2.06353E-06

Chr, chromosome number; Gene.id, Gene ID; Name, Gene; Start, start point of the gene; End, end point of the gene; SNP.id, top SNP associated with the gene; SNP.pos, position of the top SNP; pval(headache), p-value of the gene in headache dataset; pval(hypoPAN), p-value of the gene in hypothyroidism dataset

Supplementary Table S11. Genes overlapping headache and hyperthyroidism at $p < 0.05$ and Fisher combined p-value ($p_{FCP} < 2.09 \times 10^{-6}$).

Chr	Gene.id	Name	Start	End	SNP.id	SNP.pos	pval(headache)	pval(hyperPAN)	X^2,4df	FCP
6	GeneID:3117	*HLA-DQA1*	32605183	32611429	rs9272729	32609594	4.50E-05	2.12E-103	492.8471481	2.3607E-105
6	GeneID:3119	*HLA-DQB1*	32627657	32634466	rs1130389	32632627	1.09E-05	1.16E-102	492.2771185	3.1356E-105
6	GeneID:100131609	*HNRNPA1P2*	32293175	32294298	rs116667074	32285362	7.28E-06	3.36E-100	481.7524418	5.9208E-103
6	GeneID:10665	*C6orf10*	32260475	32339656	rs116667074	32285362	1.46E-05	6.76E-100	478.9579529	2.3806E-102
6	GeneID:3127	*HLA-DRB5*	32485151	32498006	rs115250958	32509842	3.77E-05	1.74E-99	475.1804575	1.5614E-101
6	GeneID:3128	*HLA-DRB6*	32520490	32527779	rs115250958	32509842	3.81E-05	1.76E-99	475.1275133	1.6032E-101
6	GeneID:721	*C4B*	31982572	32003195	rs433061	32014828	1.16E-07	2.26E-92	453.9823452	5.9828E-97
6	GeneID:100302242	*MIR1236*	31924616	31924717	rs1270942	31918860	1.43E-07	2.35E-92	453.4845579	7.66519E-97
6	GeneID:7936	*RDBP*	31919864	31926864	rs1270942	31918860	1.48E-07	2.44E-92	453.3431535	8.2242E-97
6	GeneID:6499	*SKIV2L*	31926581	31937532	rs1270942	31918860	1.75E-07	2.88E-92	452.6725223	1.14836E-96
6	GeneID:629	*CFB*	31913721	31919861	rs1270942	31918860	1.76E-07	2.90E-92	452.649674	1.1615E-96
6	GeneID:1589	*CYP21A2*	32006093	32009447	rs433061	32014828	1.77E-07	3.46E-92	452.287627	1.39088E-96
6	GeneID:7148	*TNXB*	32008932	32077151	rs1269852	32080191	3.04E-07	3.88E-92	450.9781215	2.66927E-96
6	GeneID:717	*C2*	31868776	31913449	rs1270942	31918860	2.79E-07	4.59E-92	450.8075242	2.90585E-96
6	GeneID:1388	*ATF6B*	32083045	32096017	rs1269852	32080191	1.64E-07	1.98E-91	448.9452753	7.34287E-96
6	GeneID:4758	*NEU1*	31826829	31830709	rs501942	31840477	5.11E-07	4.58E-89	435.7903894	5.12337E-93
6	GeneID:80736	*SLC44A4*	31830969	31846823	rs501942	31840477	4.53E-07	6.18E-89	435.429764	6.13065E-93
6	GeneID:10919	*EHMT2*	31847536	31865464	rs501942	31840477	4.78E-07	6.52E-89	435.2195593	6.8068E-93
6	GeneID:80740	*LY6G6C*	31686425	31689510	rs3131383	31704294	1.48E-07	6.60E-88	432.9394231	2.11737E-92
6	GeneID:80739	*C6orf25*	31691121	31694487	rs3101018	31705864	1.77E-07	1.02E-87	431.714517	3.89545E-92
6	GeneID:23564	*DDAH2*	31694817	31698039	rs3132445	31712196	2.03E-07	1.17E-87	431.1510321	5.15647E-92
6	GeneID:1192	*CLIC1*	31698358	31704341	rs3130484	31715882	2.25E-07	1.44E-87	430.5394497	6.99105E-92
6	GeneID:401251	*C6orf26*	31730773	31732627	rs3130484	31715882	2.01E-07	2.15E-87	429.9588817	9.33313E-92
6	GeneID:80737	*C6orf27*	31733371	31745108	rs3117573	31718396	2.15E-07	2.17E-87	429.808335	1.00593E-91
6	GeneID:100532732	*MSH5-C6orf26*	31707725	31732627	rs3130484	31715882	3.09E-07	1.98E-87	429.2596351	1.32179E-91
**Chr**	**Gene.id**	**Name**	**Start**	**End**	**SNP.id**	**SNP.pos**	**pval(headache)**	**pval(hyperPAN)**	**X^2,4df**	**FCP**

6	GeneID:5491	*PPIAP9*	31486754	31488108	rs3130614	31476458	2.59E-07	6.94E-80	394.8727358	3.5653E-84
6	GeneID:4277	*MICB*	31465855	31478901	rs3130614	31476458	4.11E-07	1.10E-79	393.0203999	8.95971E-84
6	GeneID:4049	*LTA*	31539876	31542098	rs1800628	31546850	1.76E-07	3.22E-79	392.5761084	1.11759E-83
6	GeneID:259197	*NCR3*	31556660	31560762	rs1800628	31546850	1.79E-07	3.29E-79	392.4994788	1.16101E-83
6	GeneID:7124	*TNF*	31543350	31546112	rs1800628	31546850	1.86E-07	3.40E-79	392.3582939	1.24549E-83
6	GeneID:7940	*LST1*	31553956	31556686	rs1800628	31546850	1.90E-07	3.49E-79	392.2631839	1.30583E-83
6	GeneID:4050	*LTB*	31548335	31550202	rs1800628	31546850	1.92E-07	3.51E-79	392.2306421	1.32715E-83
6	GeneID:100287329	*LOC100287329*	31527348	31540474	rs1800628	31546850	2.48E-07	4.54E-79	391.2025318	2.21328E-83
6	GeneID:692233	*SNORD117*	31504151	31504226	rs9267488	31514247	6.99E-07	4.67E-78	384.4690171	6.30519E-82
6	GeneID:692199	*SNORD84*	31508878	31508955	rs9267488	31514247	8.34E-07	5.57E-78	383.7621677	8.96177E-82
6	GeneID:534	*ATP6V1G2*	31512228	31514625	rs9267488	31514247	8.95E-07	5.98E-78	383.4793061	1.03157E-81
6	GeneID:100532737	*ATP6V1G2-DDX39B*	31497996	31514625	rs9267488	31514247	1.00E-06	6.68E-78	383.0320507	1.28859E-81
6	GeneID:4795	*NFKBIL1*	31514628	31526606	rs9267488	31514247	1.10E-06	7.34E-78	382.6604054	1.55023E-81
6	GeneID:3106	*HLA-B*	31321649	31324989	rs2523593	31326703	1.14E-06	2.20E-74	366.5735009	4.62455E-78
6	GeneID:729816	*DHFRP2*	31331244	31334742	rs2523593	31326703	1.22E-06	2.34E-74	366.320963	5.24336E-78
6	GeneID:100507444	*PPP1R2P1*	32844255	32847851	rs115591082	32844103	5.24E-06	7.06E-74	361.1907275	6.72256E-77
6	GeneID:5698	*PSMB9*	32821938	32827628	rs45506201	32808299	5.28E-06	1.06E-73	360.3571513	1.01753E-76
6	GeneID:6890	*TAP1*	32812986	32821748	rs45506201	32808299	9.10E-06	1.83E-73	358.177551	3.0076E-76
6	GeneID:100507463	*LOC100507463*	32811863	32814277	rs45506201	32808299	9.66E-06	1.95E-73	357.9399602	3.38474E-76
6	GeneID:5696	*PSMB8*	32808494	32812712	rs45506201	32808299	1.03E-05	2.09E-73	357.6643812	3.88181E-76
6	GeneID:6891	*TAP2*	32789610	32806547	rs45506201	32808299	1.35E-05	2.72E-73	356.595641	6.60413E-76
6	GeneID:29113	*C6orf15*	31079000	31080332	rs2233980	31079644	9.56E-07	1.04E-65	326.9783188	1.63568E-69
6	GeneID:1041	*CDSN*	31082865	31088252	rs2233980	31079644	1.09E-06	1.19E-65	326.4386051	2.13886E-69
6	GeneID:170679	*PSORS1C1*	31082608	31107869	rs2233980	31079644	1.44E-06	1.56E-65	325.3446746	3.68364E-69
6	GeneID:170680	*PSORS1C2*	31105311	31107127	rs3130557	31094703	2.78E-06	1.02E-65	324.8818322	4.63625E-69
6	GeneID:54535	*CCHCR1*	31110216	31126015	rs3132541	31098734	4.22E-06	1.09E-65	323.9139866	7.49971E-69
6	GeneID:3108	*HLA-DMA*	32916391	32920899	rs73396802	32915797	1.41E-03	1.27E-57	275.1501612	2.47507E-58
6	GeneID:6046	*BRD2*	32936437	32949282	rs57651384	32923849	1.52E-03	1.31E-57	274.9329354	2.75687E-58
**Chr**	**Gene.id**	**Name**	**Start**	**End**	**SNP.id**	**SNP.pos**	**pval(headache)**	**pval(hyperPAN)**	**X^2,4df**	**FCP**

6	GeneID:3109	*HLA-DMB*	32902406	32908847	rs73396802	32915797	1.55E-03	2.22E-57	273.8410297	4.74029E-58
6	GeneID:720	*C4A*	31949834	31970457	rs6941112	31946614	2.32E-04	1.22E-50	246.5989953	3.51704E-52
6	GeneID:1797	*DOM3Z*	31937588	31940032	rs6941112	31946614	2.56E-04	1.34E-50	246.2035578	4.27912E-52
6	GeneID:8859	*STK19*	31938952	31949223	rs6941112	31946614	2.56E-04	1.34E-50	246.2035578	4.27912E-52
6	GeneID:3112	*HLA-DOB*	32780540	32784825	rs241437	32797684	4.26E-03	1.12E-47	227.1240215	5.49144E-48
6	GeneID:100129192	*MICC*	30382490	30387543	rs149543464	30400763	2.63E-07	8.55E-43	224.0300348	2.54467E-47
6	GeneID:3879	*KRT18P1*	28936847	28938244	rs148696809	28934352	5.80E-09	5.15E-32	182.0172361	2.74972E-38
6	GeneID:100189111	*TRNAM8*	28921042	28921114	rs148696809	28934352	7.63E-09	6.77E-32	180.924014	4.72161E-38
6	GeneID:26695	*OR2U1P*	29230436	29231856	rs144447022	29244219	6.18E-09	2.29E-31	178.9081197	1.27945E-37
6	GeneID:26694	*OR2U2P*	29236242	29237198	rs144447022	29244219	7.17E-09	2.66E-31	178.3095379	1.72014E-37
6	GeneID:442190	*OR2B4P*	29258373	29259527	rs144447022	29244219	9.80E-09	3.63E-31	177.0609619	3.18913E-37
6	GeneID:100189035	*TRNAT5*	28456770	28456843	rs34676049	28453618	2.70E-08	6.43E-29	164.6823526	1.44724E-34
6	GeneID:7211	*TRMEP1*	28448503	28448575	rs34676049	28453618	2.74E-08	6.54E-29	164.6190632	1.4932E-34
6	GeneID:100189344	*TRNAT16*	28442329	28442402	rs34676049	28453618	2.76E-08	6.59E-29	164.5876642	1.51655E-34
6	GeneID:7209	*TRNAL47P*	28446350	28446431	rs34676049	28453618	2.85E-08	6.80E-29	164.462346	1.6134E-34
6	GeneID:100189118	*TRNAL12*	28446400	28446481	rs34676049	28453618	2.85E-08	6.80E-29	164.462346	1.6134E-34
6	GeneID:222698	*NKAPL*	28227098	28228736	rs17720293	28214698	1.85E-08	4.39E-28	161.6028597	6.62439E-34
6	GeneID:3304	*HSPA1B*	31795512	31798031	rs1043618	31783507	6.14E-04	3.14E-32	159.8703493	1.55859E-33
6	GeneID:84547	*PGBD1*	28249314	28270326	rs33932084	28268824	4.40E-08	5.59E-28	159.3789637	1.98662E-33
6	GeneID:7741	*ZNF187*	28234788	28245981	rs13208096	28225311	6.43E-08	3.91E-28	159.3408229	2.02439E-33
6	GeneID:3305	*HSPA1L*	31777396	31782835	rs1043618	31783507	7.84E-04	4.01E-32	158.8917802	2.52692E-33
6	GeneID:3303	*HSPA1A*	31783291	31785719	rs1043618	31783507	8.06E-04	4.12E-32	158.7801093	2.67017E-33
6	GeneID:64288	*ZNF323*	28292514	28324048	rs13214023	28332141	5.49E-08	7.61E-28	158.3204635	3.35048E-33
6	GeneID:57819	*LSM2*	31765173	31774743	rs1043618	31783507	9.25E-04	4.73E-32	158.2279906	3.50701E-33
6	GeneID:7746	*ZNF193*	28193029	28201265	rs17720293	28214698	2.28E-08	1.95E-27	158.1948607	3.56485E-33
6	GeneID:387032	*ZKSCAN4*	28212490	28220002	rs17720293	28214698	2.24E-08	1.99E-27	158.1849703	3.5823E-33
6	GeneID:26719	*OR2E1P*	28423307	28423934	rs67381177	28411941	3.44E-08	1.31E-27	158.1720726	3.60519E-33
6	GeneID:100189159	*TRNAS13*	28180815	28180896	rs13197633	28174757	3.37E-08	1.69E-27	157.7041654	4.54215E-33
**Chr**	**Gene.id**	**Name**	**Start**	**End**	**SNP.id**	**SNP.pos**	**pval(headache)**	**pval(hyperPAN)**	**X^2,4df**	**FCP**

6	GeneID:222699	*TOB2P1*	28183116	28186707	rs13197633	28174757	3.70E-08	1.85E-27	157.3319748	5.45844E-33
6	GeneID:9753	*ZSCAN12*	28346598	28367544	rs35883476	28368508	3.71E-08	2.28E-27	156.9088988	6.72642E-33
6	GeneID:100419756	*LOC100419756*	28376002	28377027	rs35883476	28368508	3.09E-08	2.76E-27	156.8922258	6.78201E-33
6	GeneID:140468	*COX11P1*	28414697	28415407	rs67381177	28411941	3.40E-08	2.79E-27	156.6814076	7.52594E-33
6	GeneID:222696	*ZSCAN23*	28400432	28411279	rs13201681	28394680	3.46E-08	3.16E-27	156.3975991	8.65788E-33
6	GeneID:80317	*ZKSCAN3*	28317691	28336954	rs13214023	28332141	5.02E-08	2.68E-27	155.9845713	1.06161E-32
6	GeneID:222701	*ZNF192P2*	28155716	28157189	rs13195291	28169241	3.64E-08	1.34E-25	148.7992996	3.68139E-31
6	GeneID:651302	*ZNF192P1*	28129551	28137376	rs35749575	28114818	3.58E-08	1.39E-25	148.765986	3.7424E-31
6	GeneID:100419608	*LOC100419608*	28083427	28083998	rs66886492	28089731	2.61E-08	1.93E-25	148.7384049	3.79367E-31
6	GeneID:100189315	*TRNAM17*	27870271	27870342	rs13218875	27884012	3.31E-08	1.62E-25	148.6137362	4.03433E-31
6	GeneID:100189314	*TRNAG29*	27870686	27870756	rs13218875	27884012	3.41E-08	1.67E-25	148.4967279	4.27408E-31
6	GeneID:26212	*OR2B6*	27925019	27925960	rs149583087	27912437	1.69E-08	3.49E-25	148.4234477	4.43142E-31
6	GeneID:81697	*OR2B2*	27878963	27880174	rs13218875	27884012	3.53E-08	1.73E-25	148.3592279	4.57407E-31
6	GeneID:7745	*ZNF192*	28109716	28125236	rs35749575	28114818	3.81E-08	1.71E-25	148.2262665	4.88417E-31
6	GeneID:100147813	*RNU7-26P*	27865284	27865343	rs61742093	27879982	4.37E-08	1.50E-25	148.2035874	4.93913E-31
6	GeneID:81694	*OR2W2P*	28001693	28002640	rs13193295	28003228	2.65E-08	2.85E-25	147.9207943	5.67858E-31
6	GeneID:81406	*OR2W6P*	27905182	27906179	rs149583087	27912437	1.95E-08	4.02E-25	147.8519873	5.87464E-31
6	GeneID:80345	*ZSCAN16*	28092387	28097857	rs66886492	28089731	3.25E-08	2.57E-25	147.7239854	6.25757E-31
6	GeneID:442175	*RPLP2P1*	27932953	27933234	rs28360499	27945396	2.22E-08	4.36E-25	147.427854	7.24186E-31
6	GeneID:340192	*IQCB2P*	27978329	27980680	rs71559067	27994416	3.19E-08	3.41E-25	147.1952496	8.12237E-31
6	GeneID:221584	*ZSCAN12P1*	28058929	28063493	rs35952432	28074901	4.07E-08	2.89E-25	147.0412489	8.76345E-31
6	GeneID:81695	*OR2B7P*	28014213	28015147	rs13193295	28003228	2.81E-08	4.25E-25	147.007402	8.91099E-31
6	GeneID:100129195	*LOC100129195*	28058336	28105071	rs35749575	28114818	4.09E-08	3.00E-25	146.9547775	9.14534E-31
6	GeneID:81407	*OR2W4P*	27944929	27945851	rs28360499	27945396	2.52E-08	4.95E-25	146.9211175	9.29846E-31
6	GeneID:65944	*OR2B8P*	28020906	28022043	rs13200214	28017250	3.71E-08	4.98E-25	146.1412625	1.36607E-30
6	GeneID:442179	*OR1F12*	28041026	28042002	rs71559070	28038929	5.63E-08	3.84E-25	145.8234729	1.5979E-30
6	GeneID:8348	*HIST1H2BO*	27861203	27861669	rs13199649	27868792	4.66E-08	4.99E-25	145.6770201	1.71759E-30
6	GeneID:8336	*HIST1H2AM*	27860477	27860963	rs13199649	27868792	4.67E-08	4.99E-25	145.6724928	1.72143E-30
**Chr**	**Gene.id**	**Name**	**Start**	**End**	**SNP.id**	**SNP.pos**	**pval(headache)**	**pval(hyperPAN)**	**X^2,4df**	**FCP**

6	GeneID:100507241	*LOC100507241*	28046570	28048908	rs35902873	28058949	6.06E-08	4.20E-25	145.4985104	1.87567E-30
6	GeneID:8356	*HIST1H3J*	27858093	27858570	rs13199649	27868792	4.98E-08	5.33E-25	145.410396	1.95899E-30
6	GeneID:7718	*ZNF165*	28048482	28057341	rs34166054	28065801	6.34E-08	4.39E-25	145.3181574	2.05017E-30
6	GeneID:100189131	*TRNAI6*	27599200	27599293	rs35715914	27592003	2.50E-08	2.82E-24	143.4570362	5.1334E-30
6	GeneID:100189300	*TRNAT13*	27586135	27586208	rs35715914	27592003	2.53E-08	2.85E-24	143.4171827	5.23528E-30
6	GeneID:10340	*HIST1H2BPS2*	27831840	27832179	rs45509595	27840926	8.08E-08	1.08E-24	143.0375362	6.31312E-30
6	GeneID:8332	*HIST1H2AL*	27833107	27833576	rs45509595	27840926	8.22E-08	1.10E-24	142.9712725	6.52281E-30
6	GeneID:3009	*HIST1H1B*	27834570	27835359	rs45509595	27840926	8.37E-08	1.11E-24	142.9002058	6.75544E-30
6	GeneID:8368	*HIST1H4L*	27840926	27841289	rs45509595	27840926	8.63E-08	1.15E-24	142.7775351	7.17668E-30
6	GeneID:8354	*HIST1H3I*	27839623	27840099	rs45509595	27840926	8.90E-08	1.19E-24	142.6530588	7.63097E-30
6	GeneID:100189205	*TRNAS15*	27640229	27640310	rs13217620	27653120	3.55E-08	3.67E-24	142.232912	9.3875E-30
6	GeneID:100189161	*TRNAV17*	27648885	27648957	rs13217620	27653120	3.64E-08	3.77E-24	142.1256677	9.89725E-30
6	GeneID:100189428	*TRNAR29*	27638344	27638416	rs13217620	27653120	3.82E-08	3.96E-24	141.932075	1.08885E-29
6	GeneID:100189340	*TRNAT15*	27652474	27652547	rs13217620	27653120	4.05E-08	4.19E-24	141.7026757	1.21924E-29
6	GeneID:100189071	*TRNAI4*	27655967	27656040	rs13217620	27653120	4.15E-08	4.29E-24	141.6092069	1.27674E-29
6	GeneID:7738	*ZNF184*	27418521	27440897	rs35716472	27406607	9.90E-09	2.19E-23	141.2095389	1.55482E-29
6	GeneID:100189352	*TRNAS24*	27509554	27509635	rs35848276	27521096	3.03E-08	8.16E-24	140.9498883	1.76715E-29
6	GeneID:94017	*TRNAS1*	27513468	27513549	rs35848276	27521096	3.07E-08	8.25E-24	140.9039049	1.80767E-29
6	GeneID:100189268	*TRNAQ21*	27515531	27515602	rs35848276	27521096	3.08E-08	8.28E-24	140.8897772	1.82031E-29
6	GeneID:100189070	*TRNAS9*	27521192	27521273	rs35848276	27521096	3.30E-08	8.87E-24	140.6159875	2.08336E-29
6	GeneID:100189012	*TRNAR5*	27529963	27530049	rs35848276	27521096	3.57E-08	9.61E-24	140.294354	2.44133E-29
6	GeneID:100128240	*LOC100128240*	27528605	27529550	rs35848276	27521096	3.57E-08	9.61E-24	140.293067	2.44288E-29
6	GeneID:100507173	*LOC100507173*	27661814	27678001	rs13217620	27653120	6.23E-08	6.45E-24	139.9767566	2.8551E-29
6	GeneID:100189067	*TRNAL10*	27198334	27198416	rs4713071	27187919	5.29E-10	8.66E-22	139.7182869	3.24306E-29
6	GeneID:100189041	*TRNAS7*	27177628	27177709	rs4713071	27187919	4.97E-10	9.56E-22	139.6416656	3.3679E-29
6	GeneID:7212	*TRNAM2*	27745664	27745735	rs35037868	27759115	7.31E-08	6.63E-24	139.6042868	3.43053E-29
6	GeneID:100189167	*TRNAR17*	27181623	27181695	rs4713071	27187919	5.12E-10	9.84E-22	139.524717	3.56775E-29
6	GeneID:100129755	*RSL24D1P1*	27748462	27748890	rs35037868	27759115	7.50E-08	6.81E-24	139.4979661	3.61511E-29
**Chr**	**Gene.id**	**Name**	**Start**	**End**	**SNP.id**	**SNP.pos**	**pval(headache)**	**pval(hyperPAN)**	**X^2,4df**	**FCP**

6	GeneID:100189368	*TRNAV28*	27173867	27173939	rs4713071	27187919	5.18E-10	9.95E-22	139.4820314	3.64362E-29
6	GeneID:100189074	*TRNAQ10*	27763640	27763711	rs35037868	27759115	7.56E-08	6.86E-24	139.4697483	3.66575E-29
6	GeneID:100189099	*TRNAR10*	27182952	27183024	rs4713071	27187919	5.21E-10	1.00E-21	139.4561683	3.69037E-29
6	GeneID:442171	*RPL10P2*	27179023	27179663	rs4713071	27187919	5.26E-10	1.01E-21	139.4168067	3.76267E-29
6	GeneID:100189214	*TRNAQ17*	27759135	27759206	rs35037868	27759115	8.18E-08	7.42E-24	139.1523664	4.28653E-29
6	GeneID:100189101	*TRNAV15*	27696327	27696399	rs34409918	27685348	1.15E-07	9.37E-24	137.9972595	7.5747E-29
6	GeneID:100189164	*TRNAL16*	27688898	27688980	rs34409918	27685348	1.22E-07	9.88E-24	137.7867344	8.40285E-29
6	GeneID:100189374	*TRNAT18*	27694473	27694546	rs34409918	27685348	1.23E-07	1.00E-23	137.7348705	8.62041E-29
6	GeneID:394254	*GPR89P*	27704354	27706293	rs13202291	27698857	1.60E-07	9.03E-24	137.4245137	1.00451E-28
6	GeneID:100189310	*TRNAI17*	27205350	27205423	rs35589403	27219491	9.44E-10	1.61E-21	137.3190874	1.05808E-28
6	GeneID:645927	*MCFD2P1*	27375502	27377470	rs34071253	27391802	1.07E-08	1.50E-22	137.2157192	1.11338E-28
6	GeneID:10279	*PRSS16*	27215502	27224399	rs35589403	27219491	1.09E-09	1.86E-21	136.7456028	1.40365E-28
6	GeneID:442172	*LOC442172*	27235885	27237008	rs35982103	27243134	1.09E-09	1.92E-21	136.6811013	1.44898E-28
6	GeneID:7202	*TRNAI1*	27241739	27241812	rs35982103	27243134	1.11E-09	1.97E-21	136.5866695	1.518E-28
6	GeneID:346157	*ZNF391*	27356524	27369227	rs67859638	27357978	1.21E-08	1.86E-22	136.5373525	1.55535E-28
6	GeneID:100189213	*TRNAI11*	27242990	27243063	rs35982103	27243134	1.13E-09	2.00E-21	136.526175	1.56394E-28
6	GeneID:7754	*ZNF204P*	27325602	27343153	rs67859638	27357978	1.16E-08	2.08E-22	136.3869608	1.67499E-28
6	GeneID:100189014	*TRNAV12*	27203288	27203360	rs13212921	27205422	1.91E-09	1.56E-21	135.9623779	2.06479E-28
6	GeneID:100287272	*USP8P1*	31243351	31246528	rs9264490	31232578	5.11E-05	8.93E-26	135.1205135	3.12626E-28
6	GeneID:100189345	*TRNAM18*	27300764	27300835	rs71537559	27309779	3.24E-08	2.08E-22	134.3427711	4.58606E-28
6	GeneID:100189336	*TRNAI20*	27144994	27145067	rs34569203	27153984	2.62E-10	2.64E-20	134.2835347	4.72187E-28
6	GeneID:100189182	*TRNAK19*	27302769	27302841	rs71537559	27309779	3.33E-08	2.14E-22	134.2320019	4.84329E-28
6	GeneID:8363	*HIST1H4J*	27791903	27792258	rs35202262	27799514	1.21E-07	6.72E-23	133.9672912	5.51794E-28
6	GeneID:3107	*HLA-C*	31236529	31239855	rs1793890	31222056	6.16E-05	1.38E-25	133.8794583	5.76194E-28
6	GeneID:387317	*VN1R11P*	27051071	27051995	rs6904071	27047256	1.02E-09	8.39E-21	133.853817	5.83519E-28
6	GeneID:100189157	*TRNAS12*	27065085	27065166	rs34388707	27050396	8.80E-10	1.02E-20	133.7640259	6.0991E-28
6	GeneID:100189301	*TRNAL28*	27573417	27573524	rs34105070	27560805	3.65E-08	2.49E-22	133.7389824	6.17481E-28
6	GeneID:8362	*HIST1H4K*	27798952	27799305	rs35202262	27799514	1.34E-07	6.86E-23	133.7124981	6.2559E-28
**Chr**	**Gene.id**	**Name**	**Start**	**End**	**SNP.id**	**SNP.pos**	**pval(headache)**	**pval(hyperPAN)**	**X^2,4df**	**FCP**

6	GeneID:100189143	*TRNAT7*	27130050	27130123	rs13194053	27143883	4.41E-10	2.10E-20	133.7052474	6.27829E-28
6	GeneID:791231	*TRNAV8*	27618707	27618779	rs144969912	27616843	2.89E-08	3.29E-22	133.654709	6.43656E-28
6	GeneID:8341	*HIST1H2BN*	27806440	27806888	rs35202262	27799514	1.38E-07	7.19E-23	133.5672351	6.71998E-28
6	GeneID:8330	*HIST1H2AK*	27805658	27806117	rs35202262	27799514	1.38E-07	7.19E-23	133.5662836	6.72313E-28
6	GeneID:100189172	*TRNAP10*	27059521	27059592	rs6904071	27047256	8.75E-10	1.15E-20	133.5421123	6.80367E-28
6	GeneID:387046	*RPL8P1*	27620370	27620512	rs144969912	27616843	3.02E-08	3.44E-22	133.4761947	7.02822E-28
6	GeneID:100189038	*TRNAL8*	27570348	27570454	rs34105070	27560805	3.95E-08	2.70E-22	133.4204299	7.22397E-28
6	GeneID:100189297	*TRNAM15*	27560600	27560671	rs34105070	27560805	4.16E-08	2.84E-22	133.2156259	7.99082E-28
6	GeneID:790951	*TRNAS4*	27499987	27500068	rs13195636	27509493	2.93E-08	5.08E-22	132.7506296	1.00477E-27
6	GeneID:100189095	*TRNAI5*	27636362	27636435	rs56405707	27640246	4.12E-08	3.66E-22	132.7283732	1.01585E-27
6	GeneID:8969	*HIST1H2AG*	27100817	27101314	rs67330695	27103654	7.43E-10	2.12E-20	132.6407316	1.06066E-27
6	GeneID:85236	*HIST1H2BK*	27106072	27114637	rs61240102	27124904	7.30E-10	2.16E-20	132.6401219	1.06098E-27
6	GeneID:8970	*HIST1H2BJ*	27100095	27100575	rs67330695	27103654	7.66E-10	2.18E-20	132.5225826	1.12422E-27
6	GeneID:387322	*VN1R13P*	27028149	27029046	rs35608615	27040402	2.60E-09	8.84E-21	131.8859587	1.53827E-27
6	GeneID:100189462	*TRNAI28P*	27251864	27251937	rs35982103	27243134	1.35E-09	1.81E-20	131.7612361	1.63573E-27
6	GeneID:100189363	*TRNAV27*	27248049	27248121	rs35982103	27243134	1.38E-09	1.84E-20	131.6862912	1.69724E-27
6	GeneID:100189219	*TRNAY8*	26595102	26595190	rs34781270	26593037	8.07E-10	3.29E-20	131.596669	1.77383E-27
6	GeneID:100189200	*TRNAD9*	27551236	27551307	rs34105070	27560805	4.26E-08	6.39E-22	131.546418	1.81828E-27
6	GeneID:100189053	*TRNAK8*	27559593	27559665	rs34105070	27560805	4.29E-08	6.42E-22	131.522724	1.83962E-27
6	GeneID:11119	*BTN3A1*	26402465	26415444	rs2072803	26392515	1.17E-10	2.43E-19	131.4647085	1.89294E-27
6	GeneID:100189408	*TRNAV31*	27258405	27258477	rs13207082	27251379	2.01E-09	1.57E-20	131.2482653	2.10588E-27
6	GeneID:100189390	*TRNAK31*	27543846	27543918	rs10484399	27534528	5.29E-08	5.99E-22	131.2419683	2.11242E-27
6	GeneID:29777	*ABT1*	26597171	26600278	rs34781270	26593037	8.44E-10	3.88E-20	131.1773656	2.18071E-27
6	GeneID:10337	*HIST1H4PS1*	27774945	27775115	rs13195728	27771106	1.50E-07	2.26E-22	131.1069551	2.25765E-27
6	GeneID:8329	*HIST1H2AI*	27775977	27776445	rs13195728	27771106	1.53E-07	2.29E-22	131.0457083	2.32679E-27
6	GeneID:8340	*HIST1H2BL*	27775257	27775709	rs13195728	27771106	1.54E-07	2.31E-22	131.0162737	2.36076E-27
6	GeneID:645950	*HNRNPA1P1*	27491226	27492316	rs34573979	27480526	1.61E-08	2.21E-21	131.0063992	2.37227E-27
6	GeneID:100189162	*TRNAY7*	26577332	26577420	rs13190739	26587373	9.73E-10	3.70E-20	130.9877844	2.39412E-27
**Chr**	**Gene.id**	**Name**	**Start**	**End**	**SNP.id**	**SNP.pos**	**pval(headache)**	**pval(hyperPAN)**	**X^2,4df**	**FCP**

6	GeneID:100189526	*TRNAS32P*	27261671	27261744	rs13207082	27251379	2.15E-09	1.68E-20	130.977748	2.40598E-27
6	GeneID:387316	*VN1R10P*	27292695	27293631	rs56401801	27301512	4.43E-08	8.29E-22	130.9488136	2.44051E-27
6	GeneID:54718	*BTN2A3P*	26421619	26430816	rs9379875	26444732	4.74E-10	7.98E-20	130.8890807	2.51337E-27
6	GeneID:100189329	*TRNAQ27*	27263212	27263283	rs13207082	27251379	2.22E-09	1.74E-20	130.8521378	2.55952E-27
6	GeneID:100189002	*TRNAS5*	27265775	27265856	rs13207082	27251379	2.16E-09	1.80E-20	130.8317557	2.58534E-27
6	GeneID:83954	*FKSG83*	27292578	27293742	rs56401801	27301512	4.58E-08	8.57E-22	130.8165001	2.60483E-27
6	GeneID:8342	*HIST1H2BM*	27782822	27783267	rs13195728	27771106	1.63E-07	2.45E-22	130.774732	2.65897E-27
6	GeneID:79692	*ZNF322*	26634611	26659980	rs149123117	26670618	7.23E-10	5.68E-20	130.7248012	2.72516E-27
6	GeneID:8357	*HIST1H3H*	27777842	27778314	rs13195728	27771106	1.65E-07	2.49E-22	130.7235653	2.72682E-27
6	GeneID:8331	*HIST1H2AJ*	27782080	27782518	rs13195728	27771106	1.66E-07	2.50E-22	130.7050113	2.75185E-27
6	GeneID:100189189	*TRNAQ16*	27487308	27487379	rs34573979	27480526	1.74E-08	2.39E-21	130.6942031	2.76654E-27
6	GeneID:100189337	*TRNAY12*	26575798	26575887	rs13190739	26587373	1.07E-09	4.06E-20	130.6230442	2.8652E-27
6	GeneID:791230	*TRNAV7*	27721179	27721251	rs17693963	27710165	1.55E-07	2.89E-22	130.5543215	2.96383E-27
6	GeneID:100131289	*LOC100131289*	27729523	27730966	rs35501037	27739566	1.70E-07	2.67E-22	130.5238055	3.00871E-27
6	GeneID:100270746	*LOC100270746*	26987145	26988085	rs13217285	26999845	4.70E-09	1.15E-20	130.1831331	3.55826E-27
6	GeneID:100189028	*TRNAI2*	26988125	26988218	rs13217285	26999845	4.86E-09	1.19E-20	130.0483416	3.80246E-27
6	GeneID:100189372	*TRNAI22*	26745255	26745328	rs13213200	26755915	2.38E-10	2.46E-19	130.0174634	3.86072E-27
6	GeneID:10385	*BTN2A2*	26383324	26395102	rs2072803	26392515	1.73E-10	3.61E-19	129.8856555	4.11961E-27
6	GeneID:387321	*VN1R12P*	27001125	27002122	rs35741362	27007687	4.07E-09	1.57E-20	129.8354145	4.2228E-27
6	GeneID:100126484	*TRNAM-CAU*	26735574	26735646	rs35144506	26739487	5.93E-10	1.16E-19	129.6914549	4.533E-27
6	GeneID:696	*BTN1A1*	26501495	26510653	rs34148261	26511744	1.28E-09	5.62E-20	129.5992307	4.7436E-27
6	GeneID:100189272	*TRNAY10*	26569086	26569176	rs72844462	26563864	1.98E-09	3.82E-20	129.5045956	4.96987E-27
6	GeneID:100009603	*TRNAA2*	26572092	26572164	rs72844462	26563864	2.07E-09	4.00E-20	129.3257357	5.42742E-27
6	GeneID:387320	*VN1R14P*	26631313	26631964	rs67777156	26633711	1.99E-09	4.67E-20	129.0867765	6.10508E-27
6	GeneID:10384	*BTN3A3*	26440700	26453643	rs9379875	26444732	6.71E-10	1.53E-19	128.8917373	6.72043E-27
6	GeneID:100652981	*LOC100652981*	26854360	26854727	rs77666565	26851415	1.36E-09	1.51E-19	127.5025973	1.33171E-26
6	GeneID:100420941	*LOC100420941*	26478093	26478749	rs13195401	26463574	7.28E-10	2.94E-19	127.4212788	1.38611E-26
6	GeneID:729392	*POM121L6P*	26838446	26865940	rs77666565	26851415	1.77E-09	1.96E-19	126.4497651	2.23607E-26
**Chr**	**Gene.id**	**Name**	**Start**	**End**	**SNP.id**	**SNP.pos**	**pval(headache)**	**pval(hyperPAN)**	**X^2,4df**	**FCP**

6	GeneID:387036	*GUSBP2*	26839266	26924333	rs77666565	26851415	2.51E-09	1.46E-19	126.3452953	2.35406E-26
6	GeneID:11120	*BTN2A1*	26458153	26476849	rs9379875	26444732	7.43E-10	5.28E-19	126.211557	2.51423E-26
6	GeneID:94026	*POM121L2*	27276842	27280011	rs56114371	27274834	2.66E-08	1.57E-20	126.091542	2.66723E-26
6	GeneID:352996	*P5-04*	29910314	29910426	rs2524005	29899677	3.23E-04	1.31E-24	126.0580137	2.71161E-26
6	GeneID:3105	*HLA-A*	29910247	29913661	rs2524005	29899677	3.23E-04	1.31E-24	126.053422	2.71774E-26
6	GeneID:352994	*P5-05*	29894219	29894350	rs2524005	29899677	3.24E-04	1.32E-24	126.0435682	2.73096E-26
6	GeneID:285819	*LOC285819*	26472172	26482737	rs13195401	26463574	1.02E-09	4.21E-19	126.0221804	2.75986E-26
6	GeneID:80868	*HCG4B*	29892369	29893428	rs2524005	29899677	3.31E-04	1.35E-24	125.9524776	2.85618E-26
6	GeneID:3138	*HLA-K*	29894436	29897616	rs2524005	29899677	3.34E-04	1.36E-24	125.9224376	2.89872E-26
6	GeneID:353003	*HCG4P5*	29908689	29909578	rs2524005	29899677	3.36E-04	1.37E-24	125.8914393	2.94329E-26
6	GeneID:352965	*HLA-U*	29901541	29902657	rs2524005	29899677	3.42E-04	1.39E-24	125.8260977	3.03948E-26
6	GeneID:352995	*P5.8*	29903015	29904157	rs2524005	29899677	3.59E-04	1.46E-24	125.6306466	3.34639E-26
6	GeneID:23540	*TRNAS3*	27473607	27473688	rs34573979	27480526	1.56E-08	4.28E-20	125.1454114	4.24903E-26
6	GeneID:100189292	*TRNAD14*	27471523	27471594	rs34573979	27480526	1.64E-08	4.50E-20	124.9409287	4.69887E-26
6	GeneID:100189247	*TRNAS19*	27470818	27470899	rs34573979	27480526	1.70E-08	4.65E-20	124.8137313	5.00241E-26
6	GeneID:23437	*TRNAS2*	27463593	27463674	rs58616630	27474715	1.71E-08	4.96E-20	124.6663749	5.37863E-26
6	GeneID:100189077	*TRNAS10*	27446591	27446672	rs7749305	27446566	1.40E-07	5.96E-20	120.0963523	5.09426E-25
6	GeneID:100189158	*TRNAD8*	27447453	27447524	rs7749305	27446566	1.41E-07	6.01E-20	120.0586113	5.1897E-25
6	GeneID:100189288	*TRNAL26*	26521436	26521518	rs66941101	26530376	5.88E-10	7.08E-17	116.880848	2.47587E-24
6	GeneID:7231	*TRNAR2*	26537726	26537798	rs66941101	26530376	5.18E-10	8.13E-17	116.8600286	2.50134E-24
6	GeneID:100189227	*TRNAV21*	26538282	26538354	rs66941101	26530376	5.21E-10	8.18E-17	116.8356394	2.53151E-24
6	GeneID:100189319	*TRNAT14*	26533145	26533218	rs66941101	26530376	6.04E-10	9.74E-17	116.191704	3.47415E-24
6	GeneID:493812	*HCG11*	26521934	26527621	rs66941101	26530376	7.02E-10	8.45E-17	116.1731198	3.50604E-24
6	GeneID:100189223	*TRNAP13*	26555498	26555569	rs34246779	26549212	1.10E-09	5.64E-17	116.086537	3.65847E-24
6	GeneID:100189110	*TRNAK14*	26556774	26556846	rs34246779	26549212	1.11E-09	5.67E-17	116.0611405	3.70442E-24
6	GeneID:100189391	*TRNAM20*	26766444	26766516	rs13213200	26755915	2.73E-10	2.36E-16	116.0086179	3.8013E-24
6	GeneID:100289545	*HIST1H3PS1*	26322104	26343616	rs9393703	26356951	7.53E-10	8.62E-17	115.9924178	3.83169E-24
6	GeneID:100189153	*TRNAI9*	26554350	26554423	rs34246779	26549212	1.13E-09	5.78E-17	115.9892657	3.83763E-24
**Chr**	**Gene.id**	**Name**	**Start**	**End**	**SNP.id**	**SNP.pos**	**pval(headache)**	**pval(hyperPAN)**	**X^2,4df**	**FCP**

6	GeneID:10473	*HMGN4*	26538572	26547165	rs66941101	26530376	7.01E-10	1.10E-16	115.6470584	4.54058E-24
6	GeneID:100189354	*TRNAM19*	26758550	26758622	rs13213200	26755915	3.16E-10	2.73E-16	115.4254975	5.06296E-24
6	GeneID:100189308	*TRNAA29*	26553731	26553802	rs34246779	26549212	1.07E-09	8.57E-17	115.2968217	5.39349E-24
6	GeneID:80864	*EGFL8*	32132382	32136062	rs192471087	32119730	1.94E-03	5.24E-23	115.1005631	5.93963E-24
6	GeneID:100189369	*TRNAA37*	26682715	26682787	rs149123117	26670618	3.78E-10	3.17E-16	114.7680319	6.99411E-24
6	GeneID:100189156	*TRNAA20*	26771290	26771362	rs34244947	26761745	7.29E-10	1.78E-16	114.6125373	7.54951E-24
6	GeneID:80863	*PRRT1*	32116140	32119720	rs192471087	32119730	2.26E-03	6.12E-23	114.4781213	8.06503E-24
6	GeneID:100507547	*LOC100507547*	32120579	32122142	rs192471087	32119730	2.27E-03	6.13E-23	114.4739154	8.08172E-24
6	GeneID:9374	*PPT2*	32121229	32131458	rs192471087	32119730	2.40E-03	6.49E-23	114.2455912	9.04131E-24
6	GeneID:100532746	*PPT2-EGFL8*	32121776	32136062	rs192471087	32119730	2.40E-03	6.49E-23	114.2451296	9.04337E-24
6	GeneID:100189358	*TRNAA36*	26673590	26673662	rs149123117	26670618	4.92E-10	4.13E-16	113.7086751	1.17709E-23
6	GeneID:100507100	*LOC100507100*	26681354	26688069	rs149123117	26670618	5.23E-10	4.39E-16	113.4637843	1.3276E-23
6	GeneID:11118	*BTN3A2*	26365398	26378548	rs2072803	26392515	1.58E-10	1.48E-15	113.4352533	1.34634E-23
6	GeneID:100507141	*LOC100507141*	26864941	26865525	rs77666565	26851415	1.32E-09	4.15E-16	111.7317498	3.10893E-23
6	GeneID:729400	*LOC729400*	26866104	26868093	rs77666565	26851415	1.32E-09	4.16E-16	111.7144726	3.13542E-23
6	GeneID:100126501	*TRNAA-AGC*	26796006	26796078	rs7454868	26799828	3.57E-10	2.03E-15	111.1628361	4.11122E-23
6	GeneID:100129921	*RPL15P4*	31495853	31496498	rs114751021	31504194	6.52E-03	7.40E-22	107.3754523	2.64002E-22
6	GeneID:100189410	*TRNAR28*	26323046	26323118	rs34107459	26328353	1.01E-07	5.16E-17	107.2217591	2.84689E-22
6	GeneID:100189382	*TRNAS25*	26327817	26327898	rs9467714	26340785	9.25E-08	5.80E-17	107.1637685	2.92908E-22
6	GeneID:100189396	*TRNAR26*	26328368	26328440	rs9467714	26340785	9.83E-08	6.16E-17	106.9226578	3.29707E-22
6	GeneID:100189040	*TRNAM4*	26330529	26330600	rs9467714	26340785	9.87E-08	6.19E-17	106.9032616	3.32861E-22
6	GeneID:100189048	*TRNAW2*	26331672	26331743	rs9467714	26340785	1.03E-07	6.47E-17	106.7265798	3.63014E-22
6	GeneID:100189185	*TRNAQ15*	26311975	26312046	rs13220522	26316295	1.10E-07	1.32E-16	105.1807334	7.75141E-22
6	GeneID:100189321	*TRNAQ25*	26311424	26311495	rs13220522	26316295	1.10E-07	1.32E-16	105.1805513	7.7521E-22
6	GeneID:100189436	*TRNAW9*	26319330	26319401	rs34107459	26328353	9.91E-08	1.54E-16	105.0738218	8.16889E-22
6	GeneID:100189094	*TRNAM6*	26313352	26313423	rs34107459	26328353	1.01E-07	1.57E-16	104.9892892	8.51483E-22
6	GeneID:100189430	*TRNAS27*	26312824	26312905	rs13220522	26316295	1.20E-07	1.45E-16	104.8175773	9.26328E-22
6	GeneID:100189218	*TRNAS16*	26305718	26305801	rs13220522	26316295	1.53E-07	1.84E-16	103.8452681	1.49255E-21
**Chr**	**Gene.id**	**Name**	**Start**	**End**	**SNP.id**	**SNP.pos**	**pval(headache)**	**pval(hyperPAN)**	**X^2,4df**	**FCP**

6	GeneID:8344	*HIST1H2BE*	26184024	26184458	rs67575965	26196593	5.08E-09	7.05E-15	103.3690161	1.88533E-21
6	GeneID:7232	*TRNAR3*	26299905	26299977	rs10484439	26309908	1.80E-07	2.40E-16	102.9967861	2.26297E-21
6	GeneID:8345	*HIST1H2BH*	26251879	26252303	rs55706012	26266311	1.36E-07	3.52E-16	102.7840384	2.51186E-21
6	GeneID:8355	*HIST1H3G*	26271146	26271612	rs55706012	26266311	1.37E-07	3.55E-16	102.7592755	2.54255E-21
6	GeneID:8360	*HIST1H4D*	26188938	26189304	rs67575965	26196593	5.99E-09	8.32E-15	102.7082795	2.60694E-21
6	GeneID:8346	*HIST1H2BI*	26273204	26273640	rs55706012	26266311	1.39E-07	3.59E-16	102.7057984	2.61012E-21
6	GeneID:100507025	*LOC100507025*	26281278	26285763	rs55706012	26266311	1.41E-07	3.66E-16	102.636732	2.70004E-21
6	GeneID:8333	*HIST1H2APS4*	26272421	26272768	rs55706012	26266311	1.43E-07	3.71E-16	102.580006	2.77622E-21
6	GeneID:394255	*RPS10P1*	26202351	26202943	rs67575965	26196593	7.07E-09	9.83E-15	102.0402935	3.61746E-21
6	GeneID:8343	*HIST1H2BF*	26199787	26200216	rs67575965	26196593	7.17E-09	9.96E-15	101.9890426	3.70953E-21
6	GeneID:3013	*HIST1H2AD*	26199012	26199471	rs67575965	26196593	7.38E-09	1.02E-14	101.8736624	3.92546E-21
6	GeneID:8351	*HIST1H3D*	26197012	26199464	rs67575965	26196593	7.56E-09	1.05E-14	101.7773068	4.11539E-21
6	GeneID:8367	*HIST1H4E*	26204873	26205249	rs67575965	26196593	6.51E-09	1.53E-14	101.3245698	5.13834E-21
6	GeneID:442191	*OR14J1*	29274467	29275432	rs150254595	29278406	6.51E-04	1.55E-18	96.68658784	4.98887E-20
6	GeneID:100379623	*LOC100379623*	26164261	26168404	rs17598658	26175866	8.29E-08	1.48E-14	96.30114009	6.02562E-20
6	GeneID:352957	*MICF*	29819964	29821829	rs114853934	29812896	8.48E-04	1.80E-18	95.86164362	7.47293E-20
6	GeneID:3017	*HIST1H2BD*	26158349	26171577	rs17598658	26175866	1.06E-07	1.89E-14	95.32378821	9.72504E-20
6	GeneID:353009	*3.8-1.4*	29833692	29834864	rs62389319	29847388	1.35E-03	2.51E-18	94.27802719	1.62287E-19
6	GeneID:100188987	*TRNAH3*	27125906	27125977	rs67540232	27140866	4.78E-10	7.96E-12	94.03417807	1.82866E-19
6	GeneID:100422934	*MIR3143*	27115405	27115467	rs61240102	27124904	6.22E-10	7.73E-12	93.56789875	2.29758E-19
6	GeneID:85235	*HIST1H2AH*	27114908	27115346	rs61240102	27124904	6.22E-10	7.73E-12	93.56771237	2.29779E-19
6	GeneID:8294	*HIST1H4I*	27107088	27107457	rs67330695	27103654	6.42E-10	7.69E-12	93.51446896	2.35847E-19
6	GeneID:100189196	*TRNAV20*	27118022	27118094	rs61240102	27124904	6.31E-10	7.84E-12	93.5092957	2.36445E-19
6	GeneID:401250	*MCCD1*	31496739	31498008	rs114751021	31504194	6.50E-03	1.41E-17	87.67068765	4.11313E-18
6	GeneID:352993	*HCP5P6*	29883530	29885892	rs2524005	29899677	2.56E-04	4.79E-16	87.0880627	5.46836E-18
6	GeneID:353008	*3.8-1.3*	29878012	29879177	rs1632908	29893403	6.35E-04	5.80E-16	84.89182774	1.59927E-17
6	GeneID:3077	*HFE*	26087509	26095469	rs71557316	26072981	4.78E-08	1.70E-11	83.31414013	3.45585E-17
6	GeneID:3006	*HIST1H1C*	26055968	26056699	rs3752417	26045905	3.96E-08	2.09E-11	83.2737902	3.52462E-17
**Chr**	**Gene.id**	**Name**	**Start**	**End**	**SNP.id**	**SNP.pos**	**pval(headache)**	**pval(hyperPAN)**	**X^2,4df**	**FCP**

6	GeneID:8335	*HIST1H2AB*	26033320	26033796	rs3752417	26045905	3.79E-08	2.75E-11	82.80920329	4.42204E-17
6	GeneID:8352	*HIST1H3C*	26045639	26046097	rs3752417	26045905	3.84E-08	2.78E-11	82.76454322	4.51952E-17
6	GeneID:10341	*HIST1H2APS5*	26044128	26044778	rs3752417	26045905	3.85E-08	2.79E-11	82.752819	4.54546E-17
6	GeneID:8358	*HIST1H3B*	26031817	26032288	rs3752417	26045905	3.87E-08	2.80E-11	82.73338552	4.58879E-17
6	GeneID:8334	*HIST1H2AC*	26124373	26124918	rs13200797	26122957	7.26E-08	1.53E-11	82.67757849	4.71553E-17
6	GeneID:8347	*HIST1H2BC*	26123695	26124132	rs13200797	26122957	7.28E-08	1.54E-11	82.66446467	4.74581E-17
6	GeneID:3018	*HIST1H2BB*	26043455	26043885	rs3752417	26045905	3.94E-08	2.85E-11	82.65893146	4.75865E-17
6	GeneID:8364	*HIST1H4C*	26104176	26104565	rs2237228	26104630	5.85E-08	2.25E-11	82.34548374	5.54546E-17
6	GeneID:10246	*SLC17A2*	25912982	25930946	rs35169013	25918027	4.09E-08	4.02E-11	81.89941815	6.8944E-17
6	GeneID:85303	*HIST1H2APS2*	25882154	25882644	rs13208859	25894609	4.48E-08	3.82E-11	81.81832739	7.17274E-17
6	GeneID:10338	*HIST1H1PS2*	26016335	26017069	rs10484435	26031811	7.15E-08	2.71E-11	81.56924831	8.09989E-17
6	GeneID:3024	*HIST1H1A*	26017260	26018040	rs10484435	26031811	7.42E-08	2.81E-11	81.42316639	8.69843E-17
6	GeneID:8366	*HIST1H4B*	26027124	26027480	rs10484435	26031811	7.48E-08	2.83E-11	81.38956427	8.84224E-17
6	GeneID:8350	*HIST1H3A*	26020718	26021186	rs10484435	26031811	7.65E-08	2.90E-11	81.30187517	9.22884E-17
6	GeneID:8359	*HIST1H4A*	26021907	26022278	rs10484435	26031811	7.66E-08	2.90E-11	81.2960825	9.25496E-17
6	GeneID:10786	*SLC17A3*	25845328	25874471	rs6913795	25848025	5.33E-08	4.74E-11	81.03944528	1.04897E-16
6	GeneID:10475	*TRIM38*	25963071	25985358	rs13203673	25979122	7.55E-08	3.50E-11	80.94834927	1.09665E-16
6	GeneID:6568	*SLC17A1*	25783125	25832287	rs9467607	25809477	3.82E-08	7.67E-11	80.74390135	1.21169E-16
6	GeneID:10050	*SLC17A4*	25754927	25781403	rs13200921	25790378	3.74E-08	1.72E-10	79.16713793	2.61472E-16
6	GeneID:10590	*SCGN*	25652429	25702011	rs35436081	25700342	6.86E-08	1.84E-10	77.8185462	5.04651E-16
6	GeneID:221613	*HIST1H2AA*	25726291	25726790	rs34493019	25714959	1.72E-07	1.65E-10	76.20023435	1.11047E-15
6	GeneID:255626	*HIST1H2BA*	25727137	25727573	rs34493019	25714959	1.75E-07	1.69E-10	76.11882826	1.1554E-15
6	GeneID:353014	*HCG4P9*	29766201	29767903	rs143525179	29751998	7.75E-06	2.00E-11	72.80774839	5.79315E-15
6	GeneID:353016	*HCP5P13*	29760911	29762023	rs143525179	29751998	1.01E-05	2.61E-11	71.74453178	9.71791E-15
6	GeneID:352962	*HLA-V*	29759530	29760527	rs143525179	29751998	1.07E-05	2.75E-11	71.52552693	1.08103E-14
6	GeneID:554223	*LOC554223*	29759683	29765584	rs143525179	29751998	1.07E-05	2.75E-11	71.52303816	1.08234E-14
6	GeneID:54435	*HCG4*	29758808	29760850	rs143525179	29751998	1.07E-05	2.75E-11	71.52221273	1.08277E-14
6	GeneID:100419971	*LOC100419971*	25679007	25679651	rs13191296	25684606	4.57E-07	2.33E-09	68.94718522	3.78629E-14
**Chr**	**Gene.id**	**Name**	**Start**	**End**	**SNP.id**	**SNP.pos**	**pval(headache)**	**pval(hyperPAN)**	**X^2,4df**	**FCP**

6	GeneID:346171	*ZFP57*	29640169	29644931	rs138358844	29659400	7.55E-03	4.07E-13	66.83102448	1.05823E-13
6	GeneID:3010	*HIST1H1T*	26107640	26108364	rs2237228	26104630	8.76E-08	3.99E-08	66.57406268	1.19883E-13
6	GeneID:4340	*MOG*	29624758	29640149	rs62392957	29649219	1.02E-02	4.42E-13	66.0659722	1.53411E-13
6	GeneID:100507584	*LOC100507584*	33857288	33864684	rs73744723	33857439	4.85E-02	7.59E-13	61.8646257	1.17624E-12
6	GeneID:353020	*HCG4P11*	29688955	29689944	rs183103224	29690291	3.60E-03	1.11E-11	61.70464633	1.27101E-12
6	GeneID:6148	*RPL23AP1*	29694409	29694931	rs183103224	29690291	3.65E-03	1.12E-11	61.65489153	1.302E-12
6	GeneID:3134	*HLA-F*	29691117	29695073	rs183103224	29690291	3.65E-03	1.12E-11	61.65172652	1.304E-12
6	GeneID:100048907	*ZDHHC20P1*	29675918	29676324	rs183103224	29690291	2.97E-03	1.71E-11	61.22998425	1.59945E-12
6	GeneID:285830	*HLA-F-AS1*	29694378	29716826	rs183103224	29690291	4.70E-03	1.45E-11	60.6372049	2.13108E-12
6	GeneID:4280	*MICE*	29709234	29716880	rs62391801	29694443	6.49E-03	1.33E-11	60.1629625	2.6809E-12
10	GeneID:5654	*HTRA1*	124221041	124274424	rs2223089	124210160	2.31E-11	2.58E-02	56.29528528	1.73875E-11
6	GeneID:55604	*LRRC16A*	25279656	25620758	rs927985	25412811	2.27E-06	2.79E-07	56.1753489	1.84241E-11
1	GeneID:10806	*SDCCAG8*	243419307	243663393	rs12058508	243487861	2.64E-07	3.37E-06	55.49623039	2.55715E-11
6	GeneID:3008	*HIST1H1E*	26156559	26157343	rs7749823	26158079	1.47E-04	8.96E-08	50.10901034	3.42649E-10
1	GeneID:100422536	*LOC100422536*	243430920	243431421	rs12741781	243428152	7.80E-06	1.99E-06	49.77788835	4.01775E-10
6	GeneID:3122	*HLA-DRA*	32407619	32412823	rs116479312	32408500	2.78E-03	2.64E-08	46.67120197	1.78533E-09
1	GeneID:100616343	*MIR4677*	243509478	243509557	rs10926994	243500994	3.64E-05	2.37E-06	46.3475843	2.08495E-09
1	GeneID:9859	*CEP170*	243287730	243418708	rs12732083	243413125	1.58E-05	6.45E-06	46.0192173	2.44028E-09
6	GeneID:26529	*OR12D2*	29364416	29365448	rs3128852	29364135	4.93E-04	3.57E-07	44.91752903	4.13607E-09
6	GeneID:7407	*VARS*	31745295	31763712	rs2075800	31777946	4.51E-02	4.70E-08	39.94372986	4.44597E-08
14	GeneID:1531	*CYB5AP3*	93604300	93605122	rs28540738	93591673	6.93E-06	6.40E-04	38.46723329	8.9747E-08
6	GeneID:100189411	*TRNAA41*	26705606	26705678	rs2451729	26705859	4.93E-06	2.74E-03	36.23995463	2.58274E-07
6	GeneID:100189303	*TRNAM16*	26701712	26701784	rs2451729	26705859	5.23E-06	2.90E-03	36.00544802	2.88624E-07
6	GeneID:100189128	*TRNAA18*	26687485	26687557	rs9467835	26678512	7.11E-06	2.46E-03	35.72356653	3.29844E-07
6	GeneID:100533655	*LOC100533655*	25218937	25221396	rs76798435	25231495	4.62E-04	4.33E-05	35.45727665	3.74159E-07
6	GeneID:100130756	*UQCRHP1*	31578860	31579133	rs2736188	31565648	1.07E-02	1.95E-06	35.36077443	3.91644E-07
14	GeneID:3705	*ITPK1*	93403259	93582263	rs28540738	93591673	2.32E-05	9.18E-04	35.33010644	3.97369E-07
6	GeneID:100420841	*LOC100420841*	25248417	25249008	rs183779130	25244395	3.69E-04	8.26E-05	34.61250007	5.57944E-07
**Chr**	**Gene.id**	**Name**	**Start**	**End**	**SNP.id**	**SNP.pos**	**pval(headache)**	**pval(hyperPAN)**	**X^2,4df**	**FCP**

6	GeneID:394263	*MUC21*	30951485	30957675	rs141725002	30971976	1.79E-02	1.86E-06	34.44155902	6.04891E-07
6	GeneID:645452	*RPL21P68*	25261427	25261986	rs138739848	25250492	4.38E-04	8.01E-05	34.33318008	6.36675E-07
13	GeneID:23143	*LRCH1*	47127296	47327176	rs7318686	47200504	1.09E-05	3.33E-03	34.26130427	6.58667E-07
6	GeneID:3139	*HLA-L*	30227339	30234728	rs188226252	30236078	2.40E-02	1.67E-06	34.06196691	7.23698E-07
8	GeneID:100420053	*LOC100420053*	10194293	10195166	rs7818437	10209623	2.36E-06	2.20E-02	33.54128818	9.25348E-07
8	GeneID:640	*BLK*	11351521	11422108	rs12549150	11422936	1.65E-05	3.51E-03	33.32911006	1.02277E-06
6	GeneID:100420530	*LOC100420530*	30931992	30933937	rs143764990	30943138	2.56E-02	2.30E-06	33.29265977	1.04051E-06
20	GeneID:140688	*C20orf112*	31030862	31071288	rs1555132	31046567	1.85E-06	3.29E-02	33.22493658	1.07428E-06
6	GeneID:100500885	*MIR3925*	36590213	36590289	rs149829367	36580284	1.33E-04	4.73E-04	33.15946684	1.10796E-06
6	GeneID:100379658	*RNY5P5*	25192641	25192714	rs77042011	25204583	2.26E-03	3.22E-05	32.8687033	1.27074E-06
6	GeneID:221527	*ZBTB12*	31867394	31869769	rs115062572	31862876	1.46E-02	5.00E-06	32.86586676	1.27244E-06
6	GeneID:404024	*TRIM26P1*	30206078	30210056	rs191662147	30211196	2.79E-02	2.85E-06	32.69654586	1.37813E-06

Chr, chromosome number; Gene.id, Gene ID; Name, Gene; Start, start point of the gene; End, end point of the gene; SNP.id, top SNP associated with the gene; SNP.pos, position of the top SNP; pval(headache), p-value of the gene in headache dataset; pval(hyperPAN), p-value of the gene in hyperthyroidism dataset.

Supplementary Table S12. Genes overlapping headache and secondary hypothyroidism at $p < 0.05$ and Fisher combined p-value ($p_{FCP} < 2.09 \times 10^{-6}$).

Chr	Gene.id	Name	Start	End	SNP.id	SNP.pos	pval(headache)	pval(sechypoPAN)	X^2,4df	FCP
17	GeneID:284058	*KIAA1267*	44107282	44302740	rs111535660	44314148	1.71E-15	3.47E-02	74.72508151	2.27804E-15
17	GeneID:9884	*LRRC37A*	44372497	44415160	rs2458218	44361383	6.20E-15	1.36E-02	74.0270603	3.20012E-15
17	GeneID:4905	*NSF*	44668035	44834830	rs1378358	44787312	1.04E-13	4.00E-02	66.22404232	1.42082E-13
17	GeneID:201176	*ARHGAP27*	43471268	43510282	rs35489312	43517252	1.77E-13	4.47E-02	64.9432055	2.64508E-13
6	GeneID:80740	*LY6G6C*	31686425	31689510	rs3131383	31704294	1.48E-07	1.73E-07	62.5971	8.24889E-13
6	GeneID:80739	*C6orf25*	31691121	31694487	rs3101018	31705864	1.77E-07	2.80E-07	61.26969362	1.56899E-12
6	GeneID:23564	*DDAH2*	31694817	31698039	rs3132445	31712196	2.03E-07	3.23E-07	60.70621861	2.06107E-12
6	GeneID:401251	*C6orf26*	31730773	31732627	rs3130484	31715882	2.01E-07	3.42E-07	60.61556497	2.15352E-12
6	GeneID:80737	*C6orf27*	31733371	31745108	rs3117573	31718396	2.15E-07	3.45E-07	60.46501182	2.31631E-12
6	GeneID:1192	*CLIC1*	31698358	31704341	rs3130484	31715882	2.25E-07	3.83E-07	60.17115344	2.67029E-12
**Chr**	**Gene.id**	**Name**	**Start**	**End**	**SNP.id**	**SNP.pos**	**pval(headache)**	**pval(sechypoPAN)**	**X^2,4df**	**FCP**

6	GeneID:721	*C4B*	31982572	32003195	rs433061	32014828	1.16E-07	1.05E-06	59.46533749	3.75722E-12
6	GeneID:100532732	*MSH5-C6orf26*	31707725	31732627	rs3130484	31715882	3.09E-07	5.27E-07	58.89134467	4.95944E-12
6	GeneID:259197	*NCR3*	31556660	31560762	rs1800628	31546850	1.79E-07	9.75E-07	58.75257794	5.30365E-12
6	GeneID:7124	*TNF*	31543350	31546112	rs1800628	31546850	1.86E-07	1.01E-06	58.61139593	5.67834E-12
6	GeneID:4758	*NEU1*	31826829	31830709	rs501942	31840477	5.11E-07	3.83E-07	58.5217947	5.92974E-12
6	GeneID:7940	*LST1*	31553956	31556686	rs1800628	31546850	1.90E-07	1.03E-06	58.51628037	5.94557E-12
6	GeneID:4050	*LTB*	31548335	31550202	rs1800628	31546850	1.92E-07	1.04E-06	58.48373489	6.03986E-12
6	GeneID:80736	*SLC44A4*	31830969	31846823	rs501942	31840477	4.53E-07	5.18E-07	58.1611697	7.05909E-12
6	GeneID:10919	*EHMT2*	31847536	31865464	rs501942	31840477	4.78E-07	5.46E-07	57.95096874	7.814E-12
6	GeneID:100302242	*MIR1236*	31924616	31924717	rs1270942	31918860	1.43E-07	1.91E-06	57.85771558	8.17423E-12
6	GeneID:1589	*CYP21A2*	32006093	32009447	rs433061	32014828	1.77E-07	1.61E-06	57.77062788	8.52561E-12
6	GeneID:7936	*RDBP*	31919864	31926864	rs1270942	31918860	1.48E-07	1.98E-06	57.71630901	8.75237E-12
6	GeneID:6499	*SKIV2L*	31926581	31937532	rs1270942	31918860	1.75E-07	2.34E-06	57.04567608	1.21017E-11
6	GeneID:4049	*LTA*	31539876	31542098	rs1800628	31546850	1.76E-07	2.34E-06	57.03614527	1.21576E-11
6	GeneID:629	*CFB*	31913721	31919861	rs1270942	31918860	1.76E-07	2.35E-06	57.02283419	1.2236E-11
6	GeneID:1388	*ATF6B*	32083045	32096017	rs1269852	32080191	1.64E-07	2.67E-06	56.91287667	1.29035E-11
6	GeneID:5491	*PPIAP9*	31486754	31488108	rs3130614	31476458	2.59E-07	3.08E-06	55.71192029	2.30434E-11
6	GeneID:100287329	*LOC100287329*	31527348	31540474	rs1800628	31546850	2.48E-07	3.30E-06	55.66257193	2.35988E-11
6	GeneID:717	*C2*	31868776	31913449	rs1270942	31918860	2.79E-07	3.02E-06	55.60217351	2.42969E-11
6	GeneID:7148	*TNXB*	32008932	32077151	rs1269852	32080191	3.04E-07	3.63E-06	55.06531805	3.14819E-11
6	GeneID:4277	*MICB*	31465855	31478901	rs3130614	31476458	4.11E-07	4.90E-06	53.85957951	5.6313E-11
6	GeneID:692233	*SNORD117*	31504151	31504226	rs9267488	31514247	6.99E-07	3.03E-06	53.76008652	5.90798E-11
6	GeneID:692199	*SNORD84*	31508878	31508955	rs9267488	31514247	8.34E-07	3.62E-06	53.05323501	8.30595E-11
6	GeneID:534	*ATP6V1G2*	31512228	31514625	rs9267488	31514247	8.95E-07	3.88E-06	52.77037277	9.51864E-11
6	GeneID:100532737	*ATP6V1G2-DDX39B*	31497996	31514625	rs9267488	31514247	1.00E-06	4.34E-06	52.32312082	1.18068E-10
6	GeneID:4795	*NFKBIL1*	31514628	31526606	rs9267488	31514247	1.10E-06	4.76E-06	51.95147438	1.41206E-10
1	GeneID:79630	*C1orf54*	150245183	150253335	rs12740679	150262270	2.46E-10	4.79E-02	50.32890257	3.08269E-10
6	GeneID:3106	*HLA-B*	31321649	31324989	rs2523593	31326703	1.14E-06	2.11E-05	48.89924553	6.12837E-10
**Chr**	**Gene.id**	**Name**	**Start**	**End**	**SNP.id**	**SNP.pos**	**pval(headache)**	**pval(sechypoPAN)**	**X^2,4df**	**FCP**

6	GeneID:729816	*DHFRP2*	31331244	31334742	rs2523593	31326703	1.22E-06	2.25E-05	48.64670798	6.91867E-10
6	GeneID:100131609	*HNRNPA1P2*	32293175	32294298	rs116667074	32285362	7.28E-06	4.40E-06	48.32764475	8.06421E-10
6	GeneID:29113	*C6orf15*	31079000	31080332	rs2233980	31079644	9.56E-07	7.97E-05	46.59576464	1.85109E-09
6	GeneID:1041	*CDSN*	31082865	31088252	rs2233980	31079644	1.09E-06	9.12E-05	46.05606529	2.39757E-09
6	GeneID:10665	*C6orf10*	32260475	32339656	rs116667074	32285362	1.46E-05	8.85E-06	45.53315264	3.08012E-09
6	GeneID:170679	*PSORS1C1*	31082608	31107869	rs2233980	31079644	1.44E-06	1.20E-04	44.96212214	4.04871E-09
6	GeneID:6890	*TAP1*	32812986	32821748	rs45506201	32808299	9.10E-06	2.41E-05	44.48373359	5.09039E-09
6	GeneID:100507463	*LOC100507463*	32811863	32814277	rs45506201	32808299	9.66E-06	2.55E-05	44.24613576	5.70321E-09
6	GeneID:5696	*PSMB8*	32808494	32812712	rs45506201	32808299	1.03E-05	2.74E-05	43.9705589	6.50675E-09
6	GeneID:100507444	*PPP1R2P1*	32844255	32847851	rs115591082	32844103	5.24E-06	6.14E-05	43.71346139	7.35795E-09
6	GeneID:3119	*HLA-DQB1*	32627657	32634466	rs1130389	32632627	1.09E-05	3.08E-05	43.62341611	7.68163E-09
6	GeneID:170680	*PSORS1C2*	31105311	31107127	rs3130557	31094703	2.78E-06	1.38E-04	43.36371705	8.69695E-09
6	GeneID:6891	*TAP2*	32789610	32806547	rs45506201	32808299	1.35E-05	3.57E-05	42.90182117	1.08448E-08
6	GeneID:5698	*PSMB9*	32821938	32827628	rs45506201	32808299	5.28E-06	9.67E-05	42.79200331	1.14289E-08
6	GeneID:54535	*CCHCR1*	31110216	31126015	rs3132541	31098734	4.22E-06	1.36E-04	42.55210546	1.28164E-08
3	GeneID:100506815	*FOXP1-IT1*	71619425	71623608	rs62244889	71609007	1.04E-05	6.00E-05	42.39759297	1.37978E-08
6	GeneID:3117	*HLA-DQA1*	32605183	32611429	rs9272729	32609594	4.50E-05	3.70E-05	40.42936436	3.52786E-08
15	GeneID:9728	*SECISBP2L*	49280835	49338760	rs7183756	49307027	6.84E-05	2.84E-05	40.11933362	4.0893E-08
6	GeneID:3127	*HLA-DRB5*	32485151	32498006	rs115250958	32509842	3.77E-05	5.84E-05	39.86610885	4.61336E-08
6	GeneID:3128	*HLA-DRB6*	32520490	32527779	rs115250958	32509842	3.81E-05	5.91E-05	39.8226597	4.70978E-08
6	GeneID:100129192	*MICC*	30382490	30387543	rs149543464	30400763	2.63E-07	1.01E-02	39.49344184	5.50881E-08
3	GeneID:27086	*FOXP1*	71004736	71633140	rs7610856	71579022	5.40E-06	7.01E-04	38.78409886	7.71971E-08
15	GeneID:2252	*FGF7*	49715375	49779523	rs16962486	49743582	4.06E-03	2.25E-06	37.02335924	1.78145E-07
4	GeneID:4299	*AFF1*	87856154	88062206	rs342458	88060025	3.17E-07	4.26E-02	36.24127135	2.58113E-07
17	GeneID:6155	*RPL27*	41150446	41154976	rs2089858	41156153	2.73E-06	1.36E-02	34.21224471	6.7411E-07
10	GeneID:100616320	*MIR4481*	12695137	12695196	rs7072694	12692577	1.08E-05	3.62E-03	34.11471312	7.05892E-07
17	GeneID:146923	*RUNDC1*	41132582	41145707	rs2089858	41156153	3.12E-06	1.52E-02	33.73277974	8.45389E-07
15	GeneID:196951	*C15orf33*	49620590	49913118	rs16962486	49743582	8.57E-03	5.55E-06	33.72073259	8.5021E-07
**Chr**	**Gene.id**	**Name**	**Start**	**End**	**SNP.id**	**SNP.pos**	**pval(headache)**	**pval(sechypoPAN)**	**X^2,4df**	**FCP**

15	GeneID:9318	*COPS2*	49417471	49447854	rs12439497	49406214	2.00E-03	2.39E-05	33.71164819	8.53864E-07
6	GeneID:352996	*P5-04*	29910314	29910426	rs2524005	29899677	3.23E-04	1.62E-04	33.53630311	9.27527E-07
6	GeneID:3105	*HLA-A*	29910247	29913661	rs2524005	29899677	3.23E-04	1.62E-04	33.53171296	9.29538E-07
6	GeneID:352994	*P5-05*	29894219	29894350	rs2524005	29899677	3.24E-04	1.62E-04	33.52185146	9.33873E-07
20	GeneID:284804	*LOC284804*	31136309	31173628	rs6058750	31177986	3.45E-06	1.53E-02	33.51135246	9.38511E-07
6	GeneID:80868	*HCG4B*	29892369	29893428	rs2524005	29899677	3.31E-04	1.66E-04	33.43076154	9.74884E-07
6	GeneID:3138	*HLA-K*	29894436	29897616	rs2524005	29899677	3.34E-04	1.67E-04	33.40071383	9.88802E-07
6	GeneID:353003	*HCG4P5*	29908689	29909578	rs2524005	29899677	3.36E-04	1.69E-04	33.36972627	1.00336E-06
6	GeneID:352965	*HLA-U*	29901541	29902657	rs2524005	29899677	3.42E-04	1.71E-04	33.30437719	1.03477E-06
6	GeneID:352995	*P5.8*	29903015	29904157	rs2524005	29899677	3.59E-04	1.80E-04	33.10892393	1.13469E-06
15	GeneID:735247	*LOC735247*	49657062	49659024	rs8034661	49646799	6.74E-03	1.11E-05	32.8118833	1.30523E-06
15	GeneID:100420615	*LOC100420615*	49657122	49659024	rs8034661	49646799	6.74E-03	1.11E-05	32.8118833	1.30523E-06
1	GeneID:51107	*APH1A*	150237799	150241532	rs10157197	150250636	2.05E-06	3.72E-02	32.78310192	1.32306E-06
1	GeneID:23632	*CA14*	150230218	150237478	rs10157197	150250636	2.36E-06	4.29E-02	32.21430387	1.72954E-06

Chr, chromosome number; Gene.id, Gene ID; Name, Gene; Start, start point of the gene; End, end point of the gene; SNP.id, top SNP associated with the gene; SNP.pos, position of the top SNP; pval(headache), p-value of the gene in headache dataset; pval(sechypoPAN), p-value of the gene in secondary hypothyroidism dataset.

Supplementary Table S13. Genes overlapping headache and TSH at $p < 0.05$ and Fisher combined p-value ($p_{FCP} < 2.09 \times 10^{-6}$).

Chr	Gene.id	Name	Start	End	SNP.id	SNP.pos	pval(headache)	pval(TSH)	X^2,4df	FCP
6	GeneID:10846	*PDE10A*	165740778	166075584	rs73786673	166033316	3.29E-02	2.46E-50	235.2866071	9.60297E-50
15	GeneID:2252	*FGF7*	49715375	49779523	rs16962486	49743582	2.83E-03	1.12E-31	154.2660384	2.47965E-32
15	GeneID:196951	*C15orf33*	49620590	49913118	rs16962486	49743582	5.93E-03	2.74E-31	150.9993818	1.24326E-31
15	GeneID:9728	*SECISBP2L*	49280835	49338760	rs7183756	49307027	4.67E-05	5.37E-22	117.8977981	1.50176E-24
15	GeneID:729344	*KRT8P24*	49263922	49265761	rs4775791	49278326	3.78E-04	2.05E-20	106.4323048	4.19418E-22
15	GeneID:735247	*LOC735247*	49657062	49659024	rs8034661	49646799	5.14E-03	2.82E-21	105.1735668	7.77871E-22
15	GeneID:100420615	*LOC100420615*	49657122	49659024	rs8034661	49646799	5.14E-03	2.82E-21	105.1735668	7.77871E-22
15	GeneID:9318	*COPS2*	49417471	49447854	rs12439497	49406214	1.39E-03	1.16E-20	104.9546273	8.66088E-22
**Chr**	**Gene.id**	**Name**	**Start**	**End**	**SNP.id**	**SNP.pos**	**pval(headache)**	**pval(TSH)**	**X^2,4df**	**FCP**

15	GeneID:56986	*DTWD1*	49913226	49937333	rs56057624	49922325	1.01E-02	1.66E-21	104.8882701	8.9475E-22
15	GeneID:100131797	*RPL15P19*	49469498	49470980	rs34854443	49475296	3.95E-03	4.36E-21	104.8304929	9.20477E-22
15	GeneID:100616332	*MIR4716*	49461267	49461350	rs34854443	49475296	3.14E-03	6.44E-21	104.5126072	1.07584E-21
15	GeneID:100306975	*LOC100306975*	49448495	49450822	rs563533	49465581	3.82E-03	6.56E-21	104.081803	1.32903E-21
15	GeneID:2585	*GALK2*	49447976	49622002	rs11631782	49542284	4.03E-03	8.40E-21	103.4783903	1.78684E-21
17	GeneID:644315	*RPS7P11*	44798914	44799606	rs1378358	44787312	1.83E-14	8.36E-09	100.4670426	7.82372E-21
17	GeneID:4905	*NSF*	44668035	44834830	rs1378358	44787312	3.50E-14	9.98E-09	98.81355071	1.75954E-20
11	GeneID:56981	*PRDM11*	45115564	45246903	rs78469981	45185735	2.08E-02	2.34E-20	98.14788749	2.4382E-20
14	GeneID:3705	*ITPK1*	93403259	93582263	rs28540738	93591673	1.84E-05	3.97E-16	92.72876498	3.46463E-19
17	GeneID:401884	*MGC57346*	43697712	43715329	rs117368197	43715924	1.24E-16	6.56E-05	92.51092741	3.85442E-19
17	GeneID:147081	*C17orf69*	43716341	43723595	rs117368197	43715924	1.24E-16	6.56E-05	92.51092741	3.85442E-19
17	GeneID:246744	*STH*	44076616	44077060	rs112572874	44072984	1.62E-16	7.01E-05	91.85464391	5.31427E-19
17	GeneID:100130148	*LOC100130148*	43973149	43976164	rs112275277	43981958	2.87E-16	4.46E-05	91.61190421	5.98451E-19
17	GeneID:4137	*MAPT*	43971748	44105700	rs62062277	44093753	2.43E-16	8.77E-05	90.59345123	9.84991E-19
17	GeneID:284058	*KIAA1267*	44107282	44302740	rs62062277	44093753	2.24E-16	9.99E-05	90.49243741	1.03489E-18
17	GeneID:7473	*WNT3*	44841686	44896082	rs199533	44828931	3.93E-13	7.19E-08	90.02686982	1.29957E-18
17	GeneID:100128977	*LOC100128977*	43920722	43972879	rs112275277	43981958	9.85E-16	5.78E-05	88.62489673	2.57968E-18
17	GeneID:162540	*IMP5*	43922256	43924438	rs878888	43912635	1.34E-15	4.94E-05	88.32273922	2.99039E-18
17	GeneID:1394	*CRHR1*	43697710	43913194	rs111273167	43740967	2.27E-15	3.26E-05	88.10222892	3.33081E-18
17	GeneID:100423004	*MIR4315-1*	43552729	43552801	rs148269941	43540472	5.42E-15	6.95E-05	84.84560381	1.6358E-17
17	GeneID:9842	*PLEKHM1*	43513266	43568146	rs148269941	43540472	1.11E-14	9.57E-05	82.76513729	4.51821E-17
14	GeneID:1531	*CYB5AP3*	93604300	93605122	rs28540738	93591673	5.50E-06	3.62E-13	81.51247718	8.32745E-17
17	GeneID:201176	*ARHGAP27*	43471268	43510282	rs35489312	43517252	3.11E-14	1.37E-04	79.9938864	1.74703E-16
11	GeneID:57586	*SYT13*	45261853	45307884	rs11038372	45282994	1.51E-02	2.79E-13	66.19976244	1.43766E-13
10	GeneID:387715	*ARMS2*	124214179	124216868	rs78438709	124201071	2.71E-12	3.27E-02	60.10353999	2.75911E-12
6	GeneID:29113	*C6orf15*	31079000	31080332	rs2233980	31079644	7.86E-07	3.54E-07	57.82184213	8.31718E-12
6	GeneID:1041	*CDSN*	31082865	31088252	rs2233980	31079644	9.39E-07	4.22E-07	57.11250931	1.17172E-11
6	GeneID:170679	*PSORS1C1*	31082608	31107869	rs2233980	31079644	1.22E-06	5.50E-07	56.05473385	1.95289E-11
**Chr**	**Gene.id**	**Name**	**Start**	**End**	**SNP.id**	**SNP.pos**	**pval(headache)**	**pval(TSH)**	**X^2,4df**	**FCP**

6	GeneID:170680	*PSORS1C2*	31105311	31107127	rs3130557	31094703	2.47E-06	8.22E-07	53.84210065	5.67895E-11
11	GeneID:219541	*MED19*	57471186	57479673	rs143262971	57468415	1.04E-09	3.10E-03	52.9209336	8.85265E-11
6	GeneID:54535	*CCHCR1*	31110216	31126015	rs3132541	31098734	3.43E-06	1.15E-06	52.51968354	1.07404E-10
11	GeneID:25921	*ZDHHC5*	57435474	57468659	rs143262971	57468415	1.72E-09	3.24E-03	51.8183309	1.50554E-10
15	GeneID:399694	*SHC4*	49115934	49255641	rs12906122	49257201	2.25E-03	3.42E-09	51.18503707	2.04207E-10
9	GeneID:100128505	*LOC100128505*	119266562	119324572	rs6478241	119252629	1.27E-09	1.18E-02	49.83221558	3.91419E-10
10	GeneID:338591	*LOC338591*	8661896	8662573	rs72777033	8652323	7.55E-04	2.04E-08	49.78789064	3.99848E-10
11	GeneID:100528016	*TMX2-CTNND1*	57479995	57586652	rs143262971	57468415	2.73E-09	5.85E-03	49.7234332	4.12431E-10
6	GeneID:3107	*HLA-C*	31236529	31239855	rs2524107	31232590	2.21E-02	2.55E-09	47.19468559	1.38897E-09
6	GeneID:80740	*LY6G6C*	31686425	31689510	rs3131383	31704294	3.74E-08	1.66E-03	47.00821377	1.51892E-09
6	GeneID:23564	*DDAH2*	31694817	31698039	rs3132445	31712196	1.05E-07	7.98E-04	46.4115346	2.022E-09
9	GeneID:169792	*GLIS3*	3824128	4300035	rs118047658	3860001	2.60E-02	3.38E-09	46.31381309	2.11897E-09
6	GeneID:1192	*CLIC1*	31698358	31704341	rs3130484	31715882	1.12E-07	9.50E-04	45.92913501	2.54791E-09
6	GeneID:80739	*C6orf25*	31691121	31694487	rs3101018	31705864	8.54E-08	2.15E-03	44.83529681	4.30212E-09
6	GeneID:80737	*C6orf27*	31733371	31745108	rs3117573	31718396	1.32E-07	1.47E-03	44.72357438	4.53843E-09
6	GeneID:401251	*C6orf26*	31730773	31732627	rs3130484	31715882	1.31E-07	1.56E-03	44.61912007	4.77106E-09
6	GeneID:100532732	*MSH5-C6orf26*	31707725	31732627	rs3130484	31715882	1.72E-07	1.46E-03	44.21348139	5.793E-09
6	GeneID:100419608	*LOC100419608*	28083427	28083998	rs66886492	28089731	1.52E-08	2.45E-02	43.41856723	8.47191E-09
17	GeneID:113026	*PLCD3*	43189008	43209891	rs58219619	43207624	7.54E-05	4.96E-06	43.4131621	8.49383E-09
6	GeneID:221584	*ZSCAN12P1*	28058929	28063493	rs35952432	28074901	2.42E-08	1.68E-02	43.25353044	9.16722E-09
6	GeneID:7746	*ZNF193*	28193029	28201265	rs17720293	28214698	1.49E-08	3.48E-02	42.76912811	1.15545E-08
6	GeneID:80345	*ZSCAN16*	28092387	28097857	rs66886492	28089731	2.04E-08	2.69E-02	42.65281655	1.22145E-08
6	GeneID:629	*CFB*	31913721	31919861	rs1270942	31918860	1.05E-07	5.41E-03	42.56882644	1.27145E-08
6	GeneID:100129195	*LOC100129195*	28058336	28105071	rs35749575	28114818	2.49E-08	2.32E-02	42.54234545	1.28763E-08
6	GeneID:100302242	*MIR1236*	31924616	31924717	rs1270942	31918860	8.76E-08	6.70E-03	42.51331449	1.3056E-08
1	GeneID:100418820	*PHBP12*	51246012	51246821	rs1416685	51243374	8.72E-05	7.00E-06	42.43247839	1.35698E-08
6	GeneID:717	*C2*	31868776	31913449	rs1270942	31918860	1.64E-07	4.23E-03	42.17444758	1.53489E-08
6	GeneID:7936	*RDBP*	31919864	31926864	rs1270942	31918860	9.83E-08	7.52E-03	42.05040786	1.62851E-08
**Chr**	**Gene.id**	**Name**	**Start**	**End**	**SNP.id**	**SNP.pos**	**pval(headache)**	**pval(TSH)**	**X^2,4df**	**FCP**

6	GeneID:100189159	*TRNAS13*	28180815	28180896	rs13197633	28174757	2.39E-08	3.28E-02	41.93350617	1.72195E-08
6	GeneID:222701	*ZNF192P2*	28155716	28157189	rs13195291	28169241	2.71E-08	2.99E-02	41.86609677	1.77824E-08
6	GeneID:442179	*OR1F12*	28041026	28042002	rs71559070	28038929	4.14E-08	2.07E-02	41.75764003	1.8727E-08
6	GeneID:222699	*TOB2P1*	28183116	28186707	rs13197633	28174757	2.53E-08	3.49E-02	41.69467954	1.92981E-08
6	GeneID:7718	*ZNF165*	28048482	28057341	rs34166054	28065801	4.18E-08	2.12E-02	41.68570132	1.93809E-08
6	GeneID:100507241	*LOC100507241*	28046570	28048908	rs35902873	28058949	4.31E-08	2.18E-02	41.56912387	2.04894E-08
6	GeneID:1388	*ATF6B*	32083045	32096017	rs1269852	32080191	8.71E-08	1.20E-02	41.36096822	2.26281E-08
1	GeneID:359781	*MRPS6P2*	51312141	51312514	rs6684901	51298828	1.90E-04	5.70E-06	41.28412951	2.34727E-08
6	GeneID:7745	*ZNF192*	28109716	28125236	rs35749575	28114818	2.60E-08	4.53E-02	41.11556892	2.54373E-08
6	GeneID:721	*C4B*	31982572	32003195	rs433061	32014828	7.97E-08	1.50E-02	41.09084975	2.57389E-08
6	GeneID:6499	*SKIV2L*	31926581	31937532	rs1270942	31918860	1.25E-07	9.57E-03	41.08797556	2.57742E-08
2	GeneID:729582	*DIRC3*	218148746	218621316	rs7594886	218618815	3.70E-02	3.35E-08	41.02105498	2.66098E-08
6	GeneID:100287272	*USP8P1*	31243351	31246528	rs2524107	31232590	2.00E-02	7.00E-08	40.77350025	2.99428E-08
6	GeneID:100507444	*PPP1R2P1*	32844255	32847851	rs115591082	32844103	3.21E-06	4.57E-04	40.67900632	3.13221E-08
6	GeneID:7741	*ZNF187*	28234788	28245981	rs13208096	28225311	4.30E-08	3.69E-02	40.52395067	3.3724E-08
6	GeneID:6890	*TAP1*	32812986	32821748	rs45506201	32808299	7.54E-06	2.45E-04	40.21887975	3.89994E-08
6	GeneID:100507463	*LOC100507463*	32811863	32814277	rs45506201	32808299	8.22E-06	2.67E-04	39.87537493	4.59305E-08
6	GeneID:4758	*NEU1*	31826829	31830709	rs501942	31840477	4.13E-07	5.37E-03	39.85533446	4.63708E-08
6	GeneID:80736	*SLC44A4*	31830969	31846823	rs501942	31840477	3.38E-07	6.70E-03	39.80969225	4.73895E-08
6	GeneID:10919	*EHMT2*	31847536	31865464	rs501942	31840477	3.44E-07	6.82E-03	39.74017229	4.89841E-08
3	GeneID:100302112	*MIR1284*	71591121	71591240	rs7610856	71579022	1.79E-07	1.37E-02	39.64851165	5.11687E-08
6	GeneID:5696	*PSMB8*	32808494	32812712	rs45506201	32808299	9.03E-06	2.93E-04	39.49725036	5.49883E-08
1	GeneID:11124	*FAF1*	50906935	51425936	rs1278519	50897342	1.16E-04	2.37E-05	39.430244	5.677E-08
6	GeneID:1589	*CYP21A2*	32006093	32009447	rs433061	32014828	1.23E-07	2.31E-02	39.35754551	5.87682E-08
11	GeneID:710	*SERPING1*	57365027	57382326	rs184391280	57350423	1.56E-06	1.90E-03	39.27868983	6.1015E-08
6	GeneID:7148	*TNXB*	32008932	32077151	rs1269852	32080191	1.80E-07	1.93E-02	38.95641287	7.11237E-08
17	GeneID:79877	*DCAKD*	43100706	43138473	rs74656108	43086231	1.32E-03	3.10E-06	38.63001782	8.30645E-08
6	GeneID:100287329	*LOC100287329*	31527348	31540474	rs1800628	31546850	1.93E-07	2.15E-02	38.59553227	8.44374E-08
**Chr**	**Gene.id**	**Name**	**Start**	**End**	**SNP.id**	**SNP.pos**	**pval(headache)**	**pval(TSH)**	**X^2,4df**	**FCP**

11	GeneID:10978	*CLP1*	57425216	57429337	rs527528	57433327	3.03E-06	1.53E-03	38.37238471	9.38852E-08
6	GeneID:6891	*TAP2*	32789610	32806547	rs45506201	32808299	1.21E-05	3.93E-04	38.33023098	9.57848E-08
6	GeneID:259197	*NCR3*	31556660	31560762	rs1800628	31546850	1.47E-07	3.50E-02	38.16953122	1.03385E-07
17	GeneID:4836	*NMT1*	43138680	43186384	rs7207047	43198998	1.43E-03	3.69E-06	38.12702754	1.05494E-07
6	GeneID:5698	*PSMB9*	32821938	32827628	rs45506201	32808299	4.07E-06	1.35E-03	38.04069866	1.0991E-07
11	GeneID:280636	*C11orf31*	57508722	57510883	rs9420	57510294	2.00E-06	2.76E-03	38.03387168	1.10267E-07
17	GeneID:6155	*RPL27*	41150446	41154976	rs2089858	41156153	7.29E-07	7.84E-03	37.96122104	1.14139E-07
11	GeneID:100507231	*LOC100507231*	57485518	57486002	rs547891	57499065	2.07E-06	2.77E-03	37.95532371	1.14459E-07
11	GeneID:406919	*MIR130A*	57408671	57408759	rs10750866	57404779	5.86E-06	1.02E-03	37.86650052	1.19391E-07
6	GeneID:7124	*TNF*	31543350	31546112	rs1800628	31546850	1.61E-07	3.83E-02	37.80862057	1.22718E-07
11	GeneID:219539	*YPEL4*	57412560	57417417	rs10750866	57404779	5.81E-06	1.12E-03	37.69432442	1.29563E-07
11	GeneID:100507106	*LOC100507106*	57405850	57420606	rs527528	57433327	4.81E-06	1.38E-03	37.66378816	1.31455E-07
6	GeneID:7940	*LST1*	31553956	31556686	rs1800628	31546850	1.67E-07	3.99E-02	37.65178406	1.32206E-07
6	GeneID:4050	*LTB*	31548335	31550202	rs1800628	31546850	1.74E-07	4.15E-02	37.49319148	1.42544E-07
1	GeneID:100422535	*LOC100422535*	50871104	50871696	rs2484680	50868417	6.30E-06	1.16E-03	37.46137369	1.44713E-07
17	GeneID:79777	*ACBD4*	43209967	43221543	rs58219619	43207624	5.16E-05	1.49E-04	37.37124022	1.51038E-07
12	GeneID:10019	*SH2B3*	111843752	111889427	rs3184504	111884608	1.41E-03	5.44E-06	37.3674985	1.51307E-07
17	GeneID:146923	*RUNDC1*	41132582	41145707	rs2089858	41156153	8.51E-07	9.16E-03	37.33799659	1.5344E-07
14	GeneID:7043	*TGFB3*	76424442	76448092	rs146047341	76448921	1.17E-06	6.76E-03	37.31433468	1.55173E-07
6	GeneID:692233	*SNORD117*	31504151	31504226	rs9267488	31514247	4.78E-07	1.78E-02	37.16944879	1.66217E-07
1	GeneID:100129919	*HMGB1P45*	50864534	50865042	rs2484680	50868417	6.78E-06	1.25E-03	37.16503664	1.66565E-07
12	GeneID:10961	*ERP29*	112451152	112461024	rs4767296	112463366	2.46E-04	3.71E-05	37.02279977	1.78193E-07
17	GeneID:3430	*IFI35*	41158742	41166476	rs2089858	41156153	1.08E-06	8.62E-03	36.98431569	1.81475E-07
6	GeneID:692199	*SNORD84*	31508878	31508955	rs9267488	31514247	5.97E-07	1.56E-02	36.98331527	1.81562E-07
12	GeneID:724085	*LOC724085*	112437040	112438873	rs7300252	112430346	3.43E-04	3.13E-05	36.69966729	2.07704E-07
6	GeneID:534	*ATP6V1G2*	31512228	31514625	rs9267488	31514247	6.60E-07	1.72E-02	36.58858816	2.18936E-07
11	GeneID:643376	*BTBD18*	57510986	57519253	rs9420	57510294	2.94E-06	4.06E-03	36.48535939	2.29917E-07
6	GeneID:100532737	*ATP6V1G2-DDX39B*	31497996	31514625	rs9267488	31514247	7.07E-07	1.84E-02	36.31557278	2.49183E-07
**Chr**	**Gene.id**	**Name**	**Start**	**End**	**SNP.id**	**SNP.pos**	**pval(headache)**	**pval(TSH)**	**X^2,4df**	**FCP**

11	GeneID:81210	*OR5BA1P*	57633774	57634752	rs11229148	57621460	4.96E-06	2.79E-03	36.1911953	2.64311E-07
17	GeneID:10493	*VAT1*	41166622	41174459	rs2089858	41156153	1.32E-06	1.13E-02	36.03794999	2.84214E-07
12	GeneID:100101246	*LOC100101246*	112019098	112019749	rs653178	112007756	2.27E-03	6.85E-06	35.95705809	2.95316E-07
6	GeneID:4795	*NFKBIL1*	31514628	31526606	rs9267488	31514247	8.11E-07	2.11E-02	35.76577935	3.23317E-07
1	GeneID:63950	*DMRTA2*	50883223	50889119	rs2484680	50868417	6.71E-06	2.60E-03	35.72685827	3.2933E-07
20	GeneID:284805	*C20orf203*	31219427	31239783	rs750396	31218729	1.59E-06	1.10E-02	35.72507123	3.29609E-07
1	GeneID:1031	*CDKN2C*	51434367	51440309	rs12567744	51452563	1.71E-02	1.38E-06	35.13146434	4.36527E-07
12	GeneID:100500889	*MIR3657*	112475403	112475519	rs4767296	112463366	1.27E-04	1.94E-04	35.0350818	4.5689E-07
11	GeneID:1500	*CTNND1*	57529234	57586652	rs3781885	57570680	5.95E-06	4.31E-03	34.96115429	4.73148E-07
12	GeneID:89894	*TMEM116*	112369086	112451023	rs4767296	112463366	3.92E-04	6.92E-05	34.84510003	4.99842E-07
8	GeneID:286043	*MRPS18CP2*	8791228	8791520	rs878504	8783506	9.79E-07	2.82E-02	34.81208989	5.07705E-07
17	GeneID:10614	*HEXIM1*	43224684	43229468	rs1056619	43209727	3.90E-05	7.89E-04	34.59160823	5.63481E-07
17	GeneID:80755	*AARSD1*	41102543	41132545	rs323500	41140545	4.07E-06	8.75E-03	34.30141477	6.46303E-07
17	GeneID:22834	*ZNF652*	47366568	47439835	rs7209400	47450057	1.03E-02	4.01E-06	33.99879491	7.45613E-07
12	GeneID:6311	*ATXN2*	111890018	112037480	rs3184504	111884608	2.46E-03	1.71E-05	33.9643456	7.57841E-07
14	GeneID:112752	*IFT43*	76452096	76550092	rs146047341	76448921	3.43E-06	2.26E-02	32.75035247	1.34363E-06
17	GeneID:8153	*RND2*	41177258	41184058	rs455055	41167957	7.28E-06	1.12E-02	32.6439699	1.4127E-06
19	GeneID:23403	*FBXO46*	46213887	46234151	rs7256524	46224654	1.40E-04	6.21E-04	32.51543131	1.50088E-06
1	GeneID:2582	*GALE*	24122089	24127294	rs12041159	24127660	6.68E-05	1.31E-03	32.50465845	1.50852E-06
3	GeneID:100506815	*FOXP1-IT1*	71619425	71623608	rs62244889	71609007	5.07E-06	1.73E-02	32.4944075	1.51582E-06
1	GeneID:11313	*LYPLA2*	24117646	24122029	rs12041159	24127660	7.11E-05	1.40E-03	32.25077364	1.7001E-06
1	GeneID:100509213	*LOC100509213*	24114238	24120044	rs12041159	24127660	7.23E-05	1.42E-03	32.18375618	1.75459E-06
12	GeneID:654427	*PCNPP1*	112104737	112108183	rs10849966	112100773	4.00E-04	2.74E-04	32.05396611	1.86512E-06
12	GeneID:642580	*LOC642580*	111819590	111823484	rs10849943	111819589	1.42E-03	8.36E-05	31.90057235	2.00473E-06
17	GeneID:100271450	*RPL21P124*	47477791	47478162	rs2233659	47492587	1.01E-04	1.19E-03	31.8665365	2.03709E-06
17	GeneID:124871	*FLJ40194*	47325605	47336027	rs9893777	47336686	2.29E-02	5.40E-06	31.81273715	2.08931E-06

Chr, chromosome number; Gene.id, Gene ID; Name, Gene; Start, start point of the gene; End, end point of the gene; SNP.id, top SNP associated with the gene; SNP.pos, position of the top SNP; pval(headache), p-value of the gene in headache dataset; pval(TSH), p-value of the gene in TSH dataset.

Supplementary Table S14. Genes overlapping headache and fT4 at $p < 0.05$ and Fisher combined p-value ($p_{FCP} < 2.09 \times 10^{-6}$).

Chr	Gene.id	Name	Start	End	SNP.id	SNP.pos	pval(headache)	pval(fT4)	X^2,4df	FCP
1	GeneID:115353	*LRRC42*	54412037	54433839	rs12094064	54445959	2.93E-02	1.82E-90	420.3293134	1.12531E-89
1	GeneID:100421395	*LOC100421395*	54440851	54441704	rs12094064	54445959	2.57E-02	1.15E-68	320.2031427	4.74101E-68
1	GeneID:9528	*TMEM59*	54497349	54519111	rs114621143	54530630	3.12E-02	1.50E-42	199.5393416	4.71968E-42
1	GeneID:100652969	*LOC100652969*	54500068	54500521	rs75070374	54508231	3.69E-02	4.27E-40	187.9029098	1.4959E-39
1	GeneID:127428	*TCEANC2*	54519274	54565416	rs114540611	54574645	1.37E-02	5.33E-32	152.5987302	5.64654E-32
1	GeneID:100507586	*LOC100507586*	54555529	54555771	rs79659235	54548012	1.63E-02	1.10E-31	150.8045374	1.36873E-31
4	GeneID:51166	*AADAT*	170981373	171011372	rs11731427	170991011	3.12E-05	6.53E-23	122.9168955	1.27212E-25
1	GeneID:100616315	*MIR4781*	54519752	54519827	rs114621143	54530630	2.05E-02	4.39E-25	119.9480946	5.47958E-25
4	GeneID:9848	*MFAP3L*	170907748	170947429	rs7676821	170961647	5.82E-05	2.76E-21	114.1819348	9.3286E-24
4	GeneID:100506085	*LOC100506085*	170838912	170897053	rs112036152	170886594	7.90E-04	7.62E-18	93.11885282	2.86243E-19
12	GeneID:390332	*LOC390332*	57375021	57379744	rs4759042	57377347	8.69E-18	5.46E-03	88.98869812	2.15927E-18
12	GeneID:100420982	*LOC100420982*	57332234	57335266	rs840161	57323523	1.55E-17	1.89E-02	85.34843533	1.27952E-17
12	GeneID:121214	*SDR9C7*	57316938	57328189	rs840161	57323523	1.78E-17	2.16E-02	84.80663595	1.66723E-17
12	GeneID:8608	*RDH16*	57345215	57351418	rs725957	57331741	6.34E-17	8.82E-03	84.05492975	2.40685E-17
6	GeneID:10050	*SLC17A4*	25754927	25781403	rs13200921	25790378	2.72E-08	1.68E-10	79.85585195	1.86871E-16
6	GeneID:6568	*SLC17A1*	25783125	25832287	rs9467607	25809477	2.89E-08	1.87E-10	79.51789273	2.2036E-16
6	GeneID:85303	*HIST1H2APS2*	25882154	25882644	rs13208859	25894609	3.37E-08	8.93E-10	76.08759206	1.17312E-15
6	GeneID:10786	*SLC17A3*	25845328	25874471	rs6913795	25848025	3.99E-08	9.49E-10	75.62372871	1.47055E-15
6	GeneID:10338	*HIST1H1PS2*	26016335	26017069	rs10484435	26031811	4.74E-08	5.73E-09	71.68360594	1.00102E-14
14	GeneID:1734	*DIO2*	80663868	80697397	rs145536356	80687991	1.76E-02	5.97E-14	68.98158877	3.72352E-14
6	GeneID:10590	*SCGN*	25652429	25702011	rs35436081	25700342	4.99E-08	6.12E-08	66.84616898	1.05048E-13
6	GeneID:255626	*HIST1H2BA*	25727137	25727573	rs34493019	25714959	1.23E-07	3.28E-08	66.2935433	1.37369E-13
6	GeneID:221613	*HIST1H2AA*	25726291	25726790	rs34493019	25714959	1.19E-07	3.40E-08	66.27939967	1.38316E-13
1	GeneID:4209	*MEF2D*	156433519	156470529	rs1050316	156434703	3.01E-13	2.38E-02	65.13972494	2.40457E-13
14	GeneID:100628307	*LOC100628307*	80677762	80921810	rs113550880	80912456	4.17E-02	2.81E-13	64.1559074	3.8749E-13
**Chr**	**Gene.id**	**Name**	**Start**	**End**	**SNP.id**	**SNP.pos**	**pval(headache)**	**pval(fT4)**	**X^2,4df**	**FCP**

6	GeneID:100419971	*LOC100419971*	25679007	25679651	rs13191296	25684606	3.07E-07	1.36E-07	61.61695182	1.32615E-12
6	GeneID:10246	*SLC17A2*	25912982	25930946	rs35169013	25918027	3.06E-08	1.87E-06	60.98393293	1.8018E-12
6	GeneID:100652981	*LOC100652981*	26854360	26854727	rs77666565	26851415	4.30E-11	1.58E-03	60.64648741	2.12153E-12
1	GeneID:100422997	*MIR4257*	150524405	150524490	rs6693567	150510660	2.20E-11	3.55E-03	60.36478968	2.43143E-12
1	GeneID:54507	*ADAMTSL4*	150521898	150533413	rs6693567	150510660	2.78E-11	4.48E-03	59.42794755	3.82579E-12
6	GeneID:10475	*TRIM38*	25963071	25985358	rs13203673	25979122	4.50E-08	5.56E-06	58.03372626	7.50761E-12
6	GeneID:3024	*HIST1H1A*	26017260	26018040	rs10484435	26031811	4.86E-08	1.23E-05	56.28662293	1.74604E-11
6	GeneID:100189391	*TRNAM20*	26766444	26766516	rs34244947	26761745	4.05E-11	1.55E-02	56.19566258	1.82443E-11
6	GeneID:100189156	*TRNAA20*	26771290	26771362	rs34244947	26761745	4.05E-11	1.55E-02	56.19566258	1.82443E-11
6	GeneID:8350	*HIST1H3A*	26020718	26021186	rs10484435	26031811	5.03E-08	1.28E-05	56.15003408	1.86507E-11
6	GeneID:8359	*HIST1H4A*	26021907	26022278	rs10484435	26031811	5.04E-08	1.28E-05	56.14296521	1.87145E-11
6	GeneID:8366	*HIST1H4B*	26027124	26027480	rs10484435	26031811	5.09E-08	1.32E-05	56.0515337	1.95591E-11
11	GeneID:25921	*ZDHHC5*	57435474	57468659	rs143262971	57468415	1.83E-09	4.35E-04	55.71185746	2.30441E-11
6	GeneID:729392	*POM121L6P*	26838446	26865940	rs77666565	26851415	1.62E-10	5.96E-03	55.3271549	2.77454E-11
11	GeneID:219541	*MED19*	57471186	57479673	rs143262971	57468415	1.10E-09	1.93E-03	53.76227286	5.90176E-11
6	GeneID:100189354	*TRNAM19*	26758550	26758622	rs34244947	26761745	7.96E-11	3.04E-02	53.49506568	6.71303E-11
6	GeneID:11118	*BTN3A2*	26365398	26378548	rs2072803	26392515	1.32E-10	2.16E-02	53.17608959	7.82852E-11
6	GeneID:11119	*BTN3A1*	26402465	26415444	rs2072803	26392515	9.22E-11	3.46E-02	52.94073216	8.76861E-11
6	GeneID:10385	*BTN2A2*	26383324	26395102	rs2072803	26392515	1.41E-10	2.32E-02	52.89173145	8.97808E-11
11	GeneID:710	*SERPING1*	57365027	57382326	rs184391280	57350423	1.56E-06	3.12E-06	52.09779556	1.316E-10
6	GeneID:100507141	*LOC100507141*	26864941	26865525	rs77666565	26851415	1.54E-10	3.65E-02	51.8021796	1.51729E-10
6	GeneID:729400	*LOC729400*	26866104	26868093	rs77666565	26851415	1.54E-10	3.65E-02	51.8021796	1.51729E-10
6	GeneID:100188987	*TRNAH3*	27125906	27125977	rs67540232	27140866	2.56E-10	2.20E-02	51.80208346	1.51736E-10
11	GeneID:100528016	*TMX2-CTNND1*	57479995	57586652	rs143262971	57468415	2.81E-09	2.04E-03	51.76935918	1.54146E-10
6	GeneID:80740	*LY6G6C*	31686425	31689510	rs3131383	31704294	3.74E-08	1.55E-04	51.74605365	1.55885E-10
6	GeneID:100189336	*TRNAI20*	27144994	27145067	rs34569203	27153984	1.63E-10	3.59E-02	51.73042711	1.57062E-10
6	GeneID:100189143	*TRNAT7*	27130050	27130123	rs13194053	27143883	2.52E-10	2.39E-02	51.66706572	1.61926E-10
6	GeneID:3006	*HIST1H1C*	26055968	26056699	rs3752417	26045905	2.39E-08	3.38E-04	51.08175112	2.14612E-10
**Chr**	**Gene.id**	**Name**	**Start**	**End**	**SNP.id**	**SNP.pos**	**pval(headache)**	**pval(fT4)**	**X^2,4df**	**FCP**

6	GeneID:10341	*HIST1H2APS5*	26044128	26044778	rs3752417	26045905	2.46E-08	3.48E-04	50.96713144	2.2678E-10
6	GeneID:8364	*HIST1H4C*	26104176	26104565	rs2237228	26104630	4.38E-08	2.06E-04	50.86507572	2.38192E-10
6	GeneID:8352	*HIST1H3C*	26045639	26046097	rs3752417	26045905	2.54E-08	3.58E-04	50.8469264	2.40281E-10
6	GeneID:3018	*HIST1H2BB*	26043455	26043885	rs3752417	26045905	2.55E-08	3.61E-04	50.82003661	2.4341E-10
6	GeneID:100189131	*TRNAI6*	27599200	27599293	rs35715914	27592003	1.52E-08	6.83E-04	50.58519051	2.7252E-10
6	GeneID:8334	*HIST1H2AC*	26124373	26124918	rs13200797	26122957	5.99E-08	1.88E-04	50.42359748	2.94545E-10
6	GeneID:100189196	*TRNAV20*	27118022	27118094	rs61240102	27124904	3.85E-10	2.93E-02	50.41113904	2.96315E-10
6	GeneID:8347	*HIST1H2BC*	26123695	26124132	rs13200797	26122957	6.02E-08	1.88E-04	50.40750999	2.96833E-10
6	GeneID:3077	*HFE*	26087509	26095469	rs71557316	26072981	3.69E-08	3.10E-04	50.38760321	2.99688E-10
11	GeneID:406919	*MIR130A*	57408671	57408759	rs10750866	57404779	5.86E-06	2.09E-06	50.25447894	3.19501E-10
11	GeneID:219539	*YPEL4*	57412560	57417417	rs10750866	57404779	5.81E-06	2.18E-06	50.18890438	3.29736E-10
6	GeneID:8335	*HIST1H2AB*	26033320	26033796	rs3752417	26045905	2.64E-08	4.87E-04	50.15496333	3.35161E-10
6	GeneID:100422934	*MIR3143*	27115405	27115467	rs61240102	27124904	4.11E-10	3.15E-02	50.14120751	3.37385E-10
6	GeneID:85235	*HIST1H2AH*	27114908	27115346	rs61240102	27124904	4.11E-10	3.15E-02	50.1409218	3.37432E-10
6	GeneID:8358	*HIST1H3B*	26031817	26032288	rs3752417	26045905	2.71E-08	5.00E-04	50.0469554	3.53026E-10
6	GeneID:3010	*HIST1H1T*	26107640	26108364	rs2237228	26104630	6.62E-08	2.08E-04	50.01423145	3.58624E-10
6	GeneID:100189300	*TRNAT13*	27586135	27586208	rs35715914	27592003	1.76E-08	7.89E-04	50.00591007	3.60062E-10
11	GeneID:100507106	*LOC100507106*	57405850	57420606	rs527528	57433327	4.81E-06	3.10E-06	49.85811569	3.86576E-10
6	GeneID:8294	*HIST1H4I*	27107088	27107457	rs67330695	27103654	4.55E-10	3.36E-02	49.80886013	3.95838E-10
6	GeneID:442172	*LOC442172*	27235885	27237008	rs35982103	27243134	7.09E-10	2.26E-02	49.71397623	4.1431E-10
6	GeneID:85236	*HIST1H2BK*	27106072	27114637	rs61240102	27124904	5.18E-10	3.83E-02	49.28778331	5.08485E-10
6	GeneID:100189213	*TRNAI11*	27242990	27243063	rs35982103	27243134	7.02E-10	2.84E-02	49.27838228	5.10787E-10
6	GeneID:7202	*TRNAI1*	27241739	27241812	rs35982103	27243134	7.11E-10	2.88E-02	49.22321983	5.24506E-10
6	GeneID:100289545	*HIST1H3PS1*	26322104	26343616	rs9393703	26356951	5.58E-10	3.68E-02	49.21572738	5.26398E-10
6	GeneID:10279	*PRSS16*	27215502	27224399	rs35589403	27219491	6.85E-10	3.48E-02	48.91900616	6.07048E-10
6	GeneID:29777	*ABT1*	26597171	26600278	rs34781270	26593037	7.08E-10	3.45E-02	48.87482951	6.20067E-10
6	GeneID:100189301	*TRNAL28*	27573417	27573524	rs34105070	27560805	2.64E-08	9.33E-04	48.85096488	6.27216E-10
6	GeneID:100189219	*TRNAY8*	26595102	26595190	rs34781270	26593037	7.24E-10	3.53E-02	48.78221265	6.48274E-10
**Chr**	**Gene.id**	**Name**	**Start**	**End**	**SNP.id**	**SNP.pos**	**pval(headache)**	**pval(fT4)**	**X^2,4df**	**FCP**

6	GeneID:100189172	*TRNAP10*	27059521	27059592	rs6904071	27047256	6.30E-10	4.10E-02	48.76062989	6.55029E-10
6	GeneID:100189157	*TRNAS12*	27065085	27065166	rs34388707	27050396	6.90E-10	3.77E-02	48.74471523	6.60055E-10
6	GeneID:696	*BTN1A1*	26501495	26510653	rs34148261	26511744	1.09E-09	2.51E-02	48.64408873	6.92738E-10
6	GeneID:100189162	*TRNAY7*	26577332	26577420	rs13190739	26587373	8.06E-10	3.66E-02	48.4925477	7.45031E-10
6	GeneID:100420941	*LOC100420941*	26478093	26478749	rs13195401	26463574	6.36E-10	4.65E-02	48.48723709	7.46933E-10
6	GeneID:791231	*TRNAV8*	27618707	27618779	rs144969912	27616843	2.04E-08	1.54E-03	48.35932092	7.94249E-10
6	GeneID:7746	*ZNF193*	28193029	28201265	rs17720293	28214698	1.49E-08	2.16E-03	48.32139517	8.08845E-10
6	GeneID:387036	*GUSBP2*	26839266	26924333	rs77666565	26851415	8.71E-10	3.71E-02	48.31144917	8.12716E-10
6	GeneID:387046	*RPL8P1*	27620370	27620512	rs144969912	27616843	2.07E-08	1.56E-03	48.30474427	8.15337E-10
6	GeneID:100189337	*TRNAY12*	26575798	26575887	rs13190739	26587373	8.45E-10	3.84E-02	48.30310606	8.15978E-10
6	GeneID:100189363	*TRNAV27*	27248049	27248121	rs35982103	27243134	8.92E-10	4.02E-02	48.101398	8.98947E-10
6	GeneID:100189462	*TRNAI28P*	27251864	27251937	rs35982103	27243134	9.29E-10	4.19E-02	47.93633649	9.73069E-10
6	GeneID:80739	*C6orf25*	31691121	31694487	rs3101018	31705864	8.54E-08	4.75E-04	47.85621443	1.01122E-09
6	GeneID:100189038	*TRNAL8*	27570348	27570454	rs34105070	27560805	2.81E-08	1.45E-03	47.84663608	1.01588E-09
2	GeneID:151507	*MSL3P1*	234774083	234777055	rs141028193	234772421	2.07E-09	2.39E-02	47.45235929	1.22746E-09
6	GeneID:100189205	*TRNAS15*	27640229	27640310	rs13217620	27653120	2.46E-08	2.08E-03	47.39141083	1.26388E-09
6	GeneID:4049	*LTA*	31539876	31542098	rs1800628	31546850	1.42E-07	3.63E-04	47.37706971	1.27261E-09
6	GeneID:222699	*TOB2P1*	28183116	28186707	rs13197633	28174757	2.53E-08	2.17E-03	47.24694536	1.35458E-09
6	GeneID:259197	*NCR3*	31556660	31560762	rs1800628	31546850	1.47E-07	3.76E-04	47.23436875	1.36278E-09
6	GeneID:100189159	*TRNAS13*	28180815	28180896	rs13197633	28174757	2.39E-08	2.34E-03	47.22048995	1.37188E-09
6	GeneID:100189297	*TRNAM15*	27560600	27560671	rs34105070	27560805	3.13E-08	1.79E-03	47.21397417	1.37618E-09
6	GeneID:100189268	*TRNAQ21*	27515531	27515602	rs35848276	27521096	1.72E-08	3.33E-03	47.16242896	1.41063E-09
6	GeneID:100189053	*TRNAK8*	27559593	27559665	rs34105070	27560805	3.26E-08	1.86E-03	47.05265387	1.48689E-09
6	GeneID:23564	*DDAH2*	31694817	31698039	rs3132445	31712196	1.05E-07	5.83E-04	47.0396433	1.4962E-09
6	GeneID:94017	*TRNAS1*	27513468	27513549	rs35848276	27521096	1.71E-08	3.66E-03	46.9876468	1.53398E-09
6	GeneID:100189070	*TRNAS9*	27521192	27521273	rs35848276	27521096	1.82E-08	3.51E-03	46.94692738	1.56423E-09
6	GeneID:7124	*TNF*	31543350	31546112	rs1800628	31546850	1.59E-07	4.08E-04	46.91340439	1.58958E-09
6	GeneID:100189428	*TRNAR29*	27638344	27638416	rs13217620	27653120	2.79E-08	2.35E-03	46.89505506	1.60363E-09
**Chr**	**Gene.id**	**Name**	**Start**	**End**	**SNP.id**	**SNP.pos**	**pval(headache)**	**pval(fT4)**	**X^2,4df**	**FCP**

6	GeneID:100189095	*TRNAI5*	27636362	27636435	rs56405707	27640246	2.97E-08	2.26E-03	46.85303264	1.63627E-09
6	GeneID:7940	*LST1*	31553956	31556686	rs1800628	31546850	1.67E-07	4.28E-04	46.71680093	1.74672E-09
6	GeneID:4050	*LTB*	31548335	31550202	rs1800628	31546850	1.77E-07	4.54E-04	46.48444591	1.95255E-09
6	GeneID:100189352	*TRNAS24*	27509554	27509635	rs35848276	27521096	1.99E-08	4.26E-03	46.37959766	2.0532E-09
6	GeneID:80737	*C6orf27*	31733371	31745108	rs3117573	31718396	1.32E-07	6.69E-04	46.30183116	2.13117E-09
6	GeneID:100128240	*LOC100128240*	27528605	27529550	rs35848276	27521096	2.14E-08	4.14E-03	46.28966561	2.14364E-09
6	GeneID:100189012	*TRNAR5*	27529963	27530049	rs35848276	27521096	2.14E-08	4.14E-03	46.28966561	2.14364E-09
6	GeneID:1192	*CLIC1*	31698358	31704341	rs3130484	31715882	1.20E-07	7.45E-04	46.27348502	2.16032E-09
6	GeneID:100287329	*LOC100287329*	31527348	31540474	rs1800628	31546850	1.92E-07	4.92E-04	46.16483004	2.2758E-09
6	GeneID:4758	*NEU1*	31826829	31830709	rs501942	31840477	4.13E-07	2.31E-04	46.14874164	2.29342E-09
6	GeneID:100189200	*TRNAD9*	27551236	27551307	rs34105070	27560805	3.00E-08	3.32E-03	46.06199147	2.39077E-09
11	GeneID:81210	*OR5BA1P*	57633774	57634752	rs11229148	57621460	5.28E-06	1.90E-05	46.04841448	2.40637E-09
6	GeneID:80736	*SLC44A4*	31830969	31846823	rs501942	31840477	3.46E-07	2.95E-04	46.01261248	2.44801E-09
6	GeneID:387032	*ZKSCAN4*	28212490	28220002	rs17720293	28214698	1.51E-08	6.88E-03	45.9754695	2.49197E-09
6	GeneID:401251	*C6orf26*	31730773	31732627	rs3130484	31715882	1.39E-07	7.50E-04	45.96675582	2.5024E-09
6	GeneID:10919	*EHMT2*	31847536	31865464	rs501942	31840477	3.58E-07	3.05E-04	45.87700332	2.61236E-09
6	GeneID:645950	*HNRNPA1P1*	27491226	27492316	rs34573979	27480526	1.29E-08	8.74E-03	45.8068534	2.70165E-09
2	GeneID:55355	*HJURP*	234745486	234763212	rs141028193	234772421	4.15E-09	3.52E-02	45.2980288	3.44724E-09
6	GeneID:100189315	*TRNAM17*	27870271	27870342	rs13218875	27884012	2.14E-08	6.82E-03	45.29470707	3.45272E-09
6	GeneID:222698	*NKAPL*	28227098	28228736	rs17720293	28214698	1.25E-08	1.21E-02	45.23198152	3.558E-09
6	GeneID:651302	*ZNF192P1*	28129551	28137376	rs35749575	28114818	2.50E-08	6.17E-03	45.18611587	3.637E-09
6	GeneID:5491	*PPIAP9*	31486754	31488108	rs3130614	31476458	2.03E-07	7.78E-04	45.13599207	3.72535E-09
6	GeneID:100189161	*TRNAV17*	27648885	27648957	rs13217620	27653120	2.56E-08	6.24E-03	45.11767929	3.75815E-09
6	GeneID:100189314	*TRNAG29*	27870686	27870756	rs13218875	27884012	2.24E-08	7.25E-03	45.08402782	3.81919E-09
6	GeneID:100189071	*TRNAI4*	27655967	27656040	rs13217620	27653120	2.60E-08	6.33E-03	45.0573955	3.8682E-09
6	GeneID:790951	*TRNAS4*	27499987	27500068	rs13195636	27509493	2.35E-08	7.16E-03	45.01163457	3.95388E-09
6	GeneID:7754	*ZNF204P*	27325602	27343153	rs67859638	27357978	7.84E-09	2.15E-02	45.00595869	3.96463E-09
6	GeneID:100189340	*TRNAT15*	27652474	27652547	rs13217620	27653120	2.63E-08	6.43E-03	44.99887515	3.9781E-09
**Chr**	**Gene.id**	**Name**	**Start**	**End**	**SNP.id**	**SNP.pos**	**pval(headache)**	**pval(fT4)**	**X^2,4df**	**FCP**

6	GeneID:100419608	*LOC100419608*	28083427	28083998	rs66886492	28089731	1.52E-08	1.11E-02	44.99750532	3.98071E-09
6	GeneID:100189189	*TRNAQ16*	27487308	27487379	rs34573979	27480526	1.37E-08	1.25E-02	44.97578414	4.02232E-09
6	GeneID:8344	*HIST1H2BE*	26184024	26184458	rs67575965	26196593	3.65E-09	4.72E-02	44.96493179	4.04327E-09
6	GeneID:100532732	*MSH5-C6orf26*	31707725	31732627	rs3130484	31715882	1.80E-07	9.69E-04	44.9440735	4.08384E-09
6	GeneID:100147813	*RNU7-26P*	27865284	27865343	rs61742093	27879982	3.01E-08	5.98E-03	44.87495205	4.22123E-09
6	GeneID:222701	*ZNF192P2*	28155716	28157189	rs13195291	28169241	2.82E-08	6.64E-03	44.79672769	4.38228E-09
3	GeneID:100302112	*MIR1284*	71591121	71591240	rs7610856	71579022	1.79E-07	1.17E-03	44.57940622	4.8626E-09
12	GeneID:84329	*HVCN1*	111086491	111127617	rs7961345	111081598	5.07E-09	4.13E-02	44.57133827	4.88141E-09
6	GeneID:81697	*OR2B2*	27878963	27880174	rs13218875	27884012	2.53E-08	8.91E-03	44.42622359	5.23241E-09
6	GeneID:80345	*ZSCAN16*	28092387	28097857	rs66886492	28089731	2.04E-08	1.20E-02	44.26131003	5.66196E-09
6	GeneID:346157	*ZNF391*	27356524	27369227	rs67859638	27357978	9.49E-09	2.60E-02	44.24507825	5.70609E-09
6	GeneID:81695	*OR2B7P*	28014213	28015147	rs13193295	28003228	2.28E-08	1.17E-02	44.08456237	6.16147E-09
6	GeneID:721	*C4B*	31982572	32003195	rs433061	32014828	7.56E-08	3.54E-03	44.08220522	6.16842E-09
6	GeneID:100302242	*MIR1236*	31924616	31924717	rs1270942	31918860	9.34E-08	2.89E-03	44.06344085	6.22403E-09
6	GeneID:8348	*HIST1H2BO*	27861203	27861669	rs13199649	27868792	3.24E-08	8.77E-03	43.9612995	6.53563E-09
6	GeneID:8336	*HIST1H2AM*	27860477	27860963	rs13199649	27868792	3.25E-08	8.78E-03	43.95582375	6.55277E-09
6	GeneID:340192	*IQCB2P*	27978329	27980680	rs71559067	27994416	2.46E-08	1.19E-02	43.91189074	6.6919E-09
6	GeneID:7936	*RDBP*	31919864	31926864	rs1270942	31918860	9.86E-08	3.05E-03	43.84867417	6.89728E-09
6	GeneID:100507173	*LOC100507173*	27661814	27678001	rs13217620	27653120	3.93E-08	7.85E-03	43.8014694	7.05474E-09
6	GeneID:8356	*HIST1H3J*	27858093	27858570	rs13199649	27868792	3.41E-08	9.22E-03	43.7605625	7.19409E-09
6	GeneID:221584	*ZSCAN12P1*	28058929	28063493	rs35952432	28074901	2.42E-08	1.34E-02	43.70552731	7.38591E-09
11	GeneID:10978	*CLP1*	57425216	57429337	rs527528	57433327	3.03E-06	1.09E-04	43.66510165	7.53005E-09
6	GeneID:629	*CFB*	31913721	31919861	rs1270942	31918860	1.06E-07	3.27E-03	43.56885874	7.88462E-09
6	GeneID:65944	*OR2B8P*	28020906	28022043	rs13200214	28017250	2.81E-08	1.24E-02	43.56303624	7.9066E-09
6	GeneID:23540	*TRNAS3*	27473607	27473688	rs34573979	27480526	1.14E-08	3.05E-02	43.56105622	7.91408E-09
6	GeneID:4277	*MICB*	31465855	31478901	rs3130614	31476458	3.17E-07	1.10E-03	43.5559164	7.93355E-09
6	GeneID:100189292	*TRNAD14*	27471523	27471594	rs34573979	27480526	1.16E-08	3.12E-02	43.46842718	8.27239E-09
6	GeneID:81694	*OR2W2P*	28001693	28002640	rs13193295	28003228	2.23E-08	1.75E-02	43.33201958	8.82971E-09
**Chr**	**Gene.id**	**Name**	**Start**	**End**	**SNP.id**	**SNP.pos**	**pval(headache)**	**pval(fT4)**	**X^2,4df**	**FCP**

6	GeneID:100189247	*TRNAS19*	27470818	27470899	rs34573979	27480526	1.22E-08	3.28E-02	43.27494202	9.07389E-09
6	GeneID:23437	*TRNAS2*	27463593	27463674	rs58616630	27474715	1.23E-08	3.29E-02	43.24874921	9.18819E-09
6	GeneID:100129195	*LOC100129195*	28058336	28105071	rs35749575	28114818	2.49E-08	1.67E-02	43.20669813	9.3747E-09
6	GeneID:7745	*ZNF192*	28109716	28125236	rs35749575	28114818	2.60E-08	1.63E-02	43.16607119	9.55848E-09
6	GeneID:692233	*SNORD117*	31504151	31504226	rs9267488	31514247	4.99E-07	8.95E-04	43.05748257	1.00675E-08
6	GeneID:6499	*SKIV2L*	31926581	31937532	rs1270942	31918860	1.21E-07	3.74E-03	43.03242741	1.01888E-08
6	GeneID:7718	*ZNF165*	28048482	28057341	rs34166054	28065801	4.18E-08	1.30E-02	42.67100716	1.21089E-08
6	GeneID:7212	*TRNAM2*	27745664	27745735	rs35037868	27759115	5.36E-08	1.04E-02	42.61803095	1.24192E-08
15	GeneID:9728	*SECISBP2L*	49280835	49338760	rs7183756	49307027	4.56E-05	1.24E-05	42.59104539	1.25803E-08
6	GeneID:7741	*ZNF187*	28234788	28245981	rs13208096	28225311	4.30E-08	1.39E-02	42.47568032	1.32928E-08
6	GeneID:1388	*ATF6B*	32083045	32096017	rs1269852	32080191	8.28E-08	7.45E-03	42.41252749	1.36997E-08
6	GeneID:8363	*HIST1H4J*	27791903	27792258	rs35202262	27799514	9.75E-08	6.50E-03	42.35958154	1.40505E-08
6	GeneID:100189214	*TRNAQ17*	27759135	27759206	rs35037868	27759115	5.74E-08	1.11E-02	42.34396696	1.41556E-08
6	GeneID:8368	*HIST1H4L*	27840926	27841289	rs45509595	27840926	7.42E-08	8.64E-03	42.33451143	1.42197E-08
6	GeneID:1589	*CYP21A2*	32006093	32009447	rs433061	32014828	1.18E-07	5.51E-03	42.31431631	1.43574E-08
6	GeneID:692199	*SNORD84*	31508878	31508955	rs9267488	31514247	6.18E-07	1.11E-03	42.20455251	1.51299E-08
6	GeneID:100129755	*RSL24D1P1*	27748462	27748890	rs35037868	27759115	5.96E-08	1.15E-02	42.19834039	1.51748E-08
6	GeneID:8354	*HIST1H3I*	27839623	27840099	rs45509595	27840926	7.69E-08	8.96E-03	42.19038514	1.52325E-08
6	GeneID:8362	*HIST1H4K*	27798952	27799305	rs35202262	27799514	1.07E-07	6.51E-03	42.17501546	1.53447E-08
6	GeneID:10337	*HIST1H4PS1*	27774945	27775115	rs13195728	27771106	1.19E-07	6.03E-03	42.11342178	1.58026E-08
6	GeneID:8329	*HIST1H2AI*	27775977	27776445	rs13195728	27771106	1.19E-07	6.05E-03	42.10309658	1.58806E-08
6	GeneID:442179	*OR1F12*	28041026	28042002	rs71559070	28038929	4.13E-08	1.75E-02	42.09907188	1.59112E-08
6	GeneID:25803	*SPDEF*	34505580	34524091	rs2233630	34524778	5.47E-04	1.32E-06	42.09239211	1.5962E-08
6	GeneID:100507241	*LOC100507241*	28046570	28048908	rs35902873	28058949	4.16E-08	1.84E-02	41.97660163	1.6869E-08
6	GeneID:8340	*HIST1H2BL*	27775257	27775709	rs13195728	27771106	1.24E-07	6.31E-03	41.93532867	1.72045E-08
6	GeneID:8357	*HIST1H3H*	27777842	27778314	rs13195728	27771106	1.24E-07	6.31E-03	41.93275671	1.72257E-08
6	GeneID:8342	*HIST1H2BM*	27782822	27783267	rs13195728	27771106	1.25E-07	6.36E-03	41.89845158	1.751E-08
6	GeneID:717	*C2*	31868776	31913449	rs1270942	31918860	1.61E-07	5.00E-03	41.877506	1.76859E-08
**Chr**	**Gene.id**	**Name**	**Start**	**End**	**SNP.id**	**SNP.pos**	**pval(headache)**	**pval(fT4)**	**X^2,4df**	**FCP**

6	GeneID:534	*ATP6V1G2*	31512228	31514625	rs9267488	31514247	6.81E-07	1.22E-03	41.81415812	1.82287E-08
6	GeneID:8331	*HIST1H2AJ*	27782080	27782518	rs13195728	27771106	1.28E-07	6.51E-03	41.8074963	1.82867E-08
6	GeneID:84547	*PGBD1*	28249314	28270326	rs33932084	28268824	2.88E-08	2.97E-02	41.75872933	1.87172E-08
12	GeneID:5501	*PPP1CC*	111157613	111180783	rs73194035	111165793	2.98E-08	3.04E-02	41.64916536	1.97217E-08
6	GeneID:100532737	*ATP6V1G2-DDX39B*	31497996	31514625	rs9267488	31514247	7.28E-07	1.31E-03	41.54925204	2.06845E-08
6	GeneID:64288	*ZNF323*	28292514	28324048	rs13214023	28332141	4.60E-08	2.44E-02	41.21203639	2.42938E-08
6	GeneID:4795	*NFKBIL1*	31514628	31526606	rs9267488	31514247	8.25E-07	1.48E-03	41.04449288	2.63141E-08
6	GeneID:791230	*TRNAV7*	27721179	27721251	rs17693963	27710165	9.89E-08	1.25E-02	41.02108607	2.66094E-08
6	GeneID:100288742	*HIST1H2BPS1*	25732012	25732391	rs11751622	25742888	4.46E-02	2.88E-08	40.94779191	2.75557E-08
6	GeneID:100131289	*LOC100131289*	27729523	27730966	rs35501037	27739566	1.25E-07	1.05E-02	40.90257778	2.8156E-08
6	GeneID:100189074	*TRNAQ10*	27763640	27763711	rs35037868	27759115	5.76E-08	2.32E-02	40.8700275	2.85963E-08
6	GeneID:100189390	*TRNAK31*	27543846	27543918	rs10484399	27534528	3.56E-08	3.84E-02	40.82118503	2.92699E-08
6	GeneID:100379623	*LOC100379623*	26164261	26168404	rs17598658	26175866	5.03E-08	2.83E-02	40.74134211	3.04052E-08
6	GeneID:100189436	*TRNAW9*	26319330	26319401	rs34107459	26328353	6.62E-08	2.47E-02	40.46623186	3.46644E-08
11	GeneID:280636	*C11orf31*	57508722	57510883	rs9420	57510294	2.00E-06	8.65E-04	40.35438692	3.65616E-08
6	GeneID:100189410	*TRNAR28*	26323046	26323118	rs34107459	26328353	6.81E-08	2.54E-02	40.34952251	3.66464E-08
11	GeneID:81156	*OR9I2P*	57912321	57912806	rs143742976	57923874	4.83E-08	3.94E-02	40.15834997	4.01401E-08
6	GeneID:7148	*TNXB*	32008932	32077151	rs1269852	32080191	1.76E-07	1.10E-02	40.1298936	4.06878E-08
6	GeneID:3009	*HIST1H1B*	27834570	27835359	rs45509595	27840926	6.54E-08	2.97E-02	40.1179392	4.09202E-08
6	GeneID:100189094	*TRNAM6*	26313352	26313423	rs34107459	26328353	7.30E-08	2.72E-02	40.07309059	4.18036E-08
6	GeneID:100189430	*TRNAS27*	26312824	26312905	rs13220522	26316295	8.50E-08	2.46E-02	39.9728059	4.38484E-08
14	GeneID:3705	*ITPK1*	93403259	93582263	rs28540738	93591673	1.85E-05	1.18E-04	39.8967508	4.54654E-08
6	GeneID:100189185	*TRNAQ15*	26311975	26312046	rs13220522	26316295	8.37E-08	2.71E-02	39.80906659	4.74036E-08
6	GeneID:100189321	*TRNAQ25*	26311424	26311495	rs13220522	26316295	8.37E-08	2.71E-02	39.80881924	4.74092E-08
6	GeneID:10340	*HIST1H2BPS2*	27831840	27832179	rs45509595	27840926	6.55E-08	3.47E-02	39.80176277	4.75687E-08
6	GeneID:100189101	*TRNAV15*	27696327	27696399	rs34409918	27685348	7.97E-08	2.88E-02	39.78222466	4.80133E-08
6	GeneID:100189382	*TRNAS25*	26327817	26327898	rs9467714	26340785	6.66E-08	3.45E-02	39.78205797	4.80171E-08
6	GeneID:8332	*HIST1H2AL*	27833107	27833576	rs45509595	27840926	6.69E-08	3.49E-02	39.75121253	4.87274E-08
**Chr**	**Gene.id**	**Name**	**Start**	**End**	**SNP.id**	**SNP.pos**	**pval(headache)**	**pval(fT4)**	**X^2,4df**	**FCP**

6	GeneID:100189374	*TRNAT18*	27694473	27694546	rs34409918	27685348	8.14E-08	2.95E-02	39.69641891	5.00151E-08
6	GeneID:100507025	*LOC100507025*	26281278	26285763	rs55706012	26266311	8.39E-08	2.87E-02	39.69274177	5.01027E-08
6	GeneID:8341	*HIST1H2BN*	27806440	27806888	rs35202262	27799514	1.04E-07	2.38E-02	39.64161865	5.13369E-08
6	GeneID:8330	*HIST1H2AK*	27805658	27806117	rs35202262	27799514	1.04E-07	2.38E-02	39.64127686	5.13452E-08
6	GeneID:100189164	*TRNAL16*	27688898	27688980	rs34409918	27685348	8.38E-08	2.97E-02	39.6232909	5.17867E-08
6	GeneID:8355	*HIST1H3G*	26271146	26271612	rs55706012	26266311	8.71E-08	2.98E-02	39.54095078	5.38565E-08
6	GeneID:8346	*HIST1H2BI*	26273204	26273640	rs55706012	26266311	8.86E-08	3.03E-02	39.47365278	5.56093E-08
6	GeneID:8333	*HIST1H2APS4*	26272421	26272768	rs55706012	26266311	8.90E-08	3.04E-02	39.45563345	5.60882E-08
6	GeneID:100189396	*TRNAR26*	26328368	26328440	rs9467714	26340785	7.24E-08	3.75E-02	39.44714649	5.63152E-08
6	GeneID:3017	*HIST1H2BD*	26158349	26171577	rs17598658	26175866	7.00E-08	3.93E-02	39.42056318	5.70321E-08
6	GeneID:100189040	*TRNAM4*	26330529	26330600	rs9467714	26340785	7.62E-08	3.95E-02	39.24453468	6.20146E-08
6	GeneID:394254	*GPR89P*	27704354	27706293	rs13202291	27698857	1.10E-07	2.76E-02	39.21324805	6.29446E-08
6	GeneID:100189048	*TRNAW2*	26331672	26331743	rs9467714	26340785	7.81E-08	4.05E-02	39.14104653	6.51441E-08
11	GeneID:100507231	*LOC100507231*	57485518	57486002	rs547891	57499065	2.13E-06	1.57E-03	39.02957824	6.86913E-08
6	GeneID:3106	*HLA-B*	31321649	31324989	rs2523589	31327334	2.69E-04	1.31E-05	38.92951275	7.20394E-08
6	GeneID:729816	*DHFRP2*	31331244	31334742	rs2523589	31327334	2.69E-04	1.31E-05	38.92951275	7.20394E-08
11	GeneID:643376	*BTBD18*	57510986	57519253	rs9420	57510294	2.94E-06	1.27E-03	38.80587454	7.64019E-08
6	GeneID:100189218	*TRNAS16*	26305718	26305801	rs13220522	26316295	1.04E-07	3.91E-02	38.63784709	8.27559E-08
1	GeneID:100418820	*PHBP12*	51246012	51246821	rs1416685	51243374	8.72E-05	4.79E-05	38.58579063	8.48293E-08
6	GeneID:8345	*HIST1H2BH*	26251879	26252303	rs55706012	26266311	9.40E-08	4.66E-02	38.49293472	8.86573E-08
1	GeneID:2029	*ENSA*	150594599	150602098	rs138520356	150586501	4.19E-06	1.11E-03	38.36588644	9.41755E-08
6	GeneID:7232	*TRNAR3*	26299905	26299977	rs10484439	26309908	1.20E-07	4.00E-02	38.31223319	9.66076E-08
11	GeneID:390183	*RPS4XP13*	57343702	57344578	rs184391280	57350423	8.32E-07	7.50E-03	37.78537789	1.2408E-07
1	GeneID:100271104	*RPS26P12*	174891238	174891571	rs4650666	174880809	4.13E-05	1.83E-04	37.4058124	1.4858E-07
1	GeneID:100128889	*LOC100128889*	174818808	174819306	rs1894200	174811177	7.80E-05	1.10E-04	37.14517347	1.68142E-07
18	GeneID:342784	*LOC342784*	57863787	57865406	rs476828	57852587	5.59E-03	1.78E-06	36.8486392	1.93538E-07
9	GeneID:58499	*ZNF462*	109625378	109773807	rs2134063	109695139	1.13E-04	9.68E-05	36.65345325	2.12306E-07
11	GeneID:1500	*CTNND1*	57529234	57586652	rs3781885	57570680	6.11E-06	1.90E-03	36.54550347	2.23454E-07
**Chr**	**Gene.id**	**Name**	**Start**	**End**	**SNP.id**	**SNP.pos**	**pval(headache)**	**pval(fT4)**	**X^2,4df**	**FCP**

6	GeneID:100131913	*RPL5P20*	19348341	19349350	rs113153854	19343431	5.42E-03	2.14E-06	36.53979201	2.2406E-07
1	GeneID:359781	*MRPS6P2*	51312141	51312514	rs6684901	51298828	1.90E-04	6.46E-05	36.4296626	2.36067E-07
3	GeneID:100506815	*FOXP1-IT1*	71619425	71623608	rs62244889	71609007	5.07E-06	2.61E-03	36.27913631	2.53523E-07
1	GeneID:646891	*SDCCAG3P2*	175013765	175014848	rs16865298	175013165	6.50E-03	2.06E-06	36.25803859	2.56071E-07
6	GeneID:29993	*PACSIN1*	34433853	34504039	rs12202516	34490146	1.14E-02	1.22E-06	36.19647163	2.63651E-07
1	GeneID:100422535	*LOC100422535*	50871104	50871696	rs2484680	50868417	6.30E-06	2.43E-03	35.99052496	2.90671E-07
15	GeneID:729344	*KRT8P24*	49263922	49265761	rs4775791	49278326	3.66E-04	4.27E-05	35.94954632	2.96368E-07
6	GeneID:29113	*C6orf15*	31079000	31080332	rs2233980	31079644	7.76E-07	2.17E-02	35.79777291	3.18455E-07
1	GeneID:100129919	*HMGB1P45*	50864534	50865042	rs2484680	50868417	6.78E-06	2.62E-03	35.69419309	3.34464E-07
11	GeneID:23360	*FNBP4*	47738069	47788993	rs55944135	47792469	1.79E-02	1.00E-06	35.67018193	3.38288E-07
9	GeneID:266655	*LINC00094*	136890561	136896719	rs36093130	136906986	8.79E-03	2.61E-06	35.18042573	4.26532E-07
6	GeneID:1041	*CDSN*	31082865	31088252	rs2233980	31079644	9.12E-07	2.55E-02	35.15210359	4.32286E-07
1	GeneID:63950	*DMRTA2*	50883223	50889119	rs2484680	50868417	6.71E-06	3.80E-03	34.97116892	4.70913E-07
11	GeneID:23279	*NUP160*	47799670	47870057	rs55944135	47792469	1.77E-02	1.50E-06	34.89703868	4.87715E-07
15	GeneID:100131797	*RPL15P19*	49469498	49470980	rs34854443	49475296	3.95E-03	6.88E-06	34.84004032	5.01039E-07
6	GeneID:100507444	*PPP1R2P1*	32844255	32847851	rs115591082	32844103	3.22E-06	8.76E-03	34.76938447	5.1806E-07
6	GeneID:55604	*LRRC16A*	25279656	25620758	rs927985	25412811	1.91E-06	1.63E-02	34.57036065	5.69168E-07
11	GeneID:10658	*CELF1*	47487489	47574792	rs72907976	47566232	1.52E-02	2.05E-06	34.56137114	5.71592E-07
1	GeneID:11124	*FAF1*	50906935	51425936	rs1278519	50897342	1.19E-04	2.70E-04	34.50226634	5.87784E-07
15	GeneID:9318	*COPS2*	49417471	49447854	rs12439497	49406214	1.39E-03	2.78E-05	34.1320639	7.00131E-07
3	GeneID:27086	*FOXP1*	71004736	71633140	rs7610856	71579022	4.36E-06	9.01E-03	34.10538492	7.09009E-07
6	GeneID:170679	*PSORS1C1*	31082608	31107869	rs2233980	31079644	1.19E-06	3.32E-02	34.09625222	7.12074E-07
14	GeneID:1531	*CYB5AP3*	93604300	93605122	rs28540738	93591673	5.50E-06	7.31E-03	34.05717621	7.25337E-07
1	GeneID:100270975	*RPL30P1*	174059272	174059610	rs12563301	174060062	8.85E-05	4.63E-04	34.02027782	7.38087E-07
1	GeneID:405	*ARNT*	150782181	150849244	rs150253674	150788065	2.28E-05	1.97E-03	33.83831505	8.04305E-07
11	GeneID:10335	*MRVI1*	10594638	10715535	rs4909945	10673739	1.83E-06	2.52E-02	33.78005599	8.26733E-07
9	GeneID:100130548	*LOC100130548*	136919412	136924237	rs36093130	136906986	9.52E-03	5.19E-06	33.64400146	8.81565E-07
1	GeneID:63931	*MRPS14*	174982094	174992591	rs6662806	175006747	1.98E-02	2.61E-06	33.55929659	9.17518E-07
**Chr**	**Gene.id**	**Name**	**Start**	**End**	**SNP.id**	**SNP.pos**	**pval(headache)**	**pval(fT4)**	**X^2,4df**	**FCP**

11	GeneID:283193	*OR5AZ1P*	57684774	57685702	rs641325	57681828	9.00E-05	6.20E-04	33.40187313	9.88261E-07
13	GeneID:2835	*GPR12*	27329337	27334922	rs76623956	27344759	1.05E-04	5.74E-04	33.25009801	1.06161E-06
11	GeneID:219965	*OR5B17*	58125598	58126542	rs118138203	58135935	2.64E-06	2.51E-02	33.06051983	1.16088E-06
1	GeneID:100302291	*LOC100302291*	174329634	174337240	rs2049991	174325566	1.21E-04	5.69E-04	32.97949674	1.20608E-06
11	GeneID:648315	*VN2R9P*	57808240	57834431	rs140301686	57824505	3.55E-06	2.00E-02	32.92686711	1.23638E-06
9	GeneID:169792	*GLIS3*	3824128	4300035	rs118047658	3860001	2.61E-02	2.76E-06	32.89436174	1.25547E-06
11	GeneID:100420122	*LOC100420122*	58121463	58121972	rs118138203	58135935	2.60E-06	2.99E-02	32.74076798	1.34971E-06
11	GeneID:8501	*SLC43A1*	57252004	57283192	rs76642542	57290061	7.08E-06	1.11E-02	32.72044041	1.36271E-06
11	GeneID:401692	*OR5B1P*	58133394	58134041	rs118138203	58135935	3.13E-06	2.98E-02	32.37305557	1.60498E-06
15	GeneID:2585	*GALK2*	49447976	49622002	rs11631782	49542284	4.19E-03	2.24E-05	32.3669484	1.6096E-06
1	GeneID:9909	*DENND4B*	153901977	153919154	rs12033532	153888820	3.76E-06	2.75E-02	32.16745652	1.76811E-06
1	GeneID:100422536	*LOC100422536*	243430920	243431421	rs12741781	243428152	2.91E-06	3.98E-02	31.94192575	1.9661E-06
6	GeneID:5698	*PSMB9*	32821938	32827628	rs45506201	32808299	4.07E-06	2.85E-02	31.93709037	1.97058E-06
12	GeneID:84260	*TCHP*	110338079	110355874	rs116940627	110336546	2.60E-06	4.73E-02	31.82389912	2.07836E-06

Chr, chromosome number; Gene.id, Gene ID; Name, Gene; Start, start point of the gene; End, end point of the gene; SNP.id, top SNP associated with the gene; SNP.pos, position of the top SNP; pval(headache), p-value of the gene in headache dataset; pval(fT4), p-value of the gene in fT4 dataset.

Supplementary Table S15. Pathways associated with headache and hypothyroidism.

source	term_name	term_id	adjusted_ p_value	negative_log10_ of_adjusted_ p_value	term_size	query_size	intersection_ size	effective_ domain_size	intersections
GO:MF	MHC class II protein complex binding	GO:0023026	1.07E-04	3.968940607	25	225	6	20183	*HLA-DQA1,HLA-DRB5,HLA-DOB,HLA-DMA,HLA-DRA,HLA-DMB*
GO:MF	peptide antigen binding	GO:0042605	6.24E-04	3.205148403	33	225	6	20183	*HLA-DQA1,HLA-DRB5,TAP1,HLA-DRA,HLA-E,HLA-G*
GO:MF	MHC protein complex binding	GO:0023023	6.24E-04	3.205148403	33	225	6	20183	*HLA-DQA1,HLA-DRB5,HLA-DOB,HLA-DMA,HLA-DRA,HLA-DMB*
GO:MF	protein phosphatase binding	GO:0019903	2.94E-03	2.531822053	150	225	10	20183	*ENSA,STAT1,PPP1R10,PPP1R11,MFHAS1,PPP1R3B,MTMR9,PTK2,ANAPC7,PPP1CC*

GO:MF	phosphatase binding	GO:0019902	5.06E-03	2.296262875	195	225	11	20183	*ENSA,STAT1,PPP1R10,PPP1R11,MFHAS1,PPP1R3B,MTMR9,PTK2,ANAPC7,PPP1CC,MAPK1*
GO:MF	MHC class II receptor activity	GO:0032395	2.96E-02	1.528392789	8	225	3	20183	*HLA-DQA1,HLA-DOB,HLA-DRA*
GO:BP	peptide antigen assembly with MHC class II protein complex	GO:0002503	8.89E-06	5.051246559	14	232	6	21100	*HLA-DQA1,HLA-DRB5,HLA-DOB,HLA-DMA,HLA-DRA,HLA-DMB*
GO:BP	MHC class II protein complex assembly	GO:0002399	8.89E-06	5.051246559	14	232	6	21100	*HLA-DQA1,HLA-DRB5,HLA-DOB,HLA-DMA,HLA-DRA,HLA-DMB*
GO:BP	antigen processing and presentation	GO:0019882	3.49E-05	4.457607158	128	232	12	21100	*HLA-DQA1,HLA-DQB1,HLA-DRB5,TAP1,HLA-DOB,HLA-DMA,HLA-DRA,HFE,HLA-DMB,HLA-E,HLA-G,RAB5B*
GO:BP	antigen processing and presentation of peptide antigen	GO:0048002	4.38E-05	4.358404106	82	232	10	21100	*HLA-DQA1,HLA-DRB5,TAP1,HLA-DOB,HLA-DMA,HLA-DRA,HFE,HLA-DMB,HLA-E,HLA-G*
GO:BP	peptide antigen assembly with MHC protein complex	GO:0002501	5.30E-05	4.27613376	18	232	6	21100	*HLA-DQA1,HLA-DRB5,HLA-DOB,HLA-DMA,HLA-DRA,HLA-DMB*
GO:BP	MHC protein complex assembly	GO:0002396	5.30E-05	4.27613376	18	232	6	21100	*HLA-DQA1,HLA-DRB5,HLA-DOB,HLA-DMA,HLA-DRA,HLA-DMB*
GO:BP	antigen processing and presentation of peptide or polysaccharide antigen via MHC class II	GO:0002504	2.17E-04	3.663172655	36	232	7	21100	*HLA-DQA1,HLA-DQB1,HLA-DRB5,HLA-DOB,HLA-DMA,HLA-DRA,HLA-DMB*
GO:BP	antigen processing and presentation of exogenous peptide antigen	GO:0002478	4.98E-04	3.302574546	59	232	8	21100	*HLA-DQA1,HLA-DRB5,TAP1,HLA-DOB,HLA-DMA,HLA-DRA,HLA-DMB,HLA-E*
GO:BP	antigen processing and presentation of exogenous peptide antigen via MHC class II	GO:0019886	9.80E-04	3.00872805	28	232	6	21100	*HLA-DQA1,HLA-DRB5,HLA-DOB,HLA-DMA,HLA-DRA,HLA-DMB*

GO:BP	antigen processing and presentation of exogenous antigen	GO:0019884	1.91E-03	2.718546439	70	232	8	21100	*HLA-DQA1,HLA-DRB5,TAP1,HLA-DOB,HLA-DMA,HLA-DRA,HLA-DMB,HLA-E*
GO:BP	antigen processing and presentation of peptide antigen via MHC class II	GO:0002495	2.27E-03	2.643517444	32	232	6	21100	*HLA-DQA1,HLA-DRB5,HLA-DOB,HLA-DMA,HLA-DRA,HLA-DMB*
GO:BP	antigen processing and presentation of endogenous peptide antigen	GO:0002483	3.04E-03	2.517796578	19	232	5	21100	*TAP1,HLA-DRA,HFE,HLA-E,HLA-G*
GO:BP	antigen processing and presentation of endogenous antigen	GO:0019883	1.61E-02	1.792488139	26	232	5	21100	*TAP1,HLA-DRA,HFE,HLA-E,HLA-G*
GO:BP	negative regulation of cell-cell adhesion	GO:0022408	1.67E-02	1.777461143	190	232	11	21100	*RUNX3,MUC21,HFE,HLA-G,PTK2,IFNA2,SPI1,SH2B3,TRPV4,CBFB,PTPN2*
GO:BP	regulation of leukocyte cell-cell adhesion	GO:1903037	4.12E-02	1.385244886	334	232	14	21100	*RUNX3,HLA-DQA1,HLA-DRB5,HLA-DOB,HLA-DMA,HLA-DRA,HFE,HLA-DMB,HLA-E,HLA-G,IFNA2,ZMIZ1,CBFB,PTPN2*
GO:CC	MHC protein complex	GO:0042611	6.79E-10	9.168301244	24	231	9	21698	*HLA-DQA1,HLA-DQB1,HLA-DRB5,HLA-DOB,HLA-DMA,HLA-DRA,HLA-DMB,HLA-E,HLA-G*
GO:CC	MHC class II protein complex	GO:0042613	9.98E-08	7.001018832	17	231	7	21698	*HLA-DQA1,HLA-DQB1,HLA-DRB5,HLA-DOB,HLA-DMA,HLA-DRA,HLA-DMB*
GO:CC	lumenal side of endoplasmic reticulum membrane	GO:0098553	2.32E-03	2.63440666	25	231	5	21698	*HLA-DQA1,HLA-DRB5,HLA-DRA,HLA-E,HLA-G*
GO:CC	integral component of lumenal side of endoplasmic reticulum membrane	GO:0071556	2.32E-03	2.63440666	25	231	5	21698	*HLA-DQA1,HLA-DRB5,HLA-DRA,HLA-E,HLA-G*
GO:CC	lumenal side of membrane	GO:0098576	8.28E-03	2.082114665	32	231	5	21698	*HLA-DQA1,HLA-DRB5,HLA-DRA,HLA-E,HLA-G*

GO:CC	DNA packaging complex	GO:0044815	2.36E-02	1.627909516	200	231	10	21698	*HIST1H2BN,HIST1H4J,HIST1H2BK,HIST1H2BJ,HIST1H2AC,HIST1H2BD,HIST1H2AB,SMARCC2,ACD,TOP3B*
KEGG	Autoimmune thyroid disease	KEGG:05320	7.50E-09	8.125074215	49	112	11	7559	*HLA-DQB1,HLA-DRB5,HLA-DOB,HLA-DMA,HLA-DRA,HLA-DMB,HLA-E,HLA-G,IFNA2,IFNA6,IFNA14*
KEGG	Systemic lupus erythematosus	KEGG:05322	3.71E-08	7.430847613	127	112	15	7559	*HLA-DQB1,HLA-DRB5,HLA-DOB,C4B,C4A,HLA-DMA,HLA-DRA,HIST1H2BN,HIST1H4J,HIST1H2BK,HIST1H2BJ,HIST1H2AC,HIST1H2BD,HIST1H2AB,HLA-DMB*
KEGG	Antigen processing and presentation	KEGG:04612	2.65E-07	6.577198911	67	112	11	7559	*HLA-DQB1,HLA-DRB5,TAP1,HLA-DOB,HSPA1L,HSPA1B,HLA-DMA,HLA-DRA,HLA-DMB,HLA-E,HLA-G*
KEGG	Type I diabetes mellitus	KEGG:04940	4.16E-07	6.380743752	40	112	9	7559	*HLA-DQB1,HLA-DRB5,HLA-DOB,LTA,HLA-DMA,HLA-DRA,HLA-DMB,HLA-E,HLA-G*
KEGG	Toxoplasmosis	KEGG:05145	4.16E-07	6.380682943	107	112	13	7559	*STAT1,NFKB1,HLA-DQB1,HLA-DRB5,HLA-DOB,HSPA1L,HSPA1B,HLA-DMA,HLA-DRA,HLA-DMB,PIK3R5,IFNGR2,MAPK1*
KEGG	Influenza A	KEGG:05164	1.23E-06	5.911361394	163	112	15	7559	*PIK3CD,STAT1,TLR3,NFKB1,HLA-DQB1,HLA-DRB5,HLA-DOB,HLA-DMA,HLA-DRA,HLA-DMB,IFNA2,IFNA6,IFNA14,IFNGR2,MAPK1*
KEGG	Epstein-Barr virus infection	KEGG:05169	1.50E-06	5.823230955	191	112	16	7559	*RUNX3,PIK3CD,STAT1,NFKB1,HLA-DQB1,HLA-DRB5,TAP1,HLA-DOB,HLA-DMA,HLA-DRA,HLA-DMB,HLA-E,HLA-G,IFNA2,IFNA6,IFNA14*
KEGG	Allograft rejection	KEGG:05330	2.14E-06	5.670327852	34	112	8	7559	*HLA-DQB1,HLA-DRB5,HLA-DOB,HLA-DMA,HLA-DRA,HLA-DMB,HLA-E,HLA-G*
KEGG	Tuberculosis	KEGG:05152	2.76E-06	5.559561339	173	112	15	7559	*STAT1,NFKB1,HLA-DQB1,HLA-DRB5,HLA-DOB,HLA-DMA,HLA-DRA,HLA-DMB,IFNA2,IFNA6,IFNA14,RAB5B,ATP6V0D1,IFNGR2,MAPK1*

KEGG	Th1 and Th2 cell differentiation	KEGG:04658	3.98E-06	5.399625617	86	112	11	7559	*RUNX3,STAT1,NFKB1,HLA-DQB1,HLA-DRB5,HLA-DOB,HLA-DMA,HLA-DRA,HLA-DMB,IFNGR2,MAPK1*
KEGG	Graft-versus-host disease	KEGG:05332	4.38E-06	5.358706747	37	112	8	7559	*HLA-DQB1,HLA-DRB5,HLA-DOB,HLA-DMA,HLA-DRA,HLA-DMB,HLA-E,HLA-G*
KEGG	Human T-cell leukemia virus 1 infection	KEGG:05166	6.16E-06	5.210144683	211	112	16	7559	*PIK3CD,NFKB1,HLA-DQB1,HLA-DRB5,HLA-DOB,ATF6B,LTA,HLA-DMA,HLA-DRA,HLA-DMB,HLA-E,HLA-G,IL15RA,SPI1,ANAPC7,MAPK1*
KEGG	Leishmaniasis	KEGG:05140	6.61E-06	5.179569274	71	112	10	7559	*STAT1,NFKB1,HLA-DQB1,HLA-DRB5,HLA-DOB,HLA-DMA,HLA-DRA,HLA-DMB,IFNGR2,MAPK1*
KEGG	Inflammatory bowel disease	KEGG:05321	2.04E-05	4.691129951	61	112	9	7559	*STAT1,NFKB1,HLA-DQB1,HLA-DRB5,HLA-DOB,HLA-DMA,HLA-DRA,HLA-DMB,IFNGR2*
KEGG	Phagosome	KEGG:04145	6.54E-05	4.18411597	137	112	12	7559	*HLA-DQB1,HLA-DRB5,TAP1,HLA-DOB,ATP6V1G2,HLA-DMA,HLA-DRA,HLA-DMB,HLA-E,HLA-G,RAB5B,ATP6V0D1*
KEGG	Viral carcinogenesis	KEGG:05203	7.98E-05	4.097812804	194	112	14	7559	*PIK3CD,CCR8,NFKB1,ATF6B,HIST1H2BN,HIST1H4J,HIST1H2BK,HIST1H2BJ,HIST1H2BD,HLA-E,MRPS18B,HLA-G,ATP6V0D1,MAPK1*
KEGG	Viral myocarditis	KEGG:05416	1.11E-04	3.956030035	55	112	8	7559	*HLA-DQB1,HLA-DRB5,HLA-DOB,HLA-DMA,HLA-DRA,HLA-DMB,HLA-E,HLA-G*
KEGG	Asthma	KEGG:05310	1.91E-04	3.719551312	27	112	6	7559	*HLA-DQB1,HLA-DRB5,HLA-DOB,HLA-DMA,HLA-DRA,HLA-DMB*
KEGG	Th17 cell differentiation	KEGG:04659	2.49E-04	3.604557592	104	112	10	7559	*STAT1,NFKB1,HLA-DQB1,HLA-DRB5,HLA-DOB,HLA-DMA,HLA-DRA,HLA-DMB,IFNGR2,MAPK1*
KEGG	Intestinal immune network for IgA production	KEGG:04672	2.84E-04	3.546411226	44	112	7	7559	*HLA-DQB1,HLA-DRB5,HLA-DOB,HLA-DMA,HLA-DRA,HLA-DMB,IL15RA*
KEGG	Rheumatoid arthritis	KEGG:05323	3.97E-04	3.401604839	86	112	9	7559	*CTSK,HLA-DQB1,HLA-DRB5,HLA-DOB,ATP6V1G2,HLA-DMA,HLA-DRA,HLA-DMB,ATP6V0D1*

KEGG	Toll-like receptor signaling pathway	KEGG:04620	1.38E-03	2.859964232	100	112	9	7559	*CTSK,PIK3CD,STAT1,TLR3,NFKB1,IFNA2,IFNA6,IFNA14,MAPK1*
KEGG	Kaposi sarcoma-associated herpesvirus infection	KEGG:05167	2.09E-03	2.680465012	191	112	12	7559	*PIK3CD,STAT1,CCR8,TLR3,NFKB1,HLA-E,HLA-G,IFNA2,IFNA6,IFNA14,PIK3R5,MAPK1*
KEGG	Staphylococcus aureus infection	KEGG:05150	2.77E-03	2.55676837	84	112	8	7559	*HLA-DQB1,HLA-DRB5,HLA-DOB,C4B,C4A,HLA-DMA,HLA-DRA,HLA-DMB*
KEGG	Coronavirus disease - COVID-19	KEGG:05171	4.62E-03	2.335221121	207	112	12	7559	*PIK3CD,STAT1,TLR3,NFKB1,C4B,C4A,IFNA2,IFNA6,IFNA14,RPL6,RPL41,MAPK1*
KEGG	Human papillomavirus infection	KEGG:05165	5.12E-03	2.290816259	315	112	15	7559	*PIK3CD,STAT1,TLR3,NFKB1,TNXB,ATP6V1G2,HLA-E,HLA-G,PTK2,IFNA2,IFNA6,IFNA14,ATP6V0D1,WNT3,MAPK1*
KEGG	Human immunodeficiency virus 1 infection	KEGG:05170	2.08E-02	1.681466733	207	112	11	7559	*PIK3CD,NFKB1,TAP1,HLA-E,HLA-G,PTK2,IFNA2,IFNA6,IFNA14,MAPK1,LIMK2*
KEGG	Cell adhesion molecules	KEGG:04514	2.28E-02	1.642637903	143	112	9	7559	*HLA-DQB1,HLA-DRB5,HLA-DOB,HLA-DMA,HLA-DRA,HLA-DMB,HLA-E,HLA-G,CD226*
KEGG	Alcoholism	KEGG:05034	2.48E-02	1.605584125	177	112	10	7559	*ATF6B,HIST1H2BN,HIST1H4J,HIST1H2BK,HIST1H2BJ,HIST1H2AC,HIST1H2BD,HIST1H2AB,PPP1CC,MAPK1*
KEGG	Human cytomegalovirus infection	KEGG:05163	2.89E-02	1.538883593	215	112	11	7559	*PIK3CD,NFKB1,TAP1,ATF6B,HLA-E,HLA-G,PTK2,IFNA2,IFNA6,IFNA14,MAPK1*
KEGG	Natural killer cell mediated cytotoxicity	KEGG:04650	2.90E-02	1.538112416	117	112	8	7559	*PIK3CD,HLA-E,HLA-G,IFNA2,IFNA6,IFNA14,IFNGR2,MAPK1*
KEGG	Neutrophil extracellular trap formation	KEGG:04613	3.39E-02	1.470331265	184	112	10	7559	*PIK3CD,NFKB1,HIST1H2BN,HIST1H4J,HIST1H2BK,HIST1H2BJ,HIST1H2AC,HIST1H2BD,HIST1H2AB,MAPK1*
KEGG	Necroptosis	KEGG:04217	3.59E-02	1.445438662	152	112	9	7559	*FAF1,STAT1,TLR3,HIST1H2AC,HIST1H2AB,IFNA2,IFNA6,IFNA14,IFNGR2*
KEGG	JAK-STAT signaling pathway	KEGG:04630	4.99E-02	1.302152796	159	112	9	7559	*MCL1,PIK3CD,STAT1,IFNA2,IFNA6,IFNA14,IL15RA,PTPN2,IFNGR2*

KEGG	Hepatitis B	KEGG:05161	4.99E-02	1.302152796	159	112	9	7559	*PIK3CD,STAT1,TLR3,NFKB1,ATF6B,IFNA2,IFNA6,IFNA14,MAPK1*
REAC	Recognition and association of DNA glycosylase with site containing an affected pyrimidine	REAC:R-HSA-110328	5.93E-05	4.226652113	60	153	9	10770	*HIST1H2BN,HIST1H4J,HIST1H2BK,HIST1H2BJ,HIST1H2AC,HIST1H2BD,HIST1H2AB,NEIL2,ACD*
REAC	Depyrimidination	REAC:R-HSA-73928	5.93E-05	4.226652113	60	153	9	10770	*HIST1H2BN,HIST1H4J,HIST1H2BK,HIST1H2BJ,HIST1H2AC,HIST1H2BD,HIST1H2AB,NEIL2,ACD*
REAC	Cleavage of the damaged pyrimidine	REAC:R-HSA-110329	5.93E-05	4.226652113	60	153	9	10770	*HIST1H2BN,HIST1H4J,HIST1H2BK,HIST1H2BJ,HIST1H2AC,HIST1H2BD,HIST1H2AB,NEIL2,ACD*
REAC	Base-Excision Repair, AP Site Formation	REAC:R-HSA-73929	6.88E-05	4.162575461	61	153	9	10770	*HIST1H2BN,HIST1H4J,HIST1H2BK,HIST1H2BJ,HIST1H2AC,HIST1H2BD,HIST1H2AB,NEIL2,ACD*
REAC	Packaging Of Telomere Ends	REAC:R-HSA-171306	2.45E-04	3.611158788	52	153	8	10770	*HIST1H2BN,HIST1H4J,HIST1H2BK,HIST1H2BJ,HIST1H2AC,HIST1H2BD,HIST1H2AB,ACD*
REAC	Cleavage of the damaged purine	REAC:R-HSA-110331	3.82E-04	3.41783861	55	153	8	10770	*HIST1H2BN,HIST1H4J,HIST1H2BK,HIST1H2BJ,HIST1H2AC,HIST1H2BD,HIST1H2AB,ACD*
REAC	Depurination	REAC:R-HSA-73927	3.82E-04	3.41783861	55	153	8	10770	*HIST1H2BN,HIST1H4J,HIST1H2BK,HIST1H2BJ,HIST1H2AC,HIST1H2BD,HIST1H2AB,ACD*
REAC	Recognition and association of DNA glycosylase with site containing an affected purine	REAC:R-HSA-110330	3.82E-04	3.41783861	55	153	8	10770	*HIST1H2BN,HIST1H4J,HIST1H2BK,HIST1H2BJ,HIST1H2AC,HIST1H2BD,HIST1H2AB,ACD*
REAC	Meiotic synapsis	REAC:R-HSA-1221632	5.27E-04	3.27793165	77	153	9	10770	*SYCP1,HIST1H2BN,HIST1H4J,HIST1H2BK,HIST1H2BJ,HIST1H2AC,HIST1H2BD,HIST1H2AB,ACD*
REAC	Interferon Signaling	REAC:R-HSA-913531	1.40E-03	2.853719738	193	153	13	10770	*STAT1,FLNB,HLA-DQA1,HLA-DRB5,HLA-DRA,TRIM38,HLA-E,TRIM31,HLA-G,IFNA2,IFNA6,IFNA14,IFNGR2*

REAC	Senescence-Associated Secretory Phenotype (SASP)	REAC:R-HSA-2559582	1.63E-03	2.786857843	112	153	10	10770	*NFKB1,HIST1H2BN,HIST1H4J,HIST1H2BK,HIST1H2BJ,HIST1H2AC,HIST1H2BD,HIST1H2AB,ANAPC7,MAPK1*
REAC	Inhibition of DNA recombination at telomere	REAC:R-HSA-9670095	1.77E-03	2.751461326	67	153	8	10770	*HIST1H2BN,HIST1H4J,HIST1H2BK,HIST1H2BJ,HIST1H2AC,HIST1H2BD,HIST1H2AB,ACD*
REAC	Transcriptional regulation of granulopoiesis	REAC:R-HSA-9616222	1.80E-03	2.745341422	89	153	9	10770	*HIST1H2BN,HIST1H4J,HIST1H2BK,HIST1H2BJ,HIST1H2AC,HIST1H2BD,HIST1H2AB,SPI1,CBFB*
REAC	Base Excision Repair	REAC:R-HSA-73884	1.80E-03	2.745341422	89	153	9	10770	*HIST1H2BN,HIST1H4J,HIST1H2BK,HIST1H2BJ,HIST1H2AC,HIST1H2BD,HIST1H2AB,NEIL2,ACD*
REAC	ERCC6 (CSB) and EHMT2 (G9a) positively regulate rRNA expression	REAC:R-HSA-427389	4.60E-03	2.337276093	76	153	8	10770	*HIST1H2BN,HIST1H4J,HIST1H2BK,HIST1H2BJ,HIST1H2AC,HIST1H2BD,HIST1H2AB,GATAD2A*
REAC	DNA Damage/Telomere Stress Induced Senescence	REAC:R-HSA-2559586	6.74E-03	2.171324217	80	153	8	10770	*HIST1H2BN,HIST1H4J,HIST1H2BK,HIST1H2BJ,HIST1H2AC,HIST1H2BD,HIST1H2AB,ACD*
REAC	Pre-NOTCH Expression and Processing	REAC:R-HSA-1912422	9.47E-03	2.023541222	109	153	9	10770	*POGLUT1,HIST1H2BN,HIST1H4J,HIST1H2BK,HIST1H2BJ,HIST1H2AC,HIST1H2BD,HIST1H2AB,ATP2A2*
REAC	RNA Polymerase I Promoter Opening	REAC:R-HSA-73728	1.16E-02	1.936753703	63	153	7	10770	*HIST1H2BN,HIST1H4J,HIST1H2BK,HIST1H2BJ,HIST1H2AC,HIST1H2BD,HIST1H2AB*
REAC	Interferon gamma signaling	REAC:R-HSA-877300	1.25E-02	1.903275821	87	153	8	10770	*HLA-DQA1,HLA-DRB5,HLA-DRA,TRIM38,HLA-E,TRIM31,HLA-G,IFNGR2*
REAC	DNA methylation	REAC:R-HSA-5334118	1.42E-02	1.847262757	65	153	7	10770	*HIST1H2BN,HIST1H4J,HIST1H2BK,HIST1H2BJ,HIST1H2AC,HIST1H2BD,HIST1H2AB*
REAC	Meiosis	REAC:R-HSA-1500620	1.67E-02	1.778239702	117	153	9	10770	*SYCP1,HIST1H2BN,HIST1H4J,HIST1H2BK,HIST1H2BJ,HIST1H2AC,HIST1H2BD,HIST1H2AB,ACD*
REAC	Formation of the beta-catenin:TCF transactivating complex	REAC:R-HSA-201722	1.73E-02	1.761376398	91	153	8	10770	*RUNX3,HIST1H2BN,HIST1H4J,HIST1H2BK,HIST1H2BJ,HIST1H2AC,HIST1H2BD,HIST1H2AB*

REAC	Activated PKN1 stimulates transcription of AR (androgen receptor) regulated genes KLK2 and KLK3	REAC:R-HSA-5625886	1.73E-02	1.760943964	67	153	7	10770	*HIST1H2BN,HIST1H4J,HIST1H2BK,HIST1H2BJ,HIST1H2AC,HIST1H2BD,HIST1H2AB*
REAC	SIRT1 negatively regulates rRNA expression	REAC:R-HSA-427359	1.91E-02	1.718912152	68	153	7	10770	*HIST1H2BN,HIST1H4J,HIST1H2BK,HIST1H2BJ,HIST1H2AC,HIST1H2BD,HIST1H2AB*
REAC	Activation of anterior HOX genes in hindbrain development during early embryogenesis	REAC:R-HSA-5617472	2.03E-02	1.691463848	120	153	9	10770	*HIST1H2BN,HIST1H4J,HIST1H2BK,HIST1H2BJ,HIST1H2AC,HIST1H2BD,HIST1H2AB,CTCF,HOXB3*
REAC	Activation of HOX genes during differentiation	REAC:R-HSA-5619507	2.03E-02	1.691463848	120	153	9	10770	*HIST1H2BN,HIST1H4J,HIST1H2BK,HIST1H2BJ,HIST1H2AC,HIST1H2BD,HIST1H2AB,CTCF,HOXB3*
REAC	HDACs deacetylate histones	REAC:R-HSA-3214815	2.19E-02	1.659761587	94	153	8	10770	*HIST1H2BN,HIST1H4J,HIST1H2BK,HIST1H2BJ,HIST1H2AC,HIST1H2BD,HIST1H2AB,GATAD2A*
REAC	RHO GTPases activate PKNs	REAC:R-HSA-5625740	2.19E-02	1.659761587	94	153	8	10770	*HIST1H2BN,HIST1H4J,HIST1H2BK,HIST1H2BJ,HIST1H2AC,HIST1H2BD,HIST1H2AB,MYL6*
REAC	RUNX1 regulates genes involved in megakaryocyte differentiation and platelet function	REAC:R-HSA-8936459	2.74E-02	1.56196932	97	153	8	10770	*HIST1H2BN,HIST1H4J,HIST1H2BK,HIST1H2BJ,HIST1H2AC,HIST1H2BD,HIST1H2AB,CBFB*
REAC	Deposition of new CENPA-containing nucleosomes at the centromere	REAC:R-HSA-606279	3.03E-02	1.519146278	73	153	7	10770	*HIST1H2BN,HIST1H4J,HIST1H2BK,HIST1H2BJ,HIST1H2AC,HIST1H2BD,HIST1H2AB*
REAC	Defective pyroptosis	REAC:R-HSA-9710421	3.03E-02	1.519146278	73	153	7	10770	*HIST1H2BN,HIST1H4J,HIST1H2BK,HIST1H2BJ,HIST1H2AC,HIST1H2BD,HIST1H2AB*
REAC	Condensation of Prophase Chromosomes	REAC:R-HSA-2299718	3.03E-02	1.519146278	73	153	7	10770	*HIST1H2BN,HIST1H4J,HIST1H2BK,HIST1H2BJ,HIST1H2AC,HIST1H2BD,HIST1H2AB*

REAC	Nucleosome assembly	REAC:R-HSA-774815	3.03E-02	1.519146278	73	153	7	10770	*HIST1H2BN,HIST1H4J,HIST1H2BK,HIST1H2BJ,HIST1H2AC,HIST1H2BD,HIST1H2AB*
REAC	PRC2 methylates histones and DNA	REAC:R-HSA-212300	3.03E-02	1.519146278	73	153	7	10770	*HIST1H2BN,HIST1H4J,HIST1H2BK,HIST1H2BJ,HIST1H2AC,HIST1H2BD,HIST1H2AB*
REAC	RUNX1 regulates transcription of genes involved in differentiation of HSCs	REAC:R-HSA-8939236	3.80E-02	1.420385273	130	153	9	10770	*HIST1H2BN,HIST1H4J,HIST1H2BK,HIST1H2BJ,HIST1H2AC,HIST1H2BD,HIST1H2AB,SPI1,CBFB*
REAC	Cellular Senescence	REAC:R-HSA-2559583	4.12E-02	1.385530099	195	153	11	10770	*NFKB1,HIST1H2BN,HIST1H4J,HIST1H2BK,HIST1H2BJ,HIST1H2AC,HIST1H2BD,HIST1H2AB,ANAPC7,ACD,MAPK1*
REAC	Transcriptional regulation by small RNAs	REAC:R-HSA-5578749	4.20E-02	1.376905572	103	153	8	10770	*HIST1H2BN,HIST1H4J,HIST1H2BK,HIST1H2BJ,HIST1H2AC,HIST1H2BD,HIST1H2AB,IPO8*
REAC	Positive epigenetic regulation of rRNA expression	REAC:R-HSA-5250913	4.81E-02	1.318104895	105	153	8	10770	*HIST1H2BN,HIST1H4J,HIST1H2BK,HIST1H2BJ,HIST1H2AC,HIST1H2BD,HIST1H2AB,GATAD2A*
WP	Ebola virus infection in host	WP:WP4217	1.29E-05	4.888675309	126	117	13	7364	*PIK3CD,STAT1,FLNB,NFKB1,HLA-DQB1,HLA-DRB5,HLA-DOB,HLA-DMA,HLA-DRA,HLA-DMB,HLA-E,HLA-G,MAPK1*
WP	Allograft rejection	WP:WP2328	1.48E-05	4.828873928	86	117	11	7364	*STAT1,HLA-DQB1,HLA-DRB5,HLA-DOB,C4B,C4A,HLA-DMA,HLA-DRA,HLA-DMB,HLA-E,HLA-G*
WP	Toll-like receptor signaling pathway	WP:WP75	5.54E-03	2.256284575	103	117	9	7364	*PIK3CD,STAT1,TLR3,NFKB1,IFNA2,IFNA6,IFNA14,PIK3R5,MAPK1*
WP	SARS coronavirus and innate immunity	WP:WP4912	1.85E-02	1.733256037	31	117	5	7364	*STAT1,TLR3,IFNA2,IFNA6,IFNA14*
WP	RANKL/RANK signaling pathway	WP:WP2018	3.19E-02	1.495584171	54	117	6	7364	*CTSK,STAT1,NFKB1,PTK2,SPI1,MAPK1*
WP	Prion disease pathway	WP:WP3995	3.36E-02	1.474096129	35	117	5	7364	*NFKB1,PTK2,SPI1,CTCF,MAPK1*
WP	Overview of interferons-	WP:WP4558	3.36E-02	1.474096129	35	117	5	7364	*STAT1,IFNA2,IFNA6,IFNA14,IFNGR2*

	mediated signaling pathway								
WP	Type II interferon signaling	WP:WP619	3.85E-02	1.414653844	36	117	5	7364	*STAT1,TAP1,IFNA2,SPI1,IFNGR2*
WP	Immune response to tuberculosis	WP:WP4197	4.79E-02	1.319899218	21	117	4	7364	*STAT1,TAP1,PTPN2,IFNGR2*
MIRNA	hsa-miR-296-3p	MIRNA:hsa-miR-296-3p	1.11E-02	1.956039092	233	184	13	13650	*RPRD2,LYPLA2,CTSK,SETDB1,VARS,HSPA1B,BRD2,HIST1H2BD,HLA-E,ATAT1,SPI1,VMP1,PLAGL2*

Supplementary Table S16. Pathways associated with headache and hyperthyroidism.

**source**	**term_name**	**term_id**	**adjusted_ p_value**	**negative_log10_ of_adjusted_ p_value**	**term_size**	**query_size**	**Intersection _size**	**effective_ domain_size**	**Intersections**
GO:MF	MHC class II protein complex binding	GO:0023026	3.50E-07	6.456247707	25	84	6	20183	*HLA-DQA1,HLA-DRB5,HLA-DMA,HLA-DMB,HLA-DOB,HLA-DRA*
GO:MF	sialic acid transmembrane transporter activity	GO:0015136	6.75E-07	6.170434336	5	84	4	20183	*SLC17A2,SLC17A3,SLC17A1,SLC17A4*
GO:MF	MHC protein complex binding	GO:0023023	2.13E-06	5.671620483	33	84	6	20183	*HLA-DQA1,HLA-DRB5,HLA-DMA,HLA-DMB,HLA-DOB,HLA-DRA*
GO:MF	sodium:phosphate symporter activity	GO:0005436	4.37E-05	4.359165366	11	84	4	20183	*SLC17A2,SLC17A3,SLC17A1,SLC17A4*
GO:MF	peptide antigen binding	GO:0042605	1.16E-04	3.934182198	33	84	5	20183	*HLA-DQA1,HLA-DRB5,HLA-B,HLA-C,HLA-DRA*
GO:MF	MHC class II receptor activity	GO:0032395	1.86E-03	2.729945318	8	84	3	20183	*HLA-DQA1,HLA-DOB,HLA-DRA*
GO:MF	peptide binding	GO:0042277	2.38E-02	1.623018991	314	84	8	20183	*GPR149,HLA-DQA1,HLA-DRB5,HLA-B,HLA-C,POM121L2,HLA-DRA,BRAP*
GO:MF	carbohydrate derivative transmembrane transporter activity	GO:1901505	4.22E-02	1.374584178	56	84	4	20183	*SLC17A2,SLC17A3,SLC17A1,SLC17A4*
GO:BP	antigen processing and	GO:0048002	2.22E-09	8.654209781	82	84	10	21100	*HLA-DQA1,HLA-DRB5,HLA-B,PSMB9,HLA-DMA,HLA-DMB,HLA-DOB,HLA-C,HFE,HLA-DRA*

	presentation of peptide antigen								
GO:BP	antigen processing and presentation of exogenous peptide antigen	GO:0002478	3.93E-09	8.405921051	59	84	9	21100	*HLA-DQA1,HLA-DRB5,HLA-B,PSMB9,HLA-DMA,HLA-DMB,HLA-DOB,HLA-C,HLA-DRA*
GO:BP	antigen processing and presentation	GO:0019882	7.68E-09	8.114826509	128	84	11	21100	*HLA-DQA1,HLA-DQB1,HLA-DRB5,HLA-B,PSMB9,HLA-DMA,HLA-DMB,HLA-DOB,HLA-C,HFE,HLA-DRA*
GO:BP	antigen processing and presentation of exogenous antigen	GO:0019884	1.96E-08	7.707291646	70	84	9	21100	*HLA-DQA1,HLA-DRB5,HLA-B,PSMB9,HLA-DMA,HLA-DMB,HLA-DOB,HLA-C,HLA-DRA*
GO:BP	peptide antigen assembly with MHC class II protein complex	GO:0002503	2.21E-08	7.65593843	14	84	6	21100	*HLA-DQA1,HLA-DRB5,HLA-DMA,HLA-DMB,HLA-DOB,HLA-DRA*
GO:BP	MHC class II protein complex assembly	GO:0002399	2.21E-08	7.65593843	14	84	6	21100	*HLA-DQA1,HLA-DRB5,HLA-DMA,HLA-DMB,HLA-DOB,HLA-DRA*
GO:BP	peptide antigen assembly with MHC protein complex	GO:0002501	1.35E-07	6.870335597	18	84	6	21100	*HLA-DQA1,HLA-DRB5,HLA-DMA,HLA-DMB,HLA-DOB,HLA-DRA*
GO:BP	MHC protein complex assembly	GO:0002396	1.35E-07	6.870335597	18	84	6	21100	*HLA-DQA1,HLA-DRB5,HLA-DMA,HLA-DMB,HLA-DOB,HLA-DRA*
GO:BP	antigen processing and presentation of peptide or polysaccharide antigen via MHC class II	GO:0002504	2.12E-07	6.673252449	36	84	7	21100	*HLA-DQA1,HLA-DQB1,HLA-DRB5,HLA-DMA,HLA-DMB,HLA-DOB,HLA-DRA*
GO:BP	sialic acid transport	GO:0015739	2.65E-06	5.577152284	5	84	4	21100	*SLC17A2,SLC17A3,SLC17A1,SLC17A4*
GO:BP	antigen processing and presentation of exogenous peptide antigen via MHC class II	GO:0019886	2.65E-06	5.576746449	28	84	6	21100	*HLA-DQA1,HLA-DRB5,HLA-DMA,HLA-DMB,HLA-DOB,HLA-DRA*

GO:BP	antigen processing and presentation of peptide antigen via MHC class II	GO:0002495	6.29E-06	5.201079374	32	84	6	21100	*HLA-DQA1,HLA-DRB5,HLA-DMA,HLA-DMB,HLA-DOB,HLA-DRA*
GO:BP	antigen processing and presentation of endogenous peptide antigen	GO:0002483	1.97E-03	2.706199952	19	84	4	21100	*HLA-B,HLA-C,HFE,HLA-DRA*
GO:BP	antigen processing and presentation of endogenous antigen	GO:0019883	7.43E-03	2.129164643	26	84	4	21100	*HLA-B,HLA-C,HFE,HLA-DRA*
GO:BP	protein-DNA complex subunit organization	GO:0071824	2.70E-02	1.568949727	269	84	8	21100	*PSMB9,BRD2,HIST1H4J,HIST1H2BK,HIST1H2BD,HIST1H1E,SMARCC2,RRN3*
GO:BP	antigen processing and presentation of endogenous peptide antigen via MHC class I	GO:0019885	3.84E-02	1.415216071	13	84	3	21100	*HLA-B,HLA-C,HFE*
GO:CC	MHC protein complex	GO:0042611	5.61E-14	13.25096891	24	85	9	21698	*HLA-DQA1,HLA-DQB1,HLA-DRB5,HLA-B,HLA-DMA,HLA-DMB,HLA-DOB,HLA-C,HLA-DRA*
GO:CC	MHC class II protein complex	GO:0042613	6.64E-11	10.17769087	17	85	7	21698	*HLA-DQA1,HLA-DQB1,HLA-DRB5,HLA-DMA,HLA-DMB,HLA-DOB,HLA-DRA*
GO:CC	integral component of lumenal side of endoplasmic reticulum membrane	GO:0071556	1.31E-05	4.882359967	25	85	5	21698	*HLA-DQA1,HLA-DRB5,HLA-B,HLA-C,HLA-DRA*
GO:CC	lumenal side of endoplasmic reticulum membrane	GO:0098553	1.31E-05	4.882359967	25	85	5	21698	*HLA-DQA1,HLA-DRB5,HLA-B,HLA-C,HLA-DRA*
GO:CC	lumenal side of membrane	GO:0098576	4.86E-05	4.313038499	32	85	5	21698	*HLA-DQA1,HLA-DRB5,HLA-B,HLA-C,HLA-DRA*
GO:CC	ER to Golgi transport vesicle membrane	GO:0012507	9.37E-04	3.028485126	57	85	5	21698	*HLA-DQA1,HLA-DRB5,HLA-B,HLA-C,HLA-DRA*

GO:CC	DNA packaging complex	GO:0044815	4.38E-03	2.358061846	200	85	7	21698	*HIST1H4J,HIST1H2BK,HIST1H2BD,HIST1H2AC,HIST1H1E,SMARCC2,TOP3B*
GO:CC	transport vesicle membrane	GO:0030658	5.48E-03	2.261460301	207	85	7	21698	*HLA-DQA1,HLA-DRB5,HLA-B,HLA-C,PRRT1,SCGN,HLA-DRA*
GO:CC	COPII-coated ER to Golgi transport vesicle	GO:0030134	8.88E-03	2.05140611	90	85	5	21698	*HLA-DQA1,HLA-DRB5,HLA-B,HLA-C,HLA-DRA*
GO:CC	integral component of endoplasmic reticulum membrane	GO:0030176	1.41E-02	1.851557629	163	85	6	21698	*HLA-DQA1,HLA-DRB5,ATF6B,HLA-B,HLA-C,HLA-DRA*
GO:CC	intrinsic component of endoplasmic reticulum membrane	GO:0031227	1.84E-02	1.735606773	171	85	6	21698	*HLA-DQA1,HLA-DRB5,ATF6B,HLA-B,HLA-C,HLA-DRA*
KEGG	Type I diabetes mellitus	KEGG:04940	7.82E-13	12.10692565	40	29	9	7559	*HLA-DQB1,HLA-DRB5,LTA,HLA-B,HLA-DMA,HLA-DMB,HLA-DOB,HLA-C,HLA-DRA*
KEGG	Systemic lupus erythematosus	KEGG:05322	8.27E-13	12.08235807	127	29	12	7559	*HLA-DQB1,HLA-DRB5,C4B,HLA-DMA,HLA-DMB,C4A,HLA-DOB,HIST1H4J,HIST1H2BK,HIST1H2BD,HIST1H2AC,HLA-DRA*
KEGG	Allograft rejection	KEGG:05330	1.88E-11	10.72477263	34	29	8	7559	*HLA-DQB1,HLA-DRB5,HLA-B,HLA-DMA,HLA-DMB,HLA-DOB,HLA-C,HLA-DRA*
KEGG	Graft-versus-host disease	KEGG:05332	3.98E-11	10.40035265	37	29	8	7559	*HLA-DQB1,HLA-DRB5,HLA-B,HLA-DMA,HLA-DMB,HLA-DOB,HLA-C,HLA-DRA*
KEGG	Antigen processing and presentation	KEGG:04612	1.15E-10	9.94087964	67	29	9	7559	*HLA-DQB1,HLA-DRB5,HLA-B,HLA-DMA,HLA-DMB,HLA-DOB,HSPA1B,HLA-C,HLA-DRA*
KEGG	Autoimmune thyroid disease	KEGG:05320	4.51E-10	9.345813303	49	29	8	7559	*HLA-DQB1,HLA-DRB5,HLA-B,HLA-DMA,HLA-DMB,HLA-DOB,HLA-C,HLA-DRA*
KEGG	Viral myocarditis	KEGG:05416	1.20E-09	8.920950472	55	29	8	7559	*HLA-DQB1,HLA-DRB5,HLA-B,HLA-DMA,HLA-DMB,HLA-DOB,HLA-C,HLA-DRA*
KEGG	Asthma	KEGG:05310	3.50E-08	7.456331623	27	29	6	7559	*HLA-DQB1,HLA-DRB5,HLA-DMA,HLA-DMB,HLA-DOB,HLA-DRA*
KEGG	Staphylococcus aureus infection	KEGG:05150	4.00E-08	7.398345885	84	29	8	7559	*HLA-DQB1,HLA-DRB5,C4B,HLA-DMA,HLA-DMB,C4A,HLA-DOB,HLA-DRA*
KEGG	Human T-cell leukemia virus 1 infection	KEGG:05166	1.99E-07	6.701527849	211	29	10	7559	*HLA-DQB1,HLA-DRB5,ATF6B,LTA,HLA-B,HLA-DMA,HLA-DMB,HLA-DOB,HLA-C,HLA-DRA*
KEGG	Intestinal immune network for IgA production	KEGG:04672	7.98E-07	6.098221472	44	29	6	7559	*HLA-DQB1,HLA-DRB5,HLA-DMA,HLA-DMB,HLA-DOB,HLA-DRA*

KEGG	Phagosome	KEGG:04145	2.01E-06	5.697449836	137	29	8	7559	*HLA-DQB1,HLA-DRB5,HLA-B,HLA-DMA,HLA-DMB,HLA-DOB,HLA-C,HLA-DRA*
KEGG	Cell adhesion molecules	KEGG:04514	2.81E-06	5.551187472	143	29	8	7559	*HLA-DQB1,HLA-DRB5,HLA-B,HLA-DMA,HLA-DMB,HLA-DOB,HLA-C,HLA-DRA*
KEGG	Inflammatory bowel disease	KEGG:05321	6.00E-06	5.221825943	61	29	6	7559	*HLA-DQB1,HLA-DRB5,HLA-DMA,HLA-DMB,HLA-DOB,HLA-DRA*
KEGG	Toxoplasmosis	KEGG:05145	7.67E-06	5.115202006	107	29	7	7559	*HLA-DQB1,HLA-DRB5,HLA-DMA,HLA-DMB,HLA-DOB,HSPA1B,HLA-DRA*
KEGG	Leishmaniasis	KEGG:05140	1.51E-05	4.821708016	71	29	6	7559	*HLA-DQB1,HLA-DRB5,HLA-DMA,HLA-DMB,HLA-DOB,HLA-DRA*
KEGG	Epstein-Barr virus infection	KEGG:05169	2.66E-05	4.575877647	191	29	8	7559	*HLA-DQB1,HLA-DRB5,HLA-B,HLA-DMA,HLA-DMB,HLA-DOB,HLA-C,HLA-DRA*
KEGG	Th1 and Th2 cell differentiation	KEGG:04658	4.76E-05	4.322566943	86	29	6	7559	*HLA-DQB1,HLA-DRB5,HLA-DMA,HLA-DMB,HLA-DOB,HLA-DRA*
KEGG	Rheumatoid arthritis	KEGG:05323	4.76E-05	4.322566943	86	29	6	7559	*HLA-DQB1,HLA-DRB5,HLA-DMA,HLA-DMB,HLA-DOB,HLA-DRA*
KEGG	Hematopoietic cell lineage	KEGG:04640	6.66E-05	4.176669425	91	29	6	7559	*HLA-DQB1,HLA-DRB5,HLA-DMA,HLA-DMB,HLA-DOB,HLA-DRA*
KEGG	Th17 cell differentiation	KEGG:04659	1.46E-04	3.834252315	104	29	6	7559	*HLA-DQB1,HLA-DRB5,HLA-DMA,HLA-DMB,HLA-DOB,HLA-DRA*
KEGG	Influenza A	KEGG:05164	1.96E-03	2.707447325	163	29	6	7559	*HLA-DQB1,HLA-DRB5,HLA-DMA,HLA-DMB,HLA-DOB,HLA-DRA*
KEGG	Tuberculosis	KEGG:05152	2.75E-03	2.561392124	173	29	6	7559	*HLA-DQB1,HLA-DRB5,HLA-DMA,HLA-DMB,HLA-DOB,HLA-DRA*
KEGG	Viral carcinogenesis	KEGG:05203	5.21E-03	2.282780088	194	29	6	7559	*ATF6B,HLA-B,HIST1H4J,HLA-C,HIST1H2BK,HIST1H2BD*
KEGG	Alcoholism	KEGG:05034	3.40E-02	1.468495285	177	29	5	7559	*ATF6B,HIST1H4J,HIST1H2BK,HIST1H2BD,HIST1H2AC*
REAC	Interferon gamma signaling	REAC:R-HSA-877300	6.39E-05	4.194368096	87	52	7	10770	*HLA-DQA1,HLA-DRB5,HLA-B,HLA-C,TRIM38,HLA-DRA,TRIM31*
REAC	Butyrophilin (BTN) family interactions	REAC:R-HSA-8851680	8.48E-05	4.071437891	12	52	4	10770	*BTN2A2,BTN1A1,BTN3A3,BTN3A2*
REAC	Interferon Signaling	REAC:R-HSA-913531	1.29E-03	2.890683813	193	52	8	10770	*EIF4E3,HLA-DQA1,HLA-DRB5,HLA-B,HLA-C,TRIM38,HLA-DRA,TRIM31*
REAC	MHC class II antigen presentation	REAC:R-HSA-2132295	9.49E-03	2.022520507	123	52	6	10770	*HLA-DQA1,HLA-DRB5,HLA-DMA,HLA-DMB,HLA-DOB,HLA-DRA*
REAC	DNA Damage/Telomere Stress Induced Senescence	REAC:R-HSA-2559586	1.44E-02	1.842113788	80	52	5	10770	*HIST1H4J,HIST1H2BK,HIST1H2BD,HIST1H2AC,HIST1H1E*

REAC	RNA Polymerase I Promoter Escape	REAC:R-HSA-73772	2.67E-02	1.572926215	91	52	5	10770	*HIST1H4J,HIST1H2BK,HIST1H2BD,HIST1H2AC,RRN3*
REAC	Packaging Of Telomere Ends	REAC:R-HSA-171306	4.02E-02	1.39545972	52	52	4	10770	*HIST1H4J,HIST1H2BK,HIST1H2BD,HIST1H2AC*
REAC	Translocation of ZAP-70 to Immunological synapse	REAC:R-HSA-202430	4.17E-02	1.380039724	20	52	3	10770	*HLA-DQA1,HLA-DRB5,HLA-DRA*
REAC	Transcriptional regulation by RUNX1	REAC:R-HSA-8878171	4.68E-02	1.329977002	236	52	7	10770	*PSMB9,HIST1H4J,HIST1H2BK,HIST1H2BD,HIST1H2AC,BLK,SMARCC2*
WP	Allograft rejection	WP:WP2328	2.54E-10	9.594924603	86	33	10	7364	*HLA-DQB1,HLA-DRB5,C4B,HLA-B,HLA-DMA,HLA-DMB,C4A,HLA-DOB,HLA-C,HLA-DRA*
WP	Ebola virus infection in host	WP:WP4217	7.06E-06	5.151480466	126	33	8	7364	*HLA-DQB1,HLA-DRB5,HLA-B,HLA-DMA,HLA-DMB,HLA-DOB,HLA-C,HLA-DRA*

Supplementary Table S17. Pathways associated with headache and secondary hypothyroidism.

**source**	**term_name**	**term_id**	**adjusted_ p_value**	**negative_log10_ of_adjusted_ p_value**	**term_size**	**query_size**	**intersection_ size**	**effective_ domain_size**	**intersections**
GO:MF	TAP binding	GO:0046977	5.39E-07	6.268467929	9	38	4	20183	*HLA-B,TAP1,TAP2,HLA-C*
GO:MF	peptide antigen binding	GO:0042605	1.66E-06	5.780551577	33	38	5	20183	*HLA-B,TAP1,TAP2,HLA-DRB5,HLA-C*
GO:MF	MHC class II protein complex binding	GO:0023026	5.55E-03	2.255758914	25	38	3	20183	*HLA-DRB5,HLA-DMA,HLA-DOB*
GO:MF	antigen binding	GO:0003823	6.10E-03	2.214613818	168	38	5	20183	*HLA-B,TAP1,TAP2,HLA-DRB5,HLA-C*
GO:MF	MHC protein complex binding	GO:0023023	1.30E-02	1.885129244	33	38	3	20183	*HLA-DRB5,HLA-DMA,HLA-DOB*
GO:MF	ABC-type peptide antigen transporter activity	GO:0015433	1.39E-02	1.857966469	5	38	2	20183	*TAP1,TAP2*
GO:MF	ABC-type peptide transporter activity	GO:0015440	1.39E-02	1.857966469	5	38	2	20183	*TAP1,TAP2*

GO:MF	MHC class Ib protein binding	GO:0023029	2.08E-02	1.682391823	6	38	2	20183	*TAP1,TAP2*
GO:MF	TAP1 binding	GO:0046978	2.08E-02	1.682391823	6	38	2	20183	*TAP1,TAP2*
GO:BP	antigen processing and presentation	GO:0019882	1.06E-07	6.97304123	128	38	8	21100	*HLA-B,TAP1,HLA-DQB1,TAP2,HLA-DRB5,HLA-DMA,HLA-C,HLA-DOB*
GO:BP	antigen processing and presentation of peptide antigen	GO:0048002	2.03E-07	6.691437058	82	38	7	21100	*HLA-B,TAP1,TAP2,HLA-DRB5,HLA-DMA,HLA-C,HLA-DOB*
GO:BP	antigen processing and presentation of exogenous peptide antigen	GO:0002478	1.63E-06	5.786658773	59	38	6	21100	*HLA-B,TAP1,HLA-DRB5,HLA-DMA,HLA-C,HLA-DOB*
GO:BP	antigen processing and presentation of exogenous antigen	GO:0019884	4.69E-06	5.32899095	70	38	6	21100	*HLA-B,TAP1,HLA-DRB5,HLA-DMA,HLA-C,HLA-DOB*
GO:BP	antigen processing and presentation of endogenous peptide antigen via MHC class I	GO:0019885	1.09E-05	4.962814961	13	38	4	21100	*HLA-B,TAP1,TAP2,HLA-C*
GO:BP	antigen processing and presentation of endogenous peptide antigen	GO:0002483	5.86E-05	4.232098574	19	38	4	21100	*HLA-B,TAP1,TAP2,HLA-C*
GO:BP	antigen processing and presentation of endogenous antigen	GO:0019883	2.24E-04	3.649762339	26	38	4	21100	*HLA-B,TAP1,TAP2,HLA-C*
GO:BP	positive regulation of apoptotic cell clearance	GO:2000427	5.17E-04	3.286441059	8	38	3	21100	*C4B,C2,C4A*
GO:BP	antigen processing and	GO:0002504	8.71E-04	3.059852366	36	38	4	21100	*HLA-DQB1,HLA-DRB5,HLA-DMA,HLA-DOB*

	presentation of peptide or polysaccharide antigen via MHC class II								
GO:BP	regulation of apoptotic cell clearance	GO:2000425	1.11E-03	2.956528988	10	38	3	21100	*C4B,C2,C4A*
GO:BP	antigen processing and presentation of peptide antigen via MHC class I	GO:0002474	3.08E-03	2.511202794	49	38	4	21100	*HLA-B,TAP1,TAP2,HLA-C*
GO:BP	MHC class II protein complex assembly	GO:0002399	3.34E-03	2.476770851	14	38	3	21100	*HLA-DRB5,HLA-DMA,HLA-DOB*
GO:BP	peptide antigen assembly with MHC class II protein complex	GO:0002503	3.34E-03	2.476770851	14	38	3	21100	*HLA-DRB5,HLA-DMA,HLA-DOB*
GO:BP	peptide antigen assembly with MHC protein complex	GO:0002501	7.44E-03	2.128343684	18	38	3	21100	*HLA-DRB5,HLA-DMA,HLA-DOB*
GO:BP	MHC protein complex assembly	GO:0002396	7.44E-03	2.128343684	18	38	3	21100	*HLA-DRB5,HLA-DMA,HLA-DOB*
GO:BP	antigen processing and presentation of exogenous peptide antigen via MHC class II	GO:0019886	2.95E-02	1.530092236	28	38	3	21100	*HLA-DRB5,HLA-DMA,HLA-DOB*
GO:BP	antigen processing and presentation of peptide antigen via MHC class II	GO:0002495	4.45E-02	1.352114671	32	38	3	21100	*HLA-DRB5,HLA-DMA,HLA-DOB*
GO:BP	antigen processing and presentation of exogenous	GO:0042590	4.45E-02	1.352114671	32	38	3	21100	*HLA-B,TAP1,HLA-C*

	peptide antigen via MHC class I								
GO:CC	MHC protein complex	GO:0042611	9.26E-10	9.033262067	24	41	6	21698	*HLA-B,HLA-DQB1,HLA-DRB5,HLA-DMA,HLA-C,HLA-DOB*
GO:CC	integral component of endoplasmic reticulum membrane	GO:0030176	4.89E-06	5.310655198	163	41	7	21698	*ATF6B,HLA-B,TAP1,TAP2,HLA-DRB5,HLA-C,CREB3*
GO:CC	MHC class II protein complex	GO:0042613	5.83E-06	5.234450139	17	41	4	21698	*HLA-DQB1,HLA-DRB5,HLA-DMA,HLA-DOB*
GO:CC	intrinsic component of endoplasmic reticulum membrane	GO:0031227	6.81E-06	5.167090679	171	41	7	21698	*ATF6B,HLA-B,TAP1,TAP2,HLA-DRB5,HLA-C,CREB3*
GO:CC	phagocytic vesicle membrane	GO:0030670	3.05E-03	2.515190292	77	41	4	21698	*HLA-B,TAP1,TAP2,HLA-C*
GO:CC	lumenal side of endoplasmic reticulum membrane	GO:0098553	3.18E-03	2.497568361	25	41	3	21698	*HLA-B,HLA-DRB5,HLA-C*
GO:CC	integral component of lumenal side of endoplasmic reticulum membrane	GO:0071556	3.18E-03	2.497568361	25	41	3	21698	*HLA-B,HLA-DRB5,HLA-C*
GO:CC	lumenal side of membrane	GO:0098576	6.80E-03	2.167805645	32	41	3	21698	*HLA-B,HLA-DRB5,HLA-C*
GO:CC	endocytic vesicle membrane	GO:0030666	6.94E-03	2.15851091	193	41	5	21698	*HLA-B,TAP1,TAP2,HLA-DRB5,HLA-C*
GO:CC	MHC class I protein complex	GO:0042612	2.20E-02	1.657393853	8	41	2	21698	*HLA-B,HLA-C*
GO:CC	MHC class I peptide loading complex	GO:0042824	2.83E-02	1.548769783	9	41	2	21698	*TAP1,TAP2*
GO:CC	phagocytic vesicle	GO:0045335	3.17E-02	1.498324979	140	41	4	21698	*HLA-B,TAP1,TAP2,HLA-C*

GO:CC	ER to Golgi transport vesicle membrane	GO:0012507	3.88E-02	1.411255094	57	41	3	21698	*HLA-B,HLA-DRB5,HLA-C*
KEGG	Antigen processing and presentation	KEGG:04612	9.08E-12	11.04186369	67	23	9	7559	*HLA-B,TAP1,HLA-DQB1,TAP2,HLA-DRB5,HLA-DMA,HLA-C,HSPA1B,HLA-DOB*
KEGG	Type I diabetes mellitus	KEGG:04940	9.75E-10	9.0109275	40	23	7	7559	*LTA,HLA-B,HLA-DQB1,HLA-DRB5,HLA-DMA,HLA-C,HLA-DOB*
KEGG	Allograft rejection	KEGG:05330	3.15E-08	7.50197038	34	23	6	7559	*HLA-B,HLA-DQB1,HLA-DRB5,HLA-DMA,HLA-C,HLA-DOB*
KEGG	Graft-versus-host disease	KEGG:05332	5.41E-08	7.266800544	37	23	6	7559	*HLA-B,HLA-DQB1,HLA-DRB5,HLA-DMA,HLA-C,HLA-DOB*
KEGG	Staphylococcus aureus infection	KEGG:05150	2.18E-07	6.661046859	84	23	7	7559	*C4B,C2,HLA-DQB1,HLA-DRB5,HLA-DMA,C4A,HLA-DOB*
KEGG	Phagosome	KEGG:04145	2.34E-07	6.630580746	137	23	8	7559	*HLA-B,TAP1,HLA-DQB1,TAP2,HLA-DRB5,HLA-DMA,HLA-C,HLA-DOB*
KEGG	Autoimmune thyroid disease	KEGG:05320	3.18E-07	6.497649078	49	23	6	7559	*HLA-B,HLA-DQB1,HLA-DRB5,HLA-DMA,HLA-C,HLA-DOB*
KEGG	Viral myocarditis	KEGG:05416	6.52E-07	6.186080647	55	23	6	7559	*HLA-B,HLA-DQB1,HLA-DRB5,HLA-DMA,HLA-C,HLA-DOB*
KEGG	Epstein-Barr virus infection	KEGG:05169	3.22E-06	5.492245568	191	23	8	7559	*HLA-B,TAP1,HLA-DQB1,TAP2,HLA-DRB5,HLA-DMA,HLA-C,HLA-DOB*
KEGG	Systemic lupus erythematosus	KEGG:05322	3.97E-06	5.400909694	127	23	7	7559	*C4B,C2,HLA-DQB1,HLA-DRB5,HLA-DMA,C4A,HLA-DOB*
KEGG	Human T-cell leukemia virus 1 infection	KEGG:05166	6.99E-06	5.155703396	211	23	8	7559	*LTA,ATF6B,HLA-B,HLA-DQB1,HLA-DRB5,HLA-DMA,HLA-C,HLA-DOB*
KEGG	Asthma	KEGG:05310	6.91E-05	4.160615271	27	23	4	7559	*HLA-DQB1,HLA-DRB5,HLA-DMA,HLA-DOB*
KEGG	Cell adhesion molecules	KEGG:04514	2.02E-04	3.694105123	143	23	6	7559	*HLA-B,HLA-DQB1,HLA-DRB5,HLA-DMA,HLA-C,HLA-DOB*
KEGG	Intestinal immune network for IgA production	KEGG:04672	5.16E-04	3.287029975	44	23	4	7559	*HLA-DQB1,HLA-DRB5,HLA-DMA,HLA-DOB*
KEGG	Toxoplasmosis	KEGG:05145	9.00E-04	3.045979924	107	23	5	7559	*HLA-DQB1,HLA-DRB5,HLA-DMA,HSPA1B,HLA-DOB*
KEGG	Inflammatory bowel disease	KEGG:05321	1.92E-03	2.717109834	61	23	4	7559	*HLA-DQB1,HLA-DRB5,HLA-DMA,HLA-DOB*
KEGG	Leishmaniasis	KEGG:05140	3.50E-03	2.455916118	71	23	4	7559	*HLA-DQB1,HLA-DRB5,HLA-DMA,HLA-DOB*
KEGG	Th1 and Th2 cell differentiation	KEGG:04658	7.42E-03	2.129498764	86	23	4	7559	*HLA-DQB1,HLA-DRB5,HLA-DMA,HLA-DOB*

KEGG	Rheumatoid arthritis	KEGG:05323	7.42E-03	2.129498764	86	23	4	7559	*HLA-DQB1,HLA-DRB5,HLA-DMA,HLA-DOB*
KEGG	Hematopoietic cell lineage	KEGG:04640	9.25E-03	2.033998883	91	23	4	7559	*HLA-DQB1,HLA-DRB5,HLA-DMA,HLA-DOB*
KEGG	Th17 cell differentiation	KEGG:04659	1.55E-02	1.809758537	104	23	4	7559	*HLA-DQB1,HLA-DRB5,HLA-DMA,HLA-DOB*
KEGG	Human cytomegalovirus infection	KEGG:05163	2.49E-02	1.603892015	215	23	5	7559	*ATF6B,HLA-B,TAP1,TAP2,HLA-C*
REAC	Antigen Presentation: Folding, assembly and peptide loading of class I MHC	REAC:R-HSA-983170	6.88E-05	4.162432156	25	24	4	10770	*HLA-B,TAP1,TAP2,HLA-C*
REAC	Activation of C3 and C5	REAC:R-HSA-174577	1.00E-04	3.999555945	7	24	3	10770	*C4B,C2,C4A*
REAC	ER-Phagosome pathway	REAC:R-HSA-1236974	1.26E-02	1.899115798	90	24	4	10770	*HLA-B,TAP1,TAP2,HLA-C*
REAC	Antigen processing-Cross presentation	REAC:R-HSA-1236975	2.39E-02	1.620732475	106	24	4	10770	*HLA-B,TAP1,TAP2,HLA-C*
WP	Allograft rejection	WP:WP2328	5.88E-10	9.230469745	86	25	9	7364	*C4B,C2,HLA-B,HLA-DQB1,HLA-DRB5,HLA-DMA,C4A,HLA-C,HLA-DOB*
WP	Ebola virus infection in host	WP:WP4217	3.84E-04	3.415220066	126	25	6	7364	*HLA-B,HLA-DQB1,HLA-DRB5,HLA-DMA,HLA-C,HLA-DOB*
WP	Complement activation	WP:WP545	5.62E-03	2.250392449	21	25	3	7364	*C4B,C2,C4A*
TF	Factor: slug; motif: RRCAGGTGCA; match class: 1	TF:M12260_1	4.59E-02	1.338505383	27	40	3	19958	*LY6G6C,C6ORF25,NEU1*
HPA	appendix; non-germinal center cells[High]	HPA:0030443	5.29E-03	2.276740776	105	20	4	10976	*FOXP1,HLA-B,HLA-DQB1,HLA-DMA*

Supplementary Table S18. Pathways associated with headache and TSH.

source	term_name	term_id	adjusted_ p_value	negative_log10_ of_adjusted_ p_value	term_size	query_size	intersection_ size	effective_ domain_size	intersections
GO:MF	TAP binding	GO:0046977	4.80E-05	4.318441367	9	100	4	20183	*HLA-C,TAP1,TAP2,HLA-B*
GO:MF	peptide antigen binding	GO:0042605	3.93E-04	3.405555991	33	100	5	20183	*HLA-C,TAP1,TAP2,HLA-DRB1,HLA-B*
GO:MF	complement binding	GO:0001848	1.77E-03	2.75171302	20	100	4	20183	*CFB,C4B,C4A,PHB*
GO:BP	antigen processing and presentation of endogenous peptide antigen	GO:0002483	5.18E-05	4.285405765	19	100	5	21100	*HLA-C,TAP1,TAP2,HLA-DRB1,HLA-B*
GO:BP	antigen processing and presentation of peptide antigen	GO:0048002	2.68E-04	3.571369816	82	100	7	21100	*HLA-C,TAP1,TAP2,PSMB9,HLA-DRB1,HLA-DOB,HLA-B*
GO:BP	antigen processing and presentation of endogenous antigen	GO:0019883	2.86E-04	3.54423411	26	100	5	21100	*HLA-C,TAP1,TAP2,HLA-DRB1,HLA-B*
GO:BP	antigen processing and presentation of endogenous peptide antigen via MHC class I	GO:0019885	7.14E-04	3.14607405	13	100	4	21100	*HLA-C,TAP1,TAP2,HLA-B*
GO:BP	antigen processing and presentation of exogenous peptide antigen	GO:0002478	7.78E-04	3.108745394	59	100	6	21100	*HLA-C,TAP1,PSMB9,HLA-DRB1,HLA-DOB,HLA-B*
GO:BP	antigen processing and presentation of exogenous antigen	GO:0019884	2.17E-03	2.66309584	70	100	6	21100	*HLA-C,TAP1,PSMB9,HLA-DRB1,HLA-DOB,HLA-B*

GO:BP	antigen processing and presentation	GO:0019882	5.59E-03	2.252858946	128	100	7	21100	*HLA-C,TAP1,TAP2,PSMB9,HLA-DRB1,HLA-DOB,HLA-B*
GO:BP	antigen processing and presentation of peptide antigen via MHC class I	GO:0002474	7.59E-03	2.119478045	49	100	5	21100	*HLA-C,TAP1,TAP2,PSMB9,HLA-B*
GO:BP	antigen processing and presentation of exogenous peptide antigen via MHC class I	GO:0042590	3.35E-02	1.47459321	32	100	4	21100	*HLA-C,TAP1,PSMB9,HLA-B*
GO:CC	MHC protein complex	GO:0042611	1.62E-03	2.791324502	24	104	4	21698	*HLA-C,HLA-DRB1,HLA-DOB,HLA-B*
KEGG	Antigen processing and presentation	KEGG:04612	1.59E-05	4.798519832	67	49	7	7559	*HLA-C,TAP1,TAP2,TNF,HLA-DRB1,HLA-DOB,HLA-B*
KEGG	Allograft rejection	KEGG:05330	1.79E-04	3.748044795	34	49	5	7559	*HLA-C,TNF,HLA-DRB1,HLA-DOB,HLA-B*
KEGG	Graft-versus-host disease	KEGG:05332	2.76E-04	3.559439474	37	49	5	7559	*HLA-C,TNF,HLA-DRB1,HLA-DOB,HLA-B*
KEGG	Type I diabetes mellitus	KEGG:04940	4.10E-04	3.38692559	40	49	5	7559	*HLA-C,TNF,HLA-DRB1,HLA-DOB,HLA-B*
KEGG	Rheumatoid arthritis	KEGG:05323	1.37E-03	2.863739371	86	49	6	7559	*CTSK,TNF,LTB,HLA-DRB1,HLA-DOB,TGFB3*
KEGG	Epstein-Barr virus infection	KEGG:05169	2.12E-03	2.673622433	191	49	8	7559	*STAT1,HLA-C,TAP1,TAP2,TNF,HLA-DRB1,HLA-DOB,HLA-B*
KEGG	Inflammatory bowel disease	KEGG:05321	3.35E-03	2.475017815	61	49	5	7559	*STAT1,TNF,HLA-DRB1,HLA-DOB,TGFB3*
KEGG	Human T-cell leukemia virus 1 infection	KEGG:05166	4.33E-03	2.36388739	211	49	8	7559	*CDKN2C,HLA-C,TNF,HLA-DRB1,HLA-DOB,HLA-B,MAD1L1,TGFB3*
KEGG	Leishmaniasis	KEGG:05140	6.98E-03	2.155955395	71	49	5	7559	*STAT1,TNF,HLA-DRB1,HLA-DOB,TGFB3*
KEGG	Staphylococcus aureus infection	KEGG:05150	1.55E-02	1.808403967	84	49	5	7559	*CFB,C4B,HLA-DRB1,C4A,HLA-DOB*
KEGG	Phagosome	KEGG:04145	1.86E-02	1.72960932	137	49	6	7559	*HLA-C,TAP1,TAP2,HLA-DRB1,HLA-DOB,HLA-B*
KEGG	Autoimmune thyroid disease	KEGG:05320	2.12E-02	1.673282139	49	49	4	7559	*HLA-C,HLA-DRB1,HLA-DOB,HLA-B*

KEGG	Coronavirus disease - COVID-19	KEGG:05171	2.69E-02	1.570909392	207	49	7	7559	*STAT1,CFB,C4B,TNF,C4A,RPL6,RPL27*
KEGG	Viral myocarditis	KEGG:05416	3.32E-02	1.478928742	55	49	4	7559	*HLA-C,HLA-DRB1,HLA-DOB,HLA-B*
KEGG	Toxoplasmosis	KEGG:05145	4.79E-02	1.319850144	107	49	5	7559	*STAT1,TNF,HLA-DRB1,HLA-DOB,TGFB3*
REAC	Activation of C3 and C5	REAC:R-HSA-174577	2.65E-03	2.577121079	7	63	3	10770	*CFB,C4B,C4A*
REAC	Antigen Presentation: Folding, assembly and peptide loading of class I MHC	REAC:R-HSA-983170	4.95E-03	2.305797809	25	63	4	10770	*HLA-C,TAP1,TAP2,HLA-B*
WP	Allograft rejection	WP:WP2328	2.75E-05	4.560348973	86	55	8	7364	*STAT1,HLA-C,C4B,TNF,HLA-DRB1,C4A,HLA-DOB,HLA-B*
WP	Type II interferon signaling	WP:WP619	8.67E-04	3.062002411	36	55	5	7364	*STAT1,TAP1,PSMB9,HLA-B,PTPN11*
HP	Decreased serum complement C4	HP:0045042	2.20E-02	1.657953867	7	38	3	4508	*C4B,C4A,SERPING1*
HP	Unusual infection by anatomical site	HP:0032158	2.79E-02	1.554400479	107	38	7	4508	*STAT1,CFB,C4B,HLA-DRB1,C4A,HLA-B,MVK*

Supplementary Table S19. Pathways associated with headache and fT4.

**source**	**term_name**	**term_id**	**adjusted_p_value**	**negative_log10_of_adjusted_p_value**	**term_size**	**query_size**	**intersection_size**	**effective_domain_size**	**intersections**
GO:MF	sialic acid transmembrane transporter activity	GO:0015136	8.38E-04	3.076646377	5	105	3	20183	*SLC17A4,SLC17A1,SLC17A2*
GO:MF	sodium:phosphate symporter activity	GO:0005436	1.35E-02	1.869049782	11	105	3	20183	*SLC17A4,SLC17A1,SLC17A2*
GO:BP	sialic acid transport	GO:0015739	2.35E-03	2.628446438	5	106	3	21100	*SLC17A4,SLC17A1,SLC17A2*
GO:BP	positive regulation of	GO:2000427	1.30E-02	1.885034901	8	106	3	21100	*C4B,C2,C4A*

	apoptotic cell clearance								
GO:BP	regulation of apoptotic cell clearance	GO:2000425	2.77E-02	1.557224348	10	106	3	21100	*C4B,C2,C4A*
KEGG	Systemic lupus erythematosus	KEGG:05322	1.23E-05	4.91151414	127	38	8	7559	*HIST1H2AC,TNF,C4B,HIST1H4J,C2,HIST1H2BN, HIST1H2BD,C4A*
KEGG	Coronavirus disease - COVID-19	KEGG:05171	3.91E-02	1.407377393	207	38	6	7559	*TNF,C4B,CFB,C2,C4A,CHUK*
KEGG	Pertussis	KEGG:05133	4.04E-02	1.393392048	76	38	4	7559	*TNF,C4B,C2,C4A*
KEGG	Human T-cell leukemia virus 1 infection	KEGG:05166	4.33E-02	1.363258403	211	38	6	7559	*CDKN2C,LTA,TNF,ATF6B,HLA-B,CHUK*
KEGG	Alcoholic liver disease	KEGG:04936	4.84E-02	1.315450686	141	38	5	7559	*TNF,C4B,C2,C4A,CHUK*
REAC	Activation of C3 and C5	REAC:R-HSA-174577	7.21E-06	5.14184651	7	55	4	10770	*C4B,CFB,C2,C4A*
REAC	Senescence-Associated Secretory Phenotype (SASP)	REAC:R-HSA-2559582	7.25E-03	2.139606019	112	55	6	10770	*CDKN2C,HIST1H2AC,EHMT2,HIST1H4J,HIST1H2BN, HIST1H2BD*
REAC	Defective pyroptosis	REAC:R-HSA-9710421	1.14E-02	1.943168931	73	55	5	10770	*HIST1H2AC,HIST1H4J,HIST1H2BN,HIST1H2BD,PRIM1*
REAC	ERCC6 (CSB) and EHMT2 (G9a) positively regulate rRNA expression	REAC:R-HSA-427389	1.39E-02	1.858308075	76	55	5	10770	*HIST1H2AC,EHMT2,HIST1H4J,HIST1H2BN,HIST1H2BD*
REAC	DNA Damage/Telomere Stress Induced Senescence	REAC:R-HSA-2559586	1.78E-02	1.750673378	80	55	5	10770	*HIST1H2AC,HIST1H4J,HIST1H2BN,HIST1H2BD,HIST1H1E*
REAC	Cellular Senescence	REAC:R-HSA-2559583	1.91E-02	1.719492354	195	55	7	10770	*CDKN2C,HIST1H2AC,EHMT2,HIST1H4J,HIST1H2BN, HIST1H2BD,HIST1H1E*

REAC	Packaging Of Telomere Ends	REAC:R-HSA-171306	4.71E-02	1.327434054	52	55	4	10770	*HIST1H2AC,HIST1H4J,HIST1H2BN,HIST1H2BD*
WP	Complement activation	WP:WP545	1.41E-03	2.85002153	21	50	4	7364	*C4B,CFB,C2,C4A*
WP	Allograft rejection	WP:WP2328	3.70E-02	1.431779931	86	50	5	7364	*TNF,C4B,C2,HLA-B,C4A*
